# Emerging Invasion Risks of Non-Native Urban Trees in Continental Europe Under a Changing Climate

**DOI:** 10.3390/plants15152313

**Published:** 2026-07-28

**Authors:** Mihaela Britvec, Marina Piria, Ivana Vitasović-Kosić, S. Luke Flory, Božena Mitić, Sara Essert, Dario Hruševar, Seokmin Kim, Ivica Ljubičić, Lorenzo Vilizzi

**Affiliations:** 1 Faculty of Agriculture University of Zagreb, Department of Agricultural Botany, 10000 Zagreb, Croatia; mbritvec@agr.hr (M.B.); ivitasovic@agr.hr (I.V.-K.); iljubicic@agr.hr (I.L.); 2 Faculty of Agriculture University of Zagreb, Department of Fisheries, Apiculture, Wildlife Management and Special Zoology, 10000 Zagreb, Croatia; 3 Department of Ecology and Vertebrate Zoology, Faculty of Biology and Environmental Protection, University of Lodz, 90-237 Lodz, Poland; lorenzo.vilizzi@gmail.com; 4 Agronomy Department, University of Florida, Gainesville, FL 32611-0500, USA; flory@ufl.edu (S.L.F.); kimseokmin@ufl.edu (S.K.); 5 Invasion Science Institute, University of Florida, Gainesville, FL 32611-0410, USA; 6 Faculty of Science University of Zagreb, Department of Biology, Division of Botany, 10000 Zagreb, Croatia; bozena.mitic@biol.pmf.hr (B.M.); sara.essert@biol.pmf.hr (S.E.); dario.hrusevar@biol.pmf.hr (D.H.)

**Keywords:** climate change, invasive plants, Terrestrial Plant Species Invasiveness Screening Kit (TPS-ISK), urban green areas

## Abstract

Urban green areas contain numerous non-native tree species that may escape from cultivation and potentially become invasive. Climate change is expected to exacerbate this risk by creating favourable conditions for species that are currently climatically restricted. Here, we provide a comprehensive risk screening of 34 non-native urban tree species in continental Europe under current and projected future climate scenarios using the Terrestrial Plant Species Invasiveness Screening Kit (TPS-ISK). Under current conditions, 10 species (29.4%) were categorized as high risk, 23 (67.6%) as medium risk, and one (2.9%) as low risk. Under the projected climate change, 11 species were classified as high risk, including seven categorized as very high risk. *Ailanthus altissima*, *Diospyros virginiana*, and *Quercus rubra* consistently ranked among the highest-risk species, while *Acer tataricum* subsp. *ginnala*, *Koelreuteria paniculata*, *Magnolia kobus*, *Phellodendron amurense*, *Pseudotsuga menziesii*, and *Robinia pseudoacacia* showed an increased invasion potential under future climate conditions. These findings indicate that climate change may facilitate the establishment and spread of several currently cultivated urban tree species. Our study provides the first comprehensive TPS-ISK screening of non-native urban trees for mainland Europe and identifies priority species for early detection, monitoring, and management.

## 1. Introduction

More than half of the world’s population (over 4 billion people) lives in urban areas (i.e., cities, towns, and suburbs), a proportion that is expected to increase to 68% by 2050 [1]. Parks and other urban green areas provide aesthetic and cultural services to people and have positive effects on human health and well-being [2,3]. They also play an important role in providing environmental services (e.g., by reducing the impact of urban heat islands and improving air quality) and ecological services (e.g., by providing habitats for urban wildlife and biodiversity conservation) [4]. However, the planted ornamental flora in urban green areas frequently contains a large number of non-native species [5], which may escape cultivation and invade surrounding natural areas.

Ornamental horticulture is the most important pathway for non-native plant introductions worldwide [6,7,8,9]. While most of these plant species can only survive where planted under specific management practices (e.g., watering, and nutrition), others escape from cultivation [10]. After a potential escape, they face the barriers of the naturalization and invasion processes [11], while functional traits of these species [12,13] as well as climatic and environmental factors interact to influence their success and ability to disperse [14]. Among functional traits, a long flowering period, large height, seed mass, total biomass, high germination rate, and dispersal ability, as well as high stress tolerance enhance the invasion potential of plant species [11]. These characteristics are also typical for species used in horticulture because they facilitate plant establishment and growth in managed settings [14]. Given the increasing urbanization and the constant rise of horticultural practices that favour managed parks and residential gardens, the risk of ornamental plant escape to natural habitats is increasing [15].

Since dozens of ornamental plants in temperate regions come from warmer areas [16], these species often survive and grow in horticultural environments but do not establish self-sustaining populations in natural areas. In other words, these non-native ornamental plants are currently still outside their realized climatic niches but are inside their tolerance climatic niches [14]. Thus, under climate change [17], these plants may encounter more suitable environmental conditions on a larger scale, increasing the chance of escaping cultivation and becoming naturalized [18], and possibly invasive [10,18,19,20,21,22,23,24,25,26].

The early identification of non-native species with invasive potential is critical, as even a single invasive species can significantly disrupt biodiversity and cause substantial economic losses [27]. Within risk analysis frameworks, the first step, namely, risk identification, focuses on determining which species are most likely to become invasive in a given region [28]. To support this process, electronic decision-support tools have been developed, among which the Aquatic Species Invasiveness Screening Kit (AS-ISK) has become widely applied [29,30,31]. Building on this approach, AS-ISK was adapted into the Terrestrial Plant Species Invasiveness Screening Kit (TPS-ISK) [32], which currently represents the state-of-the-art tool for global application in assessing plant invasion risk.

Although non-native plants have been studied in some urban areas [10,19,24,25,33,34,35,36,37], an understanding of the risks of invasiveness of urban trees species in continental Europe is lacking. This research gap is crucial given the challenge posed by climate change, which may increase the invasion risk of certain ornamental species, thereby posing an increasing threat to biodiversity [38].

In this study, we focus on urban tree species because they are among the most problematic plant invaders worldwide [39,40,41] and some of them are considered to be the most harmful invasive species in urban systems [26,42]. Our goal is to provide the first detailed information on the invasion risk of non-native urban trees species in continental Europe under current and future climate conditions. We hypothesize that the changing climate will increase the invasion risk of these species. In this way, we aim to provide insights into the drivers of non-native species invasions in continental Europe, thereby informing management strategies and contributing to the protection of local ecosystems while mitigating potential economic losses.

## 2. Results

For all tree species, the ROC curve resulted in an AUC of 0.6947 (0.5019–0.8876 95% CI) and a threshold of 41. For the angiosperms, the ROC curve resulted in an AUC of 0.5893 (0.3548–0.8238 95% CI) and, again, in a threshold of 41. The AUC values were, overall, within the acceptable limits for the discriminatory power of the test [43]. This threshold was, therefore, used for the calibration of the risk outcomes to distinguish between medium-risk and high-risk species (Table 1; refer to the Supplementary Online Material S1 for reports of the 34 screened species).

**Figure 1:**
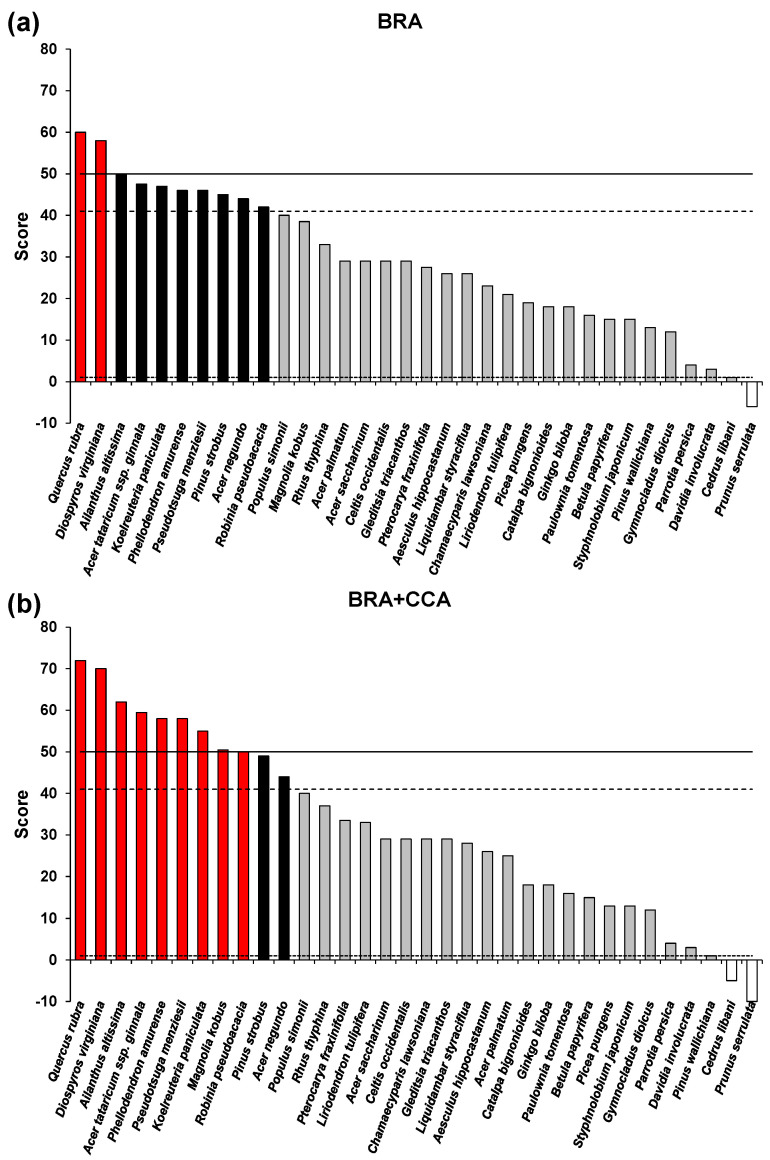
Risk outcome scores for the non-native urban trees screened with the Terrestrial Plant Species Invasiveness Screening Kit (TPS-ISK) for Continental Europe: (**a**) Basic Risk Assessment (BRA) scores; and (**b**) BRA + Climate Change Assessment (BRA + CCA) scores. Red bars = very high-risk species; black bars = high-risk species; grey bars = medium-risk species; white bars = low-risk species; solid line = very high-risk threshold; hatched line = high-risk threshold; and dotted line = medium-risk threshold. Thresholds as per Table 1.

Based on the BRA scores (Table 1, Figure 1a), 10 (29.4%) species were ranked as high risk, 23 (67.6%) as medium risk, and 1 (2.9%) as low risk. Of the 15 species categorized a priori as invasive, 8 were ranked as high risk [true positives: box elder (*Acer negundo*), tree of heaven (*Ailanthus altissima*), American persimmon (*Diospyros virginiana*), goldenrain tree (*Koelreuteria paniculata*), Eastern white pine (*Pinus strobus*), Douglas fir (*Pseudotsuga menziesii*), Northern red oak (*Quercus rubra*), and black locust (*Robinia pseudoacacia*)] and 1 as low risk [false negative: Japanese cherry (*Prunus serrulata*)]. Of the 19 species categorized a priori as non-invasive, 2 were ranked as high risk [false positives: Amur Maple (*Acer tataricum* ssp. *ginnala*), and Amur cork tree (*Phellodendron amurense*)]. Of the 23 medium-risk species, 17 were a priori non-invasive and 6 invasive.

Based on the BRA + CCA scores (Table 1, Figure 1b), 11 (32.4%) species were ranked as high risk, 21 (61.8%) as medium risk, and 2 (5.9%) as low risk. The ranking of the a priori invasive species was the same as per the BRA. Of the a priori non-invasive species, three were ranked as high risk [same species as per BRA plus Kobus magnolia (*Magnolia kobus*)] and one as low risk (same species as per BRA). Of the 21 medium-risk species, 15 were a priori non-invasive and 6 invasive.

Based on an ad hoc threshold ≥ 50, there were two very high-risk species for the BRA and BRA + CCA (*Diospyros virginiana* and *Quercus rubra*), and seven for the BRA + CCA only [Amur maple (*Acer tataricum* ssp. *ginnala*), *Ailanthus altissima*, goldenrain tree (*Koelreuteria paniculata*), Kobus magnolia (*Magnolia kobus*), Amur cork tree (*Phellodendron amurense*), Douglas fir (*Pseudotsuga menziesii*), and black locust (*Robinia pseudoacacia*)] (Table 1, Figure 1a,b). The CCA resulted in an increase in the BRA score (cf. BRA + CCA score) for 15 (44.1%) species, in no change for 13 (38.2%), and in a decrease for 6 (17.6%) (Table 1).

The mean CF_Total_ was 0.616 ± 0.020 SE, the mean CF_BRA_ 0.635 ± 0.020 SE, and the mean CFCCA 0.463 ± 0.032 SE. The mean CF_BRA_ was higher than the mean CF_CCA_ (F_{1, 126} = 0.94, *p* = 0.333).

## 3. Discussion

### 3.1. Risk Outcomes

Overall, the present results indicate that the risk screening satisfactorily differentiated species by invasion potential, with most species classified as medium risk and about one-quarter as high risk. The model’s performance metrics suggest a moderate predictive accuracy in distinguishing between medium- and high-risk species. The BRA and BRA + CCA models consistently ranked known invasive species such as *Ailanthus altissima*, *Diospyros virginiana*, and *Quercus rubra* among the highest risk categories. The CCA component slightly increased the risk scores for several species, emphasizing how future climatic conditions may heighten invasion risks.

Our findings are consistent with earlier woody-plant risk-screening studies based on WRA-type approaches. In particular, species identified as high risk, including *Ailanthus altissima*, *Acer negundo*, *Pinus strobus*, *Quercus rubra*, and *Robinia pseudoacacia*, were similarly identified as potentially invasive in Central European applications of the original WRA and WRA + Daehler frameworks [46,47]. In contrast, earlier decision-tree approaches such as the Reichard and Hamilton model [48] were shown to be less transferable across regions because they overemphasized invasiveness elsewhere without adequately accounting for the climatic suitability in Central Europe [47]. The consistency between our outcomes and previous WRA-based screenings suggests that contemporary ISK-type tools retain the core predictive strengths of earlier WRA tools while additionally incorporating climate-change-related invasion dynamics. Unlike previous studies based on the WRA and WRA + Daehler frameworks, which focused primarily on risk screening under current climatic conditions, our study explicitly incorporates future climate change scenarios. This approach enables the identification of species whose invasion potential may increase under projected warming conditions, thereby providing an early-warning framework for the management of non-native trees in continental Europe. This is particularly relevant for urban trees, as future warming may shift currently climatically constrained species into higher-invasion-risk categories.

*Ailanthus altissima*, native to northeast and central China and Taiwan, represents one of the most widespread invasive tree species globally [49]. First introduced to Europe in the 18th century, it has since spread rapidly due to its resilience to herbivory, tolerance of urban conditions, and popularity as an urban and avenue tree [49,50,51,52]. Today, *A. altissima* colonizes both heavily disturbed urban environments and semi-natural habitats, including abandoned industrial sites, roadsides, riverbanks, forest edges, and agricultural margins [53]. Its invasive success is attributed to its prolific seed production [49,54], root suckering [55], rapid growth, early maturity [56], broad environmental tolerance [57], allelopathic effects [58], and release from natural enemies [59]. These traits, combined with anthropogenic disturbance and climate change, strongly promote persistence and spread [60]. While temperature thresholds constrain establishment in cooler regions and at higher altitudes (>900 m a.s.l.) [61], the climatic overlap between its native range and southern Europe suggests continued expansion [52].

*Diospyros virginiana*, native to the eastern and southeastern United States [62], has so far shown limited spread in Europe, where it was introduced in the late 19th and early 20th centuries for ornamental and fruit purposes [63,64,65]. In Croatia, its presence dates to 1859, when it was planted in the Trsteno Arboretum [66]. While natural regeneration is mostly restricted to abandoned and ruderal sites [67], its traits—high seed viability, endozoochoric dispersal by birds and mammals, tolerance of drought and poor soils, and deep taproot systems [68]—indicate resilience. Climate change may amplify its potential by reducing frost damage and enabling establishment in formerly unsuitable regions [69]. Although its invasive behaviour remains localized, projections suggest that warmer conditions, as present in the Mediterranean region, may elevate its risk from locally naturalized to regionally established [67].

*Quercus rubra* was introduced from North America in 1691 and is now widespread across Europe, covering more than 350,000 ha, particularly in France, Germany, Poland, Hungary, and Slovenia [70,71]. Initially valued as an urban tree, its use later expanded to forestry and reforestation due to its rapid growth, high-quality timber, and ecological flexibility [72]. Compared with native oaks (*Quercus robur* and *Q. petraea*), *Quercus rubra* exhibits a higher tolerance to drought and other environmental stresses, enhancing its competitiveness under climate change [70,73]. Its regeneration relies both on acorn dispersal by animals (up to 1.5 km from parent trees) and its strong sprouting ability [70,72]. Given these advantages, *Quercus rubra* is projected to increasingly replace the declining native oak species in forests in the future [72].

Other species scored as having a high invasion risk, including *Acer negundo*, *A. tataricum* ssp. *ginnala*, *Koelreuteria paniculata*, *Phellodendron amurense*, *Pinus strobus*, *Pseudotsuga menziesii*, and *Robinia pseudoacacia*, were also found to pose significant risks under both present and future climate conditions. *Acer negundo* and *A. tataricum* ssp. *ginnala* spread via wind- and water-dispersed samaras, thrive on a wide range of soils, and display strong drought tolerance, making them successful colonizers of urban and disturbed habitats [74,75,76,77,78,79]. *Koelreuteria paniculata* and *Phellodendron amurense* are at the earlier stages of naturalization, but their reproductive strategies and climate adaptability highlight future risks [66,80,81]. *Pseudotsuga menziesii*, while valued in forestry, can regenerate naturally and alter the soil biota [20,82]. *Pinus strobus* and *Robinia pseudoacacia* are already invasive in several European countries, with the latter exerting particularly strong ecosystem impacts through nitrogen fixation, allelopathy, and competitive stand formation [83,84,85].

Overall, these findings indicate that a combination of ecological traits, reproductive strategies, and increasing climatic suitability drives the invasion potential of both currently widespread and still-emerging species. While *Ailanthus altissima*, *Diospyros virginiana*, and *Quercus rubra* present the highest immediate risks, several other taxa require close monitoring to prevent future large-scale impacts on European biodiversity. Several high-risk species identified in this study are widely planted in urban parks (e.g., *Acer negundo*), while others are extensively used in forestry plantations (e.g., *Pseudotsuga menziesii*). However, propagules of these species can spread beyond managed sites into adjacent unmanaged habitats, where they may establish self-sustaining populations in the absence of regular control [5,25]. Future trait-based analyses could help identify the combinations of reproductive and functional characteristics associated with an increased invasion risk under changing climatic conditions.

### 3.2. Climate Change

Climate change is expected to play a central role in shaping the invasive potential of non-native tree species in Europe. Climate change is often discussed in the context of rising mean temperatures; however, it encompasses a broader set of environmental changes, including shifts in precipitation regimes, increased frequency and intensity of extreme events, altered seasonality, and changes in climatic variability [86,87]. These multiple dimensions can interact with land use, disturbance, and biotic interactions to influence species performance and distribution in complex and sometimes non-linear ways [88]. In this study, climate-change effects are incorporated in a simplified manner that aligns with the screening-level objective of the method. The approach used in this study provides an overall estimate of the invasion risk rather than a process-based representation of individual climatic drivers. Consequently, it does not disentangle the roles of temperature, precipitation, and greenhouse gas emissions, or their interactions, and the results of this study should be interpreted as a first-pass assessment and complemented by spatially explicit analyses for management purposes.

Climate-change impacts vary substantially across Europe, which is climatically heterogeneous [89]. Species may benefit from changing conditions in some regions, such as *Ailanthus altissima* for southern Europe, while they may experience reduced suitability and range contraction elsewhere [60]. The modelling studies for *Ailanthus altissima* suggest that warmer and drier conditions will support its persistence and expansion, particularly in drought-prone habitats [60]. Although its spread is still limited by temperature at higher altitudes (>900 m a.s.l.) and in colder regions [61], the overlap between climatic conditions in its native range and those of southern Europe indicates considerable scope for further range expansion [52].

*Diospyros virginiana* may also benefit from ongoing climate change. Rising winter temperatures and longer growing seasons could reduce frost damage, improve seedling survival, and open up regions that were previously unsuitable for its establishment [67,69]. Its inherent drought tolerance and capacity to persist on nutrient-poor soils further increase its resilience under future climate stress. For *Quercus rubra*, warming scenarios may strengthen its competitive advantage over native European oaks. Experimental studies indicate that its greater drought tolerance compared with *Quercus robur* and *Q. petraea* could allow it to replace declining native populations in future European forests [72,73].

These dynamics are not limited to the three highest scored species. Climate change is also predicted to enhance the establishment of other non-native trees currently present in Europe. For example, warmer conditions and longer growing seasons may increase seed viability and colonization success in *Koelreuteria paniculata* [90], while facilitating the spread of *Phellodendron amurense* beyond its native climatic range [80]. Moreover, future climatic conditions will likely favour the occurrence of *Robinia pseudoacacia* in central and northeastern Europe where this species is still absent or relatively rare [91]. Similarly, the relatively high drought tolerance of both *Pseudotsuga menziesii* and *Robinia pseudoacacia* may enhance their competitiveness in European forests under future conditions, particularly as droughts become more frequent and severe [89,92].

Urban centres are consistently warmer and often have longer growing seasons than nearby rural areas, which alters the species phenology and thermal regimes relevant to establishment [93,94]. In European cities, urban heat island intensities can exceed ~8 °C under certain conditions, and urbanization has been shown to extend growing seasons relative to the surrounding rural areas, with climatic influences detectable well beyond the urban core along urban–rural gradients [95,96]. Such conditions may facilitate the establishment of cultivated ornamental species, including urban trees, by reducing climatic constraints, particularly the cold limitation, and by exposing populations to warmer and more variable conditions than those in surrounding landscapes [97]. As a result, urban environments may act as the initial establishment foci and potential pre-adaptation zones, enabling species to persist beyond cultivation and increasing the likelihood of subsequent spread as regional climates warm [94]. Although these urban-specific effects are not explicitly captured in our analysis, they highlight an additional pathway through which climate change and land use may interact to influence invasion risk. Future studies including a broader representation of gymnosperm taxa would also provide an opportunity to evaluate whether separate calibration thresholds are warranted.

Overall, climate change, particularly in urban centres, is likely to increase colonization opportunities for a wide range of non-native tree species. While it may amplify the already substantial invasive potential of *Ailanthus altissima* and *Quercus rubra*, it could also elevate species such as *Diospyros virginiana*, *Koelreuteria paniculata*, and *Phellodendron amurense* from locally naturalized or regionally constrained taxa to more widespread and ecologically significant invaders.

The present assessment should therefore be interpreted as a continental-scale screening rather than as a prediction of invasion risk within individual European regions. Consequently, individual species are expected to differ in their establishment success and invasion potential among regions according to local climatic and environmental conditions. These regional differences should be addressed through dedicated species distribution and climatic suitability analyses.

### 3.3. Management Considerations

The management of non-native tree species in Europe remains highly context-dependent, reflecting both ecological risks and socio-economic values. The EU List of Invasive Alien Species of Union concern, established under Regulation (EU) 1143/2014 and its subsequent amendments, represents the primary legislative framework for coordinated management. However, only a limited number of tree taxa are currently included. For example, *Ailanthus altissima* was added in 2019 under Commission Implementing Regulation (EU) 2019/1262 [98], following widespread evidence of its aggressive spread and ecological impacts.

By contrast, several other non-native tree species with a documented invasive potential remain excluded from the Union list. *Quercus rubra*, which is considered invasive or potentially invasive by some authors and official bodies [99], is not included in the Union list despite widespread plantings across Europe (European Union Regulation 2022/1203). *Acer tataricum* ssp. *ginnala* illustrates a comparable case: while it is an emerging invasive threat in North America [100] and has been classified as high-risk in Latvia [101], it has not been recognized as a species of Union concern [98]. *Diospyros virginiana* presents a different challenge. Its spread is still limited, but local escapes, for example, in Croatia [67], demonstrate the importance of early detection and monitoring. Restricting its further introduction into vulnerable ecosystems and promoting awareness among horticultural stakeholders may prevent future invasiveness.

The same uncertainty applies to species such as *Koelreuteria paniculata*, *Phellodendron amurense*, *Pseudotsuga menziesii*, *Pinus strobus*, and *Robinia pseudoacacia*. Although they are not currently listed at the Union level, evidence of local naturalization and ecological impacts suggests the need for proactive monitoring and risk assessment [85,102]. Conversely, some species assessed in this work, including *Ginkgo biloba* (Endangered, EN), *Aesculus hippocastanum* (Vulnerable, VU), *Cedrus libani* (Vulnerable, VU), and *Pterocarya fraxinifolia* (Vulnerable, VU), are globally threatened within their native ranges. Consequently, cultivated populations outside their native ranges may provide supplementary ex situ conservation value, although this depends on their genetic provenance and should not preclude appropriate management where these species pose invasion risks [103].

In conclusion, these examples highlight the need for continuous monitoring and risk assessment, even for species not yet included in the EU List of Invasive Alien Species of Union Concern. Particular attention should be paid to taxa that are still freely available in the horticultural trade (*Koelreuteria paniculata*, *Phellodendron amurense*), since urban plantings often act as stepping stones for the subsequent escape into semi-natural and protected habitats. Integrating the ecological, climatic, and socio-economic perspectives will be essential in determining the management priorities and mitigating the potential long-term impacts on European biodiversity.

## 4. Materials and Methods

### 4.1. Species Selection

In this study, continental Europe refers to mainland Europe used as the risk assessment area for TPS-ISK screening, excluding island systems with distinct biogeographical and invasion dynamics. The assessment considers mainland Europe as a single screening area rather than evaluating invasion risk separately for individual countries or regions. The list of urban tree species was compiled to support a screening-level of invasion risk for non-native urban trees cultivated in European urban green areas, using continental Europe as the risk assessment area. The species selection was compiled based on three criteria: (1) occurrence in urban park inventories from Zagreb, Croatia, used as a representative continental European source area, compiled from field surveys conducted in 2023–2024 [104]; (2) documented cultivation in European urban horticulture according to the Royal Horticultural Society Encyclopedia of Plants and Flowers [105]; and (3) confirmed non-native status in continental Europe based on the Euro + Med Plantbase (https://europlusmed.org/) and the Global Biodiversity Information Facility (GBIF: https://www.gbif.org/) databases. The Zagreb inventory was used solely to identify candidate non-native urban tree taxa for screening and was not intended to represent the composition of urban tree floras across mainland Europe. The subsequent risk screening was conducted for the predefined mainland European risk assessment area. The source area is characterized by a temperate continental climate and extensive urban green infrastructure, supporting a diverse assemblage of ornamental tree species representative of those commonly cultivated across European cities. To ensure continental relevance, taxa were retained only if they had documented use in European horticulture and were verified as non-native to continental Europe.

In total, 34 taxa (33 species and 1 subspecies) of urban trees were selected, representing some of the more commonly planted trees in European cities [106], and were, therefore, considered representative for a continental-scale risk screening (Table 2). Taxonomic nomenclature follows the Catalogue of Life Annual Checklist 2025 (https://www.catalogueoflife.org/), the International Plant Names Index (http://www.ipni.org), and Plants of the World Online (https://powo.science.kew.org/). All taxa are hereafter referred to as species.

### 4.2. Risk Screening

Risk screening was undertaken with TPS-ISK v2.4.1 (download at https://tinyurl.com/ISK-toolkits, accessed on 8 April 2024). This taxon-generic decision support tool complies with the ‘minimum standards’ for screening non-native species under EC Regulation No. 1143/2014 on the prevention and management of the introduction and spread of invasive alien species. The TPS-ISK consists of 55 questions, of which 49 comprise the Basic Risk Assessment (BRA) and 6 the Climate Change Assessment (CCA). The BRA addresses the biogeography/invasion history and biology/ecology of the species, and the CCA requires the assessor to predict how future predicted climatic conditions are likely to affect the BRA with respect to risks of introduction, establishment, dispersal, and impact. The CCA component is considered an ongoing and cumulative process influencing species performance and spread across Europe.

Screenings were conducted separately by eight assessors (B.M., D.H., I.LJ., I.V.K., M.B., S.E., S.K., and S.L.F.), with each assessor screening from two to nine species. All assessors used the same fixed TPS-ISK protocol. The completed screenings were then subjected to quality control by M.P. and L.V. All assessors are specialists in the biology and ecology of the urban trees under study. In this respect, ISK screenings do not represent unstructured expert opinion, but a standardized expert elicitation process governed by fixed questions, predefined scoring ranges, confidence rankings, and statistically calibrated risk thresholds. The tool constrains assessor input within a reproducible decision framework, thereby minimizing individual bias. When applied by trained assessors with taxon-specific expertise, this approach has repeatedly demonstrated high internal consistency and transferability across regions and taxa [45]. Accordingly, the use of a single trained assessor per species represents an accepted and validated application of the methodology. Previous applications of the ISK family of toolkits have demonstrated high internal consistency across assessors when applied by trained experts, supporting the robustness of single-assessor screenings [45].

Following the protocol by Vilizzi et al. [45], the assessor provided a response for each question, a confidence level, and a justification [107]. Upon completion of a screening, two outcome scores are obtained: BRA and BRA + CCA. Scores < 1 rank the species as ‘low risk’, whereas scores ≥ 1 rank the species as ‘medium risk’ or ‘high risk’. The distinction between medium and high risk is based on a calibrated threshold obtained by Receiver Operating Characteristic (ROC) curve analysis [28,45]. This approach is subject to the availability of a representative number of species (at least 15–20) categorized a priori as either invasive or non-invasive in a relatively balanced proportion [45]. Given that this criterion was satisfied for the angiosperms but not for the gymnosperms (Table 2), an overall threshold was computed for all urban trees and a separate one for the angiosperms. Additionally, an ad hoc threshold (as per Britton et al.) [108] was used to distinguish ‘very high-risk’ species within those ranked as having a high risk.

Fitting of the ROC curves was carried out with pROC [109] for R x64 v4.4.0 [110]. Permutational ANOVA with normalization of the data was used to test for differences in the confidence factor (see Vilizzi et al.) [45] between the BRA and BRA + CCA; this used a Bray–Curtis dissimilarity measure, and 9999 unrestricted permutations of the raw data, with statistical effects evaluated at α = 0.05. Following identification of the threshold score, evaluation of the risk classifications to identify false-positive and false-negative rankings was not applied to the medium-risk species because their further evaluation in a follow-up risk assessment depends on management priorities and the financial availability. All screenings were carried out under the supervision of L.V., who was responsible for quality control of the biological/ecological data and of the methodological (i.e., database-related) aspects of the study.

## Figures and Tables

**Table 1:** Non-native urban trees (origin: WFO, 2025) [44] screened using the Terrestrial Plant Species Invasiveness Screening Kit (TPS-ISK) for their invasion risk in Continental Europe. *A priori* categorization (Outcome: N = non-invasive; Y = invasive) follows the four-step protocol of Vilizzi et al. [45]: (1) Global Biodiversity Information Facility (https://www.gbif.org/, accessed on 15 July 2025); (2) Global Invasive Species Database (GISD: www.iucngisd.org); (3) European Alien Species Information Network (EASIN: https://easin.jrc.ec.europa.eu/easin, accessed on 15 July 2025); and (4) Google Scholar literature search. N = no impact/threat; Y = impact/threat; ‘–’ = absent; n.a. = not applicable.

			*A priori* Categorization				
Taxon Name	Common Name	Origin	GBIF	GISD	EASIN	GScholar	Outcome
Angiosperms							
*Acer negundo* L.	box elder	Central and Eastern North America	Y	–	Y	n.a.	Y
*Acer palmatum* Thunb.	Japanese maple	Asia (Japan, Korea)	N	–	N	N	N
*Acer saccharinum* L.	silver maple	Eastern North America	N	–	N	N	N
*Acer tataricum* ssp. *ginnala* (Maxim.) Wesm.	Amur maple	Asia (China, Japan)	N	–	N	N	N
*Aesculus hippocastanum* L.	horse chestnut	Southeastern Europe	Y	–	N	n.a.	Y
*Ailanthus altissima* (Mill.) Swingle	tree of heaven	Asia (Northern and Central China)	Y	Y	Y	n.a.	Y
*Betula papyrifera* Marshall	canoe birch	Northern North America	N	–	–	N	N
*Catalpa bignonioides* Walter	southern catalpa	Southeastern North America	Y	–	N	n.a.	Y
*Celtis occidentalis* L.	American hackberry	Eastern North America	N	–	N	N	N
*Davidia involucrata* Baill.	dove tree	Asia (Central and South Eastern China)	N	–	–	N	N
*Diospyros virginiana* L.	American persimmon	Eastern and Southeastern North America	Y	–	N	n.a.	Y
*Gleditsia triacanthos* L.	honey locust	Central and Eastern North America	Y	–	Y	n.a.	Y
*Gymnocladus dioicus* (L.) K.Koch	Kentucky coffee tree	Eastern North America	Y	–	N	n.a.	Y
*Koelreuteria paniculata* Laxm.	golden rain tree	Asia (China, Manchuria, Korea)	Y	–	N	n.a.	Y
*Liquidambar styraciflua* L.	sweetgum	Southeastern North America	N	–	N	N	N
*Liriodendron tulipifera* L.	tulip tree	Eastern North America	N	–	N	N	N
*Magnolia kobus* DC.	kobus magnolia	Asia (Japan, Korea)	N	–	–	N	N
*Parrotia persica* (DC.) C. A. Mey.	Persian ironwood	Temperate Asia (Iran)	N	–	–	N	N
*Paulownia tomentosa* (Thunb.) Steud.	princess tree	Asia (China, Korea)	N	Y	Y	n.a.	Y
*Phellodendron amurense* Rupr.	Amur cork tree	Asia (China, Korea, Japan)	N	–	N	N	N
*Populus simonii* Carrière	Simon poplar	Asia (China)	N	–	N	N	N
*Prunus serrulata* Lindl.	Japanese cherry	Temperate East Asia (China, Japan, Korea, Vietnam)	Y	–	N	n.a.	Y
*Pterocarya fraxinifolia* (Poir.) Spach	Caucasian wingnut	Temperate and Western Asia (North Caucasus, Transcaucasus, Iran, Turkey)	N	–	N	N	N
*Quercus rubra* L.	Northern red oak	Eastern and Central North America	Y	–	N	n.a.	Y
*Rhus typhina* L.	staghorn sumac	Eastern North America	Y	–	–	n.a.	Y
*Robinia pseudoacacia* L.	black locust	Eastern North America	Y	Y	Y	n.a.	Y
*Styphnolobium japonicum* (L.) Schott	Chinese scholar tree	Asia (China, Korea)	N	–	N	N	N
Gymnosperms							
*Cedrus libani* A. Rich.	cedar of Lebanon	Eastern Mediterranean (Turkey, Syria, Lebanon)	N	–	N	N	N
*Chamaecyparis lawsoniana* (A. Murray bis) Parl.	Lawson cypress	North America (Oregon, California)	N	–	N	N	N
*Ginkgo biloba* L.	ginkgo	Eastern Asia	N	–	N	N	N
*Picea pungens* Engelm.	blue spruce	Western North America	N	–	N	N	N
*Pinus strobus* Thunb.	Eastern white pine	Eastern North America	Y	–	N	n.a.	Y
*Pinus wallichiana* A.B. Jacks.	Bhutan pine	Temperate and Tropical Asia (Himalaya, Tibet)	N	–	N	N	N
*Pseudotsuga menziesii* (Mirb.) Franco	Douglas fir	Western North America	Y	–	N	n.a.	Y

**Table 2:** Risk outcomes for the non-native urban trees screened with the TPS-ISK for Continental Europe. For each taxon, the following information is provided: *a priori* categorization of invasiveness (N = non-invasive; Y = invasive; see Table 1), Basic Risk Assessment (BRA) and BRA + Climate Change Assessment (BRA + CCA) scores with corresponding risk ranks based on a calibrated threshold of 41 (L = Low; M = Medium; H = High; VH = Very high, based on an ad hoc threshold ≥ 50; see text for details), classification (Class: FP = false positive; TN = true negative; TP = true positive; ‘–’ = not applicable as medium-risk; see text for details), CCA as difference between BRA + CCA and BRA scores, and confidence factor (CF). Risk outcomes for the BRA scores (within interval): L [−20, 1[; M, [1, 41[; H ]41, 50[; and VH [50, 72]. Risk outcomes for the BRA + CCA scores: L, [−32, 1[; M [1, 41[; H ]41, 50[; and VH [50, 82]. Note the reverse bracket notation indicating an open interval.

		BRA			BRA + CCA				CF		
Taxon Name	*A priori*	Score	Rank	Class	Score	Rank	Class	CCA	Total	BRA	CCA
Angiosperms											
*Acer negundo*	Y	44.0	H	TP	44.0	H	TP	0.0	0.68	0.68	0.71
*Acer palmatum*	N	29.0	M	–	25.0	M	–	−4.0	0.56	0.59	0.29
*Acer saccharinum*	N	29.0	M	–	29.0	M	–	0.0	0.68	0.73	0.25
*Acer tataricum* ssp. *ginnala*	N	47.5	H	FP	59.5	H	FP	12.0	0.58	0.60	0.46
*Aesculus hippocastanum*	Y	26.0	M	–	26.0	M	–	0.0	0.68	0.71	0.42
*Ailanthus altissima*	Y	50.0	H	TP	62.0	H	TP	12.0	0.85	0.85	0.83
*Betula papyrifera*	N	15.0	M	–	15.0	M	–	0.0	0.64	0.65	0.50
*Catalpa bignonioides*	Y	18.0	M	–	18.0	M	–	0.0	0.66	0.70	0.38
*Celtis occidentalis*	N	29.0	M	–	29.0	M	–	0.0	0.62	0.63	0.50
*Davidia involucrata*	N	3.0	M	–	3.0	M	–	0.0	0.59	0.61	0.46
*Diospyros virginiana*	Y	58.0	H	TP	70.0	H	TP	12.0	0.78	0.78	0.83
*Gleditsia triacanthos*	Y	29.0	M	–	29.0	M	–	0.0	0.69	0.73	0.38
*Gymnocladus dioicus*	Y	12.0	M	–	12.0	M	–	0.0	0.57	0.58	0.50
*Koelreuteria paniculata*	Y	47.0	H	TP	55.0	H	TP	8.0	0.71	0.74	0.50
*Liquidambar styraciflua*	N	26.0	M	–	28.0	M	–	2.0	0.49	0.52	0.25
*Liriodendron tulipifera*	N	21.0	M	–	33.0	M	–	12.0	0.42	0.43	0.33
*Magnolia kobus*	N	38.5	M	–	50.5	H	FP	12.0	0.55	0.57	0.38
*Parrotia persica*	N	4.0	M	–	4.0	M	–	0.0	0.46	0.47	0.38
*Paulownia tomentosa*	Y	16.0	M	–	16.0	M	–	0.0	0.57	0.61	0.25
*Phellodendron amurense*	N	46.0	H	FP	58.0	H	FP	12.0	0.47	0.50	0.25
*Populus simonii*	N	40.0	M	–	40.0	M	–	0.0	0.48	0.51	0.25
*Prunus serrulata*	Y	−6.0	L	FN	−10.0	L	FN	−4.0	0.65	0.63	0.75
*Pterocarya fraxinifolia*	N	27.5	M	–	33.5	M	–	6.0	0.73	0.75	0.58
*Quercus rubra*	Y	60.0	H	TP	72.0	H	TP	12.0	0.65	0.65	0.63
*Rhus typhina*	Y	33.0	M	–	37.0	M	–	4.0	0.70	0.74	0.38
*Robinia pseudoacacia*	Y	42.0	H	TP	50.0	H	TP	8.0	0.85	0.85	0.88
*Styphnolobium japonicum*	N	15.0	M	–	13.0	M	–	−2.0	0.64	0.66	0.46
Gymnosperms											
*Cedrus libani*	N	1.0	M	–	−5.0	L	TN	−6.0	0.65	0.64	0.71
*Chamaecyparis lawsoniana*	N	23.0	M	–	29.0	M	–	6.0	0.66	0.69	0.38
*Ginkgo biloba*	N	18.0	M	–	18.0	M	–	0.0	0.53	0.54	0.50
*Picea pungens*	N	19.0	M	–	13.0	M	–	−6.0	0.43	0.45	0.25
*Pinus strobus*	Y	45.0	H	TP	49.0	H	TP	4.0	0.75	0.77	0.54
*Pinus wallichiana*	N	13.0	M	–	1.0	M	–	−12.0	0.39	0.41	0.25
*Pseudotsuga menziesii*	Y	46.0	H	TP	58.0	H	TP	12.0	0.59	0.61	0.38

## Data Availability

The original data of this study are contained within the article and Supplementary Materials.

## Supplementary Materials

The following supporting information can be downloaded at: https://www.mdpi.com/article/10.3390/plants15152313/s1, Material S1: The TPS-ISK v2 risk screening reports for the 28 non-native tree species assessed in this study.
