# Supplementary material for: Emerging Invasion Risks of Non-Native Urban Trees in Continental Europe Under a Changing Climate
Source: Plants (Basel). 2026 Jul 28;15(15):2313. doi: 10.3390/plants15152313 (PMC13467876; doi:10.3390/plants15152313)
Supplement: Supplementary file 1 [file plants-15-02313-s001.zip › plants-4432081-supplementary Material S1.pdf]

TPS-ISK v2

**Taxon and Assessor details**	
Category	Angiosperms
Taxon name	*Acer negundo*
Common name	Box Elder
Assessor	Seokmin Kim
**Risk screening context**	
Reason and socio-economic benefits	Grown in urban settings for shade and ornamental use. Limited use for forestry
Risk assessment area	Continental Europe
Taxonomy	Family: Sapindaceae, Genus: Acer
Native range	North America
Introduced range	Eurasia and Indian subcontinent, South Africa, Australia and New Zealand, South America
URL	https://edis.ifas.ufl.edu/publication/ST020

			Response	Justification (references or other information)	Confidence
**A. Biogeography/Historical**					
1. Domestication/Cultivation					
1	1.01	Has the taxon been the subject of domestication?	No	No evidence that plant's life cycle, behavior, or appearance has been significantly alterared as a result of being under artificial	Medium
2	1.02	Is the taxon harvested in the wild and likely to be sold or used in its live form?	Yes	1. 2. Noted to be a widely cultivated ornamental species. Many cultivars exist. 1. https://keyserver.lucidcentral.org/weeds/data/media/Html/acer_negundo.htm 2. https://www.srs.fs.usda.gov/pubs/misc/ag_654	High
3	1.03	Does the taxon have invasive races, varieties, sub-taxa or congeners?	Yes	1. Acer platanoides considered invasive in North America 2. Acer pseudoplatanus considered invasive 1. https://www.brandywine.org/conservancy/blog/invasive-species-spotlight-norway-maple 2. https://landscapeplants.oregonstate.edu/plants/acer-	High
2. Climate, distribution and introduction risk					
4	2.01	How similar are the climatic conditions between the risk assessment area and the taxon's native range?	High	1. Plant hardiness of 3a to 8b. 2. Europe's plant hardiness is from 3a to 10b. 3. GBIF points corroborate these zones. 4. Noted that climatic risk of invasion in Europe is "especially high" for the taxon 1. https://edis.ifas.ufl.edu/publication/ST020 2. https://davisla.wordpress.com/plant-zones/europe-plant-hardiness-zone-map/ 3. https://www	High
5	2.02	What is the quality of the climate matching data?	Medium	Did not conduct a full climate matching process as it is difficult to pinpoint where the native range overlays with gbif observations.	Medium
6	2.03	Is the taxon known to be present outside of cultivation in the risk assessment area?	Yes	1. Plant observations found in Europe. 2. Noted to have escaped cultivation in Belgium. 1. https://www.gbif.org/species/3189866 2. https://alienplantsbelgium.myspecies.info/content/acer-	Very high
7	2.04	How many potential vectors could the taxon use to enter the risk assessment area?	>1	1. 2. Plant cultivated and planted in Europe intentionally. 2. Dispersal by wind can spread seeds over several kilometers. 3. Biotic dispersal possible, but not well recorded 1. https://www.srs.fs.usda.gov/pubs/misc/ag_654/volume_2/acer/negundo.htm 2. https://www.cabidigitallibrary.org/doi/10.1079/	Medium
8	2.05	Is the taxon currently found in close proximity to, and likely to enter, the risk assessment area in the near future (e.g. unintentional or intentional introductions)?	Not applicable	1. 2. Plant already found in the risk assessment area. 1. https://www.gbif.org/species/3189866 2.https://alienplantsbelgium.myspecies.info/content/acer-negundo	Very high
3. Invasive elsewhere					
9	3.01	Has the taxon become naturalised outside its native range?	Yes	1. Noted as having naturalized in Belgium and 2. other parts of Europe. 1. https://alienplantsbelgium.myspecies.info/content/acer-negundo 2. https://plants.usda.gov/DocumentLibrary/plantguide/pdf/pg_acne2.pdf	High
10	3.02	In the taxon's introduced range, are there any known adverse impacts to wild or commercial species?	Yes	1. 2. Noted to be invading protected areas in Poland. 3. Invading native riparian forests in the Rhone river cfloodplain communities 4. Outcompetes native species in Europe. 1. https://bsppjournals.onlinelibrary.wiley.com/doi/full/10.1111/ppa.13785 2. https://www.cabidigitallibrary.org/doi/10.1079/cabicompendium.2862 3. https://www.researchgate.net/publication/235983001_Ecological_resistance_to_Acer_negundo_invasion_in_a_European_riparian_forest_Relative_importance_of_environmental_and_biotic_drivers/link/59f05941a6fdcc1dc7b51bf7/download?_tp=eyJjb250ZXh0Ijp7ImZpcnN0UGFnZSI6InB1YmxpY2F0aW9uIiwicGFnZSI6InB1YmxpY2F0aW9uIn19 4. https://link.springer.com/content/pdf/10.1186/1472-6785-11-28.pdf	Very high
11	3.03	In the taxon's introduced range, are there any known adverse impacts to agriculture or forestry?	No	1. Noted to negatively affect forestry production. However, no examples found within the risk assessment area. Given absence of reported adverse impacts, answered NO with LOW confidence. 1. https://www.cabidigitallibrary.org/doi/10.1079/cabicompendium.2862	Low
12	3.04	In the taxon's introduced range, are there any known adverse impacts to ecosystem services?	Yes	1. Noted to limit recreation activity in Warsaw by outcompeting popular native trees in trails 2. Noted to affect riparian bank stability 3. However, also noted that plant may form an important component of riparian vegetation and increase siltation. Given absence of direct evidence on this, answered YES with MODERATE confidence. 1. https://core.ac.uk/download/pdf/237012459.pdf 2. https://muse.jhu.edu/article/634542 3. https://www.cabidigitallibrary.org/doi/10.1079/cabicompendium.2862	Medium

13	3.05	In the taxon's introduced range, are there any known adverse socio-economic impacts?	Yes	1. Noted to be able to damage railways. 2. Found near railways. Noted that it may cause damages. No direct evidence found, however. 1. https://bsppjournals.onlinelibrary.wiley.com/doi/full/10.1111/ppa.13785 2. file:///Users/seokminkim/Downloads/Invasive_flora_within_urban	Low
**B. Biology/Ecology**					
4. Undesirable (or persistence) traits					
14	4.01	Is the taxon likely to be poisonous or pose other risks to human health?	Yes	1. 2. Can cause respiratory allergic reactions 1. https://ipsmonitor.de/en/steckbriefe/acer-negundo/ 2. https://bsppjournals.onlinelibrary.wiley.com/doi/full/10.1111/ppa.13785 2. https://enticare.com/2024/05/31/exploring-box-elder-	Medium
15	4.02	Is the taxon likely to suppress the growth of one or more native species?	Yes	1. 2. Noted to be invading protected areas in Poland. 3. Invading native riparian forests in the Rhone river cfloodplain communities 4. Outcompetes native species in Europe. 1. https://bsppjournals.onlinelibrary.wiley.com/doi/full/10.1111/ppa.13785 2. https://www.cabidigitallibrary.org/doi/10.1079/cabicompendium.2862 3. https://www.researchgate.net/publication/235983001_Ecological_resistance_to_Acer_negundo_invasion_in_a_European_riparian_forest_Relative_importance_of_environmental_and_biotic_drivers/link/59f05941a6fdcc1dc7b51bf7/download?_tp=eyJjb250ZXh0Ijp7ImZpcnN0UGFnZSI6InB1YmxpY2F0aW9uIiwicGFnZSI6InB1YmxpY2F0aW9uIn19 4. https://link.springer.com/content/pdf/10.1186/1472-6785-11-28.pdf	Very high
16	4.03	Are there any threatened or protected native species that the taxon would parasitise in the risk assessment area?	No	Plant not known to be parasitic	High
17	4.04	Is the taxon adaptable in terms of climatic and other environmental conditions, thus enhancing its potential persistence if it has invaded or is likely to invade the risk assessment area?	Yes	1. Plant found in a variety of climatic areas. 2. Noted to be highly adaptable to different environments 1. https://www.gbif.org/species/3189866 2. https://www.cabidigitallibrary.org/doi/10.1079/cabicompendium.2862	High
18	4.05	Is the taxon likely to disrupt terrestrial ecosystem function if it has invaded or is likely to invade the risk assessment area?	Yes	1. Noted to affect riparian bank stability 2. However, also noted that plant may form an important component of riparian vegetation and increase siltation. Given absence of direct evidence on this, answered NO with MODERATE confidence. 1. https://muse.jhu.edu/article/634542 2. https://www.cabidigitallibrary.org/doi/10.1079/cabicompendium.2862	Low
19	4.06	Is the taxon likely to exert adverse impacts on ecosystem services in the risk assessment area?	Yes	1. Noted to limit recreation activity in Warsaw by outcompeting popular native trees in trails 2. Noted to affect riparian bank stability 3. However, also noted that plant may form an important component of riparian vegetation and increase siltation. Given absence of direct evidence on this, answered YES with MODERATE confidence. 1. https://muse.jhu.edu/article/634542 2. https://www.cabidigitallibrary.org/doi/10.1079/cabicompendium.2862	Medium
20	4.07	Is the taxon likely to host or function as a vector for recognised pests and pathogens that are present in the risk assessment area?	Yes	1. 2. Verticillium albo-atrum, which causese verticilium wilt, is a known host of taxon. Fungus considered pest in Europe. 3. Taxon host to the Asian long-horned beetle, which is also found in Europe. 1. https://www.cabidigitallibrary.org/doi/full/10.1079/cabicompendium.56268 2.	High
21	4.08	Is the taxon likely to host or function as a vector for recognised pests and pathogens that are absent in the risk assessment area?	No	1. Taxon is major host of Prociphilus mexicanus. 2. 3. 4. This has not been reported in Europe yet. Given difficulties of assessing aphid presence, answered NO with MODERATE confidence. 1. https://www.cabidigitallibrary.org/doi/10.1079/cabicompendium.2862 2. https://influentialpoints.com/Gallery/Prociphilus_aphids.htm 3. https://www.cabidigitallibrary.org/doi/10.1079/cabicompendium.4	Medium
22	4.09	Is the taxon likely to be released from cultivation?	Yes	1. 2. Taxon has shown to escape cultivation. 1. https://alienplantsbelgium.myspecies.info/content/acer-negundo 2. https://burkeherbarium.org/imagecollection/taxon.php?Taxon=Acer%20negundo#:~:text=Acer%20negundo%20%2D%20Burke%20Herbarium%20Image%20Collection&text=Distribution%3A%20Occ	Very high
23	4.10	Is the taxon versatile in habitat use?	Yes	1. Noted to be highly adaptable to different environments. 2. Observed in very different habitats and regions 1. https://www.cabidigitallibrary.org/doi/10.1079/cabicompendium.2862 2. https://www.gbif.org/species/3189866	Very high
24	4.11	Is it likely that the taxon's mode of existence or behaviours will reduce habitat quality for native species?	Yes	1. 2. Seeds noted to be toxic to horses. Unclear if also toxic to other grazing animals. 1. https://www.canr.msu.edu/news/boxelder_trees_are_toxic_to_horses 2. https://horsedvm.com/poisonous/boxelder	High
25	4.12	Is the taxon likely to maintain a viable population even when present in low densities (or persist in adverse conditions by way of a dormant form)?	No	Not much information. 1. However, plant is dioceious, therefore may need to be in sufficient densities to reproduce effectively. 2. Esepcially as plant is not known to be self fertile. Asnwered NO with LOW confidence. 1. https://www.fs.usda.gov/database/feis/plants/tree/aceneg/all.html 2. https://pfaf.org/user/Plant.aspx?LatinName=Acer+negundo	Low
5. Resource exploitation					

26	5.01	Is the taxon likely to put threatened or protected native species under competitive pressure in the risk assessment area?	Yes	1. Noted as one of the five major invasive tree species in the EU with threats to protected areas. 2. 3. Noted to be invading protected areas in Poland. 1. https://www.sciencedirect.com/science/article/pii/S1617138117300444 2. https://bsppjournals.onlinelibrary.wiley.com/doi/full/10.1111/ppa.13785 3. https://www.cabidigitallibrary.org/doi/10.1079/cabicompendium.2862	High
27	5.02	Is the taxon likely to sequester resources to the detriment of native species in the risk assessment area?	Yes	RIP value not found. However, 1. Noted to outperform native species in resource rich areas. 1. https://www.ncbi.nlm.nih.gov/pmc/articles/PMC3275484/	Medium
6. Reproduction					
28	6.01	Is the taxon likely to exhibit changes in reproductive strategy in response to environmental conditions?	No	No evidence that plant changes reproductive strategy in response to environmental conditions.	Medium
29	6.02	Is the taxon likely to produce viable propagules in the risk assessment area?	Yes	1. 2. Plant is spreading and naturalized in the risk assessment area. 1. https://alienplantsbelgium.myspecies.info/content/acer-negundo 2. https://plants.usda.gov/DocumentLibrary/plantguide/pdf/pg_acne2	Very high
30	6.03	Is the taxon likely to hybridise with native species under natural conditions?	No	No evidemce that taxon is likely to hybridise with native species. Lack of direct reporting. Answered NO with MODERATE uncertainty	Medium
31	6.04	Is the taxon likely to be hermaphroditic or to exhibit asexual reproduction?	No	1. Plant is dioceious, and 2. not known to be self fertile. 1. https://www.fs.usda.gov/database/feis/plants/tree/aceneg/all.html 2. https://pfaf.org/user/Plant.aspx?LatinName=Acer+negundo	Very high
32	6.05	Is the taxon dependent on the presence of another species (or specific habitat features) to complete its life cycle?	No	No evidence taxon is dependent on other species. 1. Primarily wind pollinated. 1. https://www.cabidigitallibrary.org/doi/10.1079/cabicompendium.2862	High
33	6.06	Is the taxon likely to produce a large number of propagules?	Yes	1. 2. Noted as being a prolific seed producer 1. https://plants.usda.gov/DocumentLibrary/plantguide/pdf/pg_acne2.pdf 2. https://dendro.cnre.vt.edu/dendrology/USDAFSSilvics/3.pdf	High
34	6.07	How many time units (days, months, years) does the taxon require to reach the age at first reproduction?	8	1. Noted to start producing seeds at 8 years old. 1. https://plants.usda.gov/DocumentLibrary/plantguide/pdf/pg_acne2.pdf	Medium
7. Dispersal mechanisms					
35	7.01	How many potential vectors or pathways could the taxon use to disperse within the risk assessment area (with suitable habitats nearby)?	>1	1. 2. Plant cultivated and planted in Europe intentionally. 2. Dispersal by wind can spread seeds over several kilometers. 3. Biotic dispersal possible, but not well recorded 1. https://www.srs.fs.usda.gov/pubs/misc/ag_654/volume_2/acer/negundo.htm 2. https://www.cabidigitallibrary.org/doi/10.1079/cabicompendium.2862. Noted to be able to spread as contaminant of soil and be dispersed biotically, but little evidence found.	High
36	7.02	Will any of these vectors or pathways bring the taxon in close proximity to one or more protected areas?	Yes	1. 2. Already noted to be invading protected areas in Poland. 1. https://bsppjournals.onlinelibrary.wiley.com/doi/full/10.1111/ppa.13785 2. https://www.cabidigitallibrary.org/doi/10.1079/cabicompendium.2862	High
37	7.03	Does the taxon have a specialised means of attachment such that it enhances the likelihood of dispersal?	No	No evidence plant has specialised means of attachment	Very high
38	7.04	Is natural dispersal of the taxon likely to occur as seeds or spores in the risk assessment area?	Yes	1. 2. Noted as being a prolific seed producer 1. https://plants.usda.gov/DocumentLibrary/plantguide/pdf/pg_acne2.pdf 2. https://dendro.cnre.vt.edu/dendrology/USDAFSSilvics/3.pdf	Very high
39	7.05	Is natural dispersal of the taxon likely to occur as fragments or seedlings in the risk assessment area?	No	1. 2. While noted as being able to vegetatively propagate, this is only for damaged individuals. No evidence that plant fragments propagates taxon. 1. https://www.cabidigitallibrary.org/doi/10.1079/cabicompendium.2862 2. https://www.fs.usda.gov/database/feis/plants/tree/aceneg/all.html	Medium
40	7.06	Are any life stages of the taxon likely to migrate into the risk assessment area for reproduction?	Yes	1. Plant observations already found in Europe. 2. Known prolific seed producer. 1. https://www.gbif.org/species/3189866 2. https://plants.usda.gov/DocumentLibrary/plantguide/pdf/pg_acne2.pdf	Very high
41	7.07	Are propagules of the taxon likely to be dispersed in the risk assessment area by other species?	No	1. 2. While biotic dispersal by birds and squirrels is noted to be possible, could not find any direct evidence. 1. https://www.cabidigitallibrary.org/doi/10.1079/cabicompendium.2862 2. https://digitalcommons.unl.edu/cgi/viewcontent.cgi?params=/context/agronomyfacpub/article/1496/&path_info=Weaver_EM_1960_Flood_plain_vegetation.pdf	Medium
42	7.08	Is dispersal of the taxon along any of the vectors or pathways mentioned in the previous seven Questions (35–41: i.e. either unintentional or intentional) likely to be	Yes	Wind dispersal may provide rapid dispersal	Medium
43	7.09	Is dispersal of the taxon density dependent?	Yes	Not much information. 1. However, plant is dioceious, therefore may need to be in sufficient densities to reproduce effectively. 2. Esepcially as plant is not known to be self fertile. Asnwered YES with LOW confidence 1. https://www.fs.usda.gov/database/feis/plants/tree/aceneg/all.html 2. https://pfaf.org/user/Plant.aspx?LatinName=Acer+negundo	Low
8. Tolerance attributes					

44	8.01	Is the taxon able to withstand being in water for extended periods at some stage of its life cycle?	Yes	1. 2. Exists in ruderal floodplains, which submerge the plant at times. 1. https://explorer.natureserve.org/Taxon/ELEMENT_GLOBAL.2.686021/Acer_negundo_Ruderal_Floodplain_Forest 2. https://www.fs.usda.gov/database/feis/plants/tree/aceneg/all.html	Medium
45	8.02	Is the taxon tolerant of a wide range of soil or air quality conditions?	Yes	1. Noted to be able to tolerate a wide verity of soils. 2. Noted to be able to tolerate urban conditions, implying it tolerates varying air quality conditions. 1. https://www.fs.usda.gov/database/feis/plants/tree/aceneg/all.html#23 2. https://canr.udel.edu/udbg/?plant=acer-negundo	High
46	8.03	Can the taxon be controlled or eradicated in the wild with chemical, biological or other agents and means?	Yes	1. Girding shown to be effective 2. 3. 4. Herbicide and mechanical treatments shown to be effective 1. https://muse.jhu.edu/article/634542 2. https://goodoak.com/info/weeds/boxelder.pdf 3. https://www.bio-conferences.org/articles/bioconf/full_html/2020/08/bioconf_pd2020_00063/bioconf_pd2020_00063.html 4. https://www.bio-conferences.org/articles/bioconf/full_html/2020/08/bioconf_pd2020_00063/bioconf_pd2020_00063.html	Very high
47	8.04	Is the taxon likely to benefit from environmental or human disturbance?	Yes	1. 2. Noted to be able to tolerate cultivation, mutilation, and fires 1. https://www.cabidigitallibrary.org/doi/10.1079/cabicompendium.2862 2. https://www.fs.usda.gov/database/feis/plants/tree/aceneg/all.html	High
48	8.05	Is the taxon able to tolerate soil acidity, salinity or other parameter levels that are higher or lower than those found in its usual	Yes	1. 2. Noted to be able to tolerate acidic soils (pH ~ 5) 1. https://woodyplants.cals.cornell.edu/plant/361 2. https://www.jstor.org/stable/pdf/2484104.pdf	Very high
49	8.06	Are there effective natural enemies of the taxon present in the risk assessment area?	No	Not much information regarding effective biotic control agents for this taxon. Instead, chemical and mechanical means are better studied. Given absence of information, answered NO with LOW	Low
**C. Climate change**					
9. Climate change					
50	9.01	Under the predicted future climatic conditions, are the risks of entry into the risk assessment area posed by the taxon likely to increase, decrease or not change?	No change	1. Plant already well established in the risk assessment area. 1. https://www.gbif.org/species/3189866	High
51	9.02	Under the predicted future climatic conditions, are the risks of establishment within the risk assessment area posed by the taxon likely to increase, decrease or not	No change	1. Plant already well established in the risk assessment area. 1. https://www.gbif.org/species/3189866	High
52	9.03	Under the predicted future climatic conditions, are the risks of dispersal within the risk assessment area posed by the taxon likely to increase, decrease or not change?	No change	Plant is mainly propagated by wind. Potential that plant will outcompete native species if they do not tolerate climate change, but no information on that found	Medium
53	9.04	Under the predicted future climatic conditions, what is the likely magnitude of future potential impacts by the taxon on biodiversity or ecosystem integrity and	No change	1. Plant already well established in the risk assessment area. 1. https://www.gbif.org/species/3189866	High
54	9.05	Under the predicted future climatic conditions, what is the likely magnitude of future potential impacts by the taxon on ecosystem structure or function?	No change	1. Plant already well established in the risk assessment area. 1. https://www.gbif.org/species/3189866	High
55	9.06	Under the predicted future climatic conditions, what is the likely magnitude of future potential impacts by the taxon on ecosystem services and related socio-	No change	1. Plant already well established in the risk assessment area. 1. https://www.gbif.org/species/3189866	High

**Statistics**	
**Scores**	
**BRA**	**44.0**
**BRA Outcome**	**High**
**BRA+CCA**	**44.0**
**BRA+CCA Outcome**	**High**
**Score partition**	
**A. Biogeography/Historical**	**18.0**
1. Domestication/Cultivation	*2.0*
2. Climate, distribution and introduction risk	*2.0*
3. Invasive elsewhere	*14.0*
**B. Biology/Ecology**	**26.0**
4. Undesirable (or persistence) traits	*9.0*
5. Resource exploitation	*7.0*
6. Reproduction	*0.0*
7. Dispersal mechanisms	*3.0*
8. Tolerance attributes	*7.0*
**C. Climate change**	**0.0**
9. Climate change	*0.0*
**Answered Questions**	
Total	***55***
**A. Biogeography/Historical**	**13**
1. Domestication/Cultivation	*3*
2. Climate, distribution and introduction risk	*5*
3. Invasive elsewhere	*5*
**B. Biology/Ecology**	**36**
4. Undesirable (or persistence) traits	*12*
5. Resource exploitation	*2*
6. Reproduction	*7*

7. Dispersal mechanisms	*9*
8. Tolerance attributes	*6*
**C. Climate change**	**6**
9. Climate change	*6*
**Sectors affected**	
**Commercial**	**13**
**Environmental**	**13**
**Species or population nuisance traits**	**23**

**Thresholds**	
**BRA**	**41**
**BRA+CCA**	**41**
**Confidence**	
**BRA+CCA**	**0.68**
**BRA**	**0.68**
**CCA**	**0.71**

**Date and Time**
**15/08/2024 09:49:09**

TPS-ISK v2

**Taxon and Assessor details**	
Category	Angiosperms
Taxon name	*Acer palmatum*
Common name	Japanese Maple
Assessor	Dario Hruševar
**Risk screening context**	
Reason and socio-economic benefits	horticulture
Risk assessment area	Continental Europe
Taxonomy	Sapindaceae; VERNACULAR NAME INVALID (Green Taxonomy)
Native range	Japan, China; Mongolia; eastern Asia, temperate Asia
Introduced range	Europe, Americas, Australia
URL	

			**Response**	**Justification (references or other information)**	**Confidence**
**A. Biogeography/Historical**					
1. Domestication/Cultivation					
1	1.01	Has the taxon been the subject of domestication?	Yes	https://www.gbif.org/species/141266283; https://bie.ala.org.au/species/https://id.biodiversity.org.au/node/apni/2909487	Very high
2	1.02	Is the taxon harvested in the wild and likely to be sold or used in its live form?	Yes	numerous web-pages, e.g. https://www.acersonline.co.uk/; https://bie.ala.org.au/species/https://id.biodiversity.org.au/node/apni/2909487	Very high
3	1.03	Does the taxon have invasive races, varieties, sub-taxa or congeners?	No	e.g. https://bie.ala.org.au/species/https://id.biodiversity.org.au/node/apni/2909487	High
2. Climate, distribution and introduction risk					
4	2.01	How similar are the climatic conditions between the risk assessment area and the taxon's native range?	High	Peeel et al. 2007	High
5	2.02	What is the quality of the climate matching data?	High	Peel et al. 2007	High
6	2.03	Is the taxon known to be present outside of cultivation in the risk assessment area?	Yes	https://alienplantsbelgium.myspecies.info/content/acer-palmatum#	Very high
7	2.04	How many potential vectors could the taxon use to enter the risk assessment area?	>1	https://alienplantsbelgium.myspecies.info/content/acer-palmatum#; https://bie.ala.org.au/species/https://id.biodiversity.org.au/node/a	High
8	2.05	Is the taxon currently found in close proximity to, and likely to enter, the risk assessment area in the near future (e.g. unintentional or intentional introductions)?	Yes	https://alienplantsbelgium.myspecies.info/content/acer-palmatum#	High
3. Invasive elsewhere					
9	3.01	Has the taxon become naturalised outside its native range?	Yes	https://alienplantsbelgium.myspecies.info/content/acer-palmatum#; https://bie.ala.org.au/species/https://id.biodiversity.org.au/node/a	High
10	3.02	In the taxon's introduced range, are there any known adverse impacts to wild or commercial species?	No	professional judgement; no references found	Medium
11	3.03	In the taxon's introduced range, are there any known adverse impacts to agriculture or forestry?	No	professional judgement; no references found	Low
12	3.04	In the taxon's introduced range, are there any known adverse impacts to ecosystem	No	professional judgement; no references found	Low
13	3.05	In the taxon's introduced range, are there any known adverse socio-economic impacts?	Yes	https://www.acersonline.co.uk/; https://alienplantsbelgium.myspecies.info/content/acer-palmatum#; https://bie.ala.org.au/species/https://id.biodiversity.org.au/node/a	High
**B. Biology/Ecology**					
4. Undesirable (or persistence) traits					
14	4.01	Is the taxon likely to be poisonous or pose other risks to human health?	No	numerous web-pages - rare human health risk is only known related to same allergenic reactions (eg. https://plantaddicts.com/are-maple-trees-poisonous/)	High
15	4.02	Is the taxon likely to suppress the growth of one or more native species?	No	No data, professional judgement	Medium
16	4.03	Are there any threatened or protected native species that the taxon would parasitise in the risk assessment area?	No	No data, professional judgement	Medium
17	4.04	Is the taxon adaptable in terms of climatic and other environmental conditions, thus enhancing its potential persistence if it has invaded or is likely to invade the risk	Yes	Peel et al 207; professional judgement	Medium
18	4.05	Is the taxon likely to disrupt terrestrial ecosystem function if it has invaded or is likely to invade the risk assessment area?	No	No data, professional judgement	Medium
19	4.06	Is the taxon likely to exert adverse impacts on ecosystem services in the risk	No	No data, professional judgement	Low
20	4.07	Is the taxon likely to host or function as a vector for recognised pests and pathogens that are present in the risk assessment area?	Yes	https://vtechworks.lib.vt.edu/server/api/core/bitstreams/0aa98a4c-c9c2-4cfe-833c-4dd803ccc9ee/content; https://www.gardeningknowhow.com/ornamental/trees/japanese-maple/japanese-maple-problems.htm	Medium

21	4.08	Is the taxon likely to host or function as a vector for recognised pests and pathogens that are absent in the risk assessment area?	Yes	https://vtechworks.lib.vt.edu/server/api/core/bitstreams/0aa98a4c-c9c2-4cfe-833c-4dd803ccc9ee/content; https://www.gardeningknowhow.com/ornamental/trees/japanese-maple/japanese-maple-problems.htm; https://hgic.clemson.edu/factsheet/maple-diseases-insect-pests/	Medium
22	4.09	Is the taxon likely to be released from cultivation?	Yes	At the moment in the risk assesment area it is considered casual (eg. https://alienplantsbelgium.myspecies.info/content/acer-	Medium
23	4.10	Is the taxon versatile in habitat use?	Yes	professional judgement	Low
24	4.11	Is it likely that the taxon's mode of existence or behaviours will reduce habitat quality for native species?	Yes	https://plantaddicts.com/are-maple-trees-poisonous/	Medium
25	4.12	Is the taxon likely to maintain a viable population even when present in low densities (or persist in adverse conditions by way of a dormant form)?	Yes	In Australia (mentioned in https://alienplantsbelgium.myspecies.info/content/acer-palmatum#).	Medium
5. Resource exploitation					
26	5.01	Is the taxon likely to put threatened or protected native species under competitive pressure in the risk assessment area?	No	No data, professional judgement	Medium
27	5.02	Is the taxon likely to sequester resources to the detriment of native species in the risk assessment area?	No	professional judgement	Medium
6. Reproduction					
28	6.01	Is the taxon likely to exhibit changes in reproductive strategy in response to environmental conditions?	No	No data, professional judgement	Low
29	6.02	Is the taxon likely to produce viable propagules in the risk assessment area?	Yes	https://alienplantsbelgium.myspecies.info/content/acer-palmatum#	Medium
30	6.03	Is the taxon likely to hybridise with native species under natural conditions?	Yes	Forrest 2019: Jackson Sci J 1(1): 4-7.	Medium
31	6.04	Is the taxon likely to be hermaphroditic or to exhibit asexual reproduction?	Yes	https://vtechworks.lib.vt.edu/server/api/core/bitstreams/0aa98a4c-c9c2-4cfe-833c-4dd803ccc9ee/content	High
32	6.05	Is the taxon dependent on the presence of another species (or specific habitat features) to complete its life cycle?	No	No data, professional judgement	Very high
33	6.06	Is the taxon likely to produce a large number of propagules?	Yes	https://www.invasive.org/browse/subinfo.cfm?sub=3001	Very high
34	6.07	How many time units (days, months, years) does the taxon require to reach the age at first reproduction?	4	flowering stage typically begins when the tree is between 4 and 6 years old (https://greg.app/japanese-maple-lifecycle/)	Medium
7. Dispersal mechanisms					
35	7.01	How many potential vectors or pathways could the taxon use to disperse within the risk assessment area (with suitable habitats nearby)?	>1	humans, wind, animals (professional judgement)	Medium
36	7.02	Will any of these vectors or pathways bring the taxon in close proximity to one or more protected areas?	Yes	professional judgement. However, in the screening area it is considering casual (https://alienplantsbelgium.myspecies.info/content/acer-	Low
37	7.03	Does the taxon have a specialised means of attachment such that it enhances the likelihood of dispersal?	Yes	winged samaras (eg. https://bie.ala.org.au/species/https://id.biodiversity.org.au/node/apni/2909487)	Medium
38	7.04	Is natural dispersal of the taxon likely to occur as seeds or spores in the risk	Yes	Nave et al. 2021: Biomimetics (Basel), 6(2): 23, doi: 10.3390/biomimetics6020023	High
39	7.05	Is natural dispersal of the taxon likely to occur as fragments or seedlings in the risk assessment area?	No	no data, professional judgement	Low
40	7.06	Are any life stages of the taxon likely to migrate into the risk assessment area for reproduction?	Yes	Gao, J., Liao, PC., Huang, BH. et al. Historical biogeography of Acer L. (Sapindaceae): genetic evidence for Out-of-Asia hypothesis with multiple dispersals to North America and Europe. Sci Rep 10, 21178 (2020). https://doi.org/10.1038/s41598-020-	High
41	7.07	Are propagules of the taxon likely to be dispersed in the risk assessment area by other species?	No	No data, professional judgement	Low
42	7.08	Is dispersal of the taxon along any of the vectors or pathways mentioned in the previous seven Questions (35–41: i.e. either unintentional or intentional) likely to be	No	professional judgement	Low
43	7.09	Is dispersal of the taxon density dependent?	No	No data,professional judgement	Low
8. Tolerance attributes					
44	8.01	Is the taxon able to withstand being in water for extended periods at some stage of its life cycle?	No	No data	Medium
45	8.02	Is the taxon tolerant of a wide range of soil or air quality conditions?	Yes	https://vtechworks.lib.vt.edu/server/api/core/bitstreams/0aa98a4c-c9c2-4cfe-833c-4dd803ccc9ee/content	Very high
46	8.03	Can the taxon be controlled or eradicated in the wild with chemical, biological or other agents and means?	Yes	data on erradication of some other species of the genus Acer (eg. https://treetesting.com/Controlling_Invasive_Plants.pdf)	High
47	8.04	Is the taxon likely to benefit from environmental or human disturbance?	Yes	https://www.invasive.org/browse/subinfo.cfm?sub=3001	High
48	8.05	Is the taxon able to tolerate soil acidity, salinity or other parameter levels that are higher or lower than those found in its usual	Yes	https://vtechworks.lib.vt.edu/server/api/core/bitstreams/0aa98a4c-c9c2-4cfe-833c-4dd803ccc9ee/content	Very high
49	8.06	Are there effective natural enemies of the taxon present in the risk assessment area?	Yes	https://vtechworks.lib.vt.edu/server/api/core/bitstreams/0aa98a4c-c9c2-4cfe-833c-4dd803ccc9ee/content	High
**C. Climate change**					

9. Climate change					
50	9.01	Under the predicted future climatic conditions, are the risks of entry into the risk assessment area posed by the taxon likely to increase, decrease or not change?	Decrease	professional judgement (also influenced due to Gao, J., Liao, PC., Huang, BH. et al. Historical biogeography of Acer L. (Sapindaceae): genetic evidence for Out-of-Asia hypothesis with multiple dispersals to North America and Europe. Sci Rep 10, 21178 (2020). https://doi.org/10.1038/s41598-020-78145-0)	Medium
51	9.02	Under the predicted future climatic conditions, are the risks of establishment within the risk assessment area posed by the taxon likely to increase, decrease or not change?	No change	professional judgement (also influenced due to Gao, J., Liao, PC., Huang, BH. et al. Historical biogeography of Acer L. (Sapindaceae): genetic evidence for Out-of-Asia hypothesis with multiple dispersals to North America and Europe. Sci Rep 10, 21178 (2020). https://doi.org/10.1038/s41598-020-78145-0)	Low
52	9.03	Under the predicted future climatic conditions, are the risks of dispersal within the risk assessment area posed by the taxon likely to increase, decrease or not change?	Decrease	professional judgement (also influenced due to Gao, J., Liao, PC., Huang, BH. et al. Historical biogeography of Acer L. (Sapindaceae): genetic evidence for Out-of-Asia hypothesis with multiple dispersals to North America and Europe. Sci Rep 10, 21178 (2020). https://doi.org/10.1038/s41598-020-78145-0)	Low
53	9.04	Under the predicted future climatic conditions, what is the likely magnitude of future potential impacts by the taxon on biodiversity or ecosystem integrity and	No change	professional judgement	Low
54	9.05	Under the predicted future climatic conditions, what is the likely magnitude of future potential impacts by the taxon on ecosystem structure or function?	No change	professional judgement	Low
55	9.06	Under the predicted future climatic conditions, what is the likely magnitude of future potential impacts by the taxon on ecosystem services and related socio-	No change	professional judgement	Low

**Statistics**	
**Scores**	
**BRA**	**29.0**
**BRA Outcome**	**Medium**
**BRA+CCA**	**25.0**
**BRA+CCA Outcome**	**Medium**
**Score partition**	
**A. Biogeography/Historical**	**12.0**
1. Domestication/Cultivation	*2.0*
2. Climate, distribution and introduction risk	*4.0*
3. Invasive elsewhere	*6.0*
**B. Biology/Ecology**	**17.0**
4. Undesirable (or persistence) traits	*7.0*
5. Resource exploitation	*0.0*
6. Reproduction	*4.0*
7. Dispersal mechanisms	*3.0*
8. Tolerance attributes	*3.0*
**C. Climate change**	**-4.0**
9. Climate change	*-4.0*
**Answered Questions**	
Total	***55***
**A. Biogeography/Historical**	**13**
1. Domestication/Cultivation	*3*
2. Climate, distribution and introduction risk	*5*
3. Invasive elsewhere	*5*
**B. Biology/Ecology**	**36**
4. Undesirable (or persistence) traits	*12*
5. Resource exploitation	*2*
6. Reproduction	*7*
7. Dispersal mechanisms	*9*
8. Tolerance attributes	*6*
**C. Climate change**	**6**
9. Climate change	*6*
**Sectors affected**	
**Commercial**	**12**
**Environmental**	**2**
**Species or population nuisance traits**	**17**

**Thresholds**	
**BRA**	**41**
**BRA+CCA**	**41**
**Confidence**	
**BRA+CCA**	**0.56**
**BRA**	**0.59**
**CCA**	**0.29**

**Date and Time**
**20/07/2024 23:21:24**

TPS-ISK v2

**Taxon and Assessor details**	
Category	Angiosperms
Taxon name	*Acer saccharinum*
Common name	Silver Maple
Assessor	Dario Hruševar
**Risk screening context**	
Reason and socio-economic benefits	ethnobotanical data, food source for animals; pulp for paper; horticulture; biofuel?
Risk assessment area	Continental Europe
Taxonomy	fam. Sapindaceae
Native range	North Amerika (US, Canada)
Introduced range	all other continents, incl. some western parts of North America
URL	https://bplant.org/plant/92; https://en.wikipedia.org/wiki/Acer_saccharinum; Sullivan, Janet. 1994. Acer_

			Response	Justification (references or other information)	Confidence
**A. Biogeography/Historical**					
1. Domestication/Cultivation					
1	1.01	Has the taxon been the subject of domestication?	Yes	Sullivan, Janet. 1994. Acer saccharinum. In: Fire Effects Information System, [Online]. U.S. Department of Agriculture, Forest Service, Rocky Mountain Research Station, Fire Sciences Laboratory (Producer). Available: https://www.fs.usda.gov/database/feis/plants/tree/acesah/all.html [2024, July 27]; https://alienplantsbelgium.myspecies.info/content/acer-	Very high
2	1.02	Is the taxon harvested in the wild and likely to be sold or used in its live form?	Yes	Sullivan, Janet. 1994. Acer saccharinum. In: Fire Effects Information System, [Online]. U.S. Department of Agriculture, Forest Service, Rocky Mountain Research Station, Fire Sciences Laboratory (Producer). Available: https://www.fs.usda.gov/database/feis/plants/tree/acesah/all.html [2024, July 27]; https://alienplantsbelgium.myspecies.info/content/acer-	Very high
3	1.03	Does the taxon have invasive races, varieties, sub-taxa or congeners?	Yes	Acer negundo (e.g. Nikolić et al. 2014: Veselkin, D.V., Dubrovin, D.I. & Pustovalova, L.A. High canopy cover of invasive Acer negundo L. affects ground vegetation taxonomic richness. Sci Rep 11, 20758 (2021). https://doi.org/10.1038/s41598-021-00258-x	Very high
2. Climate, distribution and introduction risk					
4	2.01	How similar are the climatic conditions between the risk assessment area and the taxon's native range?	High	Peel et al. 2007	High
5	2.02	What is the quality of the climate matching data?	Medium	Peel et al. 2007	Medium
6	2.03	Is the taxon known to be present outside of cultivation in the risk assessment area?	Yes	https://alienplantsbelgium.myspecies.info/content/acer-saccharinum#	Very high
7	2.04	How many potential vectors could the taxon use to enter the risk assessment area?	>1	Professional judgement (it can be spread by humans, and than by wind, water...).	High
8	2.05	Is the taxon currently found in close proximity to, and likely to enter, the risk assessment area in the near future (e.g. unintentional or intentional introductions)?	Yes	https://alienplantsbelgium.myspecies.info/content/acer-saccharinum#	Very high
3. Invasive elsewhere					
9	3.01	Has the taxon become naturalised outside its native range?	Yes	https://alienplantsbelgium.myspecies.info/content/acer-saccharinum#	Very high
10	3.02	In the taxon's introduced range, are there any known adverse impacts to wild or commercial species?	No	no data found (professional judgement)	Medium
11	3.03	In the taxon's introduced range, are there any known adverse impacts to agriculture or forestry?	No	No data found (professional judgement)	Medium
12	3.04	In the taxon's introduced range, are there any known adverse impacts to ecosystem	Yes	https://alienplantsbelgium.myspecies.info/content/acer-saccharinum#; professional judgement	High
13	3.05	In the taxon's introduced range, are there any known adverse socio-economic impacts?	No	No data found (professional judgement)	Medium
**B. Biology/Ecology**					
4. Undesirable (or persistence) traits					
14	4.01	Is the taxon likely to be poisonous or pose other risks to human health?	No	No data found (professional judgement)	High
15	4.02	Is the taxon likely to suppress the growth of one or more native species?	No	No data found (professional judgement)	Medium
16	4.03	Are there any threatened or protected native species that the taxon would parasitise in the risk assessment area?	No	No data found (professional judgement)	High
17	4.04	Is the taxon adaptable in terms of climatic and other environmental conditions, thus enhancing its potential persistence if it has invaded or is likely to invade the risk	No	No data found (professional judgement)	Medium
18	4.05	Is the taxon likely to disrupt terrestrial ecosystem function if it has invaded or is likely to invade the risk assessment area?	No	No data found (professional judgement)	Medium

19	4.06	Is the taxon likely to exert adverse impacts on ecosystem services in the risk assessment area?	Yes	Information for eg. A. negundo (personal judgement; Sikorska, D.; Sikorski, P.; Archiciński, P.; Chormański, J.; Hopkins, R.J. You Can't See the Woods for the Trees: Invasive Acer negundo L. in Urban Riparian Forests Harms Biodiversity and Limits Recreation Activity. Sustainability 2019, 11, 5838. https://doi.org/10.3390/su11205838; Veselkin, D.V., Dubrovin, D.I. & Pustovalova, L.A. High canopy cover of invasive Acer negundo L. affects ground vegetation taxonomic richness. Sci Rep 11, 20758 (2021). https://doi.org/10.1038/s41598-021-00258-x)	Medium
20	4.07	Is the taxon likely to host or function as a vector for recognised pests and pathogens that are present in the risk assessment area?	No	No data found (professional judgement)	Medium
21	4.08	Is the taxon likely to host or function as a vector for recognised pests and pathogens that are absent in the risk assessment area?	No	No data found (professional judgement)	Medium
22	4.09	Is the taxon likely to be released from cultivation?	Yes	https://alienplantsbelgium.myspecies.info/content/acer-saccharinum#	High
23	4.10	Is the taxon versatile in habitat use?	No	https://bplant.org/plant/92	High
24	4.11	Is it likely that the taxon's mode of existence or behaviours will reduce habitat quality for native species?	Yes	https://poisonousplants.ansci.cornell.edu/php/plants.php?action=indiv&byname=common&keynum=98	Very high
25	4.12	Is the taxon likely to maintain a viable population even when present in low densities (or persist in adverse conditions by way of a dormant form)?	Yes	https://alienplantsbelgium.myspecies.info/content/acer-saccharinum#	High
5. Resource exploitation					
26	5.01	Is the taxon likely to put threatened or protected native species under competitive pressure in the risk assessment area?	No	No data found (professional judgement)	Medium
27	5.02	Is the taxon likely to sequester resources to the detriment of native species in the risk assessment area?	No	No data found (professional judgement)	Medium
6. Reproduction					
28	6.01	Is the taxon likely to exhibit changes in reproductive strategy in response to environmental conditions?	No	No data found (professional judgement)	Medium
29	6.02	Is the taxon likely to produce viable propagules in the risk assessment area?	Yes	https://alienplantsbelgium.myspecies.info/content/acer-saccharinum#; professional judgement	Very high
30	6.03	Is the taxon likely to hybridise with native species under natural conditions?	No	No data found (professional judgement)	Medium
31	6.04	Is the taxon likely to be hermaphroditic or to exhibit asexual reproduction?	Yes	https://www.missouribotanicalgarden.org/PlantFinder/PlantFinderDetails.aspx?taxonid=275381	Very high
32	6.05	Is the taxon dependent on the presence of another species (or specific habitat features) to complete its life cycle?	No	professional judgement; https://www.missouribotanicalgarden.org/PlantFinder/PlantFinderDetails.aspx?taxonid=275381 etc.	Very high
33	6.06	Is the taxon likely to produce a large number of propagules?	Yes	https://www.missouribotanicalgarden.org/PlantFinder/PlantFinderDetails.aspx?taxonid=275381; https://bplant.org/plant/92 etc.	Very high
34	6.07	How many time units (days, months, years) does the taxon require to reach the age at first reproduction?	>10	https://en.wikipedia.org/wiki/Acer_saccharum; https://bplant.org/plant/92	Very high
7. Dispersal mechanisms					
35	7.01	How many potential vectors or pathways could the taxon use to disperse within the risk assessment area (with suitable habitats nearby)?	>1	By wind (even water "support") and humans: https://en.wikipedia.org/wiki/Acer_saccharum; https://bplant.org/plant/92; https://dengarden.com/landscaping/Silver-Maple-The-Nuisance-Tree; https://alienplantsbelgium.myspecies.info/content/acer-	Very high
36	7.02	Will any of these vectors or pathways bring the taxon in close proximity to one or more protected areas?	Yes	https://alienplantsbelgium.myspecies.info/content/acer-saccharinum#; professional judgement	High
37	7.03	Does the taxon have a specialised means of attachment such that it enhances the likelihood of dispersal?	Yes	samara "wings" (eg. https://dengarden.com/landscaping/Silver-Maple-The-Nuisance-Tree; https://en.wikipedia.org/wiki/Acer_saccharum; https://bplant.org/plant/92 etc.)	Very high
38	7.04	Is natural dispersal of the taxon likely to occur as seeds or spores in the risk assessment area?	Yes	eg. https://alienplantsbelgium.myspecies.info/content/acer-saccharinum#; https://en.wikipedia.org/wiki/Acer_saccharum; https://bplant.org/plant/92 etc.	Very high
39	7.05	Is natural dispersal of the taxon likely to occur as fragments or seedlings in the risk assessment area?	Yes	https://bplant.org/plant/92 (but data are not for EU)	Very high
40	7.06	Are any life stages of the taxon likely to migrate into the risk assessment area for reproduction?	Yes	https://bplant.org/plant/92; https://alienplantsbelgium.myspecies.info/content/acer-saccharinum#	High
41	7.07	Are propagules of the taxon likely to be dispersed in the risk assessment area by other species?	No	No data found (professional judgement)	Medium
42	7.08	Is dispersal of the taxon along any of the vectors or pathways mentioned in the previous seven Questions (35–41: i.e. either unintentional or intentional) likely to be	No	No data found (professional judgement)	Medium
43	7.09	Is dispersal of the taxon density dependent?	No	No data found (professional judgement)	Medium
8. Tolerance attributes					
44	8.01	Is the taxon able to withstand being in water for extended periods at some stage of its life cycle?	Yes	https://bplant.org/plant/92; https://alienplantsbelgium.myspecies.info/content/acer-saccharinum#	Very high

45	8.02	Is the taxon tolerant of a wide range of soil or air quality conditions?	No	https://bplant.org/plant/92	Medium
46	8.03	Can the taxon be controlled or eradicated in the wild with chemical, biological or other agents and means?	Yes	Data for Acer negundo (eg. Merceron, Nastasia R., et al. "Killing it Softly: Girdling as an Efficient Eco-friendly Method to Locally Remove Invasive Acer negundo." Ecological Restoration, vol. 34 no. 4, 2016, p. 297-305. Project MUSE, https://muse.jhu.edu/article/634542.)	Very high
47	8.04	Is the taxon likely to benefit from environmental or human disturbance?	Yes	https://alienplantsbelgium.myspecies.info/content/acer-saccharinum#; professional judgement	Medium
48	8.05	Is the taxon able to tolerate soil acidity, salinity or other parameter levels that are higher or lower than those found in its usual	Yes	https://www.missouribotanicalgarden.org/PlantFinder/PlantFinderDetails.aspx?taxonid=275381; https://bplant.org/plant/92	High
49	8.06	Are there effective natural enemies of the taxon present in the risk assessment area?	No	No data found (professional judgement)	Low
**C. Climate change**					
9. Climate change					
50	9.01	Under the predicted future climatic conditions, are the risks of entry into the risk assessment area posed by the taxon likely to increase, decrease or not change?	No change	professional judgement	Low
51	9.02	Under the predicted future climatic conditions, are the risks of establishment within the risk assessment area posed by the taxon likely to increase, decrease or not	No change	professional judgement	Low
52	9.03	Under the predicted future climatic conditions, are the risks of dispersal within the risk assessment area posed by the taxon likely to increase, decrease or not change?	No change	professional judgement	Low
53	9.04	Under the predicted future climatic conditions, what is the likely magnitude of future potential impacts by the taxon on biodiversity or ecosystem integrity and	No change	professional judgement	Low
54	9.05	Under the predicted future climatic conditions, what is the likely magnitude of future potential impacts by the taxon on ecosystem structure or function?	No change	professional judgement	Low
55	9.06	Under the predicted future climatic conditions, what is the likely magnitude of future potential impacts by the taxon on ecosystem services and related socio-	No change	professional judgement	Low

**Statistics**	
**Scores**	
**BRA**	**29.0**
**BRA Outcome**	**Medium**
**BRA+CCA**	**29.0**
**BRA+CCA Outcome**	**Medium**
**Score partition**	
**A. Biogeography/Historical**	**14.0**
1. Domestication/Cultivation	*4.0*
2. Climate, distribution and introduction risk	*4.0*
3. Invasive elsewhere	*6.0*
**B. Biology/Ecology**	**15.0**
4. Undesirable (or persistence) traits	*3.0*
5. Resource exploitation	*0.0*
6. Reproduction	*2.0*
7. Dispersal mechanisms	*5.0*
8. Tolerance attributes	*5.0*
**C. Climate change**	**0.0**
9. Climate change	*0.0*
**Answered Questions**	
Total	***55***
**A. Biogeography/Historical**	**13**
1. Domestication/Cultivation	*3*
2. Climate, distribution and introduction risk	*5*
3. Invasive elsewhere	*5*
**B. Biology/Ecology**	**36**
4. Undesirable (or persistence) traits	*12*
5. Resource exploitation	*2*
6. Reproduction	*7*
7. Dispersal mechanisms	*9*
8. Tolerance attributes	*6*
**C. Climate change**	**6**
9. Climate change	*6*
**Sectors affected**	
**Commercial**	**8**
**Environmental**	**7**
**Species or population nuisance traits**	**20**

**Thresholds**	
**BRA**	**41**
**BRA+CCA**	**41**
**Confidence**	
**BRA+CCA**	**0.68**
**BRA**	**0.73**

CCA	0.25

Date and Time
08/08/2024 22:03:00

TPS-ISK v2

**Taxon and Assessor details**	
Category	Angiosperms
Taxon name	*Acer tataricum ssp. ginnala*
Common name	Amur Maple
Assessor	Mihaela Britvec
**Risk screening context**	
Reason and socio-economic benefits	
Risk assessment area	Continental Europe
Taxonomy	
Native range	
Introduced range	
URL	

			**Response**	**Justification (references or other information)**	**Confidence**
**A. Biogeography/Historical**					
1. Domestication/Cultivation					
1	1.01	Has the taxon been the subject of domestication?	Yes	Amur Maple is native to northeastern Asia from easternmost Mongolia east to Korea and Japan, and north to the Russian Far East in the Amur River valley. Amur Maple was introduced to North America in 1860 (Bailey 1949). Amur Maple is tree with high aesthetic value. Attractive habitus and exotic leaf and inflorescence morphology predetermine himfor wide utilisation in parks, urban greenery and private ornamental gardens (Ferus	Very high
2	1.02	Is the taxon harvested in the wild and likely to be sold or used in its live form?	Yes	The Amur Maple is used mainly for ornamental purposes, having bright red leaves in the fall, red seed pods in winter, fragrant flower clusters, and a spreading crown which makes it ideal for screenings and hedges (https://www.iucngisd.org/gisd/speciesname/Acer+ginnala).	Very high
3	1.03	Does the taxon have invasive races, varieties, sub-taxa or congeners?	Yes	For example: Acer negundo https://www.cabidigitallibrary.org/doi/10.1079/cabicompendium.2862)	Very high
2. Climate, distribution and introduction risk					
4	2.01	How similar are the climatic conditions between the risk assessment area and the taxon's native range?	Medium	professional judgement	Medium
5	2.02	What is the quality of the climate matching data?	Medium	professional judgement	Medium
6	2.03	Is the taxon known to be present outside of cultivation in the risk assessment area?	Yes	Amur Maple is known in Denmark (https://www.nobanis.org/national-species-	Very high
7	2.04	How many potential vectors could the taxon use to enter the risk assessment area?	>1	The seeds of Amur Maple are dispersed primarily by wind with the help of winged samaras (https://www.invasive.org/weedcd/pdfs/ipane/Acerginnala.pdf). Amur Maple can be dispersed by humans.	Very high
8	2.05	Is the taxon currently found in close proximity to, and likely to enter, the risk assessment area in the near future (e.g. unintentional or intentional introductions)?	Not applicable	Amur Maple is known in Denmark (https://www.nobanis.org/national-species-info/?taxaid=18270&countryID=DK) and in Latvia (doi: 10.2478/fsmu-2022-0001).	High
3. Invasive elsewhere					
9	3.01	Has the taxon become naturalised outside its native range?	Yes	Amur Maple is known in Denmark (https://www.nobanis.org/national-species-	Low
10	3.02	In the taxon's introduced range, are there any known adverse impacts to wild or commercial species?	Yes	Amur Maple has the potential to displace native vegetation, including native maple species, due to its tolerance of partial shade and its ability to shade out other species. The Amur maple also has the potential to outcompete native flora with its ability to establish in a wide range of habitats and environmental conditions.	Low
11	3.03	In the taxon's introduced range, are there any known adverse impacts to agriculture or forestry?	Yes	Reductions in cover caused by Amur Maple were consistent with its effect on light availability. Thus impacts of Amur Maple were consistently associated with low light availability, and indicate severe detriment to forest biodiversity (Schuster and Reich 2018).	Medium
12	3.04	In the taxon's introduced range, are there any known adverse impacts to ecosystem services?	Yes	Amur Maple may alter ecosystem structure and function, for example: the quality of habitat and forage for wildlife (Schuster and Reich 2018).	Medium
13	3.05	In the taxon's introduced range, are there any known adverse socio-economic impacts?	Yes	Acer tataricum ssp. ginnala does not to have deleterious attributes such as spines or health-inducing risks and as such probably has limited social impacts. However, in the USA the insect Boisea trivittata, after feeding on pistillate trees of Acer negundo, over-winters on or near buildings. In spring, when it starts disperse to breed, this largish insect (over 1 cm long), may enter houses in large numbers and stain walls and curtains with brown faecal material. When crushed they produce a foul odour and may bite if handled carelessly. This harmful social impact has yet to be reported from Europe and other regions where the species has been introduced (https://www.cabidigitallibrary.org/doi/10.1079/cabicompendium.2	Low
**B. Biology/Ecology**					
4. Undesirable (or persistence) traits					
14	4.01	Is the taxon likely to be poisonous or pose other risks to human health?	No	https://forestadaptation.org/sites/default/files/2024-01/Final_Boston_FullTreeSpeciesVulnerabilityAssesment.pdf	Medium
15	4.02	Is the taxon likely to suppress the growth of one or more native species?	Yes	It was described Amur Maple's tendency to form dense canopies and have suggested that this is a key component of the species' mechanism of invasion and impact (Schuster and Reich 2018).	Medium

16	4.03	Are there any threatened or protected native species that the taxon would parasitise in the risk assessment area?	No	no evidence	Low
17	4.04	Is the taxon adaptable in terms of climatic and other environmental conditions, thus enhancing its potential persistence if it has invaded or is likely to invade the risk	Yes	Amur Maple can grow in a wide range of habitats, from open to distrubed forests, early successional forests, streamsides, swamps, urban areas, roadsides, edges, and gardens (https://www.iucngisd.org/gisd/speciesname/Acer+ginnala).	Very high
18	4.05	Is the taxon likely to disrupt terrestrial ecosystem function if it has invaded or is likely to invade the risk assessment area?	Yes	Amur Maple may alter ecosystem function (Schuster and Reich 2018).	Medium
19	4.06	Is the taxon likely to exert adverse impacts on ecosystem services in the risk	Yes	Amur Maple may alter ecosystem structure and function (Schuster and Reich 2018).	Low
20	4.07	Is the taxon likely to host or function as a vector for recognised pests and pathogens that are present in the risk assessment area?	No	no evidence	Low
21	4.08	Is the taxon likely to host or function as a vector for recognised pests and pathogens that are absent in the risk assessment area?	No	Amur Maple is usually pest-free. Aphids infest maples, usually Norway Maple, and may be numerous at times. Perhaps the most common is cottony maple scale (Gilman and Watson 1993).	Low
22	4.09	Is the taxon likely to be released from cultivation?	Yes	Containment of Amur Maple will become more problematic with the continued commercial distribution of the species, which increases propagule pressure and opportunities for further adaptation (Schuster and Reich 2018).	Medium
23	4.10	Is the taxon versatile in habitat use?	Yes	Amur Maple can establish in a variety of soils, from sandy loam to heavy clays (https://www.iucngisd.org/gisd/speciesname/Acer+ginnala).	Very high
24	4.11	Is it likely that the taxon's mode of existence or behaviours will reduce habitat quality for native species?	Yes	Ecological impacts are some of the most poorly understood aspect of Amur Maple invasions. Amur Maple invasion suppress native woody species and shade out herbaceous species. Indeed, one of the most consistent impacts of Amur maple invasions are lowered native biodiversity. Reductions in native biodiversity have been fundamentally linked to altered ecosystem structure and function, including changes in ecosystem processes and the quality of habitat and forage for wildlife (Schuster and Reich 2018). Amur Maple can also release compounds that can inhibit the growth of nearby plants (Morris, 2004).	Low
25	4.12	Is the taxon likely to maintain a viable population even when present in low densities (or persist in adverse conditions by way of a dormant form)?	Yes	Amur Maple is a prolific seed producer and a mature individual can produce up to 5000 seeds annually (Schuster and Reich 2018).	High
5. Resource exploitation					
26	5.01	Is the taxon likely to put threatened or protected native species under competitive pressure in the risk assessment area?	Yes	Based on its low seed mass, rapid reproductive rate, and short maturation period, Amur Maple should be expected to be invasive (Schuster and Reich 2018).	Medium
27	5.02	Is the taxon likely to sequester resources to the detriment of native species in the risk assessment area?	Yes	Amur Maple created dense canopies that only allowed 2% canopy light penetration, strongly reducing cover of all plants except Amur Maple (Schuster and Reich 2018).	High
6. Reproduction					
28	6.01	Is the taxon likely to exhibit changes in reproductive strategy in response to environmental conditions?	Yes	The increasing occurrence of wild Amur Maple populations in North America suggest that the species has adapted to its new range, that abiotic conditions are becoming more favorable, or that resident native communities are becoming less resistant to invasion, thus concluding a lag period and starting a new period of expansion for Amur Maple. However, data on Amur Maple are scarce, and most is based on observations from land managers and local herbaria. This is consistent with the lack of attention the species has received from researchers (Schuster and Reich 2018).	Low
29	6.02	Is the taxon likely to produce viable propagules in the risk assessment area?	Yes	doi: 10.2478/fsmu-2022-0001	High
30	6.03	Is the taxon likely to hybridise with native species under natural conditions?	No	Not known to hybridize (https://dnr.wisconsin.gov/sites/default/files/topic/Invasives/LR_Acer_tataricum.pdf).	Low
31	6.04	Is the taxon likely to be hermaphroditic or to exhibit asexual reproduction?	Yes	Stem cuttings are commonly used as a means of asexual propagation of Amur Maple (doi: 10.24266/0738-2898-38.2.37).	High
32	6.05	Is the taxon dependent on the presence of another species (or specific habitat features) to complete its life cycle?	No	no evidence	Low
33	6.06	Is the taxon likely to produce a large number of propagules?	Yes	Amur Maple can produce up to 5000 seeds annually (Schuster and Reich 2018).	Very high
34	6.07	How many time units (days, months, years) does the taxon require to reach the age at first reproduction?	>10	MMMM..........................jOZO	High
7. Dispersal mechanisms					
35	7.01	How many potential vectors or pathways could the taxon use to disperse within the risk assessment area (with suitable habitats nearby)?	>1	dispersal by wind and dispersal by humans	High
36	7.02	Will any of these vectors or pathways bring the taxon in close proximity to one or more protected areas?	Yes	dispersal by wind	High
37	7.03	Does the taxon have a specialised means of attachment such that it enhances the likelihood of dispersal?	No	no evidence	High
38	7.04	Is natural dispersal of the taxon likely to occur as seeds or spores in the risk assessment area?	Yes	The seeds of Amur Maple are dispersed primarily by wind with the help of winged samaras (https://www.invasive.org/weedcd/pdfs/ipane/Acerginnala.pdf).	Very high

39	7.05	Is natural dispersal of the taxon likely to occur as fragments or seedlings in the risk assessment area?	Yes	Stem cuttings are commonly used as a means of asexual propagation of Amur Maple (doi: 10.24266/0738-2898-38.2.37).	Medium
40	7.06	Are any life stages of the taxon likely to migrate into the risk assessment area for reproduction?	Yes	The seeds of Amur Maple are dispersed by wind with the help of winged samaras (https://www.invasive.org/weedcd/pdfs/ipane/Acerginnala.pdf).	Very high
41	7.07	Are propagules of the taxon likely to be dispersed in the risk assessment area by other species?	No	no evidence	Low
42	7.08	Is dispersal of the taxon along any of the vectors or pathways mentioned in the previous seven Questions (35–41: i.e. either unintentional or intentional) likely to be	Yes	dispersal by wind	High
43	7.09	Is dispersal of the taxon density dependent?	Yes	More plants have more seeds.	Medium
8. Tolerance attributes					
44	8.01	Is the taxon able to withstand being in water for extended periods at some stage of its life cycle?	No	no evidence	Low
45	8.02	Is the taxon tolerant of a wide range of soil or air quality conditions?	Yes	Acer tataricum ssp. ginnala grows best in moist well-drained soil, but can tolerate a wide variety of soils, poor soil fertility, and are pH adaptable. This species displays excellent tolerance to dry and alkaline soils (USDA NRCS National Plant Data Center).	Very high
46	8.03	Can the taxon be controlled or eradicated in the wild with chemical, biological or other agents and means?	Yes	Multiple techniques have been suggested for removing and controlling Amur Maple invasions. Both mechanical removal of Amur Maple and chemical controls, including the application of glyphosate to a cut stem and basal bark application of triclopyr, have been suggested as viable management options. However, the efficacy of any of these options in removing existing invasions of Amur Maple has not been addressed in the literature (Schuster	Medium
47	8.04	Is the taxon likely to benefit from environmental or human disturbance?	Yes	Planted cultivars of Amur Maple have been reported as moderately drought tolerant (USDA NRCS National Plant Data Center).	Low
48	8.05	Is the taxon able to tolerate soil acidity, salinity or other parameter levels that are higher or lower than those found in its usual	Yes	For example, Amur Maple trees are pH adaptable (USDA NRCS National Plant Data Center).	Very high
49	8.06	Are there effective natural enemies of the taxon present in the risk assessment area?	No	no evidence	Low
**C. Climate change**					
9. Climate change					
50	9.01	Under the predicted future climatic conditions, are the risks of entry into the risk assessment area posed by the taxon likely to increase, decrease or not change?	Increase	professional judgement	Low
51	9.02	Under the predicted future climatic conditions, are the risks of establishment within the risk assessment area posed by the taxon likely to increase, decrease or not	Increase	professional judgement	Medium
52	9.03	Under the predicted future climatic conditions, are the risks of dispersal within the risk assessment area posed by the taxon likely to increase, decrease or not change?	Increase	professional judgement	Medium
53	9.04	Under the predicted future climatic conditions, what is the likely magnitude of future potential impacts by the taxon on biodiversity or ecosystem integrity and	Higher	professional judgement	Medium
54	9.05	Under the predicted future climatic conditions, what is the likely magnitude of future potential impacts by the taxon on ecosystem structure or function?	Higher	professional judgement	Medium
55	9.06	Under the predicted future climatic conditions, what is the likely magnitude of future potential impacts by the taxon on ecosystem services and related socio-	Higher	professional judgement	Medium

Statistics	
**Scores**	
**BRA**	**47.5**
**BRA Outcome**	**High**
**BRA+CCA**	**59.5**
**BRA+CCA Outcome**	**High**
**Score partition**	
**A. Biogeography/Historical**	**19.5**
1. Domestication/Cultivation	*4.0*
2. Climate, distribution and introduction risk	*2.0*
3. Invasive elsewhere	*13.5*
**B. Biology/Ecology**	**28.0**
4. Undesirable (or persistence) traits	*8.0*
5. Resource exploitation	*7.0*
6. Reproduction	*3.0*
7. Dispersal mechanisms	*5.0*
8. Tolerance attributes	*5.0*
**C. Climate change**	**12.0**
9. Climate change	*12.0*
**Answered Questions**	
Total	***55***
**A. Biogeography/Historical**	**13**

1. Domestication/Cultivation	*3*
2. Climate, distribution and introduction risk	*5*
3. Invasive elsewhere	*5*
**B. Biology/Ecology**	**36**
4. Undesirable (or persistence) traits	*12*
5. Resource exploitation	*2*
6. Reproduction	*7*
7. Dispersal mechanisms	*9*
8. Tolerance attributes	*6*
**C. Climate change**	**6**
9. Climate change	*6*
**Sectors affected**	
**Commercial**	**18**
**Environmental**	**16**
**Species or population nuisance traits**	**31**

**Thresholds**	
**BRA**	**41**
**BRA+CCA**	**41**
**Confidence**	
**BRA+CCA**	**0.58**
**BRA**	**0.60**
**CCA**	**0.46**

**Date and Time**
**20/06/2024 11:18:03**

TPS-ISK v2

**Taxon and Assessor details**	
Category	Angiosperms
Taxon name	*Aesculus hippocastanum*
Common name	Horse Chestnut
Assessor	Sara Essert
**Risk screening context**	
Reason and socio-economic benefits	
Risk assessment area	Continental Europe
Taxonomy	
Native range	
Introduced range	
URL	

			**Response**	**Justification (references or other information)**	**Confidence**
**A. Biogeography/Historical**					
1. Domestication/Cultivation					
1	1.01	Has the taxon been the subject of domestication?	Yes	"Flowers are first produced at 10–15 years old." "Horse-chestnut has, however, been extensively planted since the 17th century across central Europe and south into Italy and Serbia. It is also grown widely in urban areas of Iran, northern India, Asia Minor, United States, Canada (as far north as Edmonton, Alberta) and New Zealand (Kapusta et al., 2007; Loenhart, 2002; Zhang et al., 2010) and further north in the Faeroe Islands, Iceland and Norway (Højgaard, Jóhansen, & Ødum, 1989), still producing fruit at 65°N in Sweden and Norway (Bellini & Nin, 2005)" - Thomas et al (2019) Biological Flora of the British Isles: Aesculus hippocastanum. Journal of Ecology, Volume107, Issue2, Special	Very high
2	1.02	Is the taxon harvested in the wild and likely to be sold or used in its live form?	Yes	"Extracts of seeds, bark and leaves have long been used in traditional and folk medicine (Tiffany et al., 2002). The triterpenoid saponins extracted from the seed have been used as a treatment for rheumatism, coughs, rectal complaints including haemorrhoids, bladder and gastrointestinal disorders, fever (the first written account in 1720) and leg cramps (Anon, 2009; Küçükkurt et al., 2010; Sirtori, 2001; Zhang et al., 2010). In Bosnia and Herzegovina, Redžić (2007) records that horse-chestnut "fruits" are still carried by people who suffer from rheumatism and sciatica. Currently, extracts from horse-chestnut seeds are widely used to treat peripheral vascular disorders including chronic venous insufficiency, haemorrhoids and post-operative oedema (Dickson, Gallagher, McIntyre, Suter, & Tan, 2004; Dudek-Makuch & Studzińska-Sroka, 2015; Facino, Carini, Stefani, Aldini, & Saibene, 1995; Gurel et al., 2013; Pittler & Ernst, 1998; Ruffini, Belcaro, Cesarone, & Dugall, 2004; Suter, Bommer, & Rechner, 2006; Underland, Sæterdal, & Nilsen, 2012), as a preventative of dental plaque and periodontitis in toothpaste (Aravind, Lakshmi, & Arun, 2012; Kim et al., 2017), and to counter male infertility by improving sperm quality (Fang et al., 2010). Saponin extracts have been used to prevent colon cancer in rats (Szabadosova et al., 2013) and may also reduce growth of tumours in a number of cancers in humans (Cheong et al., 2018; Geran, Greenberg, McDonald, Schumacher, & Abbott, 1972; Turkekul et al., 2018)." - Thomas et al (2019) Biological Flora of [illegible]	Very high
3	1.03	Does the taxon have invasive races, varieties, sub-taxa or congeners?	No	Accoridng to: https://www.gbif.org/species/3189801 there are no invasive relatives but the species it self is recognized as invasive in Norway according to: Global Register of Introduced and Invasive Species - Norway	Very high
2. Climate, distribution and introduction risk					
4	2.01	How similar are the climatic conditions between the risk assessment area and the taxon's native range?	High	When comparing the climate where the species has original populations (https://en.wikipedia.org/wiki/Aesculus_hippocastanum) with the climate of the researched area (Peel et al (2007) Updated world map of the K¨oppen-Geiger climate classification, Hydrol. Earth	Very high
5	2.02	What is the quality of the climate matching data?	High	I used the map from the scientific artice M. C. Peel et al.(2007) Updated world K¨oppen-Geiger climate classification map, Hydrol. Earth Syst. Sci., 11, 1633–1644), so I guess that the quality of the data and my assessment is high. Although, the map does not show the climate in detail in terms of altitudes, etc.	High
6	2.03	Is the taxon known to be present outside of cultivation in the risk assessment area?	Yes	Given that the species is recognized as alien species with potentialy high impact in Norway according to the 1) Gederaas, L., Moen, T.L., Skjelseth, S. & Larsen, L.-K. (eds.). Alien species in Norway – with the Norwegian Black List 2012. The Norwegian Biodiversity Information Centre, Norway and Global Register of Introduced and 2) Invasive Species – Norway, https://www.gbif.org/species/3189815 I conclude that it was	Very high

7	2.04	How many potential vectors could the taxon use to enter the risk assessment area?	One	The main vector of entry is by humans in the way that the plant is planted as an ornamental tree in parks, gardens, squares, etc. - "Horse-chestnut has, however, been extensively planted since the 17th century across central Europe and south intoItaly and Serbia..." - Thomas et al (2019) Biological Flora of the British Isles: Aesculus hippocastanum, Journal of Ecology, Volume107, Issue2, Special Feature: Ecological Succession in a Changing World, Pages 992-1030 It is theoretically possible for the plant to spread by seeds, but seeds do not spread over long distances, so	Medium
8	2.05	Is the taxon currently found in close proximity to, and likely to enter, the risk assessment area in the near future (e.g. unintentional or intentional introductions)?	Not applicable	Given that the species is recognized as alien species with potentialy high impact in Norway according to 1)Gederaas, L., Moen, T.L., Skjelseth, S. & Larsen, L.-K. (eds.). Alien species in Norway – with the Norwegian Black List 2012. The Norwegian Biodiversity Information Centre, Norway and Global Register of Introduced and 2) Global Register of Introduced and Invasive Species – Norway, https://www.gbif.org/species/3189815 I conclude that the found species is already naturalised in RA. "In mainland Europe, it is planted in a wide range of urban and garden areas and is naturalised in grassy places, copses, thickets and hedges and rough ground through west and central Europe	Very high
3. Invasive elsewhere					
9	3.01	Has the taxon become naturalised outside its native range?	Yes	"In mainland Europe, it is planted in a wide range of urban and garden areas and is naturalised in grassy places, copses, thickets and hedges and rough ground through west and central Europe (Łukasiewicz, 2003)." Given that the species is recognized as alien species with potentialy high impact in Norway according to 1) Gederaas, L., Moen, T.L., Skjelseth, S. & Larsen, L.-K. (eds.). Alien species in Norway – with the Norwegian Black List 2012. The Norwegian Biodiversity Information Centre, Norway and Global Register of Introduced and 2) Global Register of Introduced and Invasive Species – Norway, https://www.gbif.org/species/3189815 I conclude that the found	Very high
10	3.02	In the taxon's introduced range, are there any known adverse impacts to wild or commercial species?	No	I found no evidence of that, although the species is recognized as alien species with potentialy high impact in Norway.	Medium
11	3.03	In the taxon's introduced range, are there any known adverse impacts to agriculture or forestry?	No	I haven't found any information about it, and it doesn't seem likely that there is a harmful effect.	Medium
12	3.04	In the taxon's introduced range, are there any known adverse impacts to ecosystem	No	I haven't found any information about it, and it doesn't seem likely that there is a harmful effect.	Medium
13	3.05	In the taxon's introduced range, are there any known adverse socio-economic impacts?	No	I haven't found any information about it, and it doesn't seem likely that there is a harmful effect.	Medium
**B. Biology/Ecology**					
4. Undesirable (or persistence) traits					
14	4.01	Is the taxon likely to be poisonous or pose other risks to human health?	Yes	https://www.poison.org/articles/horse-chestnuts-are-toxic-214 - "Horse chestnuts contain a toxin called saponin aesculin that makes all parts of these trees poisonous. This toxin isn't absorbed very well, so it tends to produce mild to moderate symptoms when people eat horse chestnuts."	Very high
15	4.02	Is the taxon likely to suppress the growth of one or more native species?	No	Although the species is recognized as alien species with potentialy high impact in Norway I found no data that the species suppress the growth of one or more native species.	Medium
16	4.03	Are there any threatened or protected native species that the taxon would parasitise in the risk assessment area?	No	It is not a parasitic plant.	Very high
17	4.04	Is the taxon adaptable in terms of climatic and other environmental conditions, thus enhancing its potential persistence if it has invaded or is likely to invade the risk	Yes	Considering that the species is widely distributed across the whole Europe due to horticulture, and has naturalized in many places, I conclude that it is adaptable.	Medium
18	4.05	Is the taxon likely to disrupt terrestrial ecosystem function if it has invaded or is likely to invade the risk assessment area?	Yes	The species is recognized as alien species with potentialy high impact in Norway according to 1) Gederaas, L., Moen, T.L., Skjelseth, S. & Larsen, L.-K. (eds.). Alien species in Norway – with the Norwegian Black List 2012. The Norwegian Biodiversity Information Centre, Norway and Global Register of Introduced and 2) Global Register of Introduced and Invasive Species – Norway, https://www.gbif.org/species/3189815	High
19	4.06	Is the taxon likely to exert adverse impacts on ecosystem services in the risk assessment area?	No	I found no evidence of that and according to the current distribution and behavior of the species in RA, I estimate that the species should not affect ecosystem services.	Medium
20	4.07	Is the taxon likely to host or function as a vector for recognised pests and pathogens that are present in the risk assessment area?	Yes	The horse chestnut leaf-miner (Cameraria ohridella) (and many others) is a very common pest that causes great damage to the species. - Thomas et al (2019) Biological Flora of the British Isles: Aesculus hippocastanum, Journal of Ecology, Volume107, Issue2, Special Feature: Ecological Succession in a Changing World, Pages	Very high
21	4.08	Is the taxon likely to host or function as a vector for recognised pests and pathogens that are absent in the risk assessment area?	Yes	The species has numerous parasites and pests. The most significant pest is Cameraria ohridella. See pages 1006-1014: Thomas et al (2019) Biological Flora of the British Isles: Aesculus hippocastanum, Journal of Ecology, Volume107, Issue2, Special Feature: Ecological Succession in a Changing World, Pages 992-	Very high

22	4.09	Is the taxon likely to be released from cultivation?	Not applicable	The species is already released from cptivity. Species is recognized as alien species with potentialy high impact in Norway according to 1) Gederaas, L., Moen, T.L., Skjelseth, S. & Larsen, L.-K. (eds.). Alien species in Norway – with the Norwegian Black List 2012. The Norwegian Biodiversity Information Centre, Norway and Global Register of Introduced and 2) Global Register of Introduced and Invasive Species – Norway, https://www.gbif.org/species/3189815 I conclude that the found species is already out of cultivation areas in RA. And there are also naturalised populations elswhere in Europe: "In mainland Europe, it is planted in a wide range of urban and garden areas and is naturalised in grassy places, copses, thickets and hedges and rough ground through west and central Europe (Łukasiewicz,	Very high
23	4.10	Is the taxon versatile in habitat use?	No	f	Medium
24	4.11	Is it likely that the taxon's mode of existence or behaviours will reduce habitat quality for native species?	No	https://www.aspca.org/pet-care/animal-poison-control/toxic-and-non-toxic-plants/horse-chestnut - The plant is toxic to animals, but in greatest danger are dogs who want to play with the fruit and consume it while playing. I found no data about cattle or farm animals that were poisoned.	High
25	4.12	Is the taxon likely to maintain a viable population even when present in low densities (or persist in adverse conditions by way of a dormant form)?	Yes	I found no articles about naturalised populations, but I presume that it is not necessary that the population is dense in order to survive.	Medium
5. Resource exploitation					
26	5.01	Is the taxon likely to put threatened or protected native species under competitive pressure in the risk assessment area?	Yes	I found no data about that, but since in Norway there is high risk of impact of Aesculus, it is also possible that the taxon endageres also some endagered species.	Medium
27	5.02	Is the taxon likely to sequester resources to the detriment of native species in the risk assessment area?	Yes	I did not calculate or find RIP, but the species does not have any specialized organs, and considering that it is woody plant - I would say that it will use the light resource the most and cover lower species and possibly have a negative effect on them.	Low
6. Reproduction					
28	6.01	Is the taxon likely to exhibit changes in reproductive strategy in response to environmental conditions?	No	I found no data with proof that the species has efficient reproductive strategy in the case of habitat changes.	Medium
29	6.02	Is the taxon likely to produce viable propagules in the risk assessment area?	Yes	"The species produces seeds in the study area" - Bellini & Nin (2005) Horse Chestnut: Cultivation for Ornamental Purposes and Non-Food Crop Production, Article in Journal of Herbs Spices & Medicinal Plants. The plan is already naturalized (at least) in	Very high
30	6.03	Is the taxon likely to hybridise with native species under natural conditions?	No	On research area native species from the genus Aesculus is only the species A. hippocastanum. There are some Aesculus hybrids, but on the research area there are no A. hippocastanum x native plants hybrids. "Aesculus hippocastanum is known to hybridise with the four North American species of Section Pavia when they are grown together— A. pavia L., A. glabra Willd., A. flava Sol. (=A. octandra Marsh.) and A. sylvatica L. (Hardin, 1957). The only hybrid commonly found in Europe is the red horse-chestnut, A. carnea Willd. (=A. rubicunda Lodd., A. rubicunda Loisel., A. intermedia Andre.), a hybrid of A. hippocastanum and A. pavia (note that as a fully fertile hybrid, the species name is not preceded by "x" by convention). This hybrid is fertile and breeds true (linked to being a tetraploid)." - Thomas et al (2019) Biological Flora of the British Isles: Aesculus hippocastanum, Journal of Ecology, Volume107, Issue2, Special Feature:	Very high
31	6.04	Is the taxon likely to be hermaphroditic or to exhibit asexual reproduction?	Yes	"Reproduction is primarily by seeds, but occasionally, it will spread vegetatively by new shoots growing from adventitious buds on the roots of established trees up to 4–5 m from the trunk (Czekalski, 2005). Hill et al. (2004) classified horse-chestnut as not spreading clonally, but Howard (1945) noted that branches that touch the ground may root and produce new shoots." - Thomas et al (2019) Biological Flora of the British Isles: Aesculus hippocastanum, Journal of Ecology, Volume107, Issue2, Special Feature: Ecological Succession in a Changing World, Pages 992-	Very high
32	6.05	Is the taxon dependent on the presence of another species (or specific habitat features) to complete its life cycle?	No	The taxon is not dependend of any other specific species for reproduction. "Pollination is primarily by insects, but due to the large number of pollen grains produced, wind pollination is considered a viable supplement." - Thomas et al (2019) Biological Flora of the British Isles: Aesculus hippocastanum, Journal of Ecology, Volume107, Issue2, Special Feature: Ecological Succession in a Changing World, Pages 992-1030	Very high
33	6.06	Is the taxon likely to produce a large number of propagules?	No	Since seeds are large I would say the taxon does not produce many seeds: "Seeds are large, each typically 13–20 g fresh mass (Daws et al., 2004; Bonner & Karrfalt, 2008), although are largest (15.3–22.6 g) in street trees in Serbia (Ocokoljić & Stojanović, 2009; Ocokoljić et al., 2013), but smaller (mean 9.9– 14.5 g) in natural populations in Greece (Daws et al., 2004; Tsiroukis, 2008) and smallest (1.2 g) at the northern end of its planted range in Scotland (Daws et al., 2004)."). Athough the numer sounds quite high ("from 2–3 to 25 kg of fresh seeds per tree (Bellini & Nin, 2005), which equates to approximately 125 to 1,600 seeds per tree"- Thomas et al (2019) Biological Flora of the British Isles: Aesculus hippocastanum, Journal of Ecology, Volume107, Issue2, Special Feature: Ecological Succession in a Changing World, Pages 992-1030) I guess that are not many seeds (even for a tree)	Low

34	6.07	How many time units (days, months, years) does the taxon require to reach the age at first reproduction?	>10	"Flowers are first produced at 10–15 years old." - Thomas et al (2019) Biological Flora of the British Isles: Aesculus hippocastanum, Journal of Ecology, Volume107, Issue2, Special Feature: Ecological Succession in a Changing World, Pages 992-	Very high
7. Dispersal mechanisms					
35	7.01	How many potential vectors or pathways could the taxon use to disperse within the risk assessment area (with suitable habitats nearby)?	>1	1) Horticulture 2) Seed dispersal by gravity 3) children 4) rodents 5) water - "Seeds are primarily dispersed by gravity (barochory), with seeds falling from the fruits more or less under the crown of the mother tree. Aesculus turbinata seeds are known to be dispersed by rodents (Hoshizaki et al., 1999) and this is likely to happen, to some extent at least, in horse-chestnut. Seedlings have been seen far away from mature trees in Greece with rodent movement the most likely cause (M. Dering and G. Iszkuło, pers. comm., September 7, 2018). In Britain, other vectors undoubtedly include grey squirrels (Sciurus carolinensis Gmelin), corvids (Briggs, 1989) and children collecting and ultimately discarding conkers. There is also a suggestion that seeds of horse-chestnut are secondarily dispersed by water, particularly during snow-melt, primarily based on the consistent occurrence of horse-chestnut along mountain streams and rivulets in the native Greek populations (Briggs, 1989; Tsiroukis, 2008). However, this is probably a result of dispersal by gravity and the needs of germination and early growth rather than a facet of secondary dispersal." - Thomas et al (2019) Biological Flora of the British Isles: Aesculus hippocastanum, Journal of Ecology, Volume107, Issue2, Special Feature: Ecological Succession in a Changing	Very high
36	7.02	Will any of these vectors or pathways bring the taxon in close proximity to one or more protected areas?	Yes	The plant is widespread across Europe, making it possible, and even likely, that the species is already in the vicinity of the protected areas. It has been recognized as an alien species with potentially high impact in Norway, further increasing the likelihood of its presence, as horticulture is not the only successful	Medium
37	7.03	Does the taxon have a specialised means of attachment such that it enhances the likelihood of dispersal?	No	The capsule epidermis has "spines" (prickles), but the fruit is too heavy, so I do not believe that it can be attached to the animal hair or anything else.	High
38	7.04	Is natural dispersal of the taxon likely to occur as seeds or spores in the risk assessment area?	Yes	1) Seed dispersal by gravity 2) children playing with seed and taking them around 3) rodents 4) water) - "Seeds are primarily dispersed by gravity (barochory), with seeds falling from the fruits more or less under the crown of the mother tree. Aesculus turbinata seeds are known to be dispersed by rodents (Hoshizaki et al., 1999) and this is likely to happen, to some extent at least, in horse-chestnut. Seedlings have been seen far away from mature trees in Greece with rodent movement the most likely cause (M. Dering and G. Iszkuło, pers. comm., September 7, 2018). In Britain, other vectors undoubtedly include grey squirrels (Sciurus carolinensis Gmelin), corvids (Briggs, 1989) and children collecting and ultimately discarding conkers. There is also a suggestion that seeds of horse-chestnut are secondarily dispersed by water, particularly during snow-melt, primarily based on the consistent occurrence of horse-chestnut along mountain streams and rivulets in the native Greek populations (Briggs, 1989; Tsiroukis, 2008). However, this is probably a result of dispersal by gravity and the needs of germination and early growth rather than a facet of secondary dispersal." - Thomas et al (2019) Biological Flora of the British Isles: Aesculus hippocastanum, Journal of Ecology, Volume107, Issue2, Special Feature: Ecological	High
39	7.05	Is natural dispersal of the taxon likely to occur as fragments or seedlings in the risk assessment area?	No	I found no evidence of that occurrence in literature.	Medium
40	7.06	Are any life stages of the taxon likely to migrate into the risk assessment area for reproduction?	Yes	Question similar to the Q 38 "Seeds are primarily dispersed by gravity (barochory), with seeds falling from the fruits more or less under the crown of the mother tree. Aesculus turbinata seeds are known to be dispersed by rodents (Hoshizaki et al., 1999) and this is likely to happen, to some extent at least, in horse-chestnut. Seedlings have been seen far away from mature trees in Greece with rodent movement the most likely cause (M. Dering and G. Iszkuło, pers. comm., September 7, 2018). In Britain, other vectors undoubtedly include grey squirrels (Sciurus carolinensis Gmelin), corvids (Briggs, 1989) and children collecting and ultimately discarding conkers. There is also a suggestion that seeds of horse-chestnut are secondarily dispersed by water, particularly during snow-melt, primarily based on the consistent occurrence of horse-chestnut along mountain streams and rivulets in the native Greek populations (Briggs, 1989; Tsiroukis, 2008). However, this is probably a result of dispersal by gravity and the needs of germination and early growth rather than a facet of secondary dispersal." - Thomas et al (2019) Biological Flora of the British Isles: Aesculus hippocastanum, Journal of Ecology, Volume107, Issue2, Special Feature: Ecological Succession in a	Very high

41	7.07	Are propagules of the taxon likely to be dispersed in the risk assessment area by other species?	Yes	"Aesculus turbinata seeds are known to be dispersed by rodents (Hoshizaki et al., 1999) and this is likely to happen, to some extent at least, in horse-chestnut. Seedlings have been seen far away from mature trees in Greece with rodent movement the most likely cause (M. Dering and G. Iszkuło, pers. comm., September 7, 2018). In Britain, other vectors undoubtedly include grey squirrels (Sciurus carolinensis Gmelin), corvids (Briggs, 1989)" - Thomas et al (2019) Biological Flora of the British Isles: Aesculus hippocastanum, Journal of Ecology, Volume107, Issue2, Special Feature: Ecological Succession in a Changing World, Pages	High
42	7.08	Is dispersal of the taxon along any of the vectors or pathways mentioned in the previous seven Questions (35–41: i.e. either unintentional or intentional) likely to be	Yes	Given that horticulture and intentional planting is one of the main vectors of transmission, rapid spread in RA is certainly possible.	Very high
43	7.09	Is dispersal of the taxon density dependent?	No	I wouldn't say that population density is crucial, but surely a larger number of seeds increases the possibility of the species spreading. I have not found data that this matter has been researched for Aesculus hippocastanum.	Medium
8. Tolerance attributes					
44	8.01	Is the taxon able to withstand being in water for extended periods at some stage of its life cycle?	No	Although there are theses that the seeds are spread secondarily by water, I have not found information that the plant can often or for a long time retain a viable seed in this way. "There is also a suggestion that seeds of horse-chestnut are secondarily dispersed by water, particularly during snow-melt, primarily based on the consistent occurrence of horse-chestnut along mountain streams and rivulets in the native Greek populations (Briggs, 1989; Tsiroukis, 2008). However, this is probably a result of dispersal by gravity and the needs of germination and early growth rather than a facet of secondary dispersal." - Thomas et al (2019) Biological Flora of the British Isles: Aesculus hippocastanum, Journal of Ecology, Volume107, Issue2, Special Feature: Ecological Succession in a Changing World, Pages 992-1030	Low
45	8.02	Is the taxon tolerant of a wide range of soil or air quality conditions?	Yes	The species is often planted and successfully maintained in urban/polluted areas of RA. "Horse-chestnut can tolerate atmospheric pollution and persists in polluted inner cities in Europe. Leaves collected in polluted areas of mainland Europe can appear small, curved and brittle, though the degree of pollution causing this is not stated (Godzik & Sassen, 1978). Pollution can alter the epidermis of leaves, particularly the adaxial surface, leading to it becoming porous or cracked with "abnormal" stomata (Godzik & Halbwachs, 1986; Godzik & Sassen, 1978). Horsechestnut is listed as sensitive to chlorine and moderately sensitive to hydrogen chloride by Khan and Abbasi (2000) but tolerant to sulphur dioxide and hydrogen sulphide (Velagić-Habul, Lazarev, & Custović, 1991); "Horse-chestnut is generally tolerant of saline soils and urban salt spray (Chaney, 1991; Šerá, 2017) although some authors have described it as being sensitive to salt spray (Dobson, 1991)." - Thomas et al (2019) Biological Flora of the British Isles: Aesculus hippocastanum, Journal of Ecology, Volume107, Issue2, Special Feature: Ecological Succession in a	High
46	8.03	Can the taxon be controlled or eradicated in the wild with chemical, biological or other agents and means?	Yes	I would say that it is possible, but I found no research about that. I believe there was no need (so far) to investigate the ways of eradication of the species.	Low
47	8.04	Is the taxon likely to benefit from environmental or human disturbance?	Yes	The taxon does not thrive in unfavorable environmental conditions, but I believe that clearing native vegetation in suitable habitats could encourage the species' growth in those areas, as the reduced competition would provide it with an advantage. I found no researh data abut the matter.	Low
48	8.05	Is the taxon able to tolerate soil acidity, salinity or other parameter levels that are higher or lower than those found in its usual	Yes	"Horse-chestnut is generally tolerant of saline soils and urban salt spray (Chaney, 1991; Šerá, 2017) although some authors have described it as being sensitive to salt spray (Dobson, 1991)."	Medium
49	8.06	Are there effective natural enemies of the taxon present in the risk assessment area?	Yes	The species has numerous parasites and pests (See pages 1006-1014: Thomas et al (2019) Biological Flora of the British Isles: Aesculus hippocastanum, Journal of Ecology, Volume107, Issue2, Special Feature: Ecological Succession in a Changing World, Pages 992-1030), but I found no research if those pests are effective natural enemies that could maintain the population in such a way as to prevent excessive expansion.	Medium
**C. Climate change**					
9. Climate change					
50	9.01	Under the predicted future climatic conditions, are the risks of entry into the risk assessment area posed by the taxon likely to increase, decrease or not change?	Not applicable	The species is already present in RA and grows mostly in horticulture in various climates.	Medium

51	9.02	Under the predicted future climatic conditions, are the risks of establishment within the risk assessment area posed by the taxon likely to increase, decrease or not change?	No change	I found no data about the predictions for the whole RA, but it is evident that the phenology of the species is changing due to climate change (warmer winters, droughts, etc.). However, I found no evidence that it will significantly affect the chance of naturalisation of the species. "Modelling based on the natural range of horse-chestnut suggests that the distribution and abundance of horse-chestnut should not change significantly in the Balkan Peninsula under current climate change scenarios (Walas et al., 2018)." & "Fu et al. (2015) looked at the number of days' advance of leaf unfolding per °C of warming across Europe and found that horse-chestnut advancement had changed from 4.2 ± 1.5 (SD) days/°C in 1980–1994 to 2.1 ± 1.5 days/°C in 1999–2013, a reduction of 2.1 days/°C, the most of the seven species tested."; The species does not prefer droughts ("A. hippocastanum is often considered unsuitable for dry urban areas due to its moderate sensitivity to drought (Hirons & Sjöman, 2018; Roloff, Korn, & Gillner, 2009"), so in many areas od RA it	Medium
52	9.03	Under the predicted future climatic conditions, are the risks of dispersal within the risk assessment area posed by the taxon likely to increase, decrease or not change?	No change	I found no data about the predictions for the whole RA, but it is evident that the phenology of the species is changing due to climate change (warmer winters, droughts, etc.). However, I found no evidence that it will significantly affect the chance of distribution of the species within RA. The species does not prefer droughts ("A. hippocastanum is often considered unsuitable for dry urban areas due to its moderate sensitivity to drought (Hirons & Sjöman, 2018; Roloff, Korn, & Gillner, 2009"), so in many areas od RA it will probably have even smaller risk of distribution.	Low
53	9.04	Under the predicted future climatic conditions, what is the likely magnitude of future potential impacts by the taxon on biodiversity or ecosystem integrity and status?	No change	I found no data about the predictions for the whole RA, but it is evident that the phenology of the species is changing due to climate change (warmer winters, droughts, etc.). However, I found no evidence that it will significantly affect the chance of distribution of the species within RA. The species does not prefer droughts ("A. hippocastanum is often considered unsuitable for dry urban areas due to its moderate sensitivity to drought (Hirons & Sjöman, 2018; Roloff, Korn, & Gillner, 2009"), so in many areas od RA it will probably have even smaller risk of distribution.	Low
54	9.05	Under the predicted future climatic conditions, what is the likely magnitude of future potential impacts by the taxon on ecosystem structure or function?	No change	I found no data about the predictions for the whole RA, but it is evident that the phenology of the species is changing due to climate change (warmer winters, droughts, etc.). However, I found no evidence that it will significantly affect the chance of distribution of the species within RA. The species does not prefer droughts ("A. hippocastanum is often considered unsuitable for dry urban areas due to its moderate sensitivity to drought (Hirons & Sjöman, 2018; Roloff, Korn, & Gillner, 2009"), so in many areas od RA it will probably have even smaller risk of distribution.	Medium
55	9.06	Under the predicted future climatic conditions, what is the likely magnitude of future potential impacts by the taxon on ecosystem services and related socio-	No change	I believe that there will be no influence at all, given that even now there is no data that the species affects ecosystem services.	Medium

**Statistics**	
**Scores**	
**BRA**	**26.0**
**BRA Outcome**	**Medium**
**BRA+CCA**	**26.0**
**BRA+CCA Outcome**	**Medium**
**Score partition**	
**A. Biogeography/Historical**	**5.0**
1. Domestication/Cultivation	*2.0*
2. Climate, distribution and introduction risk	*1.0*
3. Invasive elsewhere	*2.0*
**B. Biology/Ecology**	**21.0**
4. Undesirable (or persistence) traits	*6.0*
5. Resource exploitation	*7.0*
6. Reproduction	*1.0*
7. Dispersal mechanisms	*4.0*
8. Tolerance attributes	*3.0*
**C. Climate change**	**0.0**
9. Climate change	*0.0*
**Answered Questions**	
Total	***55***
**A. Biogeography/Historical**	**13**
1. Domestication/Cultivation	*3*
2. Climate, distribution and introduction risk	*5*
3. Invasive elsewhere	*5*
**B. Biology/Ecology**	**36**
4. Undesirable (or persistence) traits	*12*
5. Resource exploitation	*2*
6. Reproduction	*7*
7. Dispersal mechanisms	*9*
8. Tolerance attributes	*6*
**C. Climate change**	**6**
9. Climate change	*6*
**Sectors affected**	
**Commercial**	**6**
**Environmental**	**7**

Species or population nuisance traits	17

Thresholds	
BRA	41
BRA+CCA	41
**Confidence**	
BRA+CCA	0.68
BRA	0.71
CCA	0.42

Date and Time
09/08/2024 15:34:18

TPS-ISK v2

**Taxon and Assessor details**	
Category	Angiosperms
Taxon name	*Ailanthus altissima*
Common name	Tree of Heaven
Assessor	Ivana Vitasović Kosić
**Risk screening context**	
Reason and socio-economic benefits	
Risk assessment area	Continental Europe
Taxonomy	
Native range	
Introduced range	
URL	

			Response	Justification (references or other information)	Confidence
**A. Biogeography/Historical**					
1. Domestication/Cultivation					
1	1.01	Has the taxon been the subject of domestication?	Yes	In China, the tree of heaven has a long and rich history. It was mentioned in the oldest extant Chinese dictionary and listed in countless Chinese medical texts ...In Europe, the plant first appeared in 1751. In Poland 1808 in the Cracow Botanical Garden (Seneta, 1991), and the status of this specie is domesticated anthropophyte (ZARAŚ-JANUSZKIEWICZ et al 2014)ZARAŚ-JANUSZKIEWICZ, E., ŻARSKA, B., FORNAL-PIENIAK, B., & ROSŁON-SZERYŃSKA, E. (2014). Phenological observations of Ailanthus altissima (Mill.) swingle at different urban areas. Plants	High
2	1.02	Is the taxon harvested in the wild and likely to be sold or used in its live form?	Yes	The roots, leaves and bark are still used today in traditional Chinese medicine, primarily as an astringent. In addition to its use as an ornamental plant, the tree of heaven is also used for its wood, medicinal properties, and as a host plant to feed silkworms of the moth Samia cynthia, which produces silk that is stronger and cheaper than mulberry silk, although with inferior gloss and texture. It is also unable to take dye (Duke, 1983). DUKE, J. A. 1983. Ailanthus altissima. Handbook of Energy Crops. Purdue University Center for New Crops & Plant Products, 1983ZARAŚ-JANUSZKIEWICZ, E., ŻARSKA, B., FORNAL-PIENIAK, B., & ROSŁON-SZERYŃSKA, E. (2014). Phenological observations of Ailanthus altissima (Mill.) swingle at different urban areas. Plants	High
3	1.03	Does the taxon have invasive races, varieties, sub-taxa or congeners?	No	ii didn' find any literature on google sholar	Medium
2. Climate, distribution and introduction risk					
4	2.01	How similar are the climatic conditions between the risk assessment area and the taxon's native range?	Medium	It is native to both northeast and central China and Taiwan. The tree grows rapidly and is capable of reaching heights of 15 metres in 25 years. However, the species is also short lived and rarely lives more than 50 years. With climate change, it will be even more suitable this European areas. ZARAŚ-JANUSZKIEWICZ, E., ŻARSKA, B., FORNAL-PIENIAK, B., & ROSŁON-SZERYŃSKA, E. (2014). Phenological observations of Ailanthus altissima (Mill.) swingle at different urban areas. Plants in Urban Areas and	High
5	2.02	What is the quality of the climate matching data?	High	the climate of China is extremely diverse. It ranges from tropical in the far south to subarctic in the far north, and alpine in the higher elevations of the Tibetan Plateau. Taiwan's climate is subtropical, except for the very southern part of the island, which is tropical. Summers are long and hot, lasting from April or May to September or October. The winters are short and mild, although snow does fall in the mountains and occasionally at lower elevations in the north. The mean annual high temperature on the island is 70 °F (21 °C); elevation makes a considerable difference in the temperature in the winter, though it is not much of a factor	High
6	2.03	Is the taxon known to be present outside of cultivation in the risk assessment area?	Yes	it is well known facts, the Ailanthus is one of the top 100 invasive plants in the worllld.	Very high
7	2.04	How many potential vectors could the taxon use to enter the risk assessment area?	>1	as we can see it is spreaded vegetatively and generatively without any problems	Very high
8	2.05	Is the taxon currently found in close proximity to, and likely to enter, the risk assessment area in the near future (e.g. unintentional or intentional introductions)?	Not applicable	Soler, J., & Izquierdo, J. (2024). The Invasive Ailanthus altissima: A Biology, Ecology, and Control Review. Plants, 13(7), 931. from: A. altissima is present in all continents except Antarctica and prefers areas with high human disturbance [2]. The initial ornamental use of this tree in many gardens and backyards favored its spread; however, because of its invasive traits, ornamental uses have decreased, and the origin of new infestations nowadays lies with existing natural populations [11]. Infestations seem to be dependent on the level of disturbance of the invaded area [12], as a positive correlation is found between the presence of A. altissima along different parts of the Danube river and areas with dense human population [13]. Additionally, it is not affected by urban pollution [14]. It adapts well to a wide range of soils and prefers warm climes [3], although it is able to establish itself in many different climatic conditions [11]. It is well established in the Mediterranean basin, in which many eradication programs have been carried out [15]. liter ref can be found in the	Very high
3. Invasive elsewhere					
9	3.01	Has the taxon become naturalised outside its native range?	Yes	Soler, J., & Izquierdo, J. (2024). The Invasive Ailanthus altissima: A Biology, Ecology, and Control Review. Plants, 13(7), 931.	Very high

10	3.02	In the taxon's introduced range, are there any known adverse impacts to wild or commercial species?	Yes	Soler, J., & Izquierdo, J. (2024). The Invasive Ailanthus altissima: A Biology, Ecology, and Control Review. Plants, 13(7), 931. from paper: apart from physical damage, this species has allelopathic effects. Ailanthone, a quassinoid compound, has exhibited pre- and postemergence herbicidal activity against other species [11]. It can be found in different parts of the tree and negatively affects the growth of different native species [23], allowing an increase in the presence of other non-native species [24]. Assays with concentrated extracts of ailanthone have shown different effects towards different weeds [25], with dicots being the more affected species [26]. Perhaps this fact may explain why the removal of A. altissima in natural areas does not allow native plants to recover until two years later [27]. The modification of bacterial colonies from soil and retardations in the growth of plants under A. altissima canopies have also been reported [28]. literature ref can	Very high
11	3.03	In the taxon's introduced range, are there any known adverse impacts to agriculture or forestry?	Yes	Soler, J., & Izquierdo, J. (2024). The Invasive Ailanthus altissima: A Biology, Ecology, and Control Review. Plants, 13(7), 931. from the paper: As an invasive plant, A. altissima has all the detrimental ecological effects on local ecosystems that have been described for these types of species elsewhere [16,17]. However, it has several particular traits that contribute to generating further damage. The rapid entry into flowering (fourth year), the huge amount of seed production, the wind dispersal, and the vegetative reproduction favor its expansion and enhance its persistency [7]. Its ecological preference for altered and degraded ecosystems and its well-developed root system make this species frequent in communication corridors such as railways, freeways, or walkways [2], in which it damages constructions and pavements and reduces visibility [18], in rural areas along fencerows, woodland edges, or forest openings [2], and in heritage areas such as archaeological monuments, degrading them [19,20,21,22].	Very high
12	3.04	In the taxon's introduced range, are there any known adverse impacts to ecosystem	Yes	Soler, J., & Izquierdo, J. (2024). The Invasive Ailanthus altissima: A Biology, Ecology, and Control Review. Plants, 13(7), 931.	Very high
13	3.05	In the taxon's introduced range, are there any known adverse socio-economic impacts?	Yes	Soler, J., & Izquierdo, J. (2024). The Invasive Ailanthus altissima: A Biology, Ecology, and Control Review. Plants, 13(7), 931. Its presence endangers natural ecosystems by displacing native species, modifying habitats, changing community structures, and affecting ecosystem processes. Its ecological preference for altered and degraded ecosystems and its well-developed root system make this species frequent in communication corridors such as railways, freeways, or walkways [2], in which it damages constructions and pavements and reduces visibility [18], in rural areas along fencerows, woodland edges, or forest openings [2], and in heritage areas such as archaeological monuments, degrading them [19,20,21,22]. But, apart from physical damage	High
**B. Biology/Ecology**					
4. Undesirable (or persistence) traits					
14	4.01	Is the taxon likely to be poisonous or pose other risks to human health?	Yes	Soler, J., & Izquierdo, J. (2024). The Invasive Ailanthus altissima: A Biology, Ecology, and Control Review. Plants, 13(7), 931. But, apart from physical damage, this species has allelopathic effects. Ailanthone, a quassinoid compound, has exhibited pre- and postemergence herbicidal activity against other species [11]. It can be found in different parts of the tree and negatively affects the growth of different native species [23], allowing an increase in the presence of other non-native species [24]. Assays with concentrated extracts of ailanthone have shown different effects towards different weeds [25], with dicots being the more affected species [26]. Perhaps this fact may explain why the removal of A. altissima in natural areas does not allow native plants to recover until two years later [27]. The modification of bacterial colonies from soil and retardations in the growth of plants under A. altissima canopies have also been reported [28].	Very high
15	4.02	Is the taxon likely to suppress the growth of one or more native species?	Yes	Soler, J., & Izquierdo, J. (2024). The Invasive Ailanthus altissima: A Biology, Ecology, and Control Review. Plants, 13(7), 931. Seeds, which are wind dispersed, are the main mechanism by which this species reaches new habitats. When they germinate and develop the root system, roots emit new shoots that contribute to a rapid increase in the tree density and the subsequent expansion of the population nearby. The contradictory results about the ecological requirements for seeds to germinate and their degree of dormancy and longevity indicate the complexity and difficulty of understanding the mechanisms that govern the biology and adaptability of this plant. The management of this weed aims at its eradication, with programs based on herbicide applications carried out by injecting the active ingredient directly to the trunk. But, not many active ingredients have shown total control, so new ones should be tested in order to increase the range of available herbicides. During the last few decades, some biological agents have been identified, but their efficacy in controlling the tree and their safety for the local flora have not yet been determined. A correct management strategy should take into account all these aspects in order to contain the expansion of this species and	Very high
16	4.03	Are there any threatened or protected native species that the taxon would parasitise in the risk assessment area?	No	I did not find any information in the literature about the potential parasitic effect of the species.	Medium

17	4.04	Is the taxon adaptable in terms of climatic and other environmental conditions, thus enhancing its potential persistence if it has invaded or is likely to invade the risk assessment area?	Yes	Motti, R., Zotti, M., Bonanomi, G. et al. Climatic and anthropogenic factors affect Ailanthus altissima invasion in a Mediterranean region. Plant Ecol 222, 1347–1359 (2021). https://doi.org/10.1007/s11258-021-01183-9 Overall, we found that temperature and rainfall in Campania are suitable for Ailanthus, with the only major constraint being the temperature at an altitude exceeding 900 m a.s.l.. We found that Ailanthus is unable to spread where the mean annual temperature is lower than 11.1 °C. By contrast, precipitation variables showed poor correlation with Ailanthus distribution, suggesting that rainfall in the selected study sites is suitable to sustain the growth of this tree. About land use variables, roads were the primary landscape feature along which this species spread and invaded new areas. Roads probably combine high propagule pressure and favorable growing conditions in terms of available resources i.e., light, water, and mineral nutrients, that allow Ailanthus to establish and spread along roadside edges in different ecosystems. In conclusion, we found that climate and human-associated variables are correlated with the current occurrence of Ailanthus, with the temperature being more influential at high elevation sites and	Very high
18	4.05	Is the taxon likely to disrupt terrestrial ecosystem function if it has invaded or is likely to invade the risk assessment area?	Yes	Sladonja, B., Sušek, M. & Guillermic, J. Review on Invasive Tree of Heaven (Ailanthus altissima (Mill.) Swingle) Conflicting Values: Assessment of Its Ecosystem Services and Potential Biological Threat. Environmental Management 56, 1009–1034 (2015). https://doi.org/10.1007/s00267-015-0546-5 from this paper: There it is shown to negatively impact the environment by altering the local vegetation structure and compromising the ecosystem stability (Vilá et al. 2006; Constán-Nava et al. 2010). biodiversity decrease: Table 3 Ailanthus altissima negative effects From: Review on Invasive Tree of Heaven (Ailanthus altissima (Mill.) Swingle) Conflicting Values: Assessment of Its Ecosystem	Very high
19	4.06	Is the taxon likely to exert adverse impacts on ecosystem services in the risk assessment area?	Yes	neggatove effects on enironment (loss of biodiversity...) and human health Soler, J., & Izquierdo, J. (2024). The Invasive Ailanthus altissima: A Biology, Ecology, and Control Review. Plants, 13(7), 931. Motti, R., Zotti, M., Bonanomi, G. et al. Climatic and anthropogenic factors affect Ailanthus altissima invasion in a Mediterranean region. Plant Ecol 222, 1347–1359 (2021). https://doi.org/10.1007/s11258-021-01183-9 Sladonja, B., Sušek, M. & Guillermic, J. Review on Invasive Tree of Heaven (Ailanthus altissima (Mill.) Swingle) Conflicting Values: Assessment of Its Ecosystem Services and Potential Biological Threat. Environmental Management 56, 1009–1034 (2015).	Very high
20	4.07	Is the taxon likely to host or function as a vector for recognised pests and pathogens that are present in the risk assessment area?	Yes	Regarding biological control, A. altissima acts as a host for the invasive pest Lycorma delicatula in North America. here in Europe are similar climatic conditions, so I beieve thee is a great chance. ref: Kreitman, D.; Keena, M.A.; Nielsen, A.L.; Hamilton, G. Effects of temperature on development and survival of Nymphal Lycorma delicatula (Hemiptera: Fulgoridae). Environ. Entomol. 2021, 50, 183–191. [Google Scholar] [CrossRef]	High
21	4.08	Is the taxon likely to host or function as a vector for recognised pests and pathogens that are absent in the risk assessment area?	Yes	yes, because in North America; Asia and in UK is already present, and our climate here is very similar for the pests Lycorma deliculata: chrome-extension://efaidnbmnnnibpcajpcglclefindmkaj/https://planthealthportal.defra.gov.uk/pests-and-diseases/uk-plant-health-risk-register/downloadExternalPra.cfm?id=4463 Rapid Pest Risk Analysis (PRA) for: Lycorma delicatula Date in format: July 2023 Summary and conclusions of the rapid PRA This rapid PRA was requested as Lycorma delicatula has been spreading rapidly in the northeast states of the United States of America (USA) having first been reported there in 2014. The pest, which is of Asian origin, has been added to Great Britain (GB) legislation as a Provisional Quarantine Pest, and this PRA has been developed to determine if it should become a Regulated Quarantine Pest. It is already regulated in Northern Ireland. Risk of entry The main pathways of entry are hitchhiking, plants for planting and wood products from countries with L. delicatula (USA and east Asia). Due to the nature of hitchhiking in the inconspicuous egg stage especially, the risk is rated very likely with high confidence as this has been the main mode of large-scale movement of L. delicatula. The second pathway on plants for planting, is moderately likely with low confidence. It could be a future pathway if countries that trade in large volumes of plants for planting with the United Kingdom (UK) become infested but this is uncertain and so it is rated with low confidence. climate change will likely increase the number of hot days in summer months, which might increase population growth. Ailanthus altissima, commonly called tree of heaven, is the preferred host and is found throughout the south of the UK. Therefore, if L. delicatula enters into the south of the UK, then establishment outdoors has been judged unlikely with low [illegible]	Very high
22	4.09	Is the taxon likely to be released from cultivation?	Yes	A. altissima is horticultural species	Very high

23	4.10	Is the taxon versatile in habitat use?	Yes	Soler, J., & Izquierdo, J. (2024). The Invasive Ailanthus altissima: A Biology, Ecology, and Control Review. Plants, 13(7), 931. It adapts well to a wide range of soils and prefers warm climes [3], although it is able to establish itself in many different climatic conditions [11]. It is well established in the Mediterranean basin, in which many eradication programs have been carried out [15]. 3- Miller, J.H. Ailanthus altissima (Mill.) Swingle. Ailanthus. Silv. N. Am. 1990, 2, 101–104. 11 - Sladonja, B.; Sušek, M.; Guillermic, J. Review on invasive Tree of Heaven (Ailanthus altissima (Mill.) Swingle) conflicting values: Assessment of its ecosystem services and potential biological threat. Environ. Manag. 2015, 56, 1009–1034. [Google Scholar] [CrossRef] [PubMed] 15 - Brunel, S.; Brundu, G.; Fried, G. Eradication and control of Invasive Alien Plants in the Mediterranean Basin: Towards Better Coordination to Enhance Existing Initiatives. Bull. OEPP/EPPO Bull. 2013, 43	Very high
24	4.11	Is it likely that the taxon's mode of existence or behaviours will reduce habitat quality for native species?	Yes	Soler, J., & Izquierdo, J. (2024). The Invasive Ailanthus altissima: A Biology, Ecology, and Control Review. Plants, 13(7), 931.	Very high
25	4.12	Is the taxon likely to maintain a viable population even when present in low densities (or persist in adverse conditions by way of a dormant form)?	Yes	Soler, J., & Izquierdo, J. (2024). The Invasive Ailanthus altissima: A Biology, Ecology, and Control Review. Plants, 13(7), 931. Seeds are mainly dispersed by wind [9], but they can remain in the canopy throughout the winter [5]. The species has a root system with high potential to expand by producing new shoots that contribute to its vegetative dispersion. Most of the roots are found in the first 46 cm of soil [3], with roots sprouts found up to 27 m from the parent tree [10]. More information about the biology of this species can be found in [11]. Howard, J.L. Ailanthus altissima. In Fire Effects Information System; US Department of Agriculture, Forest Service, Rocky Mountain Research Station, Fire Sciences Laboratory (Producer): Fort Collins, CO, USA, 2004. [Google Scholar] Sladonja, B.; Sušek, M.; Guillermic, J. Review on invasive Tree of Heaven (Ailanthus altissima (Mill.) Swingle) conflicting values: Assessment of its ecosystem services and potential biological threat. Environ. Manag. 2015, 56, 1009–1034.	Very high
5. Resource exploitation					
26	5.01	Is the taxon likely to put threatened or protected native species under competitive pressure in the risk assessment area?	Yes	It also produces various herbicidal compounds, the most famous of which is ailanton, whose accumulation in the soil prevents the growth of native plants and affects the soil fauna. https://lifeailanthus.hr/o-pajasenu/ Does the invasive species Ailanthus altissima threaten floristic diversity of temperate peri-urban forests? Motard E, Muratet A, Clair-Maczulajtys D, Machon N. C R Biol. 2011 Dec;334(12):872-9. doi: 10.1016/j.crvi.2011.06.003. Epub 2011 Sep 15. PMID: 22123089 We examined the influence of the invasive species Ailanthus altissima (Mill.) Swingle on the understory of the Fontainebleau forest, a peri-urban forest of Paris (France), by comparing invaded versus control plots. We performed floristic inventories in fixed plots around the base of A. altissima vs native trees in different habitat types of the forest. Our findings suggest that the understory vegetation is significantly poorer and more common under A. altissima than under the other tree species and that the floristic composition is significantly different. Furthermore, the number of A. altissima root suckers growing in the plots was significantly negatively correlated with floristic richness. This effect can be attributed to both interspecific competition and allelopathic properties of A. altissima. These results give an	Medium
27	5.02	Is the taxon likely to sequester resources to the detriment of native species in the risk assessment area?	Yes	Does the invasive species Ailanthus altissima threaten floristic diversity of temperate peri-urban forests? Motard E, Muratet A, Clair-Maczulajtys D, Machon N.C R Biol. 2011 Dec;334(12):872-9. doi: 10.1016/j.crvi.2011.06.003. Epub 2011 Sep 15.PMID: 22123089 We examined the influence of the invasive species Ailanthus altissima (Mill.) Swingle on the understory of the Fontainebleau forest, a peri-urban forest of Paris (France), by comparing invaded versus control plots. We performed floristic inventories in fixed plots around the base of A. altissima vs native trees in different habitat types of the forest. Our findings suggest that the understory vegetation is significantly poorer and more common under A. altissima than under the other tree species and that the floristic composition is significantly different. Furthermore, the number of A. altissima root suckers growing in the plots was significantly negatively correlated with floristic richness. This effect can be attributed to both interspecific competition and allelopathic properties of A. altissima. These results give an estimate of the threat to biodiversity ascribed to A.	Very high
6. Reproduction					

28	6.01	Is the taxon likely to exhibit changes in reproductive strategy in response to environmental conditions?	Yes	Does the invasive species Ailanthus altissima threaten floristic diversity of temperate peri-urban forests?Motard E, Muratet A, Clair-Maczulajtys D, Machon N.C R Biol. 2011 Dec;334(12):872-9. doi: 10.1016/j.crvi.2011.06.003. Epub 2011 Sep 15.PMID: 22123089 We examined the influence of the invasive species Ailanthus altissima (Mill.) Swingle on the understory of the Fontainebleau forest, a peri-urban forest of Paris (France), by comparing invaded versus control plots. We performed floristic inventories in fixed plots around the base of A. altissima vs native trees in different habitat types of the forest. Our findings suggest that the understory vegetation is significantly poorer and more common under A. altissima than under the other tree species and that the floristic composition is significantly different. Furthermore, the number of A. altissima root suckers growing in the plots was significantly negatively correlated with floristic richness. This effect can be attributed to both interspecific competition and allelopathic properties of A. altissima. These results give an estimate of the threat to biodiversity ascribed to A. altissima in the Fontainebleau forest. AILANTHUS ALTISSIMA INTERFERS WITH BENEFICIAL SYMBIONTS AND NEGETIVELY IMPACTS OAK REGENERATION1 Jenise M. Bauman2, Caitlin Byrne and Shiv Hiremath The invasion of Tree-of-Heaven (Ailanthus altissima) has been documented in disturbed landscapes leading to biodiversity loss and degradation of ecosystem function. Ailanthus interferes with the restoration of native species by its aggressive growth habit, alteration of nutrient cycles, and allelopathic chemical production. Recent studies suggest that allelopathy has a negative effect on the growth of red oak (Quercus rubra), possibly by interfering with the symbiosis of beneficial ectomycorrhizal fungi (ECM). This fungal symbiont is essential for healthy tree growth and the unavailability of these fungi may impede the success of seedling regeneration. This study investigated the effects of Ailanthus on biomass production and ectomycorrhizal fungal (ECM) colonization of red oak (Q. rubra) seedlings on a reclaimed coal mine site in eastern Ohio. Six plots were designated in an existing riparian buffer zone in a wetland at The Wilds Conservation Center in Muskingum County. Three of the plots were in an area where mature Ailanthus was present. The other three plots were located in the same riparian zone that was without Ailanthus. Naturally regenerating twoyear-old red oak seedlings were selected for study (10 seedlings per plot, 60 seedlings total). The oak seedlings were sampled for biomass (g) and ECM root colonization. Two-year-old oak seedlings growing among mature Tree-ofHeaven produced significantly less biomass, specifically in root production, than the oaks growing without the invasive tree (P = 0.02). There was a decrease in ECM colonization (P = 0.001) and a shift in ECM community composition in plots where the Tree-of-Heaven was present (P = 0.0004). The increase in root biomass and ECM colonization may	Very high
29	6.02	Is the taxon likely to produce viable propagules in the risk assessment area?	Yes	Motti, R., Zotti, M., Bonanomi, G. et al. Climatic and anthropogenic factors affect Ailanthus altissima invasion in a Mediterranean region. Plant Ecol 222, 1347–1359 (2021). https://doi.org/10.1007/s11258-021-01183-9 Since Ailanthus is a wind dispersal species with heavy seeds, corridors such as roads could be especially effective in promoting dispersal of large seeds that otherwise would have a limited dispersal capability in a closed forest. Invasion, however, depends on both propagule pressure and favorable growing conditions that determine community invasibility (Lonsdale 1999). In this regard, roads provide a suitable habitat for Ailanthus establishment and growth. Roadsides are subject to frequent disturbance due to vehicle passage, periodic cleaning activities that include vegetation cutting and, sometimes, localized fire. As a consequence, roadsides may provide more resources compared to surrounding plant communities both in terms of soil nutrients and light availability (Trombulak and Frissell 2000). Trombulak SC, Frissell CA (2000) Review of ecological effects of roads on terrestrial and	Very high

30	6.03	Is the taxon likely to hybridise with native species under natural conditions?	No	European Commission. Regulation (EU) No 1143/2014 of the European Parliament and of the Council of 22 October 2014 on the prevention and management of the introduction and spread of invasive alien species. Off. J. Eur. Union 2014, 57, 35–55. [Google Scholar] Ailanthus altissima is one of the most important IAS in Europe, also due to its wide presence. Montecchiari, S., Tesei, G., & Allegrezza, M. (2020). Ailanthus altissima forests determine a shift in herbaceous layer richness: A paired comparison with hardwood native forests in sub-Mediterranean Europe. Plants, 9(10), 1404. We analyzed how species richness, diversity, life forms, life strategies, structural characteristics, and ecological parameters changed in A. altissima forests compared with native ones. We found that in A. altissima forests, there is a shift in herbaceous layer richness, with a higher presence of annual ruderal herbs and the absence of herbaceous species linked to the forest environment. The ecological parameters that diverge from the native forests were total nitrogen, total carbon, and C/N ratio. A. altissima forest communities could threaten the biodiversity of the native forest ecosystems in the sub-Mediterranean landscape, favoring ruderal species and inhibiting the presence of typical	Medium
31	6.04	Is the taxon likely to be hermaphroditic or to exhibit asexual reproduction?	Yes	It spreads aggressively both by seeds and vegetatively by root sprouts, re-sprouting rapidly after being cut. Miller, James H. (1990). "Ailanthus altissima". In Burns, Russell M.; Honkala, Barbara H. (eds.). Hardwoods. Silvics of North America. Vol. 2. Washington, D.C.: United States Forest Service (USFS), United States Department of Agriculture (USDA). Retrieved 7 February 2002 – via Southern Research Station. TREE OF HEAVEN – PLANT FROM HELL The LIFE CONTRA Ailanthus – Establishing control of Ailanthus in protected habitats of Mediterranean Croatia. The LIFE CONTRA Ailanthus team plans to establish control of Ailanthus in protected habitats of two Natura 2000 sites and historic cities of Mediterranean Croatia. Duration of the project: 1. 10. 2020. – 1. 4. 2025. the results showed that some individuals hybridized with each other and were hermatophrodites	Very high
32	6.05	Is the taxon dependent on the presence of another species (or specific habitat features) to complete its life cycle?	No	there is no literature data about, so I believe that there is no.	High
33	6.06	Is the taxon likely to produce a large number of propagules?	Yes	it is well known fact for A. altissima ; germination studies and complementary tetrazolium assays also confirmed high propagule viability from a 7-year-old Ailanthus and supports tetrazolium assays as a proxy for germination studies. Seed Production, Viability, and Reproductive Limits of the Invasive Ailanthus altissima (Tree-of-Heaven) within Invaded Environments by Kristen L. Wickert 1,Eric S. O'Neal 2,Donald D. Davis 3 andMatthew T. Kasson 1Forests 2017, 8(7), 226;	High
34	6.07	How many time units (days, months, years) does the taxon require to reach the age at first reproduction?	>10	Seed Production, Viability, and Reproductive Limits of the Invasive Ailanthus altissima (Tree-of-Heaven) within Invaded Environments by Kristen L. Wickert 1,Eric S. O'Neal 2,Donald D. Davis 3 andMatthew T. Kasson 1Forests 2017, 8(7), 226; https://doi.org/10.3390/f8070226 The success of some invasive tree species is attributed, in part, to high fecundity in the form of sexual propagules. If invasive trees produce more seed annually than co-occurring native trees, they will have a greater ability to disperse and establish across the landscape. In this study, seed production of female Ailanthus trees was investigated to determine (1) reproductive age limits; (2) annual and cumulative seed output; and (3) seed viability. Existing data on Ailanthus seed production were combined with a novel dataset to compare variability in seed production and explore relationships with tree diameter and age. Results from this study showed Ailanthus' reproductive window is exceptional, spanning more than a century, with seed viability exceeding 65% from a 104-year-old individual. Germination studies and complementary tetrazolium assays also confirmed high propagule viability from a 7-year-old Ailanthus and supports tetrazolium assays as a proxy for germination studies. Not only can individual Ailanthus produce >1 million seeds annually, but a significant relationship exists between seed production and tree diameter. Using this relationship, cumulative seed production in individual Ailanthus can reach ca. 10 and 52 million seeds over a 40-year and 100- [illegible]	Very high
7. Dispersal mechanisms					
35	7.01	How many potential vectors or pathways could the taxon use to disperse within the risk assessment area (with suitable habitats nearby)?	>1	it is already well known fact	Very high
36	7.02	Will any of these vectors or pathways bring the taxon in close proximity to one or more protected areas?	Yes	already present almoust in all National parks in Croatia (NP Krka, UNESCO site Dubrovnik walls ect)	Very high

37	7.03	Does the taxon have a specialised means of attachment such that it enhances the likelihood of dispersal?	Yes	Seed Production, Viability, and Reproductive Limits of the Invasive Ailanthus altissima (Tree-of-Heaven) within Invaded Environments † by Kristen L. Wickert 1,Eric S. O'Neal 2,Donald D. Davis 3 andMatthew T. Kasson 1Forests 2017, 8(7), 226; https://doi.org/10.3390/f8070226 type of fruit - Not only can individual Ailanthus produce >1 million seeds annually, but a significant relationship exists between seed production and tree diameter. Using this relationship, cumulative seed production in individual Ailanthus can reach ca. 10 and 52 million seeds over a	Medium
38	7.04	Is natural dispersal of the taxon likely to occur as seeds or spores in the risk assessment area?	Yes	The seeds are enclosed in yellow to red samaras, whose color, size, weight, and thickness are highly variable, and dependent on the location of the tree (Kowarik and Säumel 2007). The samaras are numerous, arranged in clusters on trees, and are primarily dispersed by wind. Roots consist of taproot for storage, and extensive lateral roots, highly variable in length, but known to reach up to 27 m (Kiermeier 1987; Kowarik and Säumel 2007). from: Sladonja, B., Sušek, M. & Guillermic, J. Review on Invasive Tree of Heaven (Ailanthus altissima (Mill.) Swingle) Conflicting Values: Assessment of Its Ecosystem Services and Potential Biological Threat. Environmental Management 56, 1009–1034 (2015). https://doi.org/10.1007/s00267-015-0546-5 Kowarik I, Säumel I (2007) Biological flora of Central Europe: Ailanthus altissima (Mill.) Swingle. Perspec Plant Ecol Evol Syst 8:207–237	Very high
39	7.05	Is natural dispersal of the taxon likely to occur as fragments or seedlings in the risk assessment area?	Yes	it is already well known fact	Very high
40	7.06	Are any life stages of the taxon likely to migrate into the risk assessment area for reproduction?	Yes	Vegetative Reproduction Asexual reproduction is an important trait to consider for this species, as new shoots from roots act as a dispersion mechanism [11]. The absence of a taproot is common [3] and a root system that has an asymmetrical shape, adapted to the soil characteristics [2]. New shoots can appear from stumps or roots, and the shapes of leaves vary if they appear from root sprouts (from unifoliolate to pentafoliolatemore or others) or seedlings (trifoliolate) [5]. When aerial parts suffer damage or die, new shoots from the root system appear [3]. from:Soler, J., & Izquierdo, J. (2024). The Invasive Ailanthus altissima: A Biology, Ecology, and Control Review. Plants, 13(7), 931. The tree of heaven is an opportunistic plant that thrives in full sun and disturbed areas. It spreads aggressively both by seeds and vegetatively by root sprouts, re-sprouting rapidly after being cut. from: Miller, James H. (1990). "Ailanthus altissima". In Burns, Russell M.; Honkala, Barbara H. (eds.). Hardwoods. Silvics of North America. Vol. 2. Washington, D.C.: United States Forest	Very high
41	7.07	Are propagules of the taxon likely to be dispersed in the risk assessment area by other species?	Yes	I believe yes	Medium
42	7.08	Is dispersal of the taxon along any of the vectors or pathways mentioned in the previous seven Questions (35–41: i.e. either unintentional or intentional) likely to be	Yes	already mentioned	High
43	7.09	Is dispersal of the taxon density dependent?	Yes	I believe it is not,The trees have a very heterogeneous distribution and it is difficult to clearly explain the large differences in density. from:ACTA CLIMATOLOGICA ET CHOROLOGICA Universitatis Szegediensis, Tomus 49-50, 2016, 920 ASSESSMENT OF TREE OF HEAVEN (AILANTHUS ALTISSIMA) SPREAD DYNAMICS ON EXAMPLE OF SZEGED (HUNGARY) ÁK CSETE, E TANÁCS and Á GULYÁS	Medium
8. Tolerance attributes					
44	8.01	Is the taxon able to withstand being in water for extended periods at some stage of its life cycle?	Yes	Rebbeck, J., & Jolliff, J. (2018). How long do seeds of the invasive tree, Ailanthus altissima remain viable?. Forest Ecology and Management, 429, 175-179. Beside, Ailanthus seeds buried at 10 cm remain viable beyond five years. Kaproth and McGraw (2008) reported that Ailanthus seeds submerged in water for five months retained high germination rates (94%). These traits verify its potential for high fecundity in unfavorable growing environments. from: Kaproth, M.A., and McGraw, J.B., 2008. Seed viability and dispersal of the winddispersed invasive Ailanthus altissima in aqueous environments. Forest Science 54: 490496.	Very high
45	8.02	Is the taxon tolerant of a wide range of soil or air quality conditions?	Yes	It adapts well to a wide range of soils and prefers warm climes [3], although it is able to establish itself in many different climatic conditions [11]. It is well established in the Mediterranean basin, in which many eradication programs have been carried out [15] from: Soler, J., & Izquierdo, J. (2024). The Invasive Ailanthus altissima: A Biology, Ecology, and Control Review. Plants, 13(7),	Very high

46	8.03	Can the taxon be controlled or eradicated in the wild with chemical, biological or other agents and means?	Yes	from: Soler, J., & Izquierdo, J. (2024). The Invasive Ailanthus altissima: A Biology, Ecology, and Control Review. Plants, 13(7), 931. Herbicides are the most popular method to manage A. altissima populations [75]. Systemic herbicides are the most efficient particularly when applied at the end of the growing season because they are transported to the root system via the phloem with the descending movement of the sap [69]. The application of the herbicides on the trees is performed by means of different techniques: stem injection, basal bark, or cut stump. Stem injection is performed by making holes with a drill into the trunk and filling each hole with herbicide, by the E-Z-ject Lance system (injecting into the trunk solid capsules containing herbicide) or by hack-and-squirt which is spraying into cuts performed with a hack along the stem. Basal bark consists of spraying herbicides into the lower part of an uncut trunk. Cut stump consists of spraying or injecting the herbicide on the cut surface of the trunk. Herbicides have been applied diluted and undiluted, with different results. The best results with the stem injection technique were observed using undiluted glyphosate and making holes [76]. However, undiluted triclopyr applied by hack-and-squirt showed no total effectiveness over the trees [73]. The E-Z-ject Lance system has been tested with triclopyr and imazapyr [77,78], and glyphosate [71,78,79] with varied efficacy. The efficacy of basal bark applications depended on the diameter of the tree. Diluted triclopyr showed good control in most cases [70,74,77,79], but diluted mixtures of triclopyr + fluroxypyr, aminopyralid + fluroxypyr or glyphosate alone did not show total mortality when applied to trees with bigger diameters [21]. For cut stump applications, mortality seems to depend on the concentration of the active ingredient. Spraying diluted active ingredients alone (i.e., triclopyr or glyphosate) or mixed (i.e., triclopyr + fluroxypyr, aminopyralid + fluroxypyr or glyphosate) did not achieve total mortality of the trees [21,73], while the same active ingredients undiluted reached total control [76]. Another case is granular herbicides like Metsulfuron methyl, where undiluted applications are not possible, which had a great mortality but not all trees died [74].The chemical does not, however, affect the tree of heaven's own seedlings, indicating that A. altissima has a defence mechanism to prevent autotoxicity. Mites have been reported to attack leaves, with the genus Aculus spp. being the most mentioned ones [81,82,83,84]. Coleoptera such as Eucryptorrhynchus brandti and Eucryptorrhynchus chinensis have shown good specificity over A. altissima in China [80]. These coleoptera also showed good specificity in quarantine trials, preferring this tree over others when feeding at the larval stage and for oviposition [75,85,86] making them a good option for biological control. In Italy, the orange whitefly (Leurocanthus spiniferus) has been reported on A. altissima for the first time, but this insect cannot be considered a biological agent because the	Medium
47	8.04	Is the taxon likely to benefit from environmental or human disturbance?	Yes	it is already well known fact.	Very high
48	8.05	Is the taxon able to tolerate soil acidity, salinity or other parameter levels that are higher or lower than those found in its usual	Yes	it is still unexplored thematic in the literature	Low
49	8.06	Are there effective natural enemies of the taxon present in the risk assessment area?	No	No natural enemies are known so far, and the tree is nearly unaffected by predation by wild ungulates due to the unpalatability of its above-ground parts. Its competitive ability is further enhanced by a strong allelopathic effect caused by quassinoid compounds and indole alkaloids (ailanthone, chaparrinone) that are produced and released by the roots into the soil (Heisey, 1997). These compounds prevent other species from growing with it or under its canopy, which is why the tree can form pure stands. Overall, these features make A. altissima one of the most invasive plant species at a global scale and a serious threat to a wide range of natural habitats, whose biodiversity and functioning can be irreversibly lost or altered once the species has established there. For this reason, the species is classified as a noxious weed and as invasive or a major invader in many regions (e.g., Hunter, 2000; Weber, 2017; Brunel & Tison, 2005; Wittenberg, 2005). from : Metal concentrations in invasive Ailanthus altissima vs native Fraxinus ornus on ultramafic soils: Evidence for higher efficiency in Ni exclusion and adjustments to Mg and Ca imbalance Federico Selvi, Isabella Bettarini, Marco Gabrucci, Ilaria Colzi, Andrea Coppi, Lorenzo	High
**C. Climate change**					
9. Climate change					

50	9.01	Under the predicted future climatic conditions, are the risks of entry into the risk assessment area posed by the taxon likely to increase, decrease or not change?	Increase	Isler, J., Bugmann, H., Conedera, M. et al. Long-term dynamics of tree of heaven (Ailanthus altissima) in central European forests. Eur J Forest Res 142, 1149–1166 (2023). https://doi.org/10.1007/s10342-023-01582-9 Overall, our findings suggest that in the long run, a competitive displacement of native dominant species by A. altissima appears unlikely, with the exception of drought-prone sites under strong climate change. Furthermore, our findings underline the importance of the frequency and intensity of forest management for the long-term abundance of A. altissima in forest stands. Based on these results, it is plausible to expect an increase in the abundance of A. altissima and thus a change in the tree species composition in the long term, especially at managed sites. However, our results do not indicate a complete displacement of the indigenous tree species by A. altissima, but rather a persistence of A. altissima as an additional admixed tree species. This persistence may furthermore be promoted by climate change, particularly on	Very high
51	9.02	Under the predicted future climatic conditions, are the risks of establishment within the risk assessment area posed by the taxon likely to increase, decrease or not	Increase	already mention in question before	High
52	9.03	Under the predicted future climatic conditions, are the risks of dispersal within the risk assessment area posed by the taxon likely to increase, decrease or not change?	Increase	Isler, J., Bugmann, H., Conedera, M. et al. Long-term dynamics of tree of heaven (Ailanthus altissima) in central European forests. Eur J Forest Res 142, 1149–1166 (2023). https://doi.org/10.1007/s10342-023-01582-9 Based on these results, it is plausible to expect an increase in the abundance of A. altissima and thus a change in the tree species composition in the long term, especially at managed sites. However, our results do not indicate a complete displacement of the indigenous tree species by A. altissima, but rather a persistence of A. altissima as an additional admixed tree species. This persistence may furthermore be promoted by climate change, particularly on	High
53	9.04	Under the predicted future climatic conditions, what is the likely magnitude of future potential impacts by the taxon on biodiversity or ecosystem integrity and status?	Higher	already mentioned, Ailanthus influence loos of biodiversity, and future climate changes to an even warmer period favor the species Isler, J., Bugmann, H., Conedera, M. et al. Long-term dynamics of tree of heaven (Ailanthus altissima) in central European forests. Eur J Forest Res 142, 1149–1166 (2023). https://doi.org/10.1007/s10342-023-01582-9	High
54	9.05	Under the predicted future climatic conditions, what is the likely magnitude of future potential impacts by the taxon on ecosystem structure or function?	Higher	already said in question before Isler, J., Bugmann, H., Conedera, M. et al. Long-term dynamics of tree of heaven (Ailanthus altissima) in central European forests. Eur J Forest Res 142, 1149–1166 (2023). https://doi.org/10.1007/s10342-023-01582-9	High
55	9.06	Under the predicted future climatic conditions, what is the likely magnitude of future potential impacts by the taxon on ecosystem services and related socio-economic factors?	Higher	Isler, J., Bugmann, H., Conedera, M. et al. Long-term dynamics of tree of heaven (Ailanthus altissima) in central European forests. Eur J Forest Res 142, 1149–1166 (2023). https://doi.org/10.1007/s10342-023-01582-9 Seed Production, Viability, and Reproductive Limits of the Invasive Ailanthus altissima (Tree-of-Heaven) within Invaded Environments by Kristen L. Wickert 1,Eric S. O'Neal 2,Donald D. Davis 3 andMatthew T. Kasson 1Forests 2017, 8(7), 226; https://doi.org/10.3390/f8070226 Brunel, S.; Brundu, G.; Fried, G. Eradication and control of Invasive Alien Plants in the Mediterranean Basin: Towards Better Coordination to Enhance Existing Initiatives. Bull. OEPP/EPPO Bull. 2013, 43, 290–308. [Google Scholar] [CrossRef] Sladonja, B., Sušek, M. & Guillermic, J. Review on Invasive Tree of Heaven (Ailanthus altissima (Mill.) Swingle) Conflicting Values: Assessment of Its Ecosystem Services and Potential Biological Threat. Environmental Management 56, 1009–1034 (2015). https://doi.org/10.1007/s00267-015-0546-5 Soler, J., & Izquierdo, J. (2024). The Invasive Ailanthus altissima:	Very high

Statistics	
**Scores**	
**BRA**	**50.0**
**BRA Outcome**	**High**
**BRA+CCA**	**62.0**
**BRA+CCA Outcome**	**High**
**Score partition**	
**A. Biogeography/Historical**	**13.0**
1. Domestication/Cultivation	*2.0*
2. Climate, distribution and introduction risk	*2.0*
3. Invasive elsewhere	*9.0*
**B. Biology/Ecology**	**37.0**
4. Undesirable (or persistence) traits	*11.0*
5. Resource exploitation	*7.0*
6. Reproduction	*3.0*
7. Dispersal mechanisms	*9.0*
8. Tolerance attributes	*7.0*
**C. Climate change**	**12.0**
9. Climate change	*12.0*
**Answered Questions**	
Total	***55***
**A. Biogeography/Historical**	**13**
1. Domestication/Cultivation	*3*
2. Climate, distribution and introduction risk	*5*

3. Invasive elsewhere	*5*
**B. Biology/Ecology**	**36**
4. Undesirable (or persistence) traits	*12*
5. Resource exploitation	*2*
6. Reproduction	*7*
7. Dispersal mechanisms	*9*
8. Tolerance attributes	*6*
**C. Climate change**	**6**
9. Climate change	*6*
**Sectors affected**	
**Commercial**	**15**
**Environmental**	**15**
**Species or population nuisance traits**	**37**

**Thresholds**	
**BRA**	**41**
**BRA+CCA**	**41**
**Confidence**	
**BRA+CCA**	**0.85**
**BRA**	**0.85**
**CCA**	**0.83**

**Date and Time**
**31/07/2024 17:21:34**

TPS-ISK v2

Taxon and Assessor details	
Category	Angiosperms
Taxon name	*Betula papyrifera*
Common name	Canoe Birch
Assessor	Dario Hruševar
**Risk screening context**	
Reason and socio-economic benefits	ornamental purposes, birch syrup, ethnobotanical uses
Risk assessment area	Continental Europe
Taxonomy	fam. Betulaceae
Native range	Northern America
Introduced range	Europe, Asia, Australia, NZ
URL	

			Response	Justification (references or other information)	Confidence
**A. Biogeography/Historical**					
1. Domestication/Cultivation					
1	1.01	Has the taxon been the subject of domestication?	Yes	Betula papyrifera Marshall in GBIF Secretariat (2023). GBIF Backbone Taxonomy. Checklist dataset https://doi.org/10.15468/39omei accessed via GBIF.org on 2024-07-22.	Very high
2	1.02	Is the taxon harvested in the wild and likely to be sold or used in its live form?	Yes	No data found (ornamental purposes - professional judgement)	Low
3	1.03	Does the taxon have invasive races, varieties, sub-taxa or congeners?	Yes	https://www.invasive.org/browse/subinfo.cfm?sub=11537	Very high
2. Climate, distribution and introduction risk					
4	2.01	How similar are the climatic conditions between the risk assessment area and the taxon's native range?	Medium	Peel et al. 2007	High
5	2.02	What is the quality of the climate matching data?	Medium	Peel et al. 2007	Medium
6	2.03	Is the taxon known to be present outside of cultivation in the risk assessment area?	Yes	Betula papyrifera Marshall in GBIF Secretariat (2023). GBIF Backbone Taxonomy. Checklist dataset https://doi.org/10.15468/39omei accessed via GBIF.org on 2024-08-22.	High
7	2.04	How many potential vectors could the taxon use to enter the risk assessment area?	>1	https://www.agroforestry.co.uk/product/betula-papyrifera/	High
8	2.05	Is the taxon currently found in close proximity to, and likely to enter, the risk assessment area in the near future (e.g. unintentional or intentional introductions)?	Yes	Betula papyrifera Marshall in GBIF Secretariat (2023). GBIF Backbone Taxonomy. Checklist dataset https://doi.org/10.15468/39omei accessed via GBIF.org on 2024-08-22.	High
3. Invasive elsewhere					
9	3.01	Has the taxon become naturalised outside its native range?	No	CASUAL (Betula papyrifera Marshall in GBIF Secretariat (2023). GBIF Backbone Taxonomy. Checklist dataset https://doi.org/10.15468/39omei accessed via GBIF.org on 2024-08-22.)	High
10	3.02	In the taxon's introduced range, are there any known adverse impacts to wild or commercial species?	No	No data found (professional judgement)	Medium
11	3.03	In the taxon's introduced range, are there any known adverse impacts to agriculture or forestry?	No	No data found (professional judgement)	Medium
12	3.04	In the taxon's introduced range, are there any known adverse impacts to ecosystem	No	No data found (professional judgement)	High
13	3.05	In the taxon's introduced range, are there any known adverse socio-economic impacts?	No	No data found (professional judgement)	High
**B. Biology/Ecology**					
4. Undesirable (or persistence) traits					
14	4.01	Is the taxon likely to be poisonous or pose other risks to human health?	No	https://bplant.org/plant/220	High
15	4.02	Is the taxon likely to suppress the growth of one or more native species?	No	No data found (professional judgement)	Medium
16	4.03	Are there any threatened or protected native species that the taxon would parasitise in the risk assessment area?	No	No data found (professional judgement)	Medium
17	4.04	Is the taxon adaptable in terms of climatic and other environmental conditions, thus enhancing its potential persistence if it has invaded or is likely to invade the risk	Yes	https://bplant.org/plant/220	High
18	4.05	Is the taxon likely to disrupt terrestrial ecosystem function if it has invaded or is likely to invade the risk assessment area?	No	No data found (professional judgement)	Medium
19	4.06	Is the taxon likely to exert adverse impacts on ecosystem services in the risk	No	No data found (professional judgement)	Medium
20	4.07	Is the taxon likely to host or function as a vector for recognised pests and pathogens that are present in the risk assessment area?	No	No data found	Low
21	4.08	Is the taxon likely to host or function as a vector for recognised pests and pathogens that are absent in the risk assessment area?	Yes	https://bplant.org/plant/220	Medium
22	4.09	Is the taxon likely to be released from cultivation?	Yes	Betula papyrifera Marshall in GBIF Secretariat (2023). GBIF Backbone Taxonomy. Checklist dataset https://doi.org/10.15468/39omei accessed via GBIF.org on 2024-08-22.	Medium

23	4.10	Is the taxon versatile in habitat use?	Yes	https://bplant.org/plant/220	High
24	4.11	Is it likely that the taxon's mode of existence or behaviours will reduce habitat quality for native species?	No	No data found (professional judgement)	Medium
25	4.12	Is the taxon likely to maintain a viable population even when present in low densities (or persist in adverse conditions by way of a dormant form)?	No	No data found (professional judgement)	Low
5. Resource exploitation					
26	5.01	Is the taxon likely to put threatened or protected native species under competitive pressure in the risk assessment area?	No	No data found (professional judgement)	Medium
27	5.02	Is the taxon likely to sequester resources to the detriment of native species in the risk assessment area?	No	No data found (professional judgement)	Low
6. Reproduction					
28	6.01	Is the taxon likely to exhibit changes in reproductive strategy in response to environmental conditions?	Yes	Professional judgement; https://bplant.org/plant/220	Medium
29	6.02	Is the taxon likely to produce viable propagules in the risk assessment area?	Yes	https://www.missouribotanicalgarden.org/PlantFinder/PlantFinderDetails.aspx?taxonid=242294	High
30	6.03	Is the taxon likely to hybridise with native species under natural conditions?	No	No data found (professional judgement)	Low
31	6.04	Is the taxon likely to be hermaphroditic or to exhibit asexual reproduction?	No	eg. https://en.wikipedia.org/wiki/Betula_papyrifera	Very high
32	6.05	Is the taxon dependent on the presence of another species (or specific habitat features) to complete its life cycle?	No	eg. https://en.wikipedia.org/wiki/Betula_papyrifera	High
33	6.06	Is the taxon likely to produce a large number of propagules?	Yes	eg. https://en.wikipedia.org/wiki/Betula_papyrifera; https://bplant.org/plant/220	Very high
34	6.07	How many time units (days, months, years) does the taxon require to reach the age at first reproduction?	>10	https://bplant.org/plant/220	Very high
7. Dispersal mechanisms					
35	7.01	How many potential vectors or pathways could the taxon use to disperse within the risk assessment area (with suitable habitats nearby)?	>1	eg. https://en.wikipedia.org/wiki/Betula_papyrifera; https://bplant.org/plant/220	Very high
36	7.02	Will any of these vectors or pathways bring the taxon in close proximity to one or more protected areas?	Yes	eg. https://en.wikipedia.org/wiki/Betula_papyrifera; https://bplant.org/plant/220	High
37	7.03	Does the taxon have a specialised means of attachment such that it enhances the likelihood of dispersal?	Yes	eg. https://en.wikipedia.org/wiki/Betula_papyrifera; https://bplant.org/plant/220	Very high
38	7.04	Is natural dispersal of the taxon likely to occur as seeds or spores in the risk	Yes	eg. https://en.wikipedia.org/wiki/Betula_papyrifera; https://bplant.org/plant/220	Very high
39	7.05	Is natural dispersal of the taxon likely to occur as fragments or seedlings in the risk assessment area?	Yes	eg. https://en.wikipedia.org/wiki/Betula_papyrifera; https://bplant.org/plant/220	High
40	7.06	Are any life stages of the taxon likely to migrate into the risk assessment area for reproduction?	No	No data found (professional judgement)	Medium
41	7.07	Are propagules of the taxon likely to be dispersed in the risk assessment area by other species?	No	No data found (professional judgement)	High
42	7.08	Is dispersal of the taxon along any of the vectors or pathways mentioned in the previous seven Questions (35–41: i.e. either unintentional or intentional) likely to be	No	No data found (professional judgement)	Medium
43	7.09	Is dispersal of the taxon density dependent?	No	No data found (professional judgement)	Medium
8. Tolerance attributes					
44	8.01	Is the taxon able to withstand being in water for extended periods at some stage of its life cycle?	No	No data found (professional judgement)	Medium
45	8.02	Is the taxon tolerant of a wide range of soil or air quality conditions?	Yes	https://en.wikipedia.org/wiki/Betula_papyrifera; https://bplant.org/plant/220	High
46	8.03	Can the taxon be controlled or eradicated in the wild with chemical, biological or other agents and means?	Yes	Betula pendula (https://www.invasive.org/weedcd/pdfs/wow/european-white-birch.pdf)	Very high
47	8.04	Is the taxon likely to benefit from environmental or human disturbance?	No	Professional judgement	Medium
48	8.05	Is the taxon able to tolerate soil acidity, salinity or other parameter levels that are higher or lower than those found in its usual	Yes	https://en.wikipedia.org/wiki/Betula_papyrifera; https://bplant.org/plant/220	Very high
49	8.06	Are there effective natural enemies of the taxon present in the risk assessment area?	No	Professional judgement	Low
**C. Climate change**					
9. Climate change					
50	9.01	Under the predicted future climatic conditions, are the risks of entry into the risk assessment area posed by the taxon likely to increase, decrease or not change?	No change	Professional judgement	Medium

51	9.02	Under the predicted future climatic conditions, are the risks of establishment within the risk assessment area posed by the taxon likely to increase, decrease or not	No change	Professional judgement	Medium
52	9.03	Under the predicted future climatic conditions, are the risks of dispersal within the risk assessment area posed by the taxon likely to increase, decrease or not change?	No change	Professional judgement	Medium
53	9.04	Under the predicted future climatic conditions, what is the likely magnitude of future potential impacts by the taxon on biodiversity or ecosystem integrity and	No change	Professional judgement	Medium
54	9.05	Under the predicted future climatic conditions, what is the likely magnitude of future potential impacts by the taxon on ecosystem structure or function?	No change	Professional judgement	Medium
55	9.06	Under the predicted future climatic conditions, what is the likely magnitude of future potential impacts by the taxon on ecosystem services and related socio-	No change	Professional judgement	Medium

**Statistics**	
**Scores**	
**BRA**	**15.0**
**BRA Outcome**	**Medium**
**BRA+CCA**	**15.0**
**BRA+CCA Outcome**	**Medium**
**Score partition**	
**A. Biogeography/Historical**	**6.0**
1. Domestication/Cultivation	*4.0*
2. Climate, distribution and introduction risk	*4.0*
3. Invasive elsewhere	*-2.0*
**B. Biology/Ecology**	**9.0**
4. Undesirable (or persistence) traits	*4.0*
5. Resource exploitation	*0.0*
6. Reproduction	*1.0*
7. Dispersal mechanisms	*3.0*
8. Tolerance attributes	*1.0*
**C. Climate change**	**0.0**
9. Climate change	*0.0*
**Answered Questions**	
Total	***55***
**A. Biogeography/Historical**	**13**
1. Domestication/Cultivation	*3*
2. Climate, distribution and introduction risk	*5*
3. Invasive elsewhere	*5*
**B. Biology/Ecology**	**36**
4. Undesirable (or persistence) traits	*12*
5. Resource exploitation	*2*
6. Reproduction	*7*
7. Dispersal mechanisms	*9*
8. Tolerance attributes	*6*
**C. Climate change**	**6**
9. Climate change	*6*
**Sectors affected**	
**Commercial**	**8**
**Environmental**	**1**
**Species or population nuisance traits**	**12**

**Thresholds**	
**BRA**	**41**
**BRA+CCA**	**41**
**Confidence**	
**BRA+CCA**	**0.64**
**BRA**	**0.65**
**CCA**	**0.50**

**Date and Time**
**12/08/2024 22:21:49**

TPS-ISK v2

Taxon and Assessor details	
Category	Angiosperms
Taxon name	*Catalpa bignonioides*
Common name	Southern Catalpa
Assessor	Sara Essert
**Risk screening context**	
Reason and socio-economic benefits	
Risk assessment area	Continental Europe
Taxonomy	
Native range	
Introduced range	
URL	

			Response	Justification (references or other information)	Confidence
**A. Biogeography/Historical**					
1. Domestication/Cultivation					
1	1.01	Has the taxon been the subject of domestication?	Yes	"By the early nineteenth Century, catalpa was cultivated throughout Europe, as demand for this beautiful flowering and undemanding exotic from America increased (Duhamel du Monceau, 1755, Cavanilles, 1801, Loudon, 1838). " - https://link.springer.com/article/10.1007/s12228-017-9471-7, "First Introduction in Europe RU, 1866 DAISIE-Delivering Alien Invasive Species Inventories for Europe." - https://alien.jrc.ec.europa.eu/spexplorer/species/factsheet/R02925 , The tree bloomes around three and five years of age - https://dengarden.com/gardening/Catalpa-Tree-Facts-and-Details	Very high
2	1.02	Is the taxon harvested in the wild and likely to be sold or used in its live form?	Yes	The species is sold and used for horticultural purposes.	Very high
3	1.03	Does the taxon have invasive races, varieties, sub-taxa or congeners?	No	As far as I managed to research the literature and the GBFI database - I did not find any information about invasive relatives.	Medium
2. Climate, distribution and introduction risk					
4	2.01	How similar are the climatic conditions between the risk assessment area and the taxon's native range?	High	"...is native to the southeastern United States in Alabama, Florida, Georgia, Louisiana, and Mississippi. " - Cfa climate https://en.wikipedia.org/wiki/Catalpa_bignonioides#Cultivation_and_uses The climate Cfa is also present in Europe (Peel et al 2007).	Very high
5	2.02	What is the quality of the climate matching data?	High	I used a scientifc paper (Peel et al 2007), so I would say that is high quality of data.	High
6	2.03	Is the taxon known to be present outside of cultivation in the risk assessment area?	Yes	"Records of self-sown plants are steadily increasing, not only in Belgium but in most of the surrounding countries as well, see for instance: Schmid (2005), Brandes (2011), Smettan (2011), etc." - https://alienplantsbelgium.myspecies.info/content/catalpa	Very high
7	2.04	How many potential vectors could the taxon use to enter the risk assessment area?	>1	It is already in RA, but it could and is already coming (still): 1) intentional intake for horticulture, 2) seed propagation	Very high
8	2.05	Is the taxon currently found in close proximity to, and likely to enter, the risk assessment area in the near future (e.g. unintentional or intentional introductions)?	Not applicable	The taxon is already present in RA - https://www.gbif.org/species/3172615	Very high
3. Invasive elsewhere					
9	3.01	Has the taxon become naturalised outside its native range?	No	I didn't find any information about it. "Union concern: No Native: No Impact: No" - https://alien.jrc.ec.europa.eu/spexplorer/species/factsheet/R02925	Medium
10	3.02	In the taxon's introduced range, are there any known adverse impacts to wild or commercial species?	No	Since naturalization does not seem to have happened yet, I think it has no impact.	High
11	3.03	In the taxon's introduced range, are there any known adverse impacts to agriculture or forestry?	No	Since naturalization does not seem to have happened yet, I think it has no impact.	High
12	3.04	In the taxon's introduced range, are there any known adverse impacts to ecosystem	No	Since naturalization does not seem to have happened yet, I think it has no impact.	High
13	3.05	In the taxon's introduced range, are there any known adverse socio-economic impacts?	No	Since naturalization does not seem to have happened yet, I think it has no impact.	High
**B. Biology/Ecology**					
4. Undesirable (or persistence) traits					
14	4.01	Is the taxon likely to be poisonous or pose other risks to human health?	No	"Catalpa has no toxic effects reported." - https://www.gardenersworld.com/plants/catalpa-bignonioides/ The root is poisonous, but I imagine the chances of a human eating the root are slim. Or should the answer be changed to "Yes"? - "The roots of this species are highly poisonous (Chevallier 1996)." - USDA NRCS Plant guide, 2010 (Species Coordinator: John M. Row, USDA NRCS Manhattan Plant Materials Center,	Medium
15	4.02	Is the taxon likely to suppress the growth of one or more native species?	No	Since naturalization does not seem to have happened yet, I think currently the chance is minimal.	Medium
16	4.03	Are there any threatened or protected native species that the taxon would parasitise in the risk assessment area?	No	The taxon is not a parasite.	Very high
17	4.04	Is the taxon adaptable in terms of climatic and other environmental conditions, thus enhancing its potential persistence if it has invaded or is likely to invade the risk assessment area?	Yes	I haven't found a sentence in the literature that claims that the taxon is an extremely climatically or environmentally adaptable species, but considering that the species grows successfully all over Europe, I would still say that it is adaptable. (Although, there are certainly plants that are much more adaptable.)	Low
18	4.05	Is the taxon likely to disrupt terrestrial ecosystem function if it has invaded or is likely to invade the risk assessment area?	No	I have not found any data that the taxon has a negative impact on terrestrial ecosystem function, and it does not seem to me that this could happen in the foreseeable future.	Medium

19	4.06	Is the taxon likely to exert adverse impacts on ecosystem services in the risk assessment area?	No	I have not found any data that the taxon or its relatives would have a negative impact on ecosystem services, and it does not seem to me that this could happen in the foreseeable future.	Medium
20	4.07	Is the taxon likely to host or function as a vector for recognised pests and pathogens that are present in the risk assessment area?	Yes	Although I really searched a lot, I didn't find specific scientific papers (or even horticulture articles) about pests on Catalpa in Europe. In the end, I'm pasting what AI (Chat GPT) wrote about autochthonous pests on the species: 1) Aphidoidea 2) Moth caterpillars - Various species from the order Lepidoptera 3) Big tree orderer - Cossus cossus 4) Cherry fly - Rhagoletis cerasi 5) Nematodes - Various species from the class Nematoda	Medium
21	4.08	Is the taxon likely to host or function as a vector for recognised pests and pathogens that are absent in the risk assessment area?	Yes	In North America, the species hosts is the pest Ceratomia catalpae, which has not yet arrived in Europe, but that possibility always exists. As Catalpa bignoides is widespread throughout RA, there is a high possibility that Ceratomia catalpae once it enters the RA will spread rapidly (primarily) through horticulture and planted coridors of the plants.	High
22	4.09	Is the taxon likely to be released from cultivation?	Yes	The taxon is already released from cultivation. "Records of self-sown plants are steadily increasing, not only in Belgium but in most of the surrounding countries as well, see for instance: Schmid (2005), Brandes (2011), Smettan (2011), etc." - https://alienplantsbelgium.myspecies.info/content/catalpa	Very high
23	4.10	Is the taxon versatile in habitat use?	No	I did not find data that the species is particularly adaptable to large ranges of ecological parameters.	Medium
24	4.11	Is it likely that the taxon's mode of existence or behaviours will reduce habitat quality for native species?	No	The species is not poisonous or unpleasant for animals. "Catalpa has no toxic effects reported." - https://www.gardenersworld.com/plants/catalpa-bignonioides/	Very high
25	4.12	Is the taxon likely to maintain a viable population even when present in low densities (or persist in adverse conditions by way of a dormant form)?	Yes	I haven't found data on this, but I believe that established populations can be maintained even at low densities.	Low
5. Resource exploitation					
26	5.01	Is the taxon likely to put threatened or protected native species under competitive pressure in the risk assessment area?	No	For now, it does not seem that the species is a threat to the autochthonous flora, nor to endangered species.	Medium
27	5.02	Is the taxon likely to sequester resources to the detriment of native species in the risk assessment area?	Yes	If the species becomes naturalized, then of course the species will exploit the resources in the habitat (light, space...)	Low
6. Reproduction					
28	6.01	Is the taxon likely to exhibit changes in reproductive strategy in response to environmental conditions?	No	I have not found data that the species can show a more effective reproductive strategy in case of changes in environmental conditions.	Medium
29	6.02	Is the taxon likely to produce viable propagules in the risk assessment area?	Yes	"Most taxa seem to produce viable seed and, under undisturbed circumstances," - https://alienplantsbelgium.myspecies.info/content/catalpa#	Very high
30	6.03	Is the taxon likely to hybridise with native species under natural conditions?	No	I found no data about hibridisation with native secies. And since the genus Catalpa is not native to Europe I would say that there is no risk of hybridisation.	High
31	6.04	Is the taxon likely to be hermaphroditic or to exhibit asexual reproduction?	Yes	Almost all plants have the possibility of asexual reproduction. "Root cuttings may also be used to propagate trees." - USDA NRCS chrome-extension://efaidnbmnnnibpcajpcglclefindmkaj/https://www.nrcs.usda.gov/plantmaterials/kspmcpg9634.pdf	Very high
32	6.05	Is the taxon dependent on the presence of another species (or specific habitat features) to complete its life cycle?	No	I have not found any information that such a thing would be necessary to complete the life cycle.	High
33	6.06	Is the taxon likely to produce a large number of propagules?	Yes	"There are approximately 20,480 seeds per pound" - USDA NRCS chrome-extension://efaidnbmnnnibpcajpcglclefindmkaj/https://www.nrcs.usda.gov/plantmaterials/kspmcpg9634.pdf Compared to other woody species, Catalpa has a lot of seeds (but not as many as species that have even smaller and extremely numerous seeds). I'm not sure exactly how a large number of seeds is defined, so I	Low
34	6.07	How many time units (days, months, years) does the taxon require to reach the age at first reproduction?	5	"Most catalpas begin flowering after roughly three years, and produce fruit after about five years." - https://en.wikipedia.org/wiki/Catalpa I also found data that "good seed age by the age 10" (chrome-extension://efaidnbmnnnibpcajpcglclefindmkaj/https://warnell.uga.edu/sites/default/files/inline-files/Catalpabig%20-%20ITS%20-%20Southern%20Catalpa%20-%20The%20Fish%20Bait%20Tree%20ARBOR-17.pdf), and that is the reason why I put Medium confidence level	Medium
7. Dispersal mechanisms					
35	7.01	How many potential vectors or pathways could the taxon use to disperse within the risk assessment area (with suitable habitats nearby)?	>1	1) By deliberate planting for horticulture - the main way of spreading throughout Europe 2) By seeds - the species produces viable seeds and propagation is easily possible if the conditions are favorable 3) By root cuttings - it is theoretically possible to spread the species by accident in such a way that the soil where the species grew and where parts of the roots are found is	Very high
36	7.02	Will any of these vectors or pathways bring the taxon in close proximity to one or more protected areas?	Yes	The species is planted a lot (parks, gardens...), so these are all corridors that bring it closer to protected areas.	High
37	7.03	Does the taxon have a specialised means of attachment such that it enhances the likelihood of dispersal?	No	The fruit and seeds of the species do not have hooks.	Very high

38	7.04	Is natural dispersal of the taxon likely to occur as seeds or spores in the risk assessment area?	Yes	The seeds are spread by the wind and are built so that the wind can spread them easily and far (they are light and have wings). "Seeds are about 1 inch long and 1/3 inch wide. They have a light brown coat and wings rounded at the ends terminating in a fringe of short hairs." "In late summer or autumn the fruit appear; they are siliques about 20–50 centimetres (8–20 in) long, full of small flat seeds, each with two thin wings to aid in wind dispersal." - https://en.wikipedia.org/wiki/Catalpa	Very high
39	7.05	Is natural dispersal of the taxon likely to occur as fragments or seedlings in the risk assessment area?	No	Although this possibility is not excluded, I would say that it is not very likely. The species is most often planted in parks and gardens, and even when it is uprooted and root fragments appear (which could be a way of spreading the species), I would say that there is little probability that the soil from the gardens and parks will be transported somewhere.	Medium
40	7.06	Are any life stages of the taxon likely to migrate into the risk assessment area for reproduction?	Yes	"Records of self-sown plants are steadily increasing, not only in Belgium but in most of the surrounding countries as well, see for instance: Schmid (2005), Brandes (2011), Smettan (2011), etc." - https://alienplantsbelgium.myspecies.info/content/catalpa# In Germany, for the urban area of Stuttgart, it is written that the species Catalpa bignoides belongs to the category cited below, which is proof that the species can successfully spread naturally. "Species in the process of naturalising and species locally naturalised in the urban area Species that are on the threshold of naturalisation in one or more places (i.e. have shown naturalisation tendencies in the last 10 to 20 years, but do not yet show the criteria of a fully naturalised species – the plants of the 2nd generation have not yet reached the age of reproduction) or have already exceeded this locally (in one or a few places). In most cases, the starting point of the wild growth is still traceable, the wild-growing stocks are still in the immediate vicinity of the Environment of the (planted) specimens from which they originate:" - Wörz a. " Vorwort des Schriftleiters zur Publikation „Untersuchung zur neophytischen Gehölzflora im Stuttgarter Stadtgebiet“, Jh. Ges. Naturkde. Württemberg 161 S. 178–257, https://ojs-jh-gfn.ub.uni-	Very high
41	7.07	Are propagules of the taxon likely to be dispersed in the risk assessment area by other species?	No	The seeds are spread by the wind and other species do not participate in the spread.	High
42	7.08	Is dispersal of the taxon along any of the vectors or pathways mentioned in the previous seven Questions (35–41: i.e. either unintentional or intentional) likely to be	Yes	Considering that the man/horticulture is the main vector of spread in RA, I would say that rapid spread is present and very likely in the future as well.	Very high
43	7.09	Is dispersal of the taxon density dependent?	Yes	I believe that an increase in population density could positively influence seed dispersal, as a larger number of seeds would increase the likelihood of successful dispersal. However, since I couldn't find any studies addressing this topic, my confidence in this assertion is low.	Low
8. Tolerance attributes					
44	8.01	Is the taxon able to withstand being in water for extended periods at some stage of its life cycle?	No	I have not found information that the species can survive in water at any stage.	Medium
45	8.02	Is the taxon tolerant of a wide range of soil or air quality conditions?	Yes	The species is often and successfully planted in cities, so it obviously tolerates air and soil pollution well. However, I did not find specific research on that topic, which would confirm it, so I put the Medium level of reliability. "Important tree in urban areas for its high resistance to air pollution." - https://theoriginalgarden.com/p/plants/outdoor/trees/catalpa-bignonioides-indian-bean-tree The fruit quality is worse in contaminated enviroments, but the trees stay alive, so I would not take that as an evidence for bad poor tolerance to pollution "The fruiting quality of C. bignonioides plantsbecame worse under environmental contamination by emissions of pipe factory and motor transport. " -	Medium
46	8.03	Can the taxon be controlled or eradicated in the wild with chemical, biological or other agents and means?	Yes	chrome-extension://efaidnbmnnnibpcajpcglclefindmkaj/https://wric.ucdavis.edu/information/natural%20areas/wr_C/Catalpa.pdf - These are data for USA, but I believe that the means and methods would work equally successfully in RA.	High
47	8.04	Is the taxon likely to benefit from environmental or human disturbance?	Yes	The species shows a pioneer character and inhabits degraded areas, so it could happen that it will spread if there are more such areas in the future. "Die spontanen Vorkommen in Mitteleuropa zeigen den Pioniercharakter der Art, die bisher ausschließlich an Ruderalstandorten aufzutreten scheint." = "The spontaneous occurrences in Central Europe demonstrate the pioneering character of the species, which so far seems to occur exclusively in ruderal sites." - Wörz a. " Vorwort des Schriftleiters zur Publikation „Untersuchung zur neophytischen Gehölzflora im Stuttgarter Stadtgebiet“, Jh. Ges. Naturkde. Württemberg 161 S. 178–257, https://ojs-jh-gfn.ub.uni-stuttgart.de/index.php/jahreshefte/article/view/280/382	High
48	8.05	Is the taxon able to tolerate soil acidity, salinity or other parameter levels that are higher or lower than those found in its usual	No	I did not find data that it can tolerate a wider range of these parameters.	Medium

49	8.06	Are there effective natural enemies of the taxon present in the risk assessment area?	No	The most significant pest is Ceratomia catalpae, but it did not yet reach Europe. "Larvae of the catalpa sphinx moth (Ceratomia catalpae) eat the trees leaves. Almost complete defoliation may occur in some years." The invasive alien fungus Erysiphe elevata is present and researched in Romania (Chinan & Manzu (2018) Distribution, Incidence and Severity of the Catalpa Powdery Mildew Caused by Erysiphe elevata in North-Eastern Romania. Not Sci Biol, 2018, 10(4):614-617. DOI: 10.25835/AcademicPres nsb10410374, but I would not call this an effective enemy.	Medium
**C. Climate change**					
9. Climate change					
50	9.01	Under the predicted future climatic conditions, are the risks of entry into the risk assessment area posed by the taxon likely to increase, decrease or not change?	Not applicable	The species is already present in RA and grows in horticulture in various climates.	Very high
51	9.02	Under the predicted future climatic conditions, are the risks of establishment within the risk assessment area posed by the taxon likely to increase, decrease or not change?	No change	The species in RA grows (in horticulture) in various climates and is currently not naturalized anywhere. I would not say that the climate is the decisive factor why the species did not escape from cultivation, so I do not believe that climate change will significantly affect the spread of the species. I didn't find research on that topic, so I put the confidence level "Low".	Low
52	9.03	Under the predicted future climatic conditions, are the risks of dispersal within the risk assessment area posed by the taxon likely to increase, decrease or not change?	No change	The species is already present in RA and grows in horticulture in various climates. I would not say that the climate is the decisive factor for species distributiion, so I do not believe that climate change will significantly affect the spread of the species. I didn't find research on that topic, so I put the confidence level "Low"	Low
53	9.04	Under the predicted future climatic conditions, what is the likely magnitude of future potential impacts by the taxon on biodiversity or ecosystem integrity and status?	No change	I would not say that the climate is the decisive factor for species distribution, so I do not believe that climate change will significantly affect the spread of the species and therefore it will also not affect biodiversity. I didn't find research on that topic, so I put the confidence level "Low"	Low
54	9.05	Under the predicted future climatic conditions, what is the likely magnitude of future potential impacts by the taxon on ecosystem structure or function?	No change	I would not say that the climate is the decisive factor for species distribution, so I do not believe that climate change will significantly affect the spread of the species and therefore it will also not affect structure of ecosystem. I didn't find research on that topic, so I put the confidence level "Low".	Low
55	9.06	Under the predicted future climatic conditions, what is the likely magnitude of future potential impacts by the taxon on ecosystem services and related socio-economic factors?	No change	I would not say that the climate is the decisive factor for species distribution, so I do not believe that climate change will significantly affect the spread of the species and therefore it will not ecosystem services. I didn't find research on that topic, so I put the confidence level "Low"	Low

**Statistics**	
**Scores**	
**BRA**	**18.0**
**BRA Outcome**	**Medium**
**BRA+CCA**	**18.0**
**BRA+CCA Outcome**	**Medium**
**Score partition**	
**A. Biogeography/Historical**	**4.0**
1. Domestication/Cultivation	*2.0*
2. Climate, distribution and introduction risk	*2.0*
3. Invasive elsewhere	*0.0*
**B. Biology/Ecology**	**14.0**
4. Undesirable (or persistence) traits	*5.0*
5. Resource exploitation	*2.0*
6. Reproduction	*2.0*
7. Dispersal mechanisms	*3.0*
8. Tolerance attributes	*2.0*
**C. Climate change**	**0.0**
9. Climate change	*0.0*
**Answered Questions**	
Total	***55***
**A. Biogeography/Historical**	**13**
1. Domestication/Cultivation	*3*
2. Climate, distribution and introduction risk	*5*
3. Invasive elsewhere	*5*
**B. Biology/Ecology**	**36**
4. Undesirable (or persistence) traits	*12*
5. Resource exploitation	*2*
6. Reproduction	*7*
7. Dispersal mechanisms	*9*
8. Tolerance attributes	*6*
**C. Climate change**	**6**
9. Climate change	*6*
**Sectors affected**	
**Commercial**	**7**
**Environmental**	**1**
**Species or population nuisance traits**	**15**

**Thresholds**	
**BRA**	**41**
**BRA+CCA**	**41**
**Confidence**	
**BRA+CCA**	**0.66**

BRA	0.70
CCA	0.38

Date and Time
09/08/2024 15:36:34

TPS-ISK v2

**Taxon and Assessor details**	
Category	Angiosperms
Taxon name	*Celtis occidentalis*
Common name	American hackberry (Common hackberry)
Assessor	Dario Hruševar
**Risk screening context**	
Reason and socio-economic benefits	ornamental purposes
Risk assessment area	Continental Europe
Taxonomy	fam. Cannabaceae
Native range	North America
Introduced range	all other continents
URL	

			Response	Justification (references or other information)	Confidence
**A. Biogeography/Historical**					
1. Domestication/Cultivation					
1	1.01	Has the taxon been the subject of domestication?	Yes	DAISIE - Inventory of alien invasive species in Europe https://doi.org/10.15468/ybwd3x	High
2	1.02	Is the taxon harvested in the wild and likely to be sold or used in its live form?	Yes	https://www.missouribotanicalgarden.org/PlantFinder/PlantFinderDetails.aspx?kempercode=a858	High
3	1.03	Does the taxon have invasive races, varieties, sub-taxa or congeners?	Yes	Celtis sinensis (eg. https://www.dpi.nsw.gov.au/__data/assets/pdf_file/0006/319731/Chinese-celtis-identification-and-control.pdf)	Very high
2. Climate, distribution and introduction risk					
4	2.01	How similar are the climatic conditions between the risk assessment area and the taxon's native range?	High	Peel et al. 2007	High
5	2.02	What is the quality of the climate matching data?	Medium	Peel et al. 2007	Medium
6	2.03	Is the taxon known to be present outside of cultivation in the risk assessment area?	Yes	Celtis occidentalis L. in GBIF Secretariat (2023). GBIF Backbone Taxonomy. Checklist dataset https://doi.org/10.15468/39omei accessed via GBIF.org on 2024-07-10.; https://en.wikipedia.org/wiki/Celtis_occidentalis	Very high
7	2.04	How many potential vectors could the taxon use to enter the risk assessment area?	>1	FloraVeg.EU – Database of European Vegetation, Habitats and Flora. www.floraveg.eu	High
8	2.05	Is the taxon currently found in close proximity to, and likely to enter, the risk assessment area in the near future (e.g. unintentional or intentional introductions)?	Yes	Celtis occidentalis L. in GBIF Secretariat (2023). GBIF Backbone Taxonomy. Checklist dataset https://doi.org/10.15468/39omei accessed via GBIF.org on 2024-07-10.	High
3. Invasive elsewhere					
9	3.01	Has the taxon become naturalised outside its native range?	No	Casual (eg. https://plantnet.rbgsyd.nsw.gov.au/cgi-bin/NSWfl.pl?page=nswfl&lvl=sp&name=Celtis~occidentalis)	Medium
10	3.02	In the taxon's introduced range, are there any known adverse impacts to wild or commercial species?	No	No data found (professional judgement)	Low
11	3.03	In the taxon's introduced range, are there any known adverse impacts to agriculture or forestry?	No	No data found (professional judgement)	Low
12	3.04	In the taxon's introduced range, are there any known adverse impacts to ecosystem	Yes	No data found (professional judgement)	Low
13	3.05	In the taxon's introduced range, are there any known adverse socio-economic impacts?	No	No data found (professional judgement)	Low
**B. Biology/Ecology**					
4. Undesirable (or persistence) traits					
14	4.01	Is the taxon likely to be poisonous or pose other risks to human health?	No	No data found (professional judgement); data for ethnobotanical uses (https://en.wikipedia.org/wiki/Celtis_occidentalis).	Low
15	4.02	Is the taxon likely to suppress the growth of one or more native species?	No	No data found (professional judgement)	Low
16	4.03	Are there any threatened or protected native species that the taxon would parasitise in the risk assessment area?	No	No data found (professional judgement)	Medium
17	4.04	Is the taxon adaptable in terms of climatic and other environmental conditions, thus enhancing its potential persistence if it has invaded or is likely to invade the risk	Yes	https://en.wikipedia.org/wiki/Celtis_occidentalis; https://www.missouribotanicalgarden.org/PlantFinder/PlantFinderDetails.aspx?kempercode=a858; FloraVeg.EU – Database of European Vegetation, Habitats and Flora. www.floraveg.eu	High
18	4.05	Is the taxon likely to disrupt terrestrial ecosystem function if it has invaded or is likely to invade the risk assessment area?	No	No data found (professional judgement)	Low
19	4.06	Is the taxon likely to exert adverse impacts on ecosystem services in the risk	No	No data found (professional judgement)	Low
20	4.07	Is the taxon likely to host or function as a vector for recognised pests and pathogens that are present in the risk assessment area?	No	No data found (professional judgement)	Low
21	4.08	Is the taxon likely to host or function as a vector for recognised pests and pathogens that are absent in the risk assessment area?	Yes	https://www.missouribotanicalgarden.org/PlantFinder/PlantFinderDetails.aspx?kempercode=a858; https://en.wikipedia.org/wiki/Celtis_occidentalis	High
22	4.09	Is the taxon likely to be released from cultivation?	Yes	https://en.wikipedia.org/wiki/Celtis_occidentalis; https://plantnet.rbgsyd.nsw.gov.au/cgi-bin/NSWfl.pl?page=nswfl&lvl=sp&name=Celtis~occidentalis	High

23	4.10	Is the taxon versatile in habitat use?	Yes	https://en.wikipedia.org/wiki/Celtis_occidentalis; FloraVeg.EU – Database of European Vegetation, Habitats and Flora. www.floraveg.eu; https://www.missouribotanicalgarden.org/PlantFinder/PlantFinderDetails.aspx?kempercode=a858	Very high
24	4.11	Is it likely that the taxon's mode of existence or behaviours will reduce habitat quality for native species?	No	professional judgement (based on eg. https://en.wikipedia.org/wiki/Celtis_occidentalis)	High
25	4.12	Is the taxon likely to maintain a viable population even when present in low densities (or persist in adverse conditions by way of a dormant form)?	No	No data found (professional judgement)	Low
5. Resource exploitation					
26	5.01	Is the taxon likely to put threatened or protected native species under competitive pressure in the risk assessment area?	No	Professional judgement (base on eg. Celtis occidentalis L. in GBIF Secretariat (2023). GBIF Backbone Taxonomy. Checklist dataset https://doi.org/10.15468/39omei accessed via GBIF.org on 2024-07-10.)	High
27	5.02	Is the taxon likely to sequester resources to the detriment of native species in the risk assessment area?	No	No data found	Low
6. Reproduction					
28	6.01	Is the taxon likely to exhibit changes in reproductive strategy in response to environmental conditions?	Yes	FloraVeg.EU – Database of European Vegetation, Habitats and Flora. www.floraveg.eu; https://en.wikipedia.org/wiki/Celtis_occidentalis	High
29	6.02	Is the taxon likely to produce viable propagules in the risk assessment area?	Yes	Professional judgement (based on FloraVeg.EU – Database of European Vegetation, Habitats and Flora. www.floraveg.eu and https://en.wikipedia.org/wiki/Celtis_occidentalis)	Medium
30	6.03	Is the taxon likely to hybridise with native species under natural conditions?	No	No data found. However, possibility is noticed in https://srs.fs.usda.gov/pubs/misc/ag_654/volume_2/celtis/occidentalis.htm	Low
31	6.04	Is the taxon likely to be hermaphroditic or to exhibit asexual reproduction?	Yes	https://en.wikipedia.org/wiki/Celtis_occidentalis; https://srs.fs.usda.gov/pubs/misc/ag_654/volume_2/celtis/occidentalis.htm	Very high
32	6.05	Is the taxon dependent on the presence of another species (or specific habitat features) to complete its life cycle?	No	No data found (professional judgement)	Medium
33	6.06	Is the taxon likely to produce a large number of propagules?	Yes	https://www.missouribotanicalgarden.org/PlantFinder/PlantFinderDetails.aspx?kempercode=a858; https://en.wikipedia.org/wiki/Celtis_occidentalis; https://srs.fs.usda.gov/pubs/misc/ag_654/volume_2/celtis/occidentalis.htm	Very high
34	6.07	How many time units (days, months, years) does the taxon require to reach the age at first reproduction?	>10	eg. https://srs.fs.usda.gov/pubs/misc/ag_654/volume_2/celtis/occidentalis.htm	Very high
7. Dispersal mechanisms					
35	7.01	How many potential vectors or pathways could the taxon use to disperse within the risk assessment area (with suitable habitats nearby)?	>1	birds, humans ... (eg. https://srs.fs.usda.gov/pubs/misc/ag_654/volume_2/celtis/occidentalis.htm)	Very high
36	7.02	Will any of these vectors or pathways bring the taxon in close proximity to one or more protected areas?	Yes	https://srs.fs.usda.gov/pubs/misc/ag_654/volume_2/celtis/occidentalis.htm	Medium
37	7.03	Does the taxon have a specialised means of attachment such that it enhances the likelihood of dispersal?	No	https://srs.fs.usda.gov/pubs/misc/ag_654/volume_2/celtis/occidentalis.htm; https://en.wikipedia.org/wiki/Celtis_occidentalis etc.	Very high
38	7.04	Is natural dispersal of the taxon likely to occur as seeds or spores in the risk assessment area?	Yes	https://srs.fs.usda.gov/pubs/misc/ag_654/volume_2/celtis/occidentalis.htm; https://en.wikipedia.org/wiki/Celtis_occidentalis; FloraVeg.EU – Database of European Vegetation, Habitats and Flora. www.floraveg.eu	High
39	7.05	Is natural dispersal of the taxon likely to occur as fragments or seedlings in the risk assessment area?	Yes	https://srs.fs.usda.gov/pubs/misc/ag_654/volume_2/celtis/occidentalis.htm; https://en.wikipedia.org/wiki/Celtis_occidentalis; FloraVeg.EU – Database of European Vegetation, Habitats and Flora. www.floraveg.eu	High
40	7.06	Are any life stages of the taxon likely to migrate into the risk assessment area for reproduction?	Yes	https://srs.fs.usda.gov/pubs/misc/ag_654/volume_2/celtis/occidentalis.htm; https://en.wikipedia.org/wiki/Celtis_occidentalis; FloraVeg.EU – Database of European Vegetation, Habitats and Flora. www.floraveg.eu	High
41	7.07	Are propagules of the taxon likely to be dispersed in the risk assessment area by other species?	Yes	By birds (https://srs.fs.usda.gov/pubs/misc/ag_654/volume_2/celtis/occidentalis.htm; https://en.wikipedia.org/wiki/Celtis_occidentalis; FloraVeg.EU – Database of European Vegetation, Habitats and Flora. www.floraveg.eu)	High
42	7.08	Is dispersal of the taxon along any of the vectors or pathways mentioned in the previous seven Questions (35–41: i.e. either unintentional or intentional) likely to be rapid?	No	Professional judgement (based on eg. https://srs.fs.usda.gov/pubs/misc/ag_654/volume_2/celtis/occidentalis.htm; https://en.wikipedia.org/wiki/Celtis_occidentalis; FloraVeg.EU – Database of European Vegetation, Habitats and Flora. www.floraveg.eu etc.)	Medium
43	7.09	Is dispersal of the taxon density dependent?	No	No data found	Medium
8. Tolerance attributes					
44	8.01	Is the taxon able to withstand being in water for extended periods at some stage of its life cycle?	Yes	https://srs.fs.usda.gov/pubs/misc/ag_654/volume_2/celtis/occidentalis.htm	Very high
45	8.02	Is the taxon tolerant of a wide range of soil or air quality conditions?	Yes	https://srs.fs.usda.gov/pubs/misc/ag_654/volume_2/celtis/occidentalis.htm	Very high

46	8.03	Can the taxon be controlled or eradicated in the wild with chemical, biological or other agents and means?	Yes	Data for Celstis sinensis (https://www.dpi.nsw.gov.au/__data/assets/pdf_file/0006/319731/Chinese-celtis-identification-and-control.pdf)	Very high
47	8.04	Is the taxon likely to benefit from environmental or human disturbance?	Yes	Profesional judgement (based on eg. https://srs.fs.usda.gov/pubs/misc/ag_654/volume_2/celtis/occidentalis.htm)	Medium
48	8.05	Is the taxon able to tolerate soil acidity, salinity or other parameter levels that are higher or lower than those found in its usual	Yes	https://srs.fs.usda.gov/pubs/misc/ag_654/volume_2/celtis/occidentalis.htm	Very high
49	8.06	Are there effective natural enemies of the taxon present in the risk assessment area?	No	No data found	Low
**C. Climate change**					
9. Climate change					
50	9.01	Under the predicted future climatic conditions, are the risks of entry into the risk assessment area posed by the taxon likely to increase, decrease or not change?	No change	Professional judgement	Medium
51	9.02	Under the predicted future climatic conditions, are the risks of establishment within the risk assessment area posed by the taxon likely to increase, decrease or not	No change	Professional judgement	Medium
52	9.03	Under the predicted future climatic conditions, are the risks of dispersal within the risk assessment area posed by the taxon likely to increase, decrease or not change?	No change	Professional judgement	Medium
53	9.04	Under the predicted future climatic conditions, what is the likely magnitude of future potential impacts by the taxon on biodiversity or ecosystem integrity and	No change	Professional judgement	Medium
54	9.05	Under the predicted future climatic conditions, what is the likely magnitude of future potential impacts by the taxon on ecosystem structure or function?	No change	Professional judgement	Medium
55	9.06	Under the predicted future climatic conditions, what is the likely magnitude of future potential impacts by the taxon on ecosystem services and related socio-	No change	Professional judgement	Medium

**Statistics**	
**Scores**	
**BRA**	**29.0**
**BRA Outcome**	**Medium**
**BRA+CCA**	**29.0**
**BRA+CCA Outcome**	**Medium**
**Score partition**	
**A. Biogeography/Historical**	**10.0**
1. Domestication/Cultivation	*4.0*
2. Climate, distribution and introduction risk	*4.0*
3. Invasive elsewhere	*2.0*
**B. Biology/Ecology**	**19.0**
4. Undesirable (or persistence) traits	*4.0*
5. Resource exploitation	*0.0*
6. Reproduction	*3.0*
7. Dispersal mechanisms	*5.0*
8. Tolerance attributes	*7.0*
**C. Climate change**	**0.0**
9. Climate change	*0.0*
**Answered Questions**	
Total	***55***
**A. Biogeography/Historical**	**13**
1. Domestication/Cultivation	*3*
2. Climate, distribution and introduction risk	*5*
3. Invasive elsewhere	*5*
**B. Biology/Ecology**	**36**
4. Undesirable (or persistence) traits	*12*
5. Resource exploitation	*2*
6. Reproduction	*7*
7. Dispersal mechanisms	*9*
8. Tolerance attributes	*6*
**C. Climate change**	**6**
9. Climate change	*6*
**Sectors affected**	
**Commercial**	**8**
**Environmental**	**5**
**Species or population nuisance traits**	**22**

**Thresholds**	
**BRA**	**41**
**BRA+CCA**	**41**
**Confidence**	
**BRA+CCA**	**0.62**
**BRA**	**0.63**
**CCA**	**0.50**

**Date and Time**

TPS-ISK v2

**Taxon and Assessor details**	
Category	Angiosperms
Taxon name	*Davidia involucrata*
Common name	Dove Tree
Assessor	Božena Mitić
**Risk screening context**	
Reason and socio-economic benefits	landscape tree
Risk assessment area	Continental Europe
Taxonomy	fam. Nyssaceae (Cornaceae according to
Native range	Southwestern China
Introduced range	cultivated elsvere (no data for South Am.)
URL	https://www.gbif.org/species/7322122;

			**Response**	**Justification (references or other information)**	**Confidence**
**A. Biogeography/Historical**					
1. Domestication/Cultivation					
1	1.01	Has the taxon been the subject of domestication?	Yes	https://www.sciencedirect.com/topics/agricultural-and-biological-sciences/davidia-involucrata	High
2	1.02	Is the taxon harvested in the wild and likely to be sold or used in its live form?	Yes	https://www.missouribotanicalgarden.org/plantfinder/PlantFinderDetails.aspx?taxonid=279349	High
3	1.03	Does the taxon have invasive races, varieties, sub-taxa or congeners?	No	No data found	High
2. Climate, distribution and introduction risk					
4	2.01	How similar are the climatic conditions between the risk assessment area and the taxon's native range?	High	Peel et al. 2007	High
5	2.02	What is the quality of the climate matching data?	Medium	Peel et al. 2007	Medium
6	2.03	Is the taxon known to be present outside of cultivation in the risk assessment area?	No	https://alienplantsbelgium.myspecies.info/content/cornaceae	Very high
7	2.04	How many potential vectors could the taxon use to enter the risk assessment area?	One	https://alienplantsbelgium.myspecies.info/content/cornaceae; https://www.missouribotanicalgarden.org/plantfinder/PlantFinderDetails.aspx?taxonid=279349	High
8	2.05	Is the taxon currently found in close proximity to, and likely to enter, the risk assessment area in the near future (e.g. unintentional or intentional introductions)?	No	https://alienplantsbelgium.myspecies.info/content/cornaceae	High
3. Invasive elsewhere					
9	3.01	Has the taxon become naturalised outside its native range?	No	https://alienplantsbelgium.myspecies.info/content/cornaceae; https://www.sciencedirect.com/topics/agricultural-and-biological-sciences/davidia-involucrata	High
10	3.02	In the taxon's introduced range, are there any known adverse impacts to wild or commercial species?	No	Professional judgement	High
11	3.03	In the taxon's introduced range, are there any known adverse impacts to agriculture or forestry?	No	Professional judgement	Medium
12	3.04	In the taxon's introduced range, are there any known adverse impacts to ecosystem	No	Professional judgement	Medium
13	3.05	In the taxon's introduced range, are there any known adverse socio-economic impacts?	No	Professional judgement	Medium
**B. Biology/Ecology**					
4. Undesirable (or persistence) traits					
14	4.01	Is the taxon likely to be poisonous or pose other risks to human health?	Yes	https://plantura.garden/uk/trees-shrubs/handkerchief-tree/handkerchief-tree-overview	High
15	4.02	Is the taxon likely to suppress the growth of one or more native species?	No	Professional judgement	Medium
16	4.03	Are there any threatened or protected native species that the taxon would parasitise in the risk assessment area?	No	Professional judgement	Medium
17	4.04	Is the taxon adaptable in terms of climatic and other environmental conditions, thus enhancing its potential persistence if it has invaded or is likely to invade the risk	No	https://www.missouribotanicalgarden.org/plantfinder/PlantFinderDetails.aspx?taxonid=279349	Medium
18	4.05	Is the taxon likely to disrupt terrestrial ecosystem function if it has invaded or is likely to invade the risk assessment area?	Yes	Professional judgement	Medium
19	4.06	Is the taxon likely to exert adverse impacts on ecosystem services in the risk	No	Professional judgement	Medium
20	4.07	Is the taxon likely to host or function as a vector for recognised pests and pathogens that are present in the risk assessment area?	No	No references found	Low
21	4.08	Is the taxon likely to host or function as a vector for recognised pests and pathogens that are absent in the risk assessment area?	No	No references found	Low
22	4.09	Is the taxon likely to be released from cultivation?	No	No referencs found	Medium
23	4.10	Is the taxon versatile in habitat use?	No	https://www.missouribotanicalgarden.org/plantfinder/PlantFinderDetails.aspx?taxonid=279349	High
24	4.11	Is it likely that the taxon's mode of existence or behaviours will reduce habitat quality for native species?	No	Professional judgement	Medium

25	4.12	Is the taxon likely to maintain a viable population even when present in low densities (or persist in adverse conditions by way of a dormant form)?	No	No references found	Medium
5. Resource exploitation					
26	5.01	Is the taxon likely to put threatened or protected native species under competitive pressure in the risk assessment area?	No	Professional judgement	Medium
27	5.02	Is the taxon likely to sequester resources to the detriment of native species in the risk assessment area?	No	Professional judgement	Medium
6. Reproduction					
28	6.01	Is the taxon likely to exhibit changes in reproductive strategy in response to environmental conditions?	No	Professional judgement, no references found	Medium
29	6.02	Is the taxon likely to produce viable propagules in the risk assessment area?	Yes	https://alienplantsbelgium.myspecies.info/content/cornaceae	High
30	6.03	Is the taxon likely to hybridise with native species under natural conditions?	No	Professional judgement; https://en.wikipedia.org/wiki/Davidia_involucrata	High
31	6.04	Is the taxon likely to be hermaphroditic or to exhibit asexual reproduction?	No	Ames, O. 1931. Davidia involucrata. Bulletin of Popular Information, 5(7): 25–28.	Very high
32	6.05	Is the taxon dependent on the presence of another species (or specific habitat features) to complete its life cycle?	No	Professional judgement; Ames, O. 1931. Davidia involucrata. Bulletin of Popular Information, 5(7): 25–28.	High
33	6.06	Is the taxon likely to produce a large number of propagules?	Yes	https://www.missouribotanicalgarden.org/plantfinder/PlantFinderDetails.aspx?taxonid=279349	High
34	6.07	How many time units (days, months, years) does the taxon require to reach the age at first reproduction?	>10	https://www.missouribotanicalgarden.org/plantfinder/PlantFinderDetails.aspx?taxonid=279349	Very high
7. Dispersal mechanisms					
35	7.01	How many potential vectors or pathways could the taxon use to disperse within the risk assessment area (with suitable habitats nearby)?	>1	https://www.missouribotanicalgarden.org/plantfinder/PlantFinderDetails.aspx?taxonid=279349; Idžojtić M. (2013): DENDROLOGIJA – cvijet, češer, plod, sjeme. Denona d.d. (In Croatian).	High
36	7.02	Will any of these vectors or pathways bring the taxon in close proximity to one or more protected areas?	No	Professional judgement	Low
37	7.03	Does the taxon have a specialised means of attachment such that it enhances the likelihood of dispersal?	Yes	https://www.missouribotanicalgarden.org/plantfinder/PlantFinderDetails.aspx?taxonid=279349; Ames, O. 1931. Davidia involucrata. Bulletin of Popular Information, 5(7): 25–28.; Idžojtić M. (2013): DENDROLOGIJA – cvijet, češer, plod, sjeme. Denona d.d. (In Croatian).	Very high
38	7.04	Is natural dispersal of the taxon likely to occur as seeds or spores in the risk	No	Professional judgement	Low
39	7.05	Is natural dispersal of the taxon likely to occur as fragments or seedlings in the risk assessment area?	Yes	https://alienplantsbelgium.myspecies.info/content/cornaceae	Low
40	7.06	Are any life stages of the taxon likely to migrate into the risk assessment area for reproduction?	Yes	https://alienplantsbelgium.myspecies.info/content/cornaceae	Low
41	7.07	Are propagules of the taxon likely to be dispersed in the risk assessment area by other species?	No	Professional judgement	Medium
42	7.08	Is dispersal of the taxon along any of the vectors or pathways mentioned in the previous seven Questions (35–41: i.e. either unintentional or intentional) likely to be	No	Professional judgement based on eg. https://www.missouribotanicalgarden.org/plantfinder/PlantFinderDetails.aspx?taxonid=279349; Ames, O. 1931. Davidia involucrata. Bulletin of Popular Information, 5(7): 25–28. etc.	High
43	7.09	Is dispersal of the taxon density dependent?	No	Professional judgement	Medium
8. Tolerance attributes					
44	8.01	Is the taxon able to withstand being in water for extended periods at some stage of its life cycle?	No	https://plantura.garden/uk/trees-shrubs/handkerchief-tree/handkerchief-tree-overview	Very high
45	8.02	Is the taxon tolerant of a wide range of soil or air quality conditions?	No	https://plantura.garden/uk/trees-shrubs/handkerchief-tree/handkerchief-tree-overview	Very high
46	8.03	Can the taxon be controlled or eradicated in the wild with chemical, biological or other agents and means?	No	No exact references found, professional judgement	Medium
47	8.04	Is the taxon likely to benefit from environmental or human disturbance?	No	Professional judgement	Medium
48	8.05	Is the taxon able to tolerate soil acidity, salinity or other parameter levels that are higher or lower than those found in its usual	No	Professional judgement; https://plantura.garden/uk/trees-shrubs/handkerchief-tree/handkerchief-tree-overview	Medium
49	8.06	Are there effective natural enemies of the taxon present in the risk assessment area?	No	No references found	Low
**C. Climate change**					
9. Climate change					
50	9.01	Under the predicted future climatic conditions, are the risks of entry into the risk assessment area posed by the taxon likely to increase, decrease or not change?	No change	Professional judgement	Medium
51	9.02	Under the predicted future climatic conditions, are the risks of establishment within the risk assessment area posed by the taxon likely to increase, decrease or not	No change	Professional judgement	Medium

52	9.03	Under the predicted future climatic conditions, are the risks of dispersal within the risk assessment area posed by the taxon likely to increase, decrease or not change?	No change	Professional judgement	Low
53	9.04	Under the predicted future climatic conditions, what is the likely magnitude of future potential impacts by the taxon on biodiversity or ecosystem integrity and	No change	Professional judgement	Medium
54	9.05	Under the predicted future climatic conditions, what is the likely magnitude of future potential impacts by the taxon on ecosystem structure or function?	No change	Professional judgement	Medium
55	9.06	Under the predicted future climatic conditions, what is the likely magnitude of future potential impacts by the taxon on ecosystem services and related socio-	No change	Professional judgement	Medium

Statistics	
**Scores**	
**BRA**	**3.0**
**BRA Outcome**	**Medium**
**BRA+CCA**	**3.0**
**BRA+CCA Outcome**	**Medium**
**Score partition**	
**A. Biogeography/Historical**	**2.0**
1. Domestication/Cultivation	*2.0*
2. Climate, distribution and introduction risk	*0.0*
3. Invasive elsewhere	*0.0*
**B. Biology/Ecology**	**1.0**
4. Undesirable (or persistence) traits	*1.0*
5. Resource exploitation	*0.0*
6. Reproduction	*0.0*
7. Dispersal mechanisms	*2.0*
8. Tolerance attributes	*-2.0*
**C. Climate change**	**0.0**
9. Climate change	*0.0*
**Answered Questions**	
Total	***55***
**A. Biogeography/Historical**	**13**
1. Domestication/Cultivation	*3*
2. Climate, distribution and introduction risk	*5*
3. Invasive elsewhere	*5*
**B. Biology/Ecology**	**36**
4. Undesirable (or persistence) traits	*12*
5. Resource exploitation	*2*
6. Reproduction	*7*
7. Dispersal mechanisms	*9*
8. Tolerance attributes	*6*
**C. Climate change**	**6**
9. Climate change	*6*
**Sectors affected**	
**Commercial**	**4**
**Environmental**	**1**
**Species or population nuisance traits**	**-1**

Thresholds	
**BRA**	**41**
**BRA+CCA**	**41**
**Confidence**	
**BRA+CCA**	**0.59**
**BRA**	**0.61**
**CCA**	**0.46**

Date and Time
**14/08/2024 11:49:45**

TPS-ISK v2

**Taxon and Assessor details**	
Category	Angiosperms
Taxon name	*Diospyros virginiana*
Common name	American Persimmon (Common Persimmon)
Assessor	Ivana Vitasović Kosić
**Risk screening context**	
Reason and socio-economic benefits	
Risk assessment area	Continental Europe
Taxonomy	Kingdom: Plantae Clade: Tracheophytes Clade: Angiosperms Clade: Eudicots Clade: Asterids
Native range	
Introduced range	
URL	

			Response	Justification (references or other information)	Confidence
**A. Biogeography/Historical**					
1. Domestication/Cultivation					
1	1.01	Has the taxon been the subject of domestication?	Yes	https://faculty.salisbury.edu/~chbriand/PDFs/Huntia05.pdf Marić, Mara; Žeravica, Domagoj Ivan; Paladin Soče, Ivana; Vitasović-Kosić, Ivana. New potentially invasive species in the flora of Croatia - Diospyros virginiana L. (Common persimmon) // Šumarski list, 148 (2024), 7-8; 341-352. doi: 10.31298/sl.148.7-	Very high
2	1.02	Is the taxon harvested in the wild and likely to be sold or used in its live form?	Yes	The greatest species diversity is found in the Asia–Pacific region (ca. 300 species), with several species known for their use as timber or as suppliers of edible fruits and many others as an important source of medicinal products (Lee et al. 1996; Mallavadhani et al. 1998; Wallnöffer 2001; Peyrat et al. 2016; Yi et al. 2016; Rauf et al. 2017, Tang et al. 2019). all citation are available in Marić et al_2024 Yi, Z., Qiao, J. J., Lu, G. Y., Wu, G., Xie, G. Y., & Qin, M. J. (2016). Identification of six species of medicinal Diospyros plants based on leaf macro-and micro-morphology. China Journal of Chinese Materia Medica, 41(21), 3942-3949. Marić, Mara; Žeravica, Domagoj Ivan; Paladin Soče, Ivana; Vitasović-Kosić, Ivana New potentially invasive species in the flora of Croatia - Diospyros virginiana L. (Common persimmon) // Šumarski list, 148 (2024), 7-8; 341-352. doi:	Very high
3	1.03	Does the taxon have invasive races, varieties, sub-taxa or congeners?	Yes	yes, Diospyros lotus. Marić, Mara; Žeravica, Domagoj Ivan; Paladin Soče, Ivana; Vitasović-Kosić, Ivana New potentially invasive species in the flora of Croatia - Diospyros virginiana L. (Common persimmon) // Šumarski list, 148 (2024), 7-8; 341-	Very high
2. Climate, distribution and introduction risk					
4	2.01	How similar are the climatic conditions between the risk assessment area and the taxon's native range?	High	Native range: Central & E. U.S.A. It is a tree and grows primarily in the temperate biome (POWO 2023). Introduced range: Europe, Bermuda, California, Palestine, Transcaucasus (POWO 2024). The natural areal of the species D. virginiana covers the eastern part of the United States (Idžojtić 2013), appearing in habitats from Texas to Connecticut and as far south as southern Florida (Vlahović 2019). Of concern is the fact that in humid areas of South America, it is not uncommon for D. virginiana to form thickets from its root suckers on abandoned agricultural land, which have a pronounced invasive potential through generative and vegetative spread (Peters et al. 1970; Burge, 2018). from Marić, Mara; Žeravica, Domagoj Ivan; Paladin Soče, Ivana; Vitasović-Kosić, Ivana New potentially invasive species in the flora of Croatia - Diospyros virginiana L. (Common persimmon) // Šumarski list, 148 (2024), 7-8; 341-352. doi: 10.31298/sl.148.7-	High
5	2.02	What is the quality of the climate matching data?	High	Native range: Central & E. U.S.A. It is a tree and grows primarily in the temperate biome (POWO 2023). Introduced range: Europe, Bermuda, California, Palestine, Transcaucasus (POWO 2024). The size of some populations suggests that some areas in California may be susceptible to invasion by this species. This is important because the climate in southern California, with its Mediterranean characteristics (mild, wet winters and hot, dry summers) is similar to the climate in parts of southern Europe and also southern Croatia. (Marić et al., 2024)	High
6	2.03	Is the taxon known to be present outside of cultivation in the risk assessment area?	Yes	Marić, Mara; Žeravica, Domagoj Ivan; Paladin Soče, Ivana; Vitasović-Kosić, Ivana New potentially invasive species in the flora of Croatia - Diospyros virginiana L. (Common persimmon) // Šumarski list, 148 (2024), 7-8; 341-352. doi: 10.31298/sl.148.7-8.2 Croatian localities outside the area of primary cultivation – 3 sites: according to gbif https://www.gbif.org/species/3032986 in	Very high
7	2.04	How many potential vectors could the taxon use to enter the risk assessment area?	>1	D. virginiana is a dioecious species, and on the Koločep island sites, flower and fruit formation have not yet occurred, making it in this phase impossible to determine whether they are male or female specimens. Also clonal analysis has not been done yet. Reproduction currently occurs exclusively through vegetative method. Interestingly, in the Trsteno Arboretum, aside from the original old specimen of Virginia persimmon planted in the mid-19th century (over 15 meters in height) which is female species i.e. fruiting, this species is spreading vegetatively in the area of the old olive grove near a dry-stone structure, showing potential	Very high

8	2.05	Is the taxon currently found in close proximity to, and likely to enter, the risk assessment area in the near future (e.g. unintentional or intentional introductions)?	Not applicable	Marić, Mara; Žeravica, Domagoj Ivan; Paladin Soče, Ivana; Vitasović-Kosić, Ivana New potentially invasive species in the flora of Croatia - Diospyros virginiana L. (Common persimmon) // Šumarski list, 148 (2024), 7-8; 341-352. doi: 10.31298/sl.148.7-	Very high
3. Invasive elsewhere					
9	3.01	Has the taxon become naturalised outside its native range?	Yes	Marić, Mara; Žeravica, Domagoj Ivan; Paladin Soče, Ivana; Vitasović-Kosić, Ivana New potentially invasive species in the flora of Croatia - Diospyros virginiana L. (Common persimmon) // Šumarski list, 148 (2024), 7-8; 341-352. doi: 10.31298/sl.148.7-8.2 Beside ini Brasil is invasive https://www.gbif.org/species/3032986 and inCaligornija (Burge 2018) Burge, D. O., 2018: Common persimmon (Diospyros virginiana L.), a naturalized potentially invasive species in the	Very high
10	3.02	In the taxon's introduced range, are there any known adverse impacts to wild or commercial species?	Yes	Marić, Mara; Žeravica, Domagoj Ivan; Paladin Soče, Ivana; Vitasović-Kosić, Ivana New potentially invasive species in the flora of Croatia - Diospyros virginiana L. (Common persimmon) // Šumarski list, 148 (2024), 7-8; 341-352. doi: 10.31298/sl.148.7-8.2 Records indicate that various species from the genus Diospyros were introduced to Europe from the 17th to the 19th century, serving different purposes, including ornamental and non-fruit-related uses. These species were often planted in orchards alongside citrus and olive trees (Kluge and Tessmer 2018). Most of these introductions involved evergreen species, while D. kaki L. f., D. lotus L., and D. virginiana L. are deciduous, adapted to colder climates. Kluge, R. A., M. A. Tessmer, 2018: Caqui - Diospyros kaki. In: S. Rodrigues, F.O. de Silva, E.S. de Brito	Medium
11	3.03	In the taxon's introduced range, are there any known adverse impacts to agriculture or forestry?	Yes	The habitat where the species D. virginiana appeared about four years ago is in the area of an abandoned olive grove along a road connecting two settlements on the island (Donje Čelo and Gornje Čelo), with an area of approximately 2900 m2 that is completely occupied by D. virginiana, which has started to intertwine with old neglected olive trees (Figure 2). It is anthropogenic soil of fields on colluvium of dolomite and flysch, with reduced water permeability, which is why fields in this central part of the island were the foundation of agricultural activity in the past. In the neglected field, in the herbaceous layer, ruderal and weed vegetation of the association Dauco majori-Foeniculetum vulgaris is predominantly present, with individual neglected olive trees (Olea europeae L.) and laurel (Laurus nobilis L.). Marić, Mara; Žeravica, Domagoj Ivan; Paladin Soče, Ivana; Vitasović-Kosić, Ivana New potentially invasive species in the flora of Croatia - Diospyros virginiana L. (Common persimmon) // Šumarski list	Medium
12	3.04	In the taxon's introduced range, are there any known adverse impacts to ecosystem	Yes	it reproduces very quickly and abundantly	Medium
13	3.05	In the taxon's introduced range, are there any known adverse socio-economic impacts?	Yes	my opinion	Low
**B. Biology/Ecology**					
4. Undesirable (or persistence) traits					
14	4.01	Is the taxon likely to be poisonous or pose other risks to human health?	No	no, it is edible.	Very high
15	4.02	Is the taxon likely to suppress the growth of one or more native species?	Yes	my opinion and Marić, Mara; Žeravica, Domagoj Ivan; Paladin Soče, Ivana; Vitasović-Kosić, Ivana New potentially invasive species in the flora of Croatia - Diospyros virginiana L. (Common persimmon) // Šumarski list, 148 (2024), 7-8; 341-352. doi:	Medium
16	4.03	Are there any threatened or protected native species that the taxon would parasitise in the risk assessment area?	No	my opinion	Medium
17	4.04	Is the taxon adaptable in terms of climatic and other environmental conditions, thus enhancing its potential persistence if it has invaded or is likely to invade the risk assessment area?	Yes	According to Burge (2018), in all populations, mature fruits were found in autumn, and these fruits produced viable seeds with filled embryos. It is not yet known if these populations result from asexual reproduction via root sprouting or sexual reproduction via diaspores. However, the production of viable diaspores in all surveyed populations indicates that sexual seed set is occurring. The size of some populations suggests that some areas in California may be susceptible to invasion by this species. This is important because the climate in southern California, with its Mediterranean characteristics (mild, wet winters and hot, dry summers) is similar to the climate in parts of southern Europe and also southern Croatia. Burge, D. O., 2018: Common persimmon (Diospyros virginiana L.), a naturalized potentially invasive species in the State of California, Mandroño 65(2): 96-100, San Luis Obispo County, USA Marić, Mara; Žeravica, Domagoj Ivan; Paladin Soče, Ivana; Vitasović-Kosić, Ivana New potentially invasive species in the flora of Croatia - Diospyros virginiana L. (Common persimmon) // Šumarski list, 148 (2024), 7-8; 341-	High

18	4.05	Is the taxon likely to disrupt terrestrial ecosystem function if it has invaded or is likely to invade the risk assessment area?	Yes	Marić, Mara; Žeravica, Domagoj Ivan; Paladin Soče, Ivana; Vitasović-Kosić, Ivana New potentially invasive species in the flora of Croatia - Diospyros virginiana L. (Common persimmon) // Šumarski list, 148 (2024), 7-8; 341-352. doi: 10.31298/sl.148.7-8.2 CONCLUSION In this study, the species Diospyros virginiana L. was recorded, and its distribution and spreading methods were identified. This study highlights the highly invasive nature of the species in abandoned agricultural areas of Koločep Island, where it shows a pronounced ability to spread vegetatively, and emphasizes the need for further monitoring of species D. virginiana. The species is currently spreading exclusively vegetatively. Although clonal selection has not yet taken place, i.e. it is not yet apparent that both female and male individuals are present, this study should indicate that the observed rapid vegetative spread poses a major threat to the abandoned agricultural land of Koločep Island. The eradication process should be carried out before possible differentiation into male and female individuals occurs, and thus before pollination and fruit formation, i.e., generative reproduction. Further research requires clonal selection and several years of detailed phenological observation of this species. This work aims to draw attention to the broader scientific and professional public about the need to monitor this	High
19	4.06	Is the taxon likely to exert adverse impacts on ecosystem services in the risk assessment area?	Yes	The species is currently spreading exclusively vegetatively. Although clonal selection has not yet taken place, i.e. it is not yet apparent that both female and male individuals are present, this study should indicate that the observed rapid vegetative spread poses a major threat to the abandoned agricultural land of Koločep Island. The eradication process should be carried out before possible differentiation into male and female individuals occurs, and thus before pollination and fruit formation, i.e., generative reproduction. Further research requires clonal selection and several years of detailed phenological observation of this species. This work aims to draw attention to the broader scientific and professional public about the need to monitor this species in anthropogenic and abandoned agricultural habitats.	Low
20	4.07	Is the taxon likely to host or function as a vector for recognised pests and pathogens that are present in the risk assessment area?	Yes	The species is resistant to aerosol salt also resistance to moderate soil salinity has been observed (Gilman et al. 1993), explaining the successful reproduction on the Koločep Island. In the context of the resistance of the species and its invasive potential, it is also important to mention that regarding diseases and pests, D. virginiana is a very resilient species. Namely, there has been noted only occasionally attacking by caterpillars, and occasionally exhibits leaf spot disease (causing defoliation) (Gilman et al. 1993; Berbegal et al. 2010). Gilman, E. F., D. G. Watson, 1993: Diospyros virginiana, Common persimmon, Environmental Horticulture Department, Florida Cooperative Extension Service, 1-3, University of Florida, SAD. Berbegal, M., Pérez-Sierra, A., Armengol, J. Park C. S., García-Jiménez. J., 2010: First Report of Circular Leaf Spot of Persimmon Caused by Mycosphaerella nawae in Spain, Plant Disease. 94 (3): 374., Minnesota, USA. Marić, Mara; Žeravica, Domagoj Ivan; Paladin Soče, Ivana; Vitasović-Kosić, Ivana New potentially invasive species in the flora of Croatia - Diospyros virginiana L. (Common persimmon) //	Medium
21	4.08	Is the taxon likely to host or function as a vector for recognised pests and pathogens that are absent in the risk assessment area?	Yes	.	Low
22	4.09	Is the taxon likely to be released from cultivation?	Yes	In the Dubrovnik area, the first known introduction of D. virginiana dates back to 1859 (Idžojtić et al. 2019) when it was planted in the Trsteno Arboretum during the ownership of Baltazar Paola Bassegli Gozze (Đurasović 1997; Obad Šćitaroci and Kovačević 2014). It is currently one of the eight oldest living trees in the arboretum (Idžojtić et al. 2019). During that period, plants were introduced to Trsteno from nurseries in Genoa, Padua, and Venice. There is no record of the plant being introduced to any other location in Dubrovnik during that period (Marić 2024).	High
23	4.10	Is the taxon versatile in habitat use?	Yes	The United States Department of Agriculture (USDA) notes it as a pioneer species, often colonizing abandoned fields, road edges, and fences. Its presence in forest stands and abandoned fields is considered undesirable, although some scientific articles have pointed to its strong invasive potential (Peters et al. 1970; Nesom 2000, Burge, 2018). Nesom, G., 2000: Common Persimmon, Diospyros virginiana L., USDA, NRCS, National Plant Data Center, Louisiana: https://plants.usda.gov/DocumentLibrary/plantguide/pdf/pg_divi5.pdf Peters, E. J., Stritzke, J., F., 1970: Control of Persimmon with Various Herbicides and Methods of Application. Weed Science, 18(5): 572-575, Georgia, USA. Burge, D. O., 2018: Common persimmon (Diospyros virginiana L.), a naturalized potentially invasive species in the State of California, Mandroño 65(2): 96-100, San Luis Obispo County, USA. Marić, Mara; Žeravica, Domagoj Ivan; Paladin Soče, Ivana; Vitasović-Kosić, Ivana New potentially invasive species in the flora of Croatia - Diospyros	High

24	4.11	Is it likely that the taxon's mode of existence or behaviours will reduce habitat quality for native species?	Yes	. it is edible and medicinally used. Peyrat, L. A., Eparvier, V., Eydoux, C., Guillemot, J. C., Stien, D., & Litaudon, M. (2016). Chemical diversity and antiviral potential in the pantropical Diospyros genus. Fitoterapia, 112, 9-15. Yi, Z., Qiao, J. J., Lu, G. Y., Wu, G., Xie, G. Y., & Qin, M. J. (2016). Identification of six species of medicinal Diospyros plants based on leaf macro-and micro-morphology. China Journal of Chinese Materia Medica,	High
25	4.12	Is the taxon likely to maintain a viable population even when present in low densities (or persist in adverse conditions by way of a dormant form)?	Yes	it reproduces very quickly and abundantly vegetatively.. Of concern is the fact that in humid areas of South America, it is not uncommon for D. virginiana to form thickets from its root suckers on abandoned agricultural land, which have a pronounced invasive potential through generative and vegetative spread (Peters et al. 1970; Burge, 2018). Peters, E. J., Stritzke, J., F., 1970: Control of Persimmon with Various Herbicides and Methods of Application. Weed Science, 18(5): 572-575, Georgia, USA	Very high
5. Resource exploitation					
26	5.01	Is the taxon likely to put threatened or protected native species under competitive pressure in the risk assessment area?	Yes	Marić, Mara; Žeravica, Domagoj Ivan; Paladin Soče, Ivana; Vitasović-Kosić, Ivana New potentially invasive species in the flora of Croatia - Diospyros virginiana L. (Common persimmon) // Šumarski list, 148 (2024), 7-8; 341-352. doi: 10.31298/sl.148.7-	High
27	5.02	Is the taxon likely to sequester resources to the detriment of native species in the risk assessment area?	Yes	my opinion	Low
6. Reproduction					
28	6.01	Is the taxon likely to exhibit changes in reproductive strategy in response to environmental conditions?	Yes	vegetatively invasion !!! In addition to reproductive ability, the phenotypic plasticity of a plant is mentioned, allowing plants to occupy a greater number of ecological niches, and the fact that they thrive in abandoned anthropogenic habitats (Vuković 2015). Due to the extremely invasive potential and fragile biological balance on an island ecosystem, this problem is even more pronounced on the small island of Koločep. Marić, Mara; Žeravica, Domagoj Ivan; Paladin Soče, Ivana; Vitasović-Kosić, Ivana New potentially invasive species in the flora of Croatia - Diospyros virginiana L. (Common persimmon) // Šumarski list, 148 (2024),	Very high
29	6.02	Is the taxon likely to produce viable propagules in the risk assessment area?	Yes	According to Richardson (2000), the definition of a species' invasiveness is associated with its ability to reproduce in larger numbers and at considerable distances from the parent plant (with a measure of distance >100 m; <50 years for species spreading by seeds and other propagules; >6 m/3 years for species spreading by roots, rhizomes, stolons, or creeping stems), enabling them to spread over a larger area. In the case of D. virginiana on the island of Koločep, these parameters coincide with the definition of invasiveness. Richardson, D. M., P. Pysek, M. Rejmánek, M. G. Barbour, F. D. Panetta, C. J. West, 2000: Naturalization and invasion of alien plants: concepts and definitions, Diversity and Distribution 6(1): 97, New Jersey, SAD. Marić, Mara; Žeravica, Domagoj Ivan; Paladin Soče, Ivana; Vitasović-Kosić, Ivana New potentially invasive species in the flora of Croatia - Diospyros virginiana L. (Common persimmon) //	Very high
30	6.03	Is the taxon likely to hybridise with native species under natural conditions?	No	notknown yet D. virginiana is a dioecious species, The species is currently spreading exclusively vegetatively. Although clonal selection has not yet taken place, i.e. it has not yet become apparent that both female and male indi viduals are present, this study should indicate that the ob served rapid vegetative spread poses a major threat to the abandoned agricultural land on Koločep Island. The eradi cation process should be carried out before the possible differentiation into male and female individuals occurs, and thus before pollination and fruit formation, i.e., generative reproduction. Further research requires clonal selection and several years of detailed phenological observation of this species. This work aims to draw attention to the broa der scientific and professional audience about the need to monitor this species in anthropogenic and abandoned agri cultural habitats	Medium
31	6.04	Is the taxon likely to be hermaphroditic or to exhibit asexual reproduction?	Yes	This study highlights the highly invasive nature of the species in abandoned agricultural areas of Koločep Island, where it shows a pronounced ability to spread vegetatively, and emphasizes the need for further monitoring of species D. virginiana. The species is currently spreading exclusively vegetatively. Although clonal selection has not yet taken place, i.e. it is not yet apparent that both female and male individuals are present, this study should indicate that the observed rapid vegetative spread poses a major threat to the abandoned agricultural land of Koločep Island. In the case of D. virginiana on the island of Koločep, which has a pronounced invasive potential, eradication should take place before fruits and seeds are formed that allow generative reproduction of this species, which is not yet the case. At this stage, eradication would prevent the further development of this potentially invasive species. As it is still a relatively localized site, combined mechanical and physical eradication methods should be used. It is necessary to carefully remove all fragments from which the plant can regrow and to carry out continuous monitoring over several years. from: Marić, Mara; Žeravica, Domagoj Ivan; Paladin Soče, Ivana; Vitasović-Kosić, Ivana New potentially invasive species in the flora of Croatia - Diospyros virginiana L. (Common	Very high

32	6.05	Is the taxon dependent on the presence of another species (or specific habitat features) to complete its life cycle?	No	it is not known	Medium
33	6.06	Is the taxon likely to produce a large number of propagules?	Yes	According to Richardson (2000), the definition of a species' invasiveness is associated with its ability to reproduce in larger numbers and at considerable distances from the parent plant (with a measure of distance >100 m; <50 years for species spreading by seeds and other propagules; >6 m/3 years for species spreading by roots, rhizomes, stolons, or creeping stems), enabling them to spread over a larger area. In the case of D. virginiana on the island of Koločep, these parameters coincide	Very high
34	6.07	How many time units (days, months, years) does the taxon require to reach the age at first reproduction?	>10	It is believed that the tree reaches optimal fertility at the age of 25-50 years, although it is considered that trees as young as 10 years old can bear fruit (Orwa et al. 2009). Orwa, C., A. Mutua, R. Kindt, R. Jamnadass, S. Anthony, 2009: Agroforestry Database: http://www.worldagroforestry.org/sites/treedbs/treedatabases.asp	Very high
7. Dispersal mechanisms					
35	7.01	How many potential vectors or pathways could the taxon use to disperse within the risk assessment area (with suitable habitats nearby)?	>1	The question arises about the vector of the introduction of this species to the island of Koločep. Potential of D. virginiana for invasion is evident in its vegetative growth, but this potential is also hidden by the ease with which seeds can spread by animals (primarily birds) rather than by people. However, it is known that mammals, including wild boars (Sus scrofa L.), gladly consume the fruit of this plant (Briand 2005; Mayer et al. 2009). Since the seeds are relatively large, it is highly likely that wild boars, which reach the island by swimming across the Koločep channel (approximately 1 km from the mainland) (Magaš et al. 2001), could be potential carriers of persimmon seeds to Koločep. The straight-line distance from the first introduced persimmon seedling in the Dubrovnik area, in the Trsteno Arboretum on the sea coast, where both female and male D. virginiana plants occur, to the habitat on the island of Koločep is about 5.5 km. Interestingly, Rebein et al.'s (2017) study showed that persimmon seeds that passed through the digestive system of some mammals were as viable as manually separated ones, or even better in germination compared to those germinating directly from intact fruit after the rotting process. The seeds are dormant in the winter period and begin the germination process in April or May after the soil temperature is above 15°C for about a month (Orwa et al. 2009). Briand, C. H., 2005: The common persimmon (Diospyros virginiana L.): The history of an underutilized fruit tree (16th – 19th centuries), Huntia 12 (1): 72., Pittsburgh, SAD. Mayer, J. J., I. L. Brisbin, 2009: Wild pigs, Biology, damage, control techniques, and management, Savannah River National Laboratory, 118, Aiken, SAD. Rebein, M., C. Davis, H. Abad, E. Stone, J. del Sol, N. Skinner, M. Moran, 2017: Seed dispersal of Diospyros virginiana in the past and the present: Evidence for a generalist evolutionary strategy, Ecology and Evolution, 1: 1-7, New Jersey, SAD. Orwa, C., A. Mutua, R. Kindt, R. Jamnadass, S. Anthony, 2009: Agroforestry Database: http://www.worldagroforestry.org/sites/treedbs/treedatabases.asp all from: Marić, Mara; Žeravica, Domagoj Ivan; Paladin Soče, [illegible]	Very high
36	7.02	Will any of these vectors or pathways bring the taxon in close proximity to one or more protected areas?	Yes	Marić, Mara; Žeravica, Domagoj Ivan; Paladin Soče, Ivana; Vitasović-Kosić, Ivana New potentially invasive species in the flora of Croatia - Diospyros virginiana L. (Common persimmon) // Šumarski list, 148 (2024), 7-8; 341-352. doi: 10.31298/sl.148.7-	Very high
37	7.03	Does the taxon have a specialised means of attachment such that it enhances the likelihood of dispersal?	Yes	yes, seeds passed thrue digestive system of some mammals (eg. Sus scrofa and birds) Fruit: The fruits are spherical or flattened-spherical, approximately 2.4 cm in size, orange-yellow, smooth, juicy, soft, and edible berries. The flesh is astringent when green, becoming sweet and luscious when ripe. The fruit contains 4 to 8 seeds, which are brown, shiny, and flat (approximately 1.5 cm long and 1 cm wide). nHowever, it is known that mammals, including wild boars (Sus scrofa L.), gladly consume the fruit of this plant (Briand 2005; Mayer et al. 2009). Since the seeds are relatively large, it is highly likely that wild boars, which reach the island by swimming across the Koločep channel (approximately 1 km from the mainland) (Magaš et al. 2001), could be potential carriers of persimmon seeds to Koločep. The straight-line distance from the first introduced persimmon seedling in the Dubrovnik area, in the Trsteno Arboretum on the sea coast, where both female and male D. virginiana plants occur, to the habitat on the island of Koločep is about 5.5 km. Interestingly, Rebein et al.'s (2017) study showed that persimmon seeds that passed through the digestive system of some mammals were as viable as manually separated ones, or even better in germination compared to those germinating directly from intact fruit after the rotting process. The seeds are dormant in the winter period and begin the [illegible]	High

38	7.04	Is natural dispersal of the taxon likely to occur as seeds or spores in the risk assessment area?	Yes	seeds that passed through the digestive system of some mammals were as viable as manually separated ones, or even better in germination compared to those germinating directly from intact fruit after the rotting process. Orwa et al 2009 and The species is resistant to aerosol salt also resistance to moderate soil salinity has been observed (Gilman et al. 1993), explaining the successful reproduction on the Koločep Island. Marić et al 2024, Gilman, E. F., D. G. Watson, 1993: Diospyros virginiana, Common persimmon, Environmental Horticulture Department, Florida Cooperative Extension Service, 1-3, University of Florida, SAD.	Very high
39	7.05	Is natural dispersal of the taxon likely to occur as fragments or seedlings in the risk assessment area?	Yes	Marić, Mara; Žeravica, Domagoj Ivan; Paladin Soče, Ivana; Vitasović-Kosić, Ivana New potentially invasive species in the flora of Croatia - Diospyros virginiana L. (Common persimmon) // Šumarski list, 148 (2024), 7-8; 341-352. doi: 10.31298/sl.148.7-8.2 The plant spreads vegetatively and possesses a robust root system extending laterally for several meters, completely covering the area. Additionally, it has grown into a dry stone wall structure along the road. According to the author's proposal, it was recently entered in the FCD as 2.1.1.3, a potentially invasive species, to provide a basis for future monitoring. Considering its invasive potential and the environmental damage it may cause, the authors suggest the eradication of the species from the area	Very high
40	7.06	Are any life stages of the taxon likely to migrate into the risk assessment area for reproduction?	Yes	The plant spreads vegetatively and possesses a robust root system extending laterally for several meters, completely covering the area. Additionally, it has grown into a dry stone wall structure along the road. According to the author's proposal, it was recently entered in the FCD as 2.1.1.3, a potentially invasive species, to provide a basis for future monitoring. Considering its invasive potential and the environmental damage it may cause, the authors suggest the eradication of the species from the area before it spreads to surrounding regions Marić et ell 2024	Very high
41	7.07	Are propagules of the taxon likely to be dispersed in the risk assessment area by other species?	Yes	The potential of D. vi rginiana for an invasion is evident in its vegetative growth, but this potential is also hidden by the ease with which seeds can spread by animals (primarily birds) rather than by pe ople. However, it is known that mammals, including wild boars (Sus scrofa L.), like to consume the fruit of this plant (Briand 2005; Mayer et al. 2009). Since the seeds are relati vely large, it is highly likely that wild boars, which reach the island by swimming across the Koločep channel (approxi mately 1 km from the mainland) (Magaš et al. 2001), could be potential carriers of persimmon seeds to Koločep. Marić et al 2024	Very high
42	7.08	Is dispersal of the taxon along any of the vectors or pathways mentioned in the previous seven Questions (35–41: i.e. either unintentional or intentional) likely to be	Yes	Mrić et al 2024	Very high
43	7.09	Is dispersal of the taxon density dependent?	Yes	The potential of D. vi rginiana for an invasion is evident in its vegetative growth, but this potential is also hidden by the ease with which seeds can spread by animals (primarily birds) rather than by pe ople. Marić et al. 2024	High
8. Tolerance attributes					
44	8.01	Is the taxon able to withstand being in water for extended periods at some stage of its life cycle?	Yes	my opinion	Medium
45	8.02	Is the taxon tolerant of a wide range of soil or air quality conditions?	Yes	D. virginiana often covers abandoned fields with a shrubby growth, emerging along roadsides and fences. It is frequently the first tree species to grow on abandoned and denuded cropland, well-adapted to environments with high insolation and low water supply (Morris 1965). This species is very adaptable to environmental conditions. D. virginiana is particularly resistant to cold growing condi tions (Zhabrovets et al. 2019), but also resistant to high solar radiation and drought (Gilman et al. 1993; Orwa et al. 2009; Intrigliolo et al. 2015). The altitudinal limit at which D. vir giniana does not occur is 1200 m above sea level. According to the literature, the maximum temperatures that the species can tolerate are 35°C in summer and -12°C in winter (Orwa et al. 2009). Although it prefers full sun, the plant can also thrive in the shade. In addition to prolonged drought, the plant also tolerates occasional flooding. It is important that the plant receives at least 46 mm of water from rainfall dur ing the growing season. It thrives on almost all soils, but grows best on alluvial soils such as clay and heavy loam with a neutral pH (Orwa et al. 2009). all from Marić et al. 2024 Morris, R.C., 1965: Common persimmon (Diospyros virginiana L.). In: Silvics of forest trees of the United States, Fowells, H.A., comp. U.S. Department of	High

46	8.03	Can the taxon be controlled or eradicated in the wild with chemical, biological or other agents and means?	Yes	Different management methods were tried in America in order to control this species. Peters and Stritzke (1970) compared various rates of herbicides affecting foliage, stems, and roots, with varying effectiveness (Peters, 1970; Nesom 2000), raising ecological concerns. It has been ob served that the plant can sprout from root suckers after a f ire. According to Halls (1990), inoculation of persimmon stumps with a fungus (Nalanthamala diospyri (Crand.) Sc hroers & M.J. Wingf. 2005) was found to be an effective means of preventing subsequent sprouting. Especially in the European-Mediterranean zone, to which the studied area of Koločep Island belongs, the probability of frequent f ires is high, which makes the monitoring and control of the species D. virginiana extremely important. Marić et al. 2024 Nesom, G., 2000: Common Persimmon, Diospyros virginiana L., USDA, NRCS, National Plant Data Center, Louisiana: https://plants.usda.gov/DocumentLibrary/plantguide/pdf/pg_divi5.pdf Halls, L. K., 1990: Diospyros virginiana L. common persimmon. Silvics of North America, 2: 294-298. Washington	High
47	8.04	Is the taxon likely to benefit from environmental or human disturbance?	Yes	It has been observed that the plant can sprout from root suckers after a fire. Especially in the European-Mediterranean zone, to which the studied area of Koločep Island belongs, the probability of frequent f ires is high, which makes the monitoring and control of the species D. virginiana extremely important. Marić et al 2024	High
48	8.05	Is the taxon able to tolerate soil acidity, salinity or other parameter levels that are higher or lower than those found in its usual environment?	Yes	The species is resistant to aerosol salt. Also, resistance to moderate soil salinity has been observed (Gilman et al. 1993), explaining the successful reproduction on the Kolo čep Island. In the context of the resistance of the species and its invasive potential, it is also important to mention that regarding diseases and pests, D. virginiana is a very re silient species. Namely, only occasional attacking by cater pillars, and occasional exhibits of leaf spot disease (causing defoliation) have been recorded (Gilman et al. 1993; Ber begal et al. 2010), all from Mrić et al	Very high
49	8.06	Are there effective natural enemies of the taxon present in the risk assessment area?	Yes	Different management methods were tried in America in order to control this species. Peters and Stritzke (1970) compared various rates of herbicides affecting foliage, stems, and roots, with varying effectiveness (Peters, 1970; Nesom 2000), raising ecological concerns. It has been ob served that the plant can sprout from root suckers after a f ire. According to Halls (1990), inoculation of persimmon stumps with a fungus (Nalanthamala diospyri (Crand.) Sc hroers & M.J. Wingf. 2005) was found to be an effective means of preventing subsequent sprouting. Especially in the European-Mediterranean zone, to which the studied area of Koločep Island belongs, the probability of frequent f ires is high, which makes the monitoring and control of the species D. virginiana extremely important Marić et al 2024	High
**C. Climate change**					
9. Climate change					
50	9.01	Under the predicted future climatic conditions, are the risks of entry into the risk assessment area posed by the taxon likely to increase, decrease or not change?	Increase	Common persimmon is easy to grow in the Mediterranean basin, and currently, there are no limiting factors, aside from water supply in some areas and low spring tempera tures in others (Bellini 2002). To our knowledge, data on the invasiveness of this species in other parts of Europe are not available or do not exist.	High
51	9.02	Under the predicted future climatic conditions, are the risks of establishment within the risk assessment area posed by the taxon likely to increase, decrease or not change?	Increase	Common persimmon is easy to grow in the Mediterranean basin, and currently, there are no limiting factors, aside from water supply in some areas and low spring tempera tures in others (Bellini 2002). To our knowledge, data on the invasiveness of this species in other parts of Europe are not available or do not exist.	Very high
52	9.03	Under the predicted future climatic conditions, are the risks of dispersal within the risk assessment area posed by the taxon likely to increase, decrease or not change?	Increase	Common persimmon is easy to grow in the Mediterranean basin, and currently, there are no limiting factors, aside from water supply in some areas and low spring tempera tures in others (Bellini 2002). To our knowledge, data on the invasiveness of this species in other parts of Europe are not available or do not exist.	Very high
53	9.04	Under the predicted future climatic conditions, what is the likely magnitude of future potential impacts by the taxon on biodiversity or ecosystem integrity and	Higher	Its presence in forest stands and abandoned fields is considered undesirable, although some scientific articles have pointed to its strong invasive potential (Peters et al. 1970; Nesom 2000, Burge, 2018).	High
54	9.05	Under the predicted future climatic conditions, what is the likely magnitude of future potential impacts by the taxon on ecosystem structure or function?	Higher	Its presence in forest stands and abandoned fields is considered undesirable, although some scientific articles have pointed to its strong invasive potential (Peters et al. 1970; Nesom 2000, Burge, 2018). Common persimmon is easy to grow in the Mediterranean basin, and currently, there are no limiting factors, aside from water supply in some areas and low spring tempera tures in others (Bellini 2002). To our knowledge, data on the invasiveness of this species in other parts of Europe are not available or do not	High
55	9.06	Under the predicted future climatic conditions, what is the likely magnitude of future potential impacts by the taxon on ecosystem services and related socio-economic factors?	Higher	Its presence in forest stands and abandoned fields is considered undesirable, although some scientific articles have pointed to its strong invasive potential (Peters et al. 1970; Nesom 2000, Burge, 2018). Common persimmon is easy to grow in the Mediterranean basin, and currently, there are no limiting factors, aside from water supply in some areas and low spring tempera tures in others (Bellini 2002). To our knowledge, data on the invasiveness of this species in other parts of Europe are not available or do not exist." Marić et al 2024	High

Statistics

Scores	
**BRA**	**58.0**
**BRA Outcome**	**High**
**BRA+CCA**	**70.0**
**BRA+CCA Outcome**	**High**
**Score partition**	
**A. Biogeography/Historical**	**24.0**
1. Domestication/Cultivation	*4.0*
2. Climate, distribution and introduction risk	*2.0*
3. Invasive elsewhere	*18.0*
**B. Biology/Ecology**	**34.0**
4. Undesirable (or persistence) traits	*10.0*
5. Resource exploitation	*7.0*
6. Reproduction	*3.0*
7. Dispersal mechanisms	*9.0*
8. Tolerance attributes	*5.0*
**C. Climate change**	**12.0**
9. Climate change	*12.0*
**Answered Questions**	
Total	***55***
**A. Biogeography/Historical**	**13**
1. Domestication/Cultivation	*3*
2. Climate, distribution and introduction risk	*5*
3. Invasive elsewhere	*5*
**B. Biology/Ecology**	**36**
4. Undesirable (or persistence) traits	*12*
5. Resource exploitation	*2*
6. Reproduction	*7*
7. Dispersal mechanisms	*9*
8. Tolerance attributes	*6*
**C. Climate change**	**6**
9. Climate change	*6*
**Sectors affected**	
**Commercial**	**21**
**Environmental**	**17**
**Species or population nuisance traits**	**37**

Thresholds	
**BRA**	**41**
**BRA+CCA**	**41**
**Confidence**	
**BRA+CCA**	**0.78**
**BRA**	**0.78**
**CCA**	**0.83**

Date and Time
**10/08/2024 18:59:12**

TPS-ISK v2

**Taxon and Assessor details**	
Category	Angiosperms
Taxon name	*Gleditsia triacanthos*
Common name	Honey Locust
Assessor	Sara Essert
**Risk screening context**	
Reason and socio-economic benefits	
Risk assessment area	Continental Europe
Taxonomy	
Native range	
Introduced range	
URL	

			Response	Justification (references or other information)	Confidence
**A. Biogeography/Historical**					
1. Domestication/Cultivation					
1	1.01	Has the taxon been the subject of domestication?	Yes	"From the 17th century, G. triacanthos was largely introduced into other States in the USA and in South America, Africa, South Western and South Asia and Europe (Russia, Ukraine, Romania and former Yugoslavia)." & "G. triacanthos bear seed from about 10 until 100 years of age..." - CABI digital library https://www.cabidigitallibrary.org/doi/10.1079/cabicompendium.25272#sec-9	Very high
2	1.02	Is the taxon harvested in the wild and likely to be sold or used in its live form?	Yes	"As a fast growing species with an attractive crown, it is also suitable for ornamental planting.", "use in agrosilvopastural systems producing cattle forage, particularly in semiarid regions. It allows sustained and diverse forage production during periods of fodder scarcity..." etc - CABI digital library https://www.cabidigitallibrary.org/doi/10.1079/cabicompendium.25272#sec-9	Very high
3	1.03	Does the taxon have invasive races, varieties, sub-taxa or congeners?	No	I found no data about invasive relatives (GBIF database or anywhere else).	Medium
2. Climate, distribution and introduction risk					
4	2.01	How similar are the climatic conditions between the risk assessment area and the taxon's native range?	High	The species grows in a variety of climates in its original habitat, and according to the map by Peel et al 2007, some of these climates coincide with those in RA. " It thrives in climates ranging from cold-temperate to subtropical within its native habitat " - CABI Digital library https://www.cabidigitallibrary.org/doi/10.1079/cabicompendium.25272#sec-16	Very high
5	2.02	What is the quality of the climate matching data?	High	Given that I used a map from a scientific paper (Peel et al 2007), I would say that the reliability is high.	High
6	2.03	Is the taxon known to be present outside of cultivation in the risk assessment area?	Yes	1) Italija – locally naturalised http://dryades.units.it/floritaly/index.php?procedure=taxon_page&tipo=all&id=2242) 2) Serbia - "This species is mainly present in abandoned meadows, field boundaries, forest edges, and along roads, but only in the Protection Regime III zone at the foothill of Mt. Avala. It is frequent in black locust stands north of the Vranovac brook. It also grows both as a shrub and as a tall tree, mostly along one part of the ring road on Mt. Avala and in black locust stands. It was not observed in natural forest communities." - Glišić et al (2014) GIS analysis of spatial distribution of invasive tree species in the protected natural area of Mt. Avala (Serbia), Botanica Serbica 38(1), 131-138 3) Spain – „The population of G. triacanthos spreads along 1,900 m (Figure 3), and occupies the ephemeral and temporary stretches of the river. The population structure includes seedlings, young vegetative saplings and large adult flowering and fruiting individuals, the latter often forming dense spiny thickets that impede passing through. “ - Dana et al	Very high
7	2.04	How many potential vectors could the taxon use to enter the risk assessment area?	>1	„1) G. triacanthos is probably spread along water courses during periods of flooding. 2) The plump pods constitute excellent feed for animals, and it is assumed that both livestock and wild animals are able to spread seed widely. 3) Since the outbreak of Dutch elm disease, the artificial spread and planting of G. triacanthos has increased to substitute the role of elms (Ulmus spp.) as roadside trees, in agroforestry and especially as a fodder tree.“ - CABI Digital Library https://www.cabidigitallibrary.org/doi/10.1079/cabicompendium.2	Very high
8	2.05	Is the taxon currently found in close proximity to, and likely to enter, the risk assessment area in the near future (e.g. unintentional or intentional introductions)?	Not applicable	The taxon is already present in RA - https://www.gbif.org/species/2959443	Very high
3. Invasive elsewhere					

9	3.01	Has the taxon become naturalised outside its native range?	Yes	According to GBIF (https://www.gbif.org/species/2959443) there is an evidence of impact of the species in Argentina, Uruguay, Türkiye and Turkmenistan. "It is extremely invasive in certain areas of South America (Fernandez et al. 2017), Australia (Randall 2017) and South Africa (Henderson 2007)." - Dana et al. (2022), BioInvasions Records 11(4): 819–829, https://doi.org/10.3391/bir.2022.11.4.01 "Proved invasive outside its native range" - CABI Digital Library, https://www.cabidigitallibrary.org/doi/10.1079/cabicompendium.25272#sec-12 "Introduction into Australia G. triacanthos was first introduced into Australia in the mid 1800s when William Macarthur planted it at 'Camden Park' at Camden in New South Wales. In Queensland, it was planted in 1907 as a fodder and ornamental tree. G. triacanthos was first reported as a pest in	Very high
10	3.02	In the taxon's introduced range, are there any known adverse impacts to wild or commercial species?	Yes	„…the species is colonizing spaces that could be occupied by native riparian plant communities. Another impact is the homogenisation process associated with invasions, which includes the taxonomic homogenisation of plant communities (McKinney and Lockwood 1999; Olden 2006; Garcillán et al. 2014; Elliott and Davies 2019). Physiognomy is also affected since the colonization by G. triacanthos may result either in a replacement of tree species, or in a structural change from smaller vegetation to a riparian community dominated by trees (Gantes et al. 2011). Also, and in agreement with literature (Sargent 1965; Ghersa et al. 2002; Speroni and de Viana 2001; State of Queensland 2016), we have observed in the studied area that the species forms dense thickets that could impede the development of other species or the use of the stream by animals (e.g. as an ecological corridor). OtherIn South America, for instance, it has been shown to prevent or complicate the entrance of pioneer native trees for up to 37 years (Ceballos et al. 2020), to increase primary net production and respiration in summer and to greatly reduce solar irradiation, amplitude water temperature, the richness of invertebrates communities, and the floating, emergent and submerged macrophytes (Giorgi et al. 2014)." - Dana et al. (2022), BioInvasions Records 11(4): 819–829, ttps://doi.org/10.3391/bir.2022.11.4.01 According to GBIF	Very high
11	3.03	In the taxon's introduced range, are there any known adverse impacts to agriculture or forestry?	No	I didn't find any information about it.	Medium
12	3.04	In the taxon's introduced range, are there any known adverse impacts to ecosystem	No	I didn't find any information about it.	Medium
13	3.05	In the taxon's introduced range, are there any known adverse socio-economic impacts?	No	I didn't find any information about it.	Medium
**B. Biology/Ecology**					
4. Undesirable (or persistence) traits					
14	4.01	Is the taxon likely to be poisonous or pose other risks to human health?	No	The species is not toxic. "Honey Locust trees (Gleditsia triacanthos) are not toxic to humans. - https://greg.app/honey-locust-toxic-to-humans). I put the answer "No", although it has large thorns that can just theoretically cause pain and discomfort to humans ("Branches are densely armed with reddish, simple or compound stout thorns, flattened at the base." - CABI Digital Library https://www.cabidigitallibrary.org/doi/10.1079/cabicompendium.2	Very high
15	4.02	Is the taxon likely to suppress the growth of one or more native species?	Yes	"Although information about the precise ecological impact is not yet available for the study area, it is clear that the species is colonizing spaces that could be occupied by native riparian plant communities." - Dana et al. (2022)Gleditsia triacanthos invasive in Spain, BioInvasions Records 11(4): 819–829,	High
16	4.03	Are there any threatened or protected native species that the taxon would parasitise in the risk assessment area?	No	The taxon is not a parasite.	Very high
17	4.04	Is the taxon adaptable in terms of climatic and other environmental conditions, thus enhancing its potential persistence if it has invaded or is likely to invade the risk assessment area?	Yes	I haven't found a sentence in the literature that claims that it is an extremely climatically or environmentally adaptable species, but considering that the species grows successfully all over Europe, I would still say that it is adaptable. (Although, there are certainly plants that are much more adaptable.)	Medium
18	4.05	Is the taxon likely to disrupt terrestrial ecosystem function if it has invaded or is likely to invade the risk assessment area?	No	For now, there doesn't seem to be much of a chance for that to happen in the foreseeable future.	Medium
19	4.06	Is the taxon likely to exert adverse impacts on ecosystem services in the risk	No	For now, there doesn't seem to be much of a chance for that to happen in the foreseeable future.	Medium

20	4.07	Is the taxon likely to host or function as a vector for recognised pests and pathogens that are present in the risk assessment area?	Yes	Although I really searched a lot, I didn't find specific scientific papers (or even horticulture articles) about (specific) pests on Gleditchia in Europe. In the end, I'm pasting what AI (Chat GPT) wrote about autochthonous pests on the species: "In Europe, there are several natural enemies that can affect Gleditsia triacanthos, although these enemies may not be specific to this species alone. Here are a few examples: Insects: Aphids (Aphididae): These small pests often attack various tree species, including Gleditsia, and can cause a noticeable decline in plant health. Leafhoppers (Cicadellidae) and Psyllids (Psyllidae): These insects can cause damage by sucking plant sap and transmitting plant diseases. Fungal Diseases: Fusarium spp.: Various species of fungi from the genus Fusarium can cause diseases affecting the roots and wood of Gleditsia. Leaf spot (Diplocarpon rosae): This fungus can cause leaf diseases that, although specific to roses, may also affect other tree and shrub species. Nematodes: Root-knot nematodes (Meloidogyne spp.): These microscopic worms can cause root damage, which can weaken the health of Gleditsia. Ophiostoma ulmi: Although not specific to Gleditsia, this pathogen	Medium
21	4.08	Is the taxon likely to host or function as a vector for recognised pests and pathogens that are absent in the risk assessment area?	Yes	The species Agrilus difficilis and Homadaula anisocentra are a non-native pests that are currently only present in North America, and represent a potential problem if transferred to Europe (RA). (https://scholar.valpo.edu/cgi/viewcontent.cgi?referer=&httpsredir=1&article=1552&context=tgle,)	High
22	4.09	Is the taxon likely to be released from cultivation?	Not applicable	The taxon is already released into RA.	Very high
23	4.10	Is the taxon versatile in habitat use?	Yes	The species seems to be adaptable to different habitats: "It is fairly tolerant of acid and alkaline soils, but best development is usually on soils having a pH between 6.0 and 8.0. Preliminary tests using artificially salinized soils have determined that young saplings and seed germination are not affected by salinity (for example, 0.2% of NaCl in soil dry weight), although whether honey-locust can tolerate the cumulative effects of salinity over periods of years is unclear (Burns and Honkala, 1990).It can behave as a very hardy and drought-resistant species when planted elsewhere, but water stress can induce early leaf abscission and interact with parasites (Smitley and Peterson,	Very high
24	4.11	Is it likely that the taxon's mode of existence or behaviours will reduce habitat quality for native species?	No	The plant is not totxic or hramful for animals: "Honey-locust pods are eaten by cattle, goats, deer, opossum, squirrel, rabbits, quail, crows, and starling. White-tailed deer and rabbits eat the soft bark of young trees in winter, and livestock and deer eat young vegetative growth." - USDA, NRCS, Nesom G, 2000	High
25	4.12	Is the taxon likely to maintain a viable population even when present in low densities (or persist in adverse conditions by way of a dormant form)?	Yes	I believe that this is possible (especially in places in the world where the species is invasive and where it is autochthonous), but I found no research on the topic.	Low
5. Resource exploitation					
26	5.01	Is the taxon likely to put threatened or protected native species under competitive pressure in the risk assessment area?	No	For now, there doesn't seem to be much of a chance for that to happen in the foreseeable future, because I found just two reports for species invasion in RA (Serbia and Spain).	Medium
27	5.02	Is the taxon likely to sequester resources to the detriment of native species in the risk assessment area?	Yes	The species will be in competition for space, nutrients and light with other plant species on habitat.	Medium
6. Reproduction					
28	6.01	Is the taxon likely to exhibit changes in reproductive strategy in response to environmental conditions?	No	I did not find information about this anywhere in the literature.	Medium
29	6.02	Is the taxon likely to produce viable propagules in the risk assessment area?	Yes	"The population of G. triacanthos spreads along 1,900 m, and occupies the ephemeral and temporary stretches of the river. The population structure includes seedlings, young vegetative saplings and large adult flowering and fruiting individuals, the latter often forming dense spiny thickets that impede passing through." - Dana et al. (2022), BioInvasions Records 11(4): 819–829, https://doi.org/10.3391/bir.2022.11.4.01	Very high
30	6.03	Is the taxon likely to hybridise with native species under natural conditions?	No	No other species of the genus Gleditsia grow naturally in RA, so I believe that hybridization with native species is unlikely.	Medium
31	6.04	Is the taxon likely to be hermaphroditic or to exhibit asexual reproduction?	Yes	Like most other plants species, the taxon can be propagated vegetatively. "Honey-locust also reproduces from stump and root sprouts." - USDA, NRCS, Nesom G, 2000; "G. triacanthos can easily be grown from seed, suckers or cuttings..." - CABI Digital Library, https://www.cabidigitallibrary.org/doi/10.1079/cabicompendium.2	Very high
32	6.05	Is the taxon dependent on the presence of another species (or specific habitat features) to complete its life cycle?	No	I did not find information that the taxon is dependent on the presence of another species.	High
33	6.06	Is the taxon likely to produce a large number of propagules?	No	The species has relatively large seeds, so I believe that it does not belong to the species that would be defined as producers of a large number of seeds. I'm not sure how a large number of seeds is defined, so I put the confidence level "low". "There are about 8000 seeds/kg," - CABI Digital Library, https://www.cabidigitallibrary.org/doi/10.1079/cabicompendium.25272#sec-9; "Seed per Pound - 2800" - https://plants.usda.gov/home/plantProfile?symbol=GLTR	Low

34	6.07	How many time units (days, months, years) does the taxon require to reach the age at first reproduction?	10	"G. triacanthos bear seed from about 10 until 100 years of age..." - CABI Digital Library	Very high
7. Dispersal mechanisms					
35	7.01	How many potential vectors or pathways could the taxon use to disperse within the risk assessment area (with suitable habitats nearby)?	>1	1) Intentional planting - valuable ornamental and shelterbelt species 2) Water - spread along water courses during periods of flooding 3) Livestock and wild animals - excellent feed for animals - CABI Digital Library, https://www.cabidigitallibrary.org/doi/10.1079/cabicompendium.25272#sec-12	Very high
36	7.02	Will any of these vectors or pathways bring the taxon in close proximity to one or more protected areas?	Yes	All vectors can theoretically do this, and it seems to me that the human factor (intentional planting) is the fastest and most likely, because it is a fast and efficient way of spreading.	High
37	7.03	Does the taxon have a specialised means of attachment such that it enhances the likelihood of dispersal?	No	Fruits and seeds do not have hooks.	Very high
38	7.04	Is natural dispersal of the taxon likely to occur as seeds or spores in the risk assessment area?	Yes	The species can be spread by water, livestock and wild animals - CABI Digital Library, https://www.cabidigitallibrary.org/doi/10.1079/cabicompendium.25272#sec-12 "Frequency of diameters > 1 m decreased downstream of the invaded stretch (Figure 3), which suggests a downstream advance, probably, from an initial plantation site" - Dana et al. (2022), BioInvasions Records 11(4): 819–829, https://doi.org/10.3391/bir.2022.11.4.01	Very high
39	7.05	Is natural dispersal of the taxon likely to occur as fragments or seedlings in the risk assessment area?	No	I did not find data that the species was distributed in RA in that way.	Medium
40	7.06	Are any life stages of the taxon likely to migrate into the risk assessment area for reproduction?	Yes	"The population structure includes seedlings, young vegetative saplings and large adult flowering and fruiting individuals", "Frequency of diameters > 1 m decreased downstream of the invaded stretch, which suggests a downstream advance, probably, from an initial plantation site" - Dana et al. (2022), BioInvasions Records 11(4): 819–829, https://doi.org/10.3391/bir.2022.11.4.01	Very high
41	7.07	Are propagules of the taxon likely to be dispersed in the risk assessment area by other species?	Yes	"The plump pods constitute excellent feed for animals, and it is assumed that both livestock and wild animals are able to spread seed widely. " - CABI Digital Library, https://www.cabidigitallibrary.org/doi/10.1079/cabicompendium.25272#sec-29	Very high
42	7.08	Is dispersal of the taxon along any of the vectors or pathways mentioned in the previous seven Questions (35–41: i.e. either unintentional or intentional) likely to be	Yes	I consider propagation by deliberate planting (and by water & animals) to be rapid spreading.	Very high
43	7.09	Is dispersal of the taxon density dependent?	Yes	I am of the opinion that an increase in population density could have a positive effect on dispersal, as a greater number of seeds gives a greater chance of dispersal. However, I did not find any papers on that topic, so I put a low level of confidence.	Low
8. Tolerance attributes					
44	8.01	Is the taxon able to withstand being in water for extended periods at some stage of its life cycle?	Yes	"G. triacanthos is probably spread along water courses during periods of flooding." - CABI Digital Library, https://www.cabidigitallibrary.org/doi/10.1079/cabicompendium.25272#sec-29	High
45	8.02	Is the taxon tolerant of a wide range of soil or air quality conditions?	No	I didn't find any information about it.	Medium
46	8.03	Can the taxon be controlled or eradicated in the wild with chemical, biological or other agents and means?	Yes	If this report is to be believed, it seems possible. "Queensland's honey locust tree control program ranks as one of the most successful weed control programs ever undertaken in Australia and proves that well established weeds can be wiped out. The honey locust tree is no longer a problem in Queensland and can be suppressed indefi nitely with minimal resources." - Csurhes S. M, Successful control of honey locust trees (Gleditsia triacanthos L.) in Queensland, Fourteenth Australian Weeds Conference I found this researc where the authors describe implementing a control program of G. triacanthos by drilling the stem and applying herbicide - Sosa, Beatriz; Caballero, Natalia; Carvajales, Andrés; Fernández, Gabriela; Mello, Ana; Achkar, Marcel "Control de Gleditsia triacanthos en el Parque Nacional Esteros de Farrapos	Medium
47	8.04	Is the taxon likely to benefit from environmental or human disturbance?	Yes	Invasive alien species usually occupy degraded habitats, so I believe that habitat degradation would also be beneficial for this species. However, I have not found any data on this, so I put a low level of confidence.	Low
48	8.05	Is the taxon able to tolerate soil acidity, salinity or other parameter levels that are higher or lower than those found in its usual environment?	Yes	"It is fairly tolerant of acid and alkaline soils, but best development is usually on soils having a pH between 6.0 and 8.0. Preliminary tests using artificially salinized soils have determined that young saplings and seed germination are not affected by salinity (for example, 0.2% of NaCl in soil dry weight), although whether honey-locust can tolerate the cumulative effects of salinity over periods of years is unclear (Burns and Honkala, 1990)."- CABI Digital Library, https://www.cabidigitallibrary.org/doi/10.1079/cabicompendium.2	High

49	8.06	Are there effective natural enemies of the taxon present in the risk assessment area?	No	"In general, attacks by insects or pathogens are not fatal, but they do weaken the tree and slow growth. It is worth noting that G. triacanthos is highly resistant to attack by the polyphagous defoliator and forest pest Lymantria dispar, and was the most resistant of 17 tree species tested by Smitley et al. (1993)." - CABI Digital Library, https://www.cabidigitallibrary.org/doi/10.1079/cabicompendium.2	Low
**C. Climate change**					
9. Climate change					
50	9.01	Under the predicted future climatic conditions, are the risks of entry into the risk assessment area posed by the taxon likely to increase, decrease or not change?	Not applicable	The species is already found in RA.	Very high
51	9.02	Under the predicted future climatic conditions, are the risks of establishment within the risk assessment area posed by the taxon likely to increase, decrease or not change?	No change	The species in RA grows (in horticulture) in various climates and is currently not invasive anywhere and is naturalised in just few countries. I would not say that the climate is the decisive factor why the species did not escape from cultivation, so I do not believe that climate change will significantly affect the spread of the species. I didn't find research on that topic, so I put the	Low
52	9.03	Under the predicted future climatic conditions, are the risks of dispersal within the risk assessment area posed by the taxon likely to increase, decrease or not change?	No change	The species in RA grows (in horticulture) in various climates and is currently not invasive anywhere and is naturalised in just few countries. I would not say that the climate is the decisive factor for species dispersal in RA, so I do not believe that climate change will significantly affect the spread of the species. I didn't find research on that topic, so I put the confidence level "Low"	Low
53	9.04	Under the predicted future climatic conditions, what is the likely magnitude of future potential impacts by the taxon on biodiversity or ecosystem integrity and status?	No change	The species in RA grows (in horticulture) in various climates and is currently not invasive anywhere and is naturalised in just few countries. I would not say that the climate is the decisive factor for the speces' influence on bidiversity. I didn't find research on that topic, so I put the confidence level "Low"	Low
54	9.05	Under the predicted future climatic conditions, what is the likely magnitude of future potential impacts by the taxon on ecosystem structure or function?	No change	I would not say that the climate is the decisive factor for species naturalisation in RA, so I do not believe that climate change will significantly affect ecosystem structure. I didn't find research on that topic, so I put the confidence level "Low"	Low
55	9.06	Under the predicted future climatic conditions, what is the likely magnitude of future potential impacts by the taxon on ecosystem services and related socio-	No change	I would not say that the climate is the decisive factor for species naturalisation in RA, so I do not believe that climate change will significantly affect socio-economic factors. I didn't find research on that topic, so I put the confidence level "Low"	Low

**Statistics**	
**Scores**	
**BRA**	**29.0**
**BRA Outcome**	**Medium**
**BRA+CCA**	**29.0**
**BRA+CCA Outcome**	**Medium**
**Score partition**	
**A. Biogeography/Historical**	**10.0**
1. Domestication/Cultivation	*2.0*
2. Climate, distribution and introduction risk	*2.0*
3. Invasive elsewhere	*6.0*
**B. Biology/Ecology**	**19.0**
4. Undesirable (or persistence) traits	*6.0*
5. Resource exploitation	*2.0*
6. Reproduction	*1.0*
7. Dispersal mechanisms	*5.0*
8. Tolerance attributes	*5.0*
**C. Climate change**	**0.0**
9. Climate change	*0.0*
**Answered Questions**	
Total	***55***
**A. Biogeography/Historical**	**13**
1. Domestication/Cultivation	*3*
2. Climate, distribution and introduction risk	*5*
3. Invasive elsewhere	*5*
**B. Biology/Ecology**	**36**
4. Undesirable (or persistence) traits	*12*
5. Resource exploitation	*2*
6. Reproduction	*7*
7. Dispersal mechanisms	*9*
8. Tolerance attributes	*6*
**C. Climate change**	**6**
9. Climate change	*6*
**Sectors affected**	
**Commercial**	**11**
**Environmental**	**1**
**Species or population nuisance traits**	**22**

**Thresholds**	
**BRA**	**41**
**BRA+CCA**	**41**
**Confidence**	
**BRA+CCA**	**0.69**
**BRA**	**0.73**
**CCA**	**0.38**

Date and Time
09/08/2024 15:31:06

TPS-ISK v2

**Taxon and Assessor details**	
Category	Angiosperms
Taxon name	*Gymnocladus dioicus*
Common name	Kentucky Coffee Tree
Assessor	Božena Mitić
**Risk screening context**	
Reason and socio-economic benefits	ornamental tree
Risk assessment area	Continental Europe
Taxonomy	fam. Fabaceae (Caesalpinaceae)
Native range	North America (eastern and central parts)
Introduced range	Europe
URL	

			Response	Justification (references or other information)	Confidence
**A. Biogeography/Historical**					
1. Domestication/Cultivation					
1	1.01	Has the taxon been the subject of domestication?	Yes	https://temperate.theferns.info/plant/Gymnocladus+dioicus;	Medium
2	1.02	Is the taxon harvested in the wild and likely to be sold or used in its live form?	No	No exact reference found (professional judgement based on eg. Zaya and Howe (2009): Oecologia 161:221-226.)	Medium
3	1.03	Does the taxon have invasive races, varieties, sub-taxa or congeners?	No	No exact reference found (professional judgement based on eg. Zaya and Howe (2009): Oecologia 161:221-226.)	High
2. Climate, distribution and introduction risk					
4	2.01	How similar are the climatic conditions between the risk assessment area and the taxon's native range?	Medium	Peel et al. 2007	Medium
5	2.02	What is the quality of the climate matching data?	Medium	Peel et al. 2007	Medium
6	2.03	Is the taxon known to be present outside of cultivation in the risk assessment area?	No	No exact reference found	Medium
7	2.04	How many potential vectors could the taxon use to enter the risk assessment area?	>1	Zaya and Howe (2009): Oecologia 161:221-226.; Idžojtić M. (2013): DENDROLOGIJA – cvijet, češer, plod, sjeme. Denona d.d. (In Croatian).	Very high
8	2.05	Is the taxon currently found in close proximity to, and likely to enter, the risk assessment area in the near future (e.g. unintentional or intentional introductions)?	No	No exact references found	Medium
3. Invasive elsewhere					
9	3.01	Has the taxon become naturalised outside its native range?	No	No exact references found (professional judgment)	Medium
10	3.02	In the taxon's introduced range, are there any known adverse impacts to wild or commercial species?	No	No exact references found	Medium
11	3.03	In the taxon's introduced range, are there any known adverse impacts to agriculture or forestry?	No	No exact references found (professional judgment)	Medium
12	3.04	In the taxon's introduced range, are there any known adverse impacts to ecosystem	No	No exact references found (professional judgment)	Medium
13	3.05	In the taxon's introduced range, are there any known adverse socio-economic impacts?	No	No exact references found (professional judgment)	Medium
**B. Biology/Ecology**					
4. Undesirable (or persistence) traits					
14	4.01	Is the taxon likely to be poisonous or pose other risks to human health?	Yes	https://en.wikipedia.org/wiki/Kentucky_coffeetree; https://temperate.theferns.info/plant/Gymnocladus+dioicus; Idžojtić M. (2013): DENDROLOGIJA – cvijet, češer, plod, sjeme. Denona d.d. (In Croatian).	Very high
15	4.02	Is the taxon likely to suppress the growth of one or more native species?	No	No exact references found (professional judgment)	Medium
16	4.03	Are there any threatened or protected native species that the taxon would parasitise in the risk assessment area?	No	No exact references found (professional judgment)	Medium
17	4.04	Is the taxon adaptable in terms of climatic and other environmental conditions, thus enhancing its potential persistence if it has invaded or is likely to invade the risk	No	https://temperate.theferns.info/plant/Gymnocladus+dioicus; Zaya and Howe (2009): Oecologia 161:221-226.	High
18	4.05	Is the taxon likely to disrupt terrestrial ecosystem function if it has invaded or is likely to invade the risk assessment area?	No	No exact references found (professional judgment)	Medium
19	4.06	Is the taxon likely to exert adverse impacts on ecosystem services in the risk	No	No exact references found (professional judgment)	Medium
20	4.07	Is the taxon likely to host or function as a vector for recognised pests and pathogens that are present in the risk assessment area?	No	No exact references found (professional judgment)	Medium
21	4.08	Is the taxon likely to host or function as a vector for recognised pests and pathogens that are absent in the risk assessment area?	Yes	cf. https://www.picturethisai.com/disease/Gymnocladus-dioica-Canker-and-gummosis.html	Low
22	4.09	Is the taxon likely to be released from cultivation?	Yes	Zaya and Howe (2009): Oecologia 161:221-226.	Medium
23	4.10	Is the taxon versatile in habitat use?	Yes	Professional judgement based on Zaya and Howe (2009): Oecologia 161:221-226.	Medium
24	4.11	Is it likely that the taxon's mode of existence or behaviours will reduce habitat quality for native species?	No	No exact references found (professional judgment)	Medium

25	4.12	Is the taxon likely to maintain a viable population even when present in low densities (or persist in adverse conditions by way of a dormant form)?	Yes	Zaya and Howe (2009): Oecologia 161:221-226.; https://temperate.theferns.info/plant/Gymnocladus+dioicus	High
5. Resource exploitation					
26	5.01	Is the taxon likely to put threatened or protected native species under competitive pressure in the risk assessment area?	No	No exact references found (professional judgment)	Medium
27	5.02	Is the taxon likely to sequester resources to the detriment of native species in the risk assessment area?	No	No exact references found (professional judgment)	Medium
6. Reproduction					
28	6.01	Is the taxon likely to exhibit changes in reproductive strategy in response to environmental conditions?	Yes	Professional judgement based on Zaya and Howe (2009): Oecologia 161:221-226.; https://temperate.theferns.info/plant/Gymnocladus+dioicus; https://en.wikipedia.org/wiki/Kentucky_coffeetree	Medium
29	6.02	Is the taxon likely to produce viable propagules in the risk assessment area?	Yes	Professional judgement based on Zaya and Howe (2009): Oecologia 161:221-226. and	Medium
30	6.03	Is the taxon likely to hybridise with native species under natural conditions?	No	No living relatives (Zaya and Howe (2009): Oecologia 161:221-226.; https://en.wikipedia.org/wiki/Kentucky_coffeetree)	Very high
31	6.04	Is the taxon likely to be hermaphroditic or to exhibit asexual reproduction?	No	Zaya and Howe (2009): Oecologia 161:221-226.; https://en.wikipedia.org/wiki/Kentucky_coffeetree; Idžojtić M. (2013): DENDROLOGIJA – cvijet, češer, plod, sjeme. Denona d.d. (In Croatian).	Very high
32	6.05	Is the taxon dependent on the presence of another species (or specific habitat features) to complete its life cycle?	No	Zaya and Howe (2009): Oecologia 161:221-226.; https://en.wikipedia.org/wiki/Kentucky_coffeetree; Idžojtić M. (2013): DENDROLOGIJA – cvijet, češer, plod, sjeme. Denona d.d. (In Croatian).	High
33	6.06	Is the taxon likely to produce a large number of propagules?	Yes	Zaya and Howe (2009): Oecologia 161:221-226.; https://en.wikipedia.org/wiki/Kentucky_coffeetree; Idžojtić M. (2013): DENDROLOGIJA – cvijet, češer, plod, sjeme. Denona d.d. (In Croatian).	Very high
34	6.07	How many time units (days, months, years) does the taxon require to reach the age at first reproduction?	>10	No exact data found (professional judgement based on Idžojtić M. (2013): DENDROLOGIJA – cvijet, češer, plod, sjeme. Denona d.d. (In Croatian).; Zaya and Howe (2009): Oecologia 161:221-226.; https://www.goldleafhydroponics.com/how-to-start-kentucky-coffeetree-seeds-gymnocladus-dioicus-germination/ etc.	Medium
7. Dispersal mechanisms					
35	7.01	How many potential vectors or pathways could the taxon use to disperse within the risk assessment area (with suitable habitats nearby)?	One	https://en.wikipedia.org/wiki/Kentucky_coffeetree; Zaya and Howe (2009): Oecologia 161:221-226.	Medium
36	7.02	Will any of these vectors or pathways bring the taxon in close proximity to one or more protected areas?	No	Professional judgement base on Zaya and Howe (2009): Oecologia 161:221-226.	Low
37	7.03	Does the taxon have a specialised means of attachment such that it enhances the likelihood of dispersal?	No	Idžojtić M. (2013): DENDROLOGIJA – cvijet, češer, plod, sjeme. Denona d.d. (In Croatian).; Zaya and Howe (2009): Oecologia 161:221-226.	Very high
38	7.04	Is natural dispersal of the taxon likely to occur as seeds or spores in the risk assessment area?	Yes	Idžojtić M. (2013): DENDROLOGIJA – cvijet, češer, plod, sjeme. Denona d.d. (In Croatian).; Zaya and Howe (2009): Oecologia 161:221-226.	Medium
39	7.05	Is natural dispersal of the taxon likely to occur as fragments or seedlings in the risk assessment area?	Yes	Professional judgement based on Idžojtić M. (2013): DENDROLOGIJA – cvijet, češer, plod, sjeme. Denona d.d. (In Croatian).; Zaya and Howe (2009): Oecologia 161:221-226.	Medium
40	7.06	Are any life stages of the taxon likely to migrate into the risk assessment area for reproduction?	No	No exact references foun (professional judgement)	Low
41	7.07	Are propagules of the taxon likely to be dispersed in the risk assessment area by other species?	No	No exact references foun (professional judgement)	Low
42	7.08	Is dispersal of the taxon along any of the vectors or pathways mentioned in the previous seven Questions (35–41: i.e. either unintentional or intentional) likely to be	No	Professional judgement based on Zaya and Howe (2009): Oecologia 161:221-226.	High
43	7.09	Is dispersal of the taxon density dependent?	No	Professional judgement based on Zaya and Howe (2009): Oecologia 161:221-226.	Medium
8. Tolerance attributes					
44	8.01	Is the taxon able to withstand being in water for extended periods at some stage of its life cycle?	Yes	Zaya and Howe (2009): Oecologia 161:221-226.	Very high
45	8.02	Is the taxon tolerant of a wide range of soil or air quality conditions?	Yes	https://en.wikipedia.org/wiki/Kentucky_coffeetree	High
46	8.03	Can the taxon be controlled or eradicated in the wild with chemical, biological or other agents and means?	No	No exact references foun (professional judgement)	Medium
47	8.04	Is the taxon likely to benefit from environmental or human disturbance?	Yes	Zaya and Howe (2009): Oecologia 161:221-226.	High
48	8.05	Is the taxon able to tolerate soil acidity, salinity or other parameter levels that are higher or lower than those found in its usual	Yes	Zaya and Howe (2009): Oecologia 161:221-226.; https://en.wikipedia.org/wiki/Kentucky_coffeetree	Medium
49	8.06	Are there effective natural enemies of the taxon present in the risk assessment area?	Yes	Professional judgement based on eg. https://www.picturethisai.com/disease/Gymnocladus-dioica-Canker-and-gummosis.html	Low
**C. Climate change**					
9. Climate change					

50	9.01	Under the predicted future climatic conditions, are the risks of entry into the risk assessment area posed by the taxon likely to increase, decrease or not change?	No change	No exact references foun (professional judgement)	Medium
51	9.02	Under the predicted future climatic conditions, are the risks of establishment within the risk assessment area posed by the taxon likely to increase, decrease or not	No change	No exact references foun (professional judgement)	Medium
52	9.03	Under the predicted future climatic conditions, are the risks of dispersal within the risk assessment area posed by the taxon likely to increase, decrease or not change?	No change	No exact references foun (professional judgement)	Medium
53	9.04	Under the predicted future climatic conditions, what is the likely magnitude of future potential impacts by the taxon on biodiversity or ecosystem integrity and	No change	No exact references foun (professional judgement)	Medium
54	9.05	Under the predicted future climatic conditions, what is the likely magnitude of future potential impacts by the taxon on ecosystem structure or function?	No change	No exact references foun (professional judgement)	Medium
55	9.06	Under the predicted future climatic conditions, what is the likely magnitude of future potential impacts by the taxon on ecosystem services and related socio-	No change	No exact references foun (professional judgement)	Medium

**Statistics**	
**Scores**	
**BRA**	**12.0**
**BRA Outcome**	**Medium**
**BRA+CCA**	**12.0**
**BRA+CCA Outcome**	**Medium**
**Score partition**	
**A. Biogeography/Historical**	**1.0**
1. Domestication/Cultivation	*0.0*
2. Climate, distribution and introduction risk	*1.0*
3. Invasive elsewhere	*0.0*
**B. Biology/Ecology**	**11.0**
4. Undesirable (or persistence) traits	*4.0*
5. Resource exploitation	*0.0*
6. Reproduction	*1.0*
7. Dispersal mechanisms	*-1.0*
8. Tolerance attributes	*7.0*
**C. Climate change**	**0.0**
9. Climate change	*0.0*
**Answered Questions**	
Total	***55***
**A. Biogeography/Historical**	**13**
1. Domestication/Cultivation	*3*
2. Climate, distribution and introduction risk	*5*
3. Invasive elsewhere	*5*
**B. Biology/Ecology**	**36**
4. Undesirable (or persistence) traits	*12*
5. Resource exploitation	*2*
6. Reproduction	*7*
7. Dispersal mechanisms	*9*
8. Tolerance attributes	*6*
**C. Climate change**	**6**
9. Climate change	*6*
**Sectors affected**	
**Commercial**	**2**
**Environmental**	**0**
**Species or population nuisance traits**	**11**

**Thresholds**	
**BRA**	**41**
**BRA+CCA**	**41**
**Confidence**	
**BRA+CCA**	**0.57**
**BRA**	**0.58**
**CCA**	**0.50**

**Date and Time**
**13/08/2024 17:56:57**

TPS-ISK v2

**Taxon and Assessor details**	
Category	Angiosperms
Taxon name	*Koelreuteria paniculata*
Common name	Goldenrain Tree
Assessor	Božena Mitić
**Risk screening context**	
Reason and socio-economic benefits	honey-bearing, horticulture, landscape design
Risk assessment area	Continental Europe
Taxonomy	Fam. Sapindaceae; a lot of synonyms
Native range	Asia-Temperate (China) and Eastern
Introduced range	USA, Europe, Australia
URL	https://www.gbif.org/species/5421180;

			**Response**	**Justification (references or other information)**	**Confidence**
**A. Biogeography/Historical**					
1. Domestication/Cultivation					
1	1.01	Has the taxon been the subject of domestication?	Yes	https://alienplantsbelgium.myspecies.info/content/koelreuteria-paniculata; Boršić et al. 2016 (2CSIS Zagreb); Ljubojević et al. 2021: J Environ Manag 295: 113102, https://doi.org/10.1016/j.jenvman.2021.113102.; https://www.havlis.cz/karta_en.php?kytkaid=7 etc.	Very high
2	1.02	Is the taxon harvested in the wild and likely to be sold or used in its live form?	Yes	Boršić, Igor ; Kutleša, Petra ; Desnica, Sonja ; Slivar, Sandra Invasive potential of goldenrain tree (Koelreuteria paniculata Laxm., Sapindaceae) - a case study from the City of Zagreb // Zbornik sažetaka 2. Hrvatskog simpozija o invazivnim vrstama s međunarodnim sudjelovanjem / Jelaska, Sven D. (ur.). Zagreb: Hrvatsko ekološko društvo, 2016. p. 31; Ljubojević et al. 2021: J Environ Manag 295: 113102, https://doi.org/10.1016/j.jenvman.2021.113102.; ADVERTISING FOR THE SALE OF SEEDLINGS (numerous websites, e.g. https://www.njuskalo.hr/sjeme-sadnice/koelreuteria-paniculata-sadnice-oglas-26222845;	Very high
3	1.03	Does the taxon have invasive races, varieties, sub-taxa or congeners?	Yes	personal judgement based on: eg. https://edis.ifas.ufl.edu/publication/ST339, https://bie.ala.org.au/species/ALA_DR24112_143 etc.	High
2. Climate, distribution and introduction risk					
4	2.01	How similar are the climatic conditions between the risk assessment area and the taxon's native range?	High	Peel et al. 2007	Very high
5	2.02	What is the quality of the climate matching data?	Medium	Peel et al. 2007	Medium
6	2.03	Is the taxon known to be present outside of cultivation in the risk assessment area?	Yes	https://alienplantsbelgium.myspecies.info/content/koelreuteria-paniculata; Ljubojević et al. 2021: J Environ Manag 295: 113102, https://doi.org/10.1016/j.jenvman.2021.113102.	Very high
7	2.04	How many potential vectors could the taxon use to enter the risk assessment area?	>1	https://www.growmeinstead.com.au/plant/golden-rain-trees-sw.aspx; numerous websites that offer seedlings for sale (e.g. https://kansasplantfarm.com/plants/koelreuteria-paniculata)	Very high
8	2.05	Is the taxon currently found in close proximity to, and likely to enter, the risk assessment area in the near future (e.g. unintentional or intentional introductions)?	Not applicable	https://alienplantsbelgium.myspecies.info/content/koelreuteria-paniculata (van der Meijden & al. 1996; Tison & de Foucault 2014; Stace 2010); Ljubojević et al. 2021: J Environ Manag 295: 113102, https://doi.org/10.1016/j.jenvman.2021.113102.	Very high
3. Invasive elsewhere					
9	3.01	Has the taxon become naturalised outside its native range?	Yes	https://alienplantsbelgium.myspecies.info/content/koelreuteria-paniculata (van der Meijden & al. 1996; Tison & de Foucault 2014; Stace 2010)	Very high
10	3.02	In the taxon's introduced range, are there any known adverse impacts to wild or commercial species?	Yes	https://ballina.nsw.gov.au/files/EnvironmentalWeedAlert-GoldenRainTree.pdf?v=1209825382; Ljubojević et al. 2021: J Environ Manag 295: 113102,	Very high
11	3.03	In the taxon's introduced range, are there any known adverse impacts to agriculture or forestry?	Yes	no exact references found, professional judgement	Low
12	3.04	In the taxon's introduced range, are there any known adverse impacts to ecosystem services?	Yes	Tomić et al 2020, Fuel 277: 118162, https://doi.org/10.1016/j.fuel.2020.118162; Ljubojević et al. 2021: J Environ Manag 295: 113102, https://doi.org/10.1016/j.jenvman.2021.113102.	High
13	3.05	In the taxon's introduced range, are there any known adverse socio-economic impacts?	No	professional judgement	Medium
**B. Biology/Ecology**					
4. Undesirable (or persistence) traits					
14	4.01	Is the taxon likely to be poisonous or pose other risks to human health?	No	no references found for humans; however: a) some references indicate a positive effect on human health (eg. Andonova et al. 2023, Foods 12(11): 2230, doi: 10.3390/foods12112230); b) it is mentioned that it is toxic to some animals	High
15	4.02	Is the taxon likely to suppress the growth of one or more native species?	Yes	professional judgement, no exact references found	Low
16	4.03	Are there any threatened or protected native species that the taxon would parasitise in the risk assessment area?	No	professional opinion, no exact references found	Medium
17	4.04	Is the taxon adaptable in terms of climatic and other environmental conditions, thus enhancing its potential persistence if it has invaded or is likely to invade the risk	Yes	Stojanović and Jovanović 2018, Zaštita prirode 68/1-2: 41-59; Boršić et al. 2016, 2CSIS Zagreb, Book of Abstracts, pp31 etc., https://www.gbif.org/species/152543345 etc.	Very high

18	4.05	Is the taxon likely to disrupt terrestrial ecosystem function if it has invaded or is likely to invade the risk assessment area?	No	no such references found	Low
19	4.06	Is the taxon likely to exert adverse impacts on ecosystem services in the risk assessment area?	Yes	Tomić et al 2020, Fuel 277: 118162, https://doi.org/10.1016/j.fuel.2020.118162; Ljubojević et al. 2022, JEM 295: 113102,	Medium
20	4.07	Is the taxon likely to host or function as a vector for recognised pests and pathogens that are present in the risk assessment area?	Yes	Pan et al. 2023, Mycology 14(1): 37–51, doi:10.1080/21501203.2022.2096144; https://edis.ifas.ufl.edu/publication/ST339; https://hort.ifas.ufl.edu/database/documents/pdf/tree_fact_sheets/koepana.pdf	Very high
21	4.08	Is the taxon likely to host or function as a vector for recognised pests and pathogens that are absent in the risk assessment area?	No	No references found, professional judgement	Low
22	4.09	Is the taxon likely to be released from cultivation?	Yes	https://www.gbif.org/species/143605093; https://alienplantsbelgium.myspecies.info/content/koelreuteria-paniculata	High
23	4.10	Is the taxon versatile in habitat use?	Yes	https://alienplantsbelgium.myspecies.info/content/koelreuteria-paniculata	Very high
24	4.11	Is it likely that the taxon's mode of existence or behaviours will reduce habitat quality for native species?	Yes	https://galvbayinvasives.org/invasive/P-045	Medium
25	4.12	Is the taxon likely to maintain a viable population even when present in low densities (or persist in adverse conditions by way of a dormant form)?	Yes	https://galvbayinvasives.org/invasive/P-045; https://bie.ala.org.au/species/ALA_DR24112_143; https://alienplantsbelgium.myspecies.info/content/koelreuteria-paniculata	Very high
5. Resource exploitation					
26	5.01	Is the taxon likely to put threatened or protected native species under competitive pressure in the risk assessment area?	No	professional judgement	Medium
27	5.02	Is the taxon likely to sequester resources to the detriment of native species in the risk assessment area?	Yes	professional judgement (estimated according to https://alienplantsbelgium.myspecies.info/content/koelreuteria-paniculata)	Low
6. Reproduction					
28	6.01	Is the taxon likely to exhibit changes in reproductive strategy in response to environmental conditions?	Yes	https://alienplantsbelgium.myspecies.info/content/koelreuteria-paniculata; https://bie.ala.org.au/species/ALA_DR24112_143; https://edis.ifas.ufl.edu/publication/ST339	High
29	6.02	Is the taxon likely to produce viable propagules in the risk assessment area?	Yes	Ljubojević et al. 2021: J Environ Manag 295: 113102, https://doi.org/10.1016/j.jenvman.2021.113102.; https://alienplantsbelgium.myspecies.info/content/koelreuteria-paniculata	High
30	6.03	Is the taxon likely to hybridise with native species under natural conditions?	No	No data found	Low
31	6.04	Is the taxon likely to be hermaphroditic or to exhibit asexual reproduction?	Yes	https://www.treesandshrubsonline.org/articles/koelreuteria/koelreuteria-paniculata/;	Very high
32	6.05	Is the taxon dependent on the presence of another species (or specific habitat features) to complete its life cycle?	No	Personal judgement (no references found)	Medium
33	6.06	Is the taxon likely to produce a large number of propagules?	Yes	https://www.treesandshrubsonline.org/articles/koelreuteria/koelreuteria-paniculata/; https://bie.ala.org.au/species/ALA_DR24112_143; Ljubojević et al. 2021: J Environ Manag 295: 113102, https://doi.org/10.1016/j.jenvman.2021.113102.; Idžojtić M. (2013): DENDROLOGIJA – cvijet, češer, plod, sjeme. Denona d.d.	Very high
34	6.07	How many time units (days, months, years) does the taxon require to reach the age at first reproduction?	3	No exact data found, this is my professional judgement based on https://plants.ces.ncsu.edu/plants/koelreuteria-paniculata/; https://www.housedigest.com/845025/how-to-successfully-grow-a-golden-rain-tree/ and Idžojtić M. (2013): DENDROLOGIJA – cvijet, češer, plod, sjeme. Denona d.d. (In Croatian).	Medium
7. Dispersal mechanisms					
35	7.01	How many potential vectors or pathways could the taxon use to disperse within the risk assessment area (with suitable habitats nearby)?	>1	https://www.growmeinstead.com.au/plant/golden-rain-trees-sw.aspx (wind, water, humans,)	Very high
36	7.02	Will any of these vectors or pathways bring the taxon in close proximity to one or more protected areas?	Yes	Personal judgement based on different references (e.g. Ljubojević et al. 2021: J Environ Manag 295: 113102, https://doi.org/10.1016/j.jenvman.2021.113102 - data for Novi Sad close to some protected areas)	High
37	7.03	Does the taxon have a specialised means of attachment such that it enhances the likelihood of dispersal?	Yes	inflated bladderlike pod (e.g. https://bie.ala.org.au/species/ALA_DR24112_143)	Very high
38	7.04	Is natural dispersal of the taxon likely to occur as seeds or spores in the risk assessment area?	Yes	Ljubojević et al. 2021: J Environ Manag 295: 113102, https://doi.org/10.1016/j.jenvman.2021.113102.; https://alienplantsbelgium.myspecies.info/content/koelreuteria-paniculata	Very high
39	7.05	Is natural dispersal of the taxon likely to occur as fragments or seedlings in the risk assessment area?	Yes	Personal judgement based on https://alienplantsbelgium.myspecies.info/content/koelreuteria-paniculata; Ljubojević et al. 2021: J Environ Manag 295: 113102, https://doi.org/10.1016/j.jenvman.2021.113102.	High
40	7.06	Are any life stages of the taxon likely to migrate into the risk assessment area for reproduction?	Yes	personal judgement based on https://alienplantsbelgium.myspecies.info/content/koelreuteria-paniculata, Ljubojević et al. 2021: J Environ Manag 295: 113102, https://doi.org/10.1016/j.jenvman.2021.113102.	High

41	7.07	Are propagules of the taxon likely to be dispersed in the risk assessment area by other species?	No	No data, personal judgement	Medium
42	7.08	Is dispersal of the taxon along any of the vectors or pathways mentioned in the previous seven Questions (35–41: i.e. either unintentional or intentional) likely to be	No	Personal judgement	Medium
43	7.09	Is dispersal of the taxon density dependent?	Yes	https://alienplantsbelgium.myspecies.info/content/koelreuteria-paniculata	Very high
8. Tolerance attributes					
44	8.01	Is the taxon able to withstand being in water for extended periods at some stage of its life cycle?	Yes	Personal judgement based on https://www.growmeinstead.com.au/plant/golden-rain-trees-sw.aspx	Medium
45	8.02	Is the taxon tolerant of a wide range of soil or air quality conditions?	Yes	https://www.urbanjungle.uk.com/product/koelreuteria-paniculata-2/; https://www.havlis.cz/karta_en.php?kytkaid=7; https://bie.ala.org.au/species/ALA_DR24112_143 etc.; https://www.blockhill.co.nz/golden_rain_tree130338	Very high
46	8.03	Can the taxon be controlled or eradicated in the wild with chemical, biological or other agents and means?	Yes	https://ballina.nsw.gov.au/files/EnvironmentalWeedAlert-GoldenRainTree.pdf?v=1209825382; https://weeds.org.au/profiles/chinese-rain-tree/	Very high
47	8.04	Is the taxon likely to benefit from environmental or human disturbance?	Yes	https://weeds.org.au/profiles/chinese-rain-tree/	Very high
48	8.05	Is the taxon able to tolerate soil acidity, salinity or other parameter levels that are higher or lower than those found in its usual	Yes	https://weeds.org.au/profiles/chinese-rain-tree/; https://www.blockhill.co.nz/golden_rain_tree130338	Very high
49	8.06	Are there effective natural enemies of the taxon present in the risk assessment area?	No	Ljubojević et al. 2021: J Environ Manag 295: 113102, https://doi.org/10.1016/j.jenvman.2021.113102.	Medium
**C. Climate change**					
9. Climate change					
50	9.01	Under the predicted future climatic conditions, are the risks of entry into the risk assessment area posed by the taxon likely to increase, decrease or not change?	Increase	Professional judgement based on https://alienplantsbelgium.myspecies.info/content/koelreuteria-paniculata	Medium
51	9.02	Under the predicted future climatic conditions, are the risks of establishment within the risk assessment area posed by the taxon likely to increase, decrease or not	Increase	Professional judgement based on https://alienplantsbelgium.myspecies.info/content/koelreuteria-paniculata	Medium
52	9.03	Under the predicted future climatic conditions, are the risks of dispersal within the risk assessment area posed by the taxon likely to increase, decrease or not change?	Increase	Professional judgement based on https://alienplantsbelgium.myspecies.info/content/koelreuteria-paniculata	Medium
53	9.04	Under the predicted future climatic conditions, what is the likely magnitude of future potential impacts by the taxon on biodiversity or ecosystem integrity and	Higher	Professional judgement based on https://alienplantsbelgium.myspecies.info/content/koelreuteria-paniculata	Medium
54	9.05	Under the predicted future climatic conditions, what is the likely magnitude of future potential impacts by the taxon on ecosystem structure or function?	No change	Professional judgement	Medium
55	9.06	Under the predicted future climatic conditions, what is the likely magnitude of future potential impacts by the taxon on ecosystem services and related socio-	No change	Professional judgement	Medium

**Statistics**	
**Scores**	
**BRA**	**47.0**
**BRA Outcome**	**High**
**BRA+CCA**	**55.0**
**BRA+CCA Outcome**	**High**
**Score partition**	
**A. Biogeography/Historical**	**20.0**
1. Domestication/Cultivation	*4.0*
2. Climate, distribution and introduction risk	*2.0*
3. Invasive elsewhere	*14.0*
**B. Biology/Ecology**	**27.0**
4. Undesirable (or persistence) traits	*8.0*
5. Resource exploitation	*2.0*
6. Reproduction	*4.0*
7. Dispersal mechanisms	*6.0*
8. Tolerance attributes	*7.0*
**C. Climate change**	**8.0**
9. Climate change	*8.0*
**Answered Questions**	
Total	***55***
**A. Biogeography/Historical**	**13**
1. Domestication/Cultivation	*3*
2. Climate, distribution and introduction risk	*5*
3. Invasive elsewhere	*5*
**B. Biology/Ecology**	**36**
4. Undesirable (or persistence) traits	*12*
5. Resource exploitation	*2*
6. Reproduction	*7*
7. Dispersal mechanisms	*9*
8. Tolerance attributes	*6*

**C. Climate change**	**6**
9. Climate change	*6*
**Sectors affected**	
**Commercial**	**14**
**Environmental**	**9**
**Species or population nuisance traits**	**36**

**Thresholds**	
**BRA**	**41**
**BRA+CCA**	**41**
**Confidence**	
**BRA+CCA**	**0.71**
**BRA**	**0.74**
**CCA**	**0.50**

**Date and Time**
**14/08/2024 11:43:22**

TPS-ISK v2

Taxon and Assessor details	
Category	Angiosperms
Taxon name	*Liquidambar styraciflua*
Common name	American Storax
Assessor	Mihaela Britvec
**Risk screening context**	
Reason and socio-economic benefits	
Risk assessment area	Continental Europe
Taxonomy	
Native range	
Introduced range	
URL	

			Response	Justification (references or other information)	Confidence
**A. Biogeography/Historical**					
1. Domestication/Cultivation					
1	1.01	Has the taxon been the subject of domestication?	Yes	Liquidambar styraciflua is native to warm temperate areas of eastern North America and tropical montane regions of Mexico and Central America. It is popular ornamental and forestal tree in temperate climates. The species was introduced into Europe in	Very high
2	1.02	Is the taxon harvested in the wild and likely to be sold or used in its live form?	Yes	Liquidambar styraciflua is well known ornamental tree. Its wood and resin is also used (Kormanik 1990).	Very high
3	1.03	Does the taxon have invasive races, varieties, sub-taxa or congeners?	No	no evidence	Medium
2. Climate, distribution and introduction risk					
4	2.01	How similar are the climatic conditions between the risk assessment area and the taxon's native range?	High	Liquidambar styraciflua grows in temperate climates (also in continental Europe).	High
5	2.02	What is the quality of the climate matching data?	High	professional judgement	Medium
6	2.03	Is the taxon known to be present outside of cultivation in the risk assessment area?	No	This species is known only in cultivation.	Low
7	2.04	How many potential vectors could the taxon use to enter the risk assessment area?	>1	humans, wind, animals	Medium
8	2.05	Is the taxon currently found in close proximity to, and likely to enter, the risk assessment area in the near future (e.g. unintentional or intentional introductions)?	No	no evidence	Medium
3. Invasive elsewhere					
9	3.01	Has the taxon become naturalised outside its native range?	No	no evidence	Low
10	3.02	In the taxon's introduced range, are there any known adverse impacts to wild or commercial species?	No	no evidence	Low
11	3.03	In the taxon's introduced range, are there any known adverse impacts to agriculture or forestry?	No	no evidence	Low
12	3.04	In the taxon's introduced range, are there any known adverse impacts to ecosystem	No	no evidence	Low
13	3.05	In the taxon's introduced range, are there any known adverse socio-economic impacts?	No	no evidence	Low
**B. Biology/Ecology**					
4. Undesirable (or persistence) traits					
14	4.01	Is the taxon likely to be poisonous or pose other risks to human health?	Yes	Liquidambar styraciflua is a mild allergen (https://www.pollenlibrary.com/Specie/Liquidambar+styraciflua/).	Medium
15	4.02	Is the taxon likely to suppress the growth of one or more native species?	No	Liquidambar styraciflua associates with other tree species without displacing them.	Low
16	4.03	Are there any threatened or protected native species that the taxon would parasitise in the risk assessment area?	No	It is not known that Liquidambar styraciflua is a parasite.	Medium
17	4.04	Is the taxon adaptable in terms of climatic and other environmental conditions, thus enhancing its potential persistence if it has invaded or is likely to invade the risk	Yes	Sweetgum is perhaps one of the most adaptable hardwood species in its tolerance to different soil and site conditions (Kormanik 1990).	High
18	4.05	Is the taxon likely to disrupt terrestrial ecosystem function if it has invaded or is likely to invade the risk assessment area?	No	no evidence	Low
19	4.06	Is the taxon likely to exert adverse impacts on ecosystem services in the risk assessment area?	Yes	For example, it should be careful when locating this species as a street tree since its large, aggressive roots may lift curbs and sidewalks (https://edis.ifas.ufl.edu/publication/ST358).	Low
20	4.07	Is the taxon likely to host or function as a vector for recognised pests and pathogens that are present in the risk assessment area?	Yes	This tree is relatively pest-free but may be bothered by webworms. Bacterial scorch is a fatal and potentially serious disease of trees suffering from physiological stresses (https://www.uky.edu/hort/Sweetgum).	Low
21	4.08	Is the taxon likely to host or function as a vector for recognised pests and pathogens that are absent in the risk assessment area?	Yes	Liquidambar styraciflua is relatively pest-free but may be bothered by webworms. Bacterial scorch is a fatal and potentially serious disease of trees suffering from physiological stresses (https://www.uky.edu/hort/Sweetgum).	Low
22	4.09	Is the taxon likely to be released from cultivation?	Yes	Liquidambar styraciflua grows fast. Its seeds are lightweight and have a small wing that allows them to be carried by the wind. Because of this, sweetgum is often seen as a pioneer species on flood plains or in abandoned fields (Tauer and Tauer 2011, https://www.uky.edu/hort/Sweetgum).	Medium

23	4.10	Is the taxon versatile in habitat use?	Yes	Liquidambar styraciflua is tolerant to different site conditions (Kormanik 1990).	High
24	4.11	Is it likely that the taxon's mode of existence or behaviours will reduce habitat quality for native species?	No	no evidence	Low
25	4.12	Is the taxon likely to maintain a viable population even when present in low densities (or persist in adverse conditions by way of a dormant form)?	Yes	The importance of root sprout formation with American Storax regeneration is evident from observations made in natural stands of mixed pines and hardwoods in the Georgia Piedmont that have been logged for sawtimber (Kormanik 1990).	High
5. Resource exploitation					
26	5.01	Is the taxon likely to put threatened or protected native species under competitive pressure in the risk assessment area?	No	no evidence	Low
27	5.02	Is the taxon likely to sequester resources to the detriment of native species in the risk assessment area?	No	no evidence	Low
6. Reproduction					
28	6.01	Is the taxon likely to exhibit changes in reproductive strategy in response to environmental conditions?	Yes	professional judgement	Low
29	6.02	Is the taxon likely to produce viable propagules in the risk assessment area?	Yes	doi: 10.1007/s00468-010-0418-9	Very high
30	6.03	Is the taxon likely to hybridise with native species under natural conditions?	No	No hybrids of American Storax are known to exist (Kormanik 1990).	Medium
31	6.04	Is the taxon likely to be hermaphroditic or to exhibit asexual reproduction?	Yes	It exist the tendency of sweetgum to regenerate from root sprouts that originated from suppressed root buds (Kormanik 1990).	High
32	6.05	Is the taxon dependent on the presence of another species (or specific habitat features) to complete its life cycle?	No	no evidence	Medium
33	6.06	Is the taxon likely to produce a large number of propagules?	No	no evidence	Medium
34	6.07	How many time units (days, months, years) does the taxon require to reach the age at first reproduction?	>10	Sweetgum trees produce viable seed crops when they reach 20 to 30 years of age (Tauer and Tauer 2011).	Very high
7. Dispersal mechanisms					
35	7.01	How many potential vectors or pathways could the taxon use to disperse within the risk assessment area (with suitable habitats nearby)?	>1	dispersal by humans, by wind and by animals	High
36	7.02	Will any of these vectors or pathways bring the taxon in close proximity to one or more protected areas?	Yes	The seeds are lightweight and have a small wing that allows them to be carried by the wind (https://www.uky.edu/hort/Sweetgum).The maximum dispersal distance is approximately 183 m, but American Storax seeds move an average of 61 m from the point of release (Fowells 1965).	Medium
37	7.03	Does the taxon have a specialised means of attachment such that it enhances the likelihood of dispersal?	No	no evidence	Medium
38	7.04	Is natural dispersal of the taxon likely to occur as seeds or spores in the risk assessment area?	Yes	The small winged seeds, one or two per capsule, are readily disseminated by wind (Kormanik 1990). Goldfinches, purple finches, squirrels, and chipmunks eat the seeds (https://en.wikipedia.org/wiki/Liquidambar_styraciflua#Fruit).	Very high
39	7.05	Is natural dispersal of the taxon likely to occur as fragments or seedlings in the risk assessment area?	Yes	The seedlings are often grown in nurseries.	Medium
40	7.06	Are any life stages of the taxon likely to migrate into the risk assessment area for reproduction?	Yes	The American Storax seeds move an average of 61 m from the point of release (Fowells 1965).	Very high
41	7.07	Are propagules of the taxon likely to be dispersed in the risk assessment area by other species?	Yes	Goldfinches, purple finches, and squirrels, eat the seeds (https://en.wikipedia.org/wiki/Liquidambar_styraciflua#Fruit).	High
42	7.08	Is dispersal of the taxon along any of the vectors or pathways mentioned in the previous seven Questions (35–41: i.e. either unintentional or intentional) likely to be	Yes	professional judgement	Medium
43	7.09	Is dispersal of the taxon density dependent?	No	no evidence	Low
8. Tolerance attributes					
44	8.01	Is the taxon able to withstand being in water for extended periods at some stage of its life cycle?	Yes	The seed can be stored in a water bath for 14 to 20 days at a constant 3 to 5 °C (Tauer and Tauer 2011).	Medium
45	8.02	Is the taxon tolerant of a wide range of soil or air quality conditions?	Yes	American Storax is tolerant to different soil conditions (Kormanik 1990).	High
46	8.03	Can the taxon be controlled or eradicated in the wild with chemical, biological or other agents and means?	Yes	doi: 10.1093/forsci/fxaa039	Medium
47	8.04	Is the taxon likely to benefit from environmental or human disturbance?	Yes	Liquidambar styraciflua is a common pioneer species. It does well on many soil typesis. This tree is excellent for restoration projects (https://mellowmarshfarm.com/catalog/liquidambar-styraciflua/).	Medium
48	8.05	Is the taxon able to tolerate soil acidity, salinity or other parameter levels that are higher or lower than those found in its usual	Yes	Can tolerate acid to neutral soil: pH 5.0 to 7.4 (https://woodyplants.cals.cornell.edu/plant/138).	High
49	8.06	Are there effective natural enemies of the taxon present in the risk assessment area?	No	no reference	Low
**C. Climate change**					
9. Climate change					

50	9.01	Under the predicted future climatic conditions, are the risks of entry into the risk assessment area posed by the taxon likely to increase, decrease or not change?	Increase	professional judgement	Low
51	9.02	Under the predicted future climatic conditions, are the risks of establishment within the risk assessment area posed by the taxon likely to increase, decrease or not	No change	professional judgement	Low
52	9.03	Under the predicted future climatic conditions, are the risks of dispersal within the risk assessment area posed by the taxon likely to increase, decrease or not change?	No change	professional judgement	Low
53	9.04	Under the predicted future climatic conditions, what is the likely magnitude of future potential impacts by the taxon on biodiversity or ecosystem integrity and	No change	professional judgement	Low
54	9.05	Under the predicted future climatic conditions, what is the likely magnitude of future potential impacts by the taxon on ecosystem structure or function?	No change	professional judgement	Low
55	9.06	Under the predicted future climatic conditions, what is the likely magnitude of future potential impacts by the taxon on ecosystem services and related socio-	No change	professional judgement	Low

Statistics	
**Scores**	
**BRA**	**26.0**
**BRA Outcome**	**Medium**
**BRA+CCA**	**28.0**
**BRA+CCA Outcome**	**Medium**
**Score partition**	
**A. Biogeography/Historical**	**3.0**
1. Domestication/Cultivation	*2.0*
2. Climate, distribution and introduction risk	*1.0*
3. Invasive elsewhere	*0.0*
**B. Biology/Ecology**	**23.0**
4. Undesirable (or persistence) traits	*8.0*
5. Resource exploitation	*0.0*
6. Reproduction	*2.0*
7. Dispersal mechanisms	*6.0*
8. Tolerance attributes	*7.0*
**C. Climate change**	**2.0**
9. Climate change	*2.0*
**Answered Questions**	
Total	***55***
**A. Biogeography/Historical**	**13**
1. Domestication/Cultivation	*3*
2. Climate, distribution and introduction risk	*5*
3. Invasive elsewhere	*5*
**B. Biology/Ecology**	**36**
4. Undesirable (or persistence) traits	*12*
5. Resource exploitation	*2*
6. Reproduction	*7*
7. Dispersal mechanisms	*9*
8. Tolerance attributes	*6*
**C. Climate change**	**6**
9. Climate change	*6*
**Sectors affected**	
**Commercial**	**6**
**Environmental**	**2**
**Species or population nuisance traits**	**24**

Thresholds	
**BRA**	**41**
**BRA+CCA**	**41**
**Confidence**	
**BRA+CCA**	**0.49**
**BRA**	**0.52**
**CCA**	**0.25**

Date and Time
**25/06/2024 11:33:14**

TPS-ISK v2

**Taxon and Assessor details**	
Category	Angiosperms
Taxon name	*Liriodendron tulipifera*
Common name	Tulip Tree
Assessor	Ivica Ljubičić
**Risk screening context**	
Reason and socio-economic benefits	
Risk assessment area	Continental Europe
Taxonomy	
Native range	
Introduced range	
URL	

			Response	Justification (references or other information)	Confidence
**A. Biogeography/Historical**					
1. Domestication/Cultivation					
1	1.01	Has the taxon been the subject of domestication?	Yes	Beck, D. E., & Hooper, R. M. (1986). Development of a southern Appalachian hardwood stand after clearcutting. Southern Journal of Applied Forestry, 10(3), 168–172. https://doi.org/10.1093/sjaf/10.3.168	High
2	1.02	Is the taxon harvested in the wild and likely to be sold or used in its live form?	Yes	Moerman, D. E. (1998). Native American Ethnobotany. Timber Press.	High
3	1.03	Does the taxon have invasive races, varieties, sub-taxa or congeners?	No	Liriodendron tulipifera is not characterized as an invasive species. In its natural range in the eastern part of North America, it is an autochthonous species and is part of natural forest ecosystems. In some areas where it has been introduced as an ornamental tree, it may exhibit characteristics similar to invasive species, such as rapid growth and the ability to spread beyond a controlled area.	Medium
2. Climate, distribution and introduction risk					
4	2.01	How similar are the climatic conditions between the risk assessment area and the taxon's native range?	High	According to the model of Venette et al. (2010), climatic conditions in many parts of continental Europe are similar enough to those in eastern North America to allow the successful growth of Liriodendron tulipifera. That is why the tulip tree can be successfully grown in many parts of Europe, where it is often used as an ornamental tree in parks and gardens.	Medium
5	2.02	What is the quality of the climate matching data?	High	https://www.eea.europa.eu/data-and-maps/data-providers-and-partners/worldclim-global-climate-data	Medium
6	2.03	Is the taxon known to be present outside of cultivation in the risk assessment area?	Yes	Liriodendron tulipifera has been present in continental Europe since the 17th century. https://www.gbif.org/species/3152861	High
7	2.04	How many potential vectors could the taxon use to enter the risk assessment area?	>1	It reached continental Europe through a combination of historical, botanical, horticultural and trade activities. Over the centuries, various vectors have allowed the spread of this species from its natural habitat in eastern North America to many European countries, where it is now successfully cultivated and used in a	Medium
8	2.05	Is the taxon currently found in close proximity to, and likely to enter, the risk assessment area in the near future (e.g. unintentional or intentional introductions)?	Not applicable	Liriodendron tulipifera L. in GBIF Secretariat (2023). GBIF Backbone Taxonomy. Checklist dataset https://doi.org/10.15468/39omei accessed via GBIF.org on 2024-06-11.	High
3. Invasive elsewhere					
9	3.01	Has the taxon become naturalised outside its native range?	Yes	Checklist dataset https://doi.org/10.15468/39omei accessed via GBIF.org on 2024-06-11.	High
10	3.02	In the taxon's introduced range, are there any known adverse impacts to wild or commercial species?	No	It is not an invasive species, although as with any species, it is important to monitor its interaction with the environment and other plant species to ensure the preservation of ecosystems and biodiversity.	Medium
11	3.03	In the taxon's introduced range, are there any known adverse impacts to agriculture or forestry?	No	It is grown mainly in gardens, parks and forests, not in agricultural areas.	Medium
12	3.04	In the taxon's introduced range, are there any known adverse impacts to ecosystem	No	professional judgement	Low
13	3.05	In the taxon's introduced range, are there any known adverse socio-economic impacts?	No	professional judgement	Low
**B. Biology/Ecology**					
4. Undesirable (or persistence) traits					
14	4.01	Is the taxon likely to be poisonous or pose other risks to human health?	No	It is safe for most people, but some people may have allergic reactions to the leaves or flowers of the plant	Low
15	4.02	Is the taxon likely to suppress the growth of one or more native species?	Not applicable	It is not known as an invasive species. However, in certain conditions or areas where it has been introduced or where it is grown in dense plantations, there is a possibility that it may have some impact on native plant species.	Low
16	4.03	Are there any threatened or protected native species that the taxon would parasitise in the risk assessment area?	No	professional judgement	Medium
17	4.04	Is the taxon adaptable in terms of climatic and other environmental conditions, thus enhancing its potential persistence if it has invaded or is likely to invade the risk assessment area?	Yes	It grows in a variety of climate zones in eastern North America, suggesting that it has a wide range of climate tolerance. It is known for its rapid growth and development, which may contribute to its ability to adapt to changes in the environment, including changes in temperature, rainfall and other climatic	Medium
18	4.05	Is the taxon likely to disrupt terrestrial ecosystem function if it has invaded or is likely to invade the risk assessment area?	No	professional judgement	Medium
19	4.06	Is the taxon likely to exert adverse impacts on ecosystem services in the risk	No	professional judgement	Medium

20	4.07	Is the taxon likely to host or function as a vector for recognised pests and pathogens that are present in the risk assessment area?	No	no reference	Low
21	4.08	Is the taxon likely to host or function as a vector for recognised pests and pathogens that are absent in the risk assessment area?	Yes	Masten Milek, Tatjana ; Seljak, Gabrijel ; Pintar, Maja ; Šimala, Mladen Lisna uš lagerstremije [(Sarucallis kahawaluokalani (Kirkaldy 1906)] i lisna uš liriodendrona [(Illinoia liriodendri (Monell 1879)] – štetnici u urbanim sredinama // Glasilo biljne	Medium
22	4.09	Is the taxon likely to be released from cultivation?	Yes	Liriodendron tulipifera L. in GBIF Secretariat (2023). GBIF Backbone Taxonomy. Checklist dataset https://doi.org/10.15468/39omei accessed via GBIF.org on 2024-	High
23	4.10	Is the taxon versatile in habitat use?	Yes	professional judgement	Low
24	4.11	Is it likely that the taxon's mode of existence or behaviours will reduce habitat quality for native species?	Not applicable	no reference	Medium
25	4.12	Is the taxon likely to maintain a viable population even when present in low densities (or persist in adverse conditions by way of a dormant form)?	Yes	Ellen Powell, (2007.), Common native trees of Virginia: tree identification guide, Virginia: Virginia Department of Forestry	Medium
5. Resource exploitation					
26	5.01	Is the taxon likely to put threatened or protected native species under competitive pressure in the risk assessment area?	No	In most European countries, tulips are grown in controlled conditions such as parks and botanical gardens. In these environments, the risk of suppressing protected species is minimal because the plants are managed and their growth monitored.	Low
27	5.02	Is the taxon likely to sequester resources to the detriment of native species in the risk assessment area?	Not applicable	no reference	Low
6. Reproduction					
28	6.01	Is the taxon likely to exhibit changes in reproductive strategy in response to environmental conditions?	Not applicable	no reference	Low
29	6.02	Is the taxon likely to produce viable propagules in the risk assessment area?	Yes	It has significant potential for the production of viable propagules due to its ability to produce large numbers of seeds with high germination and its ability to spread by wind.	Low
30	6.03	Is the taxon likely to hybridise with native species under natural conditions?	Yes	https://doi.org/10.1094/PDIS-06-21-1266-RE	Medium
31	6.04	Is the taxon likely to be hermaphroditic or to exhibit asexual reproduction?	Yes	Liriodendron tulipifera is a hermaphroditic plant, which enables it to self-pollinate. It can also reproduce vegetatively through shoots from the root or stump after the tree has been cut down or	High
32	6.05	Is the taxon dependent on the presence of another species (or specific habitat features) to complete its life cycle?	No	professional judgement	Low
33	6.06	Is the taxon likely to produce a large number of propagules?	Yes	Liriodendron tulipifera has significant potential for the production of a large number of propagules, thanks to the development of a large number of flowers and seeds and high seed germination.	Medium
34	6.07	How many time units (days, months, years) does the taxon require to reach the age at first reproduction?	7	Liriodendron tulipifera usually reaches sexual maturity between 15 and 20 years, depending on growing conditions and genetic factors. After reaching sexual maturity, the tree can produce flowers and seeds for a long time, allowing efficient reproduction and spread of the species.	High
7. Dispersal mechanisms					
35	7.01	How many potential vectors or pathways could the taxon use to disperse within the risk assessment area (with suitable habitats nearby)?	>1	Natural vectors (wind, water, animals), anthropogenic (horticulture, transport) and natural processes (erosion). 1. Wind: produces winged seeds that are easily spread by the wind. This natural vector allows seeds to travel considerable distances from the parent tree, increasing the possibility of colonizing new areas. 2. Water: Seeds can be carried by water, especially along rivers and streams. This vector can aid in seed dispersal downstream, allowing the plant to establish in new, suitable habitats along watercourses. 3. People (Anthropogenic Factors): Horticulture and Landscape Architecture. It is often planted in parks, gardens and urban areas for its aesthetic value. Through horticultural activities, seeds or seedlings can be transferred to new areas. 3.1. Transport and Trade: Tulip seeds and seedlings can be inadvertently transferred through transport and plant trade. This includes cases where seeds contaminate agricultural products or equipment transported between regions. 4. Animals: Although not a primary vector, some animals can carry tulip seeds. For example, seeds can be attached to the fur of mammals, or they can be transferred to birds that consume the seeds and later drop them in new locations. 5. Erosion and Natural Processes: Natural processes such as soil erosion can also play a role in moving tulip seeds. This is especially important in areas with heavy rainfall or	Medium
36	7.02	Will any of these vectors or pathways bring the taxon in close proximity to one or more protected areas?	Yes	Wind, water, anthropogenic factors and animal activities can bring Liriodendron tulipifera into the immediate vicinity of the protected area.	Low
37	7.03	Does the taxon have a specialised means of attachment such that it enhances the likelihood of dispersal?	No	Liriodendron tulipifera does not have special seed attachment mechanisms like some other plants that have spines to help them attach to animal fur or soil. Instead, the tulip tree relies on natural mechanisms of propagation through the seeds it produces.	Medium
38	7.04	Is natural dispersal of the taxon likely to occur as seeds or spores in the risk assessment area?	Yes	The likelihood of a species spreading by seed via humans, animals, or other media can vary depending on a variety of factors. Anthropogenic factors such as horticulture, transport and plant trade often have a major impact on the spread of this species, but natural vectors such as wind and water can also play a significant role, especially in conditions where natural habitats	Low

39	7.05	Is natural dispersal of the taxon likely to occur as fragments or seedlings in the risk assessment area?	Yes	https://www.dirtonmyhands.com/growing-tulip-poplar.html	Low
40	7.06	Are any life stages of the taxon likely to migrate into the risk assessment area for reproduction?	Yes	https://www.dirtonmyhands.com/growing-tulip-poplar.html	Low
41	7.07	Are propagules of the taxon likely to be dispersed in the risk assessment area by other species?	No	no reference	Medium
42	7.08	Is dispersal of the taxon along any of the vectors or pathways mentioned in the previous seven Questions (35–41: i.e. either unintentional or intentional) likely to be	Yes	no reference	Low
43	7.09	Is dispersal of the taxon density dependent?	Yes	professional judgement	Low
8. Tolerance attributes					
44	8.01	Is the taxon able to withstand being in water for extended periods at some stage of its life cycle?	No	Although it cannot survive long-term immersion in water, it can withstand temporary exposure to water, such as occasional flooding or high soil moisture levels.	Low
45	8.02	Is the taxon tolerant of a wide range of soil or air quality conditions?	No	no reference	Low
46	8.03	Can the taxon be controlled or eradicated in the wild with chemical, biological or other agents and means?	Yes	professional judgement	Low
47	8.04	Is the taxon likely to benefit from environmental or human disturbance?	Yes	Deforestation results in the creation of open habitats or marginal areas that can be favorable for tulip tree growth. This species prefers bright, sunny conditions, so the creation of new open areas can provide an opportunity for its population to expand.	Low
48	8.05	Is the taxon able to tolerate soil acidity, salinity or other parameter levels that are higher or lower than those found in its usual environment?	Yes	It is an adaptable species that can tolerate certain minor variations in environmental parameters, but its growth and success will depend on various factors such as the availability of water, nutrients, light and competition with other plants.	Low
49	8.06	Are there effective natural enemies of the taxon present in the risk assessment area?	Yes	Liriodendron tulipifera has several natural enemies and pathogens that can effectively affect its populations. These natural enemies include insects, fungal diseases and other pathogens. https://entnemdept.ufl.edu/creatures/orn/scales/tuliptree_scale.htm, Burns DP, Donley DE. 1970. Biology of the tuliptree scale, Toumeyella liriodendra (Homoptera: Coccidae). Annals of the Entomological Society of America 63: 228-235.	Medium
**C. Climate change**					
9. Climate change					
50	9.01	Under the predicted future climatic conditions, are the risks of entry into the risk assessment area posed by the taxon likely to increase, decrease or not change?	Increase	professional judgement	Low
51	9.02	Under the predicted future climatic conditions, are the risks of establishment within the risk assessment area posed by the taxon likely to increase, decrease or not	Increase	https://www.fs.usda.gov/nrs/atlas/tree/621	Medium
52	9.03	Under the predicted future climatic conditions, are the risks of dispersal within the risk assessment area posed by the taxon likely to increase, decrease or not change?	Increase	Climate change brings a general increase in temperatures, which could allow Liriodendron tulipifera to spread to more northern parts of Europe, where it is currently restricted due to colder winter conditions. It prefers areas with moderate to high rainfall. If climate change leads to changes in rainfall patterns, areas that become drier may become less suitable for this species, while wetter areas may become more suitable.	Medium
53	9.04	Under the predicted future climatic conditions, what is the likely magnitude of future potential impacts by the taxon on biodiversity or ecosystem integrity and	Higher	professional judgement	Low
54	9.05	Under the predicted future climatic conditions, what is the likely magnitude of future potential impacts by the taxon on ecosystem structure or function?	Higher	professional judgement	Low
55	9.06	Under the predicted future climatic conditions, what is the likely magnitude of future potential impacts by the taxon on ecosystem services and related socio-	Higher	professional judgement	Low

**Statistics**	
**Scores**	
**BRA**	**21.0**
**BRA Outcome**	**Medium**
**BRA+CCA**	**33.0**
**BRA+CCA Outcome**	**Medium**
**Score partition**	
**A. Biogeography/Historical**	**6.0**
1. Domestication/Cultivation	*2.0*
2. Climate, distribution and introduction risk	*2.0*
3. Invasive elsewhere	*2.0*
**B. Biology/Ecology**	**15.0**
4. Undesirable (or persistence) traits	*5.0*
5. Resource exploitation	*0.0*
6. Reproduction	*4.0*
7. Dispersal mechanisms	*5.0*
8. Tolerance attributes	*1.0*

**C. Climate change**	**12.0**
9. Climate change	*12.0*
**Answered Questions**	
Total	***55***
**A. Biogeography/Historical**	**13**
1. Domestication/Cultivation	*3*
2. Climate, distribution and introduction risk	*5*
3. Invasive elsewhere	*5*
**B. Biology/Ecology**	**36**
4. Undesirable (or persistence) traits	*12*
5. Resource exploitation	*2*
6. Reproduction	*7*
7. Dispersal mechanisms	*9*
8. Tolerance attributes	*6*
**C. Climate change**	**6**
9. Climate change	*6*
**Sectors affected**	
**Commercial**	**9**
**Environmental**	**5**
**Species or population nuisance traits**	**24**

**Thresholds**	
**BRA**	**41**
**BRA+CCA**	**41**
**Confidence**	
**BRA+CCA**	**0.42**
**BRA**	**0.43**
**CCA**	**0.33**

**Date and Time**
**03/07/2024 18:48:25**

TPS-ISK v2

**Taxon and Assessor details**	
Category	Angiosperms
Taxon name	*Magnolia kobus*
Common name	Kobus Magnolia
Assessor	Mihaela Britvec
**Risk screening context**	
Reason and socio-economic benefits	
Risk assessment area	Continental Europe
Taxonomy	
Native range	
Introduced range	
URL	

			**Response**	**Justification (references or other information)**	**Confidence**
**A. Biogeography/Historical**					
1. Domestication/Cultivation					
1	1.01	Has the taxon been the subject of domestication?	Yes	Magnolia kobus, also known as Kobus Magnolia, is a medium-sized deciduous tree native to the temperate forests of Japan. It is a popular choice in botanical gardens and horticultural landscapes, due in part to its showy white flowers and attractive foliage (Moesker, 2019). The usual claim is that its first introduction to Europe was around 1875–9 by Charles Maries, an English plant collector working for the Veitch nursery (Gardiner, 2000). However, it seems to have reached Count Wodzicki's arboretum at Niedzwiedz, S Poland, as early as 1820 (Anisko & Czekalski, 1991).	Very high
2	1.02	Is the taxon harvested in the wild and likely to be sold or used in its live form?	Yes	Magnolia kobus is an ornamental tree and is grown (and sold) in numerous nurseries.	Very high
3	1.03	Does the taxon have invasive races, varieties, sub-taxa or congeners?	Yes	For example, Magnolia champaca is an iinvasive species (https://www.cabidigitallibrary.org/doi/10.1079/cabicompendium.33923).	High
2. Climate, distribution and introduction risk					
4	2.01	How similar are the climatic conditions between the risk assessment area and the taxon's native range?	Medium	Magnolia kobus is a deciduous tree native to the temperate forests of Japan (Moesker, 2019). The average temperature and precipitation of the some region of Continental Europe are very similar to those in their native range.	Medium
5	2.02	What is the quality of the climate matching data?	Medium	professional judgement	Medium
6	2.03	Is the taxon known to be present outside of cultivation in the risk assessment area?	Yes	https://www.botanic-gardens-ljubljana.com/en/component/plants/magnolia-kobus, https://doi.org/10.15835/nbha49112223,	High
7	2.04	How many potential vectors could the taxon use to enter the risk assessment area?	>1	For example: dispersal by animals and humans (planting, transport)	High
8	2.05	Is the taxon currently found in close proximity to, and likely to enter, the risk assessment area in the near future (e.g. unintentional or intentional introductions)?	Not applicable	https://www.botanic-gardens-ljubljana.com/en/component/plants/magnolia-kobus	Very high
3. Invasive elsewhere					
9	3.01	Has the taxon become naturalised outside its native range?	Yes	Magnolia kobus is recognized as an invasive species in the State of Delaware (McAvoy 2016), and naturalized populations have been observed in the southeastern Pennsylvania counties of Chester, Montgomery, and Bucks (Moesker, 2019).	Medium
10	3.02	In the taxon's introduced range, are there any known adverse impacts to wild or commercial species?	Yes	Magnolia kobus plants contain sesquiterpene lactones, chemical compounds that can produce allelopathic effects on surrounding vegetation. In a study by Abdelgaleil and Hashinaga (2007), costunolide and parthenolide inhibited seed germination and root length of four test species, viz., wheat, lettuce, radish, and onion. A similar study by Abdelgaleil et al. (2009) using wild oats showed reductions in growth with increasing concentrations of costunolide and parthenolide. The presence of costunolide and parthenolide in M. kobus can result in allelopathic effects on nearby plant species by the release of the chemicals into the soil via roots and fallen debris (Hampton 2010).	Medium
11	3.03	In the taxon's introduced range, are there any known adverse impacts to agriculture or forestry?	Yes	The Magnolia kobus seedlings did not exhibit evidence of deer browse, despite the presence of a large local population of white-tailed deer. This suggests that these plants are not a preferred source of food for deer. The M. kobus seedlings are able to grow uninhibited, while many native tree and shrub species are continually stunted (Moesker, 2019). This can result in the species having a competitive advantage over other plants that face pressures from deer browsing, including witch hazel (Hamamelis virginiana) and dogwood species (Cornus spp.).	Low
12	3.04	In the taxon's introduced range, are there any known adverse impacts to ecosystem	No	no reference	Low
13	3.05	In the taxon's introduced range, are there any known adverse socio-economic impacts?	No	no reference	Low
**B. Biology/Ecology**					
4. Undesirable (or persistence) traits					
14	4.01	Is the taxon likely to be poisonous or pose other risks to human health?	No	no reference	Low

15	4.02	Is the taxon likely to suppress the growth of one or more native species?	Yes	The leaves of Magnolia kobus remain green until late autumn, well after the leaves of native plants have changed colour or have dropped. This gives M. kobus the ability to produce and store more energy than native plant species (Wenning 2014). This factor, in addition to the species' lack of predators, vigorous growth habit, allelopathic properties, ability to reproduce vegetatively, and tolerance of a variety of light conditions, contributes to the competitive advantage held by M. kobus. Such an advantage threatens the growth of other less competitive species, thereby threatening to alter the structure of a vegetative	Low
16	4.03	Are there any threatened or protected native species that the taxon would parasitise in the risk assessment area?	No	Magnolia kobus are not identified as parasitic species.	High
17	4.04	Is the taxon adaptable in terms of climatic and other environmental conditions, thus enhancing its potential persistence if it has invaded or is likely to invade the risk	Yes	Magnolia kobus is tolerant of a wide range of temperatures, soils, and light availability (Moesker, 2019).	High
18	4.05	Is the taxon likely to disrupt terrestrial ecosystem function if it has invaded or is likely to invade the risk assessment area?	Yes	American robins were observed consuming several seeds of Magnolia kobus at a time before flying into the adjacent forest. The birds returned within a few minutes to continue feasting on the bright red fruit before again retreating into the forest. This pattern continued until the Magnolia trees were stripped of their fruit. Native fruiting species such as Cornus racemosa are used as a food source by birds in the fall months when Magnolia seeds are also available. Since birds are drawn to the large and attractive Magnolia seeds, they consume smaller amounts of the fruit of native species, thereby contributing to the dispersal of M. kobus while limiting the spread of native species (Moesker, 2019).	Medium
19	4.06	Is the taxon likely to exert adverse impacts on ecosystem services in the risk	No	no evidence	Low
20	4.07	Is the taxon likely to host or function as a vector for recognised pests and pathogens that are present in the risk assessment area?	Yes	Magnolia kobus is host plant to a variety of scale insects in the genera Eulecanium, Pulvinaria, and Pseudaulacaspis (García Morales et al. 2019).	Medium
21	4.08	Is the taxon likely to host or function as a vector for recognised pests and pathogens that are absent in the risk assessment area?	Yes	In its native range, Magnolia kobus is a host plant for Caloptilia magnoliae and Gibbovalva kobusi, both of which are moths in the family Gracillariidae (De Prins and De Prins 2019). It is also host to a variety of scale insects in the genera Eulecanium, Pulvinaria, and Pseudaulacaspis (García Morales et al. 2019).	Medium
22	4.09	Is the taxon likely to be released from cultivation?	Yes	Magnolia kobus is a popular choice for horticultural landscapes because its ability to tolerate a variety of environmental conditions. This has allowed the species to become established in regions well beyond its native range, and it is now beginning to display characteristics that suggest a high invasive potential. The average current year's growth of five M. kobus trees in Hickory Valley was 74.6 cm (29.37 in). These trees were approximately 10 years old, based on the number of tree rings counted in the cross-section of Magnolia trees that were of similar size. A plant that grows 12 inches or less per year is considered to be slow-growing, 12–24 inches per year is a medium growth rate, and above 25 inches per year is considered fastgrowing (Arbor Day Foundation 2015; Moesker, 2019).	High
23	4.10	Is the taxon versatile in habitat use?	Yes	Magnolia kobus is tolerant of a wide range of temperatures, soils, and light availability (Moesker, 2019).	Very high
24	4.11	Is it likely that the taxon's mode of existence or behaviours will reduce habitat quality for native species?	No	no evidence	Low
25	4.12	Is the taxon likely to maintain a viable population even when present in low densities (or persist in adverse conditions by way of a dormant form)?	Yes	The pollen from Magnolia species may be transported by beetles to a different plant, but insects often move between flowers on the same plant. In Magnolia kobus, this results in self-fertilization, as the species is selfcompatible (Azuma et al. 2001). This provides an advantage when the species is established in new habitats. Self-fertilization permits the quick development of a population of individuals that have characteristics adapted to the new environment. This is also advantageous for an invading species as only one or a few individuals are required for the quick colonization of an area (Heiser 1962). In addition to spreading by seed, Magnolia kobus is also able to reproduce by layering. This is a process by which a lower branch that is still connected to the main stem forms roots where it comes into contact with the soil	Medium
5. Resource exploitation					
26	5.01	Is the taxon likely to put threatened or protected native species under competitive pressure in the risk assessment area?	Yes	Magnolia kobus plants are not a preferred source of food for deer. The vigorous nature of M. kobus combined with the lack of growth control from deer browse and insect pests is likely to result in it overwhelming and outcompeting native plant species (Moesker,	Low
27	5.02	Is the taxon likely to sequester resources to the detriment of native species in the risk assessment area?	No	no evidence	Low
6. Reproduction					
28	6.01	Is the taxon likely to exhibit changes in reproductive strategy in response to environmental conditions?	No	no evidence	Low

29	6.02	Is the taxon likely to produce viable propagules in the risk assessment area?	Yes	In addition to spreading by seed, Magnolia kobus is also able to reproduce by layering. This is a process by which a lower branch that is still connected to the main stem forms roots where it comes into contact with the soil (Evans and Blazich 1999). Although the initial spread of a population may be limited by the presence of only a few mature seed-bearing trees, young seedlings are able to reproduce vegetatively (Moesker, 2019).	High
30	6.03	Is the taxon likely to hybridise with native species under natural conditions?	No	no reference	Low
31	6.04	Is the taxon likely to be hermaphroditic or to exhibit asexual reproduction?	Yes	Magnolia kobus is also able to reproduce by layering (Moesker, 2019).	Very high
32	6.05	Is the taxon dependent on the presence of another species (or specific habitat features) to complete its life cycle?	No	Magnolia species rely on beetles for pollination, and beetles in the family Nitidulidae are the main pollinators (Fulcher and White 2012).	Medium
33	6.06	Is the taxon likely to produce a large number of propagules?	No	The seed coat (testa) of many Magnolia species consists of five layers, including a pellicle, hypodermis, fleshy tissue, inner hypodermis, and a woody inner testa. The middle fleshy layer contains oil and reducing sugars (Barbour 2008). The reducing sugars include glucose, fructose, and galactose, which are the main forms of carbohydrates used by birds for energy (Black 2015). Magnolia species produce fruit with a high fat content that ranges from 33–62% (Frick-Ruppert 2010).	High
34	6.07	How many time units (days, months, years) does the taxon require to reach the age at first reproduction?	>10	More than 10 years. Magnolia kobus takes about 15 years before it blooms for the first time (Moesker, 2019).	High
7. Dispersal mechanisms					
35	7.01	How many potential vectors or pathways could the taxon use to disperse within the risk assessment area (with suitable habitats nearby)?	>1	dispersal by birds, dispersal by humans	Medium
36	7.02	Will any of these vectors or pathways bring the taxon in close proximity to one or more protected areas?	Yes	dispersal by animals (birds)	Medium
37	7.03	Does the taxon have a specialised means of attachment such that it enhances the likelihood of dispersal?	No	no evidence	High
38	7.04	Is natural dispersal of the taxon likely to occur as seeds or spores in the risk assessment area?	Yes	Birds are attracted to the brightly coloured seeds of Magnolia kobus, the appeal of which is accentuated by the funiculus, which allows the seeds to dangle from the follicetum (Barbour 2008).	High
39	7.05	Is natural dispersal of the taxon likely to occur as fragments or seedlings in the risk assessment area?	Yes	Magnolia kobus seedlings can be propagated by cultivation in nurseries.	Medium
40	7.06	Are any life stages of the taxon likely to migrate into the risk assessment area for reproduction?	Yes	Its fruit are attractive to some birds. The fat in the seeds of M. kobus can provide migratory birds with the energy that they need to reach their destination (Moesker, 2019).	High
41	7.07	Are propagules of the taxon likely to be dispersed in the risk assessment area by other species?	Yes	Magnolia kobus fruits are attractive to some migratory birds (Moesker, 2019).	High
42	7.08	Is dispersal of the taxon along any of the vectors or pathways mentioned in the previous seven Questions (35–41: i.e. either unintentional or intentional) likely to be	Yes	dispersal by birds	Medium
43	7.09	Is dispersal of the taxon density dependent?	No	no reference	Low
8. Tolerance attributes					
44	8.01	Is the taxon able to withstand being in water for extended periods at some stage of its life cycle?	No	no reference	Low
45	8.02	Is the taxon tolerant of a wide range of soil or air quality conditions?	Yes	Magnolia kobus is tolerant of a wide range of soils and light availability (Moesker, 2019).	High
46	8.03	Can the taxon be controlled or eradicated in the wild with chemical, biological or other agents and means?	Yes	It has no reference, but as all plants, Magnolia kobus could also be eradicated with herbicides.	Medium
47	8.04	Is the taxon likely to benefit from environmental or human disturbance?	Yes	Magnolia kobus requires gaps in the forest canopy for regeneration (Nakamura and Krestov 2005), which indicates that light availability is a growth limiting factor for this species. Disturbance may also directly influence the success of an invasive species by altering the suitability of the environment, or indirectly by increasing the availability of resources through the reduction of competing species. The mortality and canopy dieback of ash trees (Fraxinus spp.) due to the invasive emerald ash borer has created gaps in the canopy. These canopy gaps provide an opportunity for M. kobus to become established as the availability of light	Medium
48	8.05	Is the taxon able to tolerate soil acidity, salinity or other parameter levels that are higher or lower than those found in its usual	Yes	The species is tolerant of a variety of soil conditions, including clay, loam, and sand (Moesker, 2019).	High
49	8.06	Are there effective natural enemies of the taxon present in the risk assessment area?	No	Magnolia kobus face few threats from pests and diseases. The plants contain compounds that have antimicrobial, nematicidal, and insecticidal properties (Fulcher and White 2012). Magnolia kobus contains several lignans, including kobusin and sesamin, whose biological activities protect the plant from insect pests (Kamikado et al. 1975; Trifunovic et al. 2003). The ability of this species to deter insect pests by chemical means increases its ability to overcome ecological barriers.	High
**C. Climate change**					
9. Climate change					

50	9.01	Under the predicted future climatic conditions, are the risks of entry into the risk assessment area posed by the taxon likely to increase, decrease or not change?	Increase	professional judgement	Medium
51	9.02	Under the predicted future climatic conditions, are the risks of establishment within the risk assessment area posed by the taxon likely to increase, decrease or not	Increase	professional judgement	Medium
52	9.03	Under the predicted future climatic conditions, are the risks of dispersal within the risk assessment area posed by the taxon likely to increase, decrease or not change?	Increase	professional judgement	Medium
53	9.04	Under the predicted future climatic conditions, what is the likely magnitude of future potential impacts by the taxon on biodiversity or ecosystem integrity and	Higher	professional judgement	Low
54	9.05	Under the predicted future climatic conditions, what is the likely magnitude of future potential impacts by the taxon on ecosystem structure or function?	Higher	professional judgement	Low
55	9.06	Under the predicted future climatic conditions, what is the likely magnitude of future potential impacts by the taxon on ecosystem services and related socio-	Higher	professional judgement	Low

**Statistics**	
**Scores**	
**BRA**	**38.5**
**BRA Outcome**	**Medium**
**BRA+CCA**	**50.5**
**BRA+CCA Outcome**	**High**
**Score partition**	
**A. Biogeography/Historical**	**13.5**
1. Domestication/Cultivation	*4.0*
2. Climate, distribution and introduction risk	*2.0*
3. Invasive elsewhere	*7.5*
**B. Biology/Ecology**	**25.0**
4. Undesirable (or persistence) traits	*8.0*
5. Resource exploitation	*5.0*
6. Reproduction	*1.0*
7. Dispersal mechanisms	*6.0*
8. Tolerance attributes	*5.0*
**C. Climate change**	**12.0**
9. Climate change	*12.0*
**Answered Questions**	
Total	***55***
**A. Biogeography/Historical**	**13**
1. Domestication/Cultivation	*3*
2. Climate, distribution and introduction risk	*5*
3. Invasive elsewhere	*5*
**B. Biology/Ecology**	**36**
4. Undesirable (or persistence) traits	*12*
5. Resource exploitation	*2*
6. Reproduction	*7*
7. Dispersal mechanisms	*9*
8. Tolerance attributes	*6*
**C. Climate change**	**6**
9. Climate change	*6*
**Sectors affected**	
**Commercial**	**15**
**Environmental**	**11**
**Species or population nuisance traits**	**30**

**Thresholds**	
**BRA**	**41**
**BRA+CCA**	**41**
**Confidence**	
**BRA+CCA**	**0.55**
**BRA**	**0.57**
**CCA**	**0.38**

**Date and Time**
**11/06/2024 11:15:14**

TPS-ISK v2

**Taxon and Assessor details**	
Category	Angiosperms
Taxon name	*Parrotia persica*
Common name	Persian Ironwood
Assessor	Mihaela Britvec
**Risk screening context**	
Reason and socio-economic benefits	
Risk assessment area	Continental Europe
Taxonomy	
Native range	
Introduced range	
URL	

			Response	Justification (references or other information)	Confidence
**A. Biogeography/Historical**					
1. Domestication/Cultivation					
1	1.01	Has the taxon been the subject of domestication?	Yes	By 1840, Parrotia was reported in cultivation in Russia, at the botanical garden in St. Petersburg, and Kew Gardens received a specimen from St. Petersburg the next year. By 1880, Parrotia reached the United States, growing at the Harvard Botanic Garden in Cambridge, Massachusetts (Nicholson 1989).	Very high
2	1.02	Is the taxon harvested in the wild and likely to be sold or used in its live form?	Yes	Parrotia persica is cultivated as an ornamental tree (Bailey 1947).	Very high
3	1.03	Does the taxon have invasive races, varieties, sub-taxa or congeners?	No	no evidence	Medium
2. Climate, distribution and introduction risk					
4	2.01	How similar are the climatic conditions between the risk assessment area and the taxon's native range?	Medium	professional judgement	Medium
5	2.02	What is the quality of the climate matching data?	Medium	professional judgement	Low
6	2.03	Is the taxon known to be present outside of cultivation in the risk assessment area?	No	In Continental Europe is this species only known in cultivation.	High
7	2.04	How many potential vectors could the taxon use to enter the risk assessment area?	One	distribution by humans	Medium
8	2.05	Is the taxon currently found in close proximity to, and likely to enter, the risk assessment area in the near future (e.g. unintentional or intentional introductions)?	No	Parrotia persica is known as an ornamental tree (Bailey 1947).	Very high
3. Invasive elsewhere					
9	3.01	Has the taxon become naturalised outside its native range?	No	Parrotia persica is only known horticulture as an ornamental tree (Bailey 1947).	High
10	3.02	In the taxon's introduced range, are there any known adverse impacts to wild or commercial species?	No	no evidence	Medium
11	3.03	In the taxon's introduced range, are there any known adverse impacts to agriculture or forestry?	No	no evidence	Medium
12	3.04	In the taxon's introduced range, are there any known adverse impacts to ecosystem	No	no evidence	Medium
13	3.05	In the taxon's introduced range, are there any known adverse socio-economic impacts?	No	no evidence	Medium
**B. Biology/Ecology**					
4. Undesirable (or persistence) traits					
14	4.01	Is the taxon likely to be poisonous or pose other risks to human health?	No	Parrotia persica is non-toxic to humans (https://www.gardenia.net/plant/parrotia-persica-persian-	High
15	4.02	Is the taxon likely to suppress the growth of one or more native species?	No	no reference	Medium
16	4.03	Are there any threatened or protected native species that the taxon would parasitise in the risk assessment area?	No	Parrotia persica is not known as parasite.	Medium
17	4.04	Is the taxon adaptable in terms of climatic and other environmental conditions, thus enhancing its potential persistence if it has invaded or is likely to invade the risk	Yes	This tree is adaptable to a wide range of a soil types (https://www.gardenia.net/plant/parrotia-persica-persian-ironwood).	Low
18	4.05	Is the taxon likely to disrupt terrestrial ecosystem function if it has invaded or is likely to invade the risk assessment area?	No	no evidence	Medium
19	4.06	Is the taxon likely to exert adverse impacts on ecosystem services in the risk	No	no evidence	Medium
20	4.07	Is the taxon likely to host or function as a vector for recognised pests and pathogens that are present in the risk assessment area?	No	Parrotia persica is relatively resistant to pests and disease, except armillaria root rot (https://www.gardenia.net/plant/parrotia-persica-persian-ironwood).	Medium
21	4.08	Is the taxon likely to host or function as a vector for recognised pests and pathogens that are absent in the risk assessment area?	No	It should be keep an eye out for Armillaria (https://www.gardenia.net/plant/parrotia-persica-persian-ironwood).	Low
22	4.09	Is the taxon likely to be released from cultivation?	No	The tree has a slow to moderate growth rate (https://www.gardenia.net/plant/parrotia-persica-persian-	Medium
23	4.10	Is the taxon versatile in habitat use?	Yes	https://www.gardenia.net/plant/parrotia-persica-persian-ironwood	Low
24	4.11	Is it likely that the taxon's mode of existence or behaviours will reduce habitat quality for native species?	No	Parrotia persica doesn't pose a threat to native vegetation (https://www.gardenia.net/plant/parrotia-persica-persian-ironwood).	Low

25	4.12	Is the taxon likely to maintain a viable population even when present in low densities (or persist in adverse conditions by way of a dormant form)?	No	no reference	Low
5. Resource exploitation					
26	5.01	Is the taxon likely to put threatened or protected native species under competitive pressure in the risk assessment area?	No	Parrotia persica doesn't pose a threat to native vegetation.	Medium
27	5.02	Is the taxon likely to sequester resources to the detriment of native species in the risk assessment area?	No	no reference	Low
6. Reproduction					
28	6.01	Is the taxon likely to exhibit changes in reproductive strategy in response to environmental conditions?	No	no reference	Low
29	6.02	Is the taxon likely to produce viable propagules in the risk assessment area?	Yes	The fruits of Parrotia persica contain seeds, but its propagation in horticulture is by cuttings.	Low
30	6.03	Is the taxon likely to hybridise with native species under natural conditions?	No	no evidence	Medium
31	6.04	Is the taxon likely to be hermaphroditic or to exhibit asexual reproduction?	Yes	Parrotia persica is reproduced through asexual reproduction by coppice (shoot) (Andrews et al. 2007).	Medium
32	6.05	Is the taxon dependent on the presence of another species (or specific habitat features) to complete its life cycle?	No	no evidence	Medium
33	6.06	Is the taxon likely to produce a large number of propagules?	No	The fruit is a two-parted capsule containing two seeds.	High
34	6.07	How many time units (days, months, years) does the taxon require to reach the age at first reproduction?	>10	more than 10 months	Low
7. Dispersal mechanisms					
35	7.01	How many potential vectors or pathways could the taxon use to disperse within the risk assessment area (with suitable habitats nearby)?	One	dispersion by humans	Medium
36	7.02	Will any of these vectors or pathways bring the taxon in close proximity to one or more protected areas?	Yes	professional judgement	Medium
37	7.03	Does the taxon have a specialised means of attachment such that it enhances the likelihood of dispersal?	No	no evidence	Medium
38	7.04	Is natural dispersal of the taxon likely to occur as seeds or spores in the risk	No	https://www.gardenia.net/plant/parrotia-persica-persian-ironwood	Medium
39	7.05	Is natural dispersal of the taxon likely to occur as fragments or seedlings in the risk assessment area?	Yes	The cuttings of Parrotia persica is cultivated (Andrews et al. 2007).	High
40	7.06	Are any life stages of the taxon likely to migrate into the risk assessment area for reproduction?	No	professional judgement	Low
41	7.07	Are propagules of the taxon likely to be dispersed in the risk assessment area by other species?	No	This fruits of Parrotia persica is not a primary source of food for wildlife.	Medium
42	7.08	Is dispersal of the taxon along any of the vectors or pathways mentioned in the previous seven Questions (35–41: i.e. either unintentional or intentional) likely to be	No	no evidence	Low
43	7.09	Is dispersal of the taxon density dependent?	No	no evidence	Low
8. Tolerance attributes					
44	8.01	Is the taxon able to withstand being in water for extended periods at some stage of its life cycle?	No	no evidence	Low
45	8.02	Is the taxon tolerant of a wide range of soil or air quality conditions?	Yes	This tree is adaptable to a wide range of soils types.	High
46	8.03	Can the taxon be controlled or eradicated in the wild with chemical, biological or other agents and means?	Yes	professional judgement (treatment with herbicides).	Low
47	8.04	Is the taxon likely to benefit from environmental or human disturbance?	No	no evidence	Low
48	8.05	Is the taxon able to tolerate soil acidity, salinity or other parameter levels that are higher or lower than those found in its usual	Yes	Parrotia persica can grow in slightly alkaline and acidic soils (Gilman and Watson 1994).	Medium
49	8.06	Are there effective natural enemies of the taxon present in the risk assessment area?	No	professional judgement	Low
**C. Climate change**					
9. Climate change					
50	9.01	Under the predicted future climatic conditions, are the risks of entry into the risk assessment area posed by the taxon likely to increase, decrease or not change?	No change	professional judgement	Low
51	9.02	Under the predicted future climatic conditions, are the risks of establishment within the risk assessment area posed by the taxon likely to increase, decrease or not	No change	professional judgement	Low

52	9.03	Under the predicted future climatic conditions, are the risks of dispersal within the risk assessment area posed by the taxon likely to increase, decrease or not change?	No change	professional judgement	Low
53	9.04	Under the predicted future climatic conditions, what is the likely magnitude of future potential impacts by the taxon on biodiversity or ecosystem integrity and	No change	professional judgement	Medium
54	9.05	Under the predicted future climatic conditions, what is the likely magnitude of future potential impacts by the taxon on ecosystem structure or function?	No change	professional judgement	Medium
55	9.06	Under the predicted future climatic conditions, what is the likely magnitude of future potential impacts by the taxon on ecosystem services and related socio-	No change	professional judgement	Medium

Statistics	
**Scores**	
**BRA**	**4.0**
**BRA Outcome**	**Medium**
**BRA+CCA**	**4.0**
**BRA+CCA Outcome**	**Medium**
**Score partition**	
**A. Biogeography/Historical**	**2.0**
1. Domestication/Cultivation	*2.0*
2. Climate, distribution and introduction risk	*0.0*
3. Invasive elsewhere	*0.0*
**B. Biology/Ecology**	**2.0**
4. Undesirable (or persistence) traits	*2.0*
5. Resource exploitation	*0.0*
6. Reproduction	*1.0*
7. Dispersal mechanisms	*-2.0*
8. Tolerance attributes	*1.0*
**C. Climate change**	**0.0**
9. Climate change	*0.0*
**Answered Questions**	
Total	***55***
**A. Biogeography/Historical**	**13**
1. Domestication/Cultivation	*3*
2. Climate, distribution and introduction risk	*5*
3. Invasive elsewhere	*5*
**B. Biology/Ecology**	**36**
4. Undesirable (or persistence) traits	*12*
5. Resource exploitation	*2*
6. Reproduction	*7*
7. Dispersal mechanisms	*9*
8. Tolerance attributes	*6*
**C. Climate change**	**6**
9. Climate change	*6*
**Sectors affected**	
**Commercial**	**3**
**Environmental**	**1**
**Species or population nuisance traits**	**1**

Thresholds	
**BRA**	**41**
**BRA+CCA**	**41**
**Confidence**	
**BRA+CCA**	**0.46**
**BRA**	**0.47**
**CCA**	**0.38**

Date and Time
**26/06/2024 10:27:09**

TPS-ISK v2

Taxon and Assessor details	
Category	Angiosperms
Taxon name	*Paulownia tomentosa*
Common name	Princess Tree
Assessor	S. Luke Flory
**Risk screening context**	
Reason and socio-economic benefits	Valued as an ornamental tree in parks and gardens, and is an important cultural species in Japan.
Risk assessment area	Continental Europe
Taxonomy	Kingdom: Plantae Phylum: Tracheophytes Class: Angiosperms Order: Lamiales Family:
Native range	Central and eastern China and the Korean Peninsula
Introduced range	Eastern US, Western and Central Europe
URL	https://www.gbif.org/species/3170823 https://en.wikipedia.org/wiki/Paulownia_tomentosa

			Response	Justification (references or other information)	Confidence
**A. Biogeography/Historical**					
1. Domestication/Cultivation					
1	1.01	Has the taxon been the subject of domestication?	No	Cultivated but no evidence for selection for specific traits, including reduced weediness.	High
2	1.02	Is the taxon harvested in the wild and likely to be sold or used in its live form?	Yes	Bergmann, B.A. Propagation method influences first year field survival and growth of Paulownia. New Forests 16, 251–264 (1998). https://doi.org/10.1023/A:1006529622871	Medium
3	1.03	Does the taxon have invasive races, varieties, sub-taxa or congeners?	Yes	Paulownia fortunei has been evaluated as high invasion risk in the US. https://assessment.ifas.ufl.edu/assessments/paulownia-	High
2. Climate, distribution and introduction risk					
4	2.01	How similar are the climatic conditions between the risk assessment area and the taxon's native range?	High	Climate of Europe is highly suitable for self-sustaining populations given the climate matching between Europe and the native range in Asia. Research in Europe has also shown that the climate is suitable for this species. Jakubowski, M. Cultivation Potential and Uses of Paulownia Wood: A Review. Forests 2022, 13, 668. https://doi.org/10.3390/f13050668	High
5	2.02	What is the quality of the climate matching data?	High	Good evidence that the climate is suitable. such as, Franz, E. S. S. L. "From ornamental to detrimental? The incipient invasion of Central Europe by Paulownia tomentosa." Preslia 79 (2007): 377-	High
6	2.03	Is the taxon known to be present outside of cultivation in the risk assessment area?	Yes	Franz, E. S. S. L. "From ornamental to detrimental? The incipient invasion of Central Europe by Paulownia tomentosa." Preslia 79 (2007): 377-389.	Very high
7	2.04	How many potential vectors could the taxon use to enter the risk assessment area?	One	Intentional introduction for forestry or ornamental uses.	High
8	2.05	Is the taxon currently found in close proximity to, and likely to enter, the risk assessment area in the near future (e.g. unintentional or intentional introductions)?	Not applicable	https://www.gbif.org/species/3170823	High
3. Invasive elsewhere					
9	3.01	Has the taxon become naturalised outside its native range?	Yes	Widespread in eastern USA where it is non-native. Introduced in 1834. https://www.gbif.org/species/3170823	Very high
10	3.02	In the taxon's introduced range, are there any known adverse impacts to wild or commercial species?	Yes	From Innes, Robin J. 2009. Paulowina tomentosa. In: Fire Effects Information System, [Online]. U.S. Department of Agriculture, Forest Service, Rocky Mountain Research Station, Fire Sciences Laboratory (Producer). Available: http://www.fs.fed.us/database/feis/ [2010, January 22] and Langdon, K.R. & K.D. Johnson (1994) Additional Notes on Invasiveness of Paulownia tomentosa in Natural Areas. Natural Areas Journal. 14(2): 139-140. In the Southeast US, it is typically considered a substantial or severe threat to native communities. Evidence of several wildfires that have resulted in significant reproduction of this invader, even though the closest parent trees were a few kilometers away. The park's (Great Smoky Mountains National Park in TN and NC) future fire program may repetitively	Medium
11	3.03	In the taxon's introduced range, are there any known adverse impacts to agriculture or forestry?	No	No evidence that agriculture or forestry are impacted by this species.	Medium
12	3.04	In the taxon's introduced range, are there any known adverse impacts to ecosystem	No	No impacts to ecosystem services identified.	High
13	3.05	In the taxon's introduced range, are there any known adverse socio-economic impacts?	No	None identified.	Medium
**B. Biology/Ecology**					
4. Undesirable (or persistence) traits					
14	4.01	Is the taxon likely to be poisonous or pose other risks to human health?	No	No evidence. https://www.gardenersworld.com/plants/paulownia-tomentosa/	High
15	4.02	Is the taxon likely to suppress the growth of one or more native species?	Yes	Williams, R.; Wang, H. Effects of Wildfire and the Presence of the Invasive Paulownia tomentosa on the Regeneration of Native Tree Species in North-Central Appalachia. Fire 2021, 4, 60. https://doi.org/10.3390/fire4030060	High
16	4.03	Are there any threatened or protected native species that the taxon would parasitise in the risk assessment area?	No	No evidence.	Low
17	4.04	Is the taxon adaptable in terms of climatic and other environmental conditions, thus enhancing its potential persistence if it has invaded or is likely to invade the risk	No	No evidence that the species is adaptable.	Low
18	4.05	Is the taxon likely to disrupt terrestrial ecosystem function if it has invaded or is likely to invade the risk assessment area?	No	No evidence.	Medium

19	4.06	Is the taxon likely to exert adverse impacts on ecosystem services in the risk	No	No evidence.	Low
20	4.07	Is the taxon likely to host or function as a vector for recognised pests and pathogens that are present in the risk assessment area?	No	No evidence.	Low
21	4.08	Is the taxon likely to host or function as a vector for recognised pests and pathogens that are absent in the risk assessment area?	No	No evidence.	Medium
22	4.09	Is the taxon likely to be released from cultivation?	Yes	Grows rapidly and produces a very large number of seeds under a wide range of conditions. 1. Innes, Robin J. 2009. Paulowina tomentosa. In: Fire Effects Information System, [Online]. U.S. Department of Agriculture, Forest Service, Rocky Mountain Research Station, Fire Sciences Laboratory (Producer). Available: http://www.fs.fed.us/database/feis/ [2010, January 22]. 1. Winged seeds are easily transported by wind and water over considerable distances; seedling have been observed more than 2 miles (3 km) from parent species in mountainous regions of NC	High
23	4.10	Is the taxon versatile in habitat use?	Yes	1.a-b. Global Invasive Species Database (ISSG). Available: http://www.issg.org/database/species/ecology.asp?si=440&fr=1st [2010, January 20]. 2. Innes, Robin J. 2009. Paulowina tomentosa. In: Fire Effects Information System, [Online]. U.S. Department of Agriculture, Forest Service, Rocky Mountain Research Station, Fire Sciences Laboratory (Producer). Available: http://www.fs.fed.us/database/feis/ [2010, January 22]. 1.a. A highly adaptable 'escapee,' P. tomentosa is found in many site, soil, and forest type conditions, including soils commonly found in the order Alfisols. 1.b. SE-EPPC reports the P. tomentosa "tolerates high soil acidity, drought, and low soil fertility." 2.a. Grows best on moist, incompacted, well-drained soils. 2.b. Best growth is obtained within strongly acidic to mildly alkaline pH levels (range: 5.5-7.5). 2.c. Soil texture may play a role in invasiveness; sandy or loamy soils with low clay content appear optimum. 2.d. Nitrogen and phosphorus are essential for tree growth; tolerant of low soil fertiltity but grows better in fertile	Very high
24	4.11	Is it likely that the taxon's mode of existence or behaviours will reduce habitat quality for native species?	No	1.a-b. Innes, Robin J. 2009. Paulowina tomentosa. In: Fire Effects Information System, [Online]. U.S. Department of Agriculture, Forest Service, Rocky Mountain Research Station, Fire Sciences Laboratory (Producer). Available: http://www.fs.fed.us/database/feis/ [2010, January 22]. 2. Longbrake, A. Christina W. (2001) Ecology and invasive potential of Paulownia tomentosa (Scrophulariaceae) in a hardwood forest landscape. Athens, Ohio: Ohio University. 174p. Dissertation. 1.a. Domestic livestock and wildlife regularly consume leaves, flowers, and branches. 1.b. Leaves were highly palatable to domestic goats in experimental studies; leaves appear to be equally palatable to wildlife. 2. High rates of herbivory were observed on young seedlings by unspecified burrowing mammals and white-tailed	High
25	4.12	Is the taxon likely to maintain a viable population even when present in low densities (or persist in adverse conditions by way of a dormant form)?	Yes	Lovenshimer JB, Madritch MD. Plant Community Effects and Genetic Diversity of Post-fire Princess Tree (Paulownia tomentosa) Invasions. Invasive Plant Science and Management. 2017;10(2):125-135. doi:10.1017/inp.2017.14	Medium
5. Resource exploitation					
26	5.01	Is the taxon likely to put threatened or protected native species under competitive pressure in the risk assessment area?	No	No evidence.	Low
27	5.02	Is the taxon likely to sequester resources to the detriment of native species in the risk assessment area?	No	No evidence.	Low
6. Reproduction					
28	6.01	Is the taxon likely to exhibit changes in reproductive strategy in response to environmental conditions?	No	No evidence.	Low
29	6.02	Is the taxon likely to produce viable propagules in the risk assessment area?	Yes	https://www.gbif.org/species/3170823	Very high
30	6.03	Is the taxon likely to hybridise with native species under natural conditions?	No	No other species from this genus are native to Europe so hybridization is unlikely.	High
31	6.04	Is the taxon likely to be hermaphroditic or to exhibit asexual reproduction?	No	No evidence.	Medium
32	6.05	Is the taxon dependent on the presence of another species (or specific habitat features) to complete its life cycle?	No	no other species needed to complete life cycle.	High
33	6.06	Is the taxon likely to produce a large number of propagules?	Yes	https://www.fs.usda.gov/database/feis/plants/tree/pautom/all.html#Seed%20production	Very high
34	6.07	How many time units (days, months, years) does the taxon require to reach the age at first reproduction?	3	3-5 years to maturity depending on environmental conditions. https://www.fs.usda.gov/database/feis/plants/tree/pautom/all.html#Seed%20production	Medium
7. Dispersal mechanisms					
35	7.01	How many potential vectors or pathways could the taxon use to disperse within the risk assessment area (with suitable habitats nearby)?	>1	Species could be spread intentionally as an ornamental species, as it is in other regions, or it could be spread unintentionally through wind dispersal after the species is established. Kuppinger, Dane Mitchell. 2008. Post-fire vegetation dynamics and the invasion of Paulownia tomentosa in the southern Appalachians. Chapel Hill, NC: University of North Carolina at Chapel Hill. 210 p. Dissertation.	High

36	7.02	Will any of these vectors or pathways bring the taxon in close proximity to one or more protected areas?	Yes	Certainly possible with a wind dispersed species.	Low
37	7.03	Does the taxon have a specialised means of attachment such that it enhances the likelihood of dispersal?	No	No attachment mechanism.	High
38	7.04	Is natural dispersal of the taxon likely to occur as seeds or spores in the risk assessment area?	Yes	The species is commonly dispersed by seed. Duncan, Wilbur H.; Duncan, Marion B. 1988. Trees of the southeastern United States. Athens, GA: The University of Georgia Press. 322 p.	Very high
39	7.05	Is natural dispersal of the taxon likely to occur as fragments or seedlings in the risk assessment area?	No	Possible because the species can sprout vegetatively but unlikely because fragments do not easily produce roots. However, seedlings may be introduced intentionally for ornamental purposes.	Medium
40	7.06	Are any life stages of the taxon likely to migrate into the risk assessment area for reproduction?	Yes	Innes, Robin J. 2009. Paulowina tomentosa. In: Fire Effects Information System, [Online]. U.S. Department of Agriculture, Forest Service, Rocky Mountain Research Station, Fire Sciences Laboratory (Producer). Available: http://www.fs.fed.us/database/feis/ [2010, January 22]. 1. Winged seeds are easily transported by wind and water over considerable distances; seedling have been observed more than 2 miles (3 km) from parent species in mountainous regions of NC	High
41	7.07	Are propagules of the taxon likely to be dispersed in the risk assessment area by other species?	No	Seed dispersal is by wind and water and not likely to occur through animal movement.	High
42	7.08	Is dispersal of the taxon along any of the vectors or pathways mentioned in the previous seven Questions (35–41: i.e. either unintentional or intentional) likely to be	No	Unlikely to be rapid.	Medium
43	7.09	Is dispersal of the taxon density dependent?	No	No evidence.	Medium
8. Tolerance attributes					
44	8.01	Is the taxon able to withstand being in water for extended periods at some stage of its life cycle?	No	No evidence.	Medium
45	8.02	Is the taxon tolerant of a wide range of soil or air quality conditions?	Yes	Tolerates a wide range of soil conditions. Donald, D. G. M. 1990. Paulownia--the tree of the future? South African Forestry Journal. 154: 94-98. Tang, R. C.; Carpenter, S. B.; Wittwer, R. F.; Graves, D. H. 1980. Paulownia -- a crop tree for wood products and reclamation of surface-mined land. Southern Journal of Applied Forestry. 4: 19-24. Turner, Gregory D., et al. "Effect of Acidity on Germination and Seedling Growth of Paulownia Tomentosa." Journal of Applied Ecology, vol. 25, no. 2, 1988, pp. 561–67. JSTOR, https://doi.org/10.2307/2403844. Accessed 31 July 2024.	High
46	8.03	Can the taxon be controlled or eradicated in the wild with chemical, biological or other agents and means?	Yes	Yes, but herbicide control should be integrated with other methods, including changes the conditions that allowed the species to establish initially. https://www.fs.usda.gov/database/feis/plants/tree/pautom/all.html#130	Medium
47	8.04	Is the taxon likely to benefit from environmental or human disturbance?	Yes	The species is highly adapted to human disturbance conditions, which allows or rapid establishment. Innes, Robin J. 2009. Paulowina tomentosa. In: Fire Effects Information System, [Online]. U.S. Department of Agriculture, Forest Service, Rocky Mountain Research Station, Fire Sciences Laboratory (Producer)	High
48	8.05	Is the taxon able to tolerate soil acidity, salinity or other parameter levels that are higher or lower than those found in its usual	No	No evidence.	Low
49	8.06	Are there effective natural enemies of the taxon present in the risk assessment area?	No	No evidence.	Low
**C. Climate change**					
9. Climate change					
50	9.01	Under the predicted future climatic conditions, are the risks of entry into the risk assessment area posed by the taxon likely to increase, decrease or not change?	No change	Tolerates a wide range of climate conditions, but there is limited evidence about how climate change will alter risk of entry. Franz, E. S. S. L. "From ornamental to detrimental? The incipient invasion of Central Europe by Paulownia tomentosa." Preslia 79	Low
51	9.02	Under the predicted future climatic conditions, are the risks of establishment within the risk assessment area posed by the taxon likely to increase, decrease or not	No change	Probability of establishment in Europe are unlikely to change significantly under future climate.	Low
52	9.03	Under the predicted future climatic conditions, are the risks of dispersal within the risk assessment area posed by the taxon likely to increase, decrease or not change?	No change	Likelihood of dispersal in Europe is not likely to change with future climate.	Low
53	9.04	Under the predicted future climatic conditions, what is the likely magnitude of future potential impacts by the taxon on biodiversity or ecosystem integrity and	No change	Unlikely to change.	Low
54	9.05	Under the predicted future climatic conditions, what is the likely magnitude of future potential impacts by the taxon on ecosystem structure or function?	No change	Unlikely to change.	Low
55	9.06	Under the predicted future climatic conditions, what is the likely magnitude of future potential impacts by the taxon on ecosystem services and related socio-	No change	Unlikely to change.	Low

Statistics	
	Scores

**BRA**	**16.0**
**BRA Outcome**	**Medium**
**BRA+CCA**	**16.0**
**BRA+CCA Outcome**	**Medium**
**Score partition**	
**A. Biogeography/Historical**	**9.0**
1. Domestication/Cultivation	*2.0*
2. Climate, distribution and introduction risk	*1.0*
3. Invasive elsewhere	*6.0*
**B. Biology/Ecology**	**7.0**
4. Undesirable (or persistence) traits	*3.0*
5. Resource exploitation	*0.0*
6. Reproduction	*1.0*
7. Dispersal mechanisms	*1.0*
8. Tolerance attributes	*2.0*
**C. Climate change**	**0.0**
9. Climate change	*0.0*
**Answered Questions**	
Total	***55***
**A. Biogeography/Historical**	**13**
1. Domestication/Cultivation	*3*
2. Climate, distribution and introduction risk	*5*
3. Invasive elsewhere	*5*
**B. Biology/Ecology**	**36**
4. Undesirable (or persistence) traits	*12*
5. Resource exploitation	*2*
6. Reproduction	*7*
7. Dispersal mechanisms	*9*
8. Tolerance attributes	*6*
**C. Climate change**	**6**
9. Climate change	*6*
**Sectors affected**	
**Commercial**	**7**
**Environmental**	**1**
**Species or population nuisance traits**	**11**

**Thresholds**	
**BRA**	**41**
**BRA+CCA**	**41**
**Confidence**	
**BRA+CCA**	**0.57**
**BRA**	**0.61**
**CCA**	**0.25**

**Date and Time**
**31/07/2024 16:09:44**

TPS-ISK v2

**Taxon and Assessor details**	
Category	Angiosperms
Taxon name	*Phellodendron amurense*
Common name	Amur Cork Tree
Assessor	Mihaela Britvec
**Risk screening context**	
Reason and socio-economic benefits	
Risk assessment area	Continental Europe
Taxonomy	
Native range	
Introduced range	
URL	

			**Response**	**Justification (references or other information)**	**Confidence**
**A. Biogeography/Historical**					
1. Domestication/Cultivation					
1	1.01	Has the taxon been the subject of domestication?	Yes	Phellodendron amurense is native to the forests of northern China, Manchuria, and Japan. It was introduced to North America in 1856 (Ma and Brach 2007). By 1910, the tree was cultivated both in Europe and in North America.	Very high
2	1.02	Is the taxon harvested in the wild and likely to be sold or used in its live form?	Yes	Amur Cork Tree is introduced by the horticulture as a street trees or ornamental shade trees in parks, arboreta, botanical gardens and cemeteries (Glaeser and Kincait 2005). Its wood and the corky bark is used. The bark is also used medicinally.	Very high
3	1.03	Does the taxon have invasive races, varieties, sub-taxa or congeners?	No	no evidence	Low
2. Climate, distribution and introduction risk					
4	2.01	How similar are the climatic conditions between the risk assessment area and the taxon's native range?	Medium	professional judgement	Low
5	2.02	What is the quality of the climate matching data?	Low	professional judgement	Low
6	2.03	Is the taxon known to be present outside of cultivation in the risk assessment area?	No	no evidence	Medium
7	2.04	How many potential vectors could the taxon use to enter the risk assessment area?	>1	dispersion by humans and animals	High
8	2.05	Is the taxon currently found in close proximity to, and likely to enter, the risk assessment area in the near future (e.g. unintentional or intentional introductions)?	No	no evidence	Low
3. Invasive elsewhere					
9	3.01	Has the taxon become naturalised outside its native range?	Yes	https://doi.org/10.1080/03071375.2005.9747416	High
10	3.02	In the taxon's introduced range, are there any known adverse impacts to wild or commercial species?	Yes	In the northeastern United States, the non-native Phellodendron amurense has invaded a number of forested sites in both urban and suburban woodlands (Morgan and Borysiewicz 2015).	High
11	3.03	In the taxon's introduced range, are there any known adverse impacts to agriculture or forestry?	Yes	Phellodendron amurense as aggressively invading the hemlock forest of the New York Botanical Garden (Morgan and Borysiewicz 2015). Amur Cork Tree outcompetes native tree and shrub species including oaks and hickories in forested natural areas (https://www.invasive.org/alien/fact/pham1.htm).	High
12	3.04	In the taxon's introduced range, are there any known adverse impacts to ecosystem	Yes	https://doi.org/10.1080/03071375.2005.9747416	Low
13	3.05	In the taxon's introduced range, are there any known adverse socio-economic impacts?	Yes	https://doi.org/10.1080/03071375.2005.9747416	Low
**B. Biology/Ecology**					
4. Undesirable (or persistence) traits					
14	4.01	Is the taxon likely to be poisonous or pose other risks to human health?	No	no evidence	Low
15	4.02	Is the taxon likely to suppress the growth of one or more native species?	Yes	The establishment and spread of Phellodendron amurense at three Audubon wildlife sanctuaries in eastern Massachusetts has been documented. It does especially well in forests and wooded areas that have been exposed to human disturbance, where it forms dense stands and crowds out native species. P. amurense changes the light regime of forest understory with shelf-like branching that shades out seedlings of competing species. The high volume of seed produced and relative lack of seed predators gives it an additional competitive advantage over native species (https://www.invasive.org/alien/fact/pham1.htm).	Medium
16	4.03	Are there any threatened or protected native species that the taxon would parasitise in the risk assessment area?	No	no evidence	Low
17	4.04	Is the taxon adaptable in terms of climatic and other environmental conditions, thus enhancing its potential persistence if it has invaded or is likely to invade the risk	Yes	This species is adaptable to different soil types. It is drought-tolerant and air-pollution-tolerant (https://plants.ces.ncsu.edu/plants/phellodendron-amurense/).	Low
18	4.05	Is the taxon likely to disrupt terrestrial ecosystem function if it has invaded or is likely to invade the risk assessment area?	Yes	professional judgement	Low
19	4.06	Is the taxon likely to exert adverse impacts on ecosystem services in the risk	Yes	professional judgement	Low

20	4.07	Is the taxon likely to host or function as a vector for recognised pests and pathogens that are present in the risk assessment area?	No	This tree is virtually pest-free (Gilman and Watson 1994).	Low
21	4.08	Is the taxon likely to host or function as a vector for recognised pests and pathogens that are absent in the risk assessment area?	No	professional judgement	Low
22	4.09	Is the taxon likely to be released from cultivation?	Yes	Amur Cork Tree grows rapidly (Gilman and Watson 1994).	High
23	4.10	Is the taxon versatile in habitat use?	Yes	For example, the tree is adaptable to different soil types (https://plants.ces.ncsu.edu/plants/phellodendron-amurense/).	Medium
24	4.11	Is it likely that the taxon's mode of existence or behaviours will reduce habitat quality for native species?	Yes	Oaks and hickories provide a nutritious fat-containing nut for wildlife that remains available through the winter, whereas Amur Cork Tree provides sugary berries that are lower in nutritional value. Many birds and mammals are dependant on oaks and hickories for food, including grackles, turkey, grouse, quail, blue jays, woodpeckers, waterfowl, deer, bear, squirrels, mice, rabbits, foxes, and raccoons. Because wildlife populations vary with the availability of acorns, populations of acorn-dependent species decrease in forests where Amur Cork Tree has become established (https://www.invasive.org/alien/fact/pham1.htm).	Medium
25	4.12	Is the taxon likely to maintain a viable population even when present in low densities (or persist in adverse conditions by way of a dormant form)?	Yes	The drupes persist on the tree through the winter (https://plants.ces.ncsu.edu/plants/phellodendron-amurense/).	Low
5. Resource exploitation					
26	5.01	Is the taxon likely to put threatened or protected native species under competitive pressure in the risk assessment area?	Yes	professional judgement	Low
27	5.02	Is the taxon likely to sequester resources to the detriment of native species in the risk assessment area?	Yes	professional judgement	Low
6. Reproduction					
28	6.01	Is the taxon likely to exhibit changes in reproductive strategy in response to environmental conditions?	No	no evidence	Low
29	6.02	Is the taxon likely to produce viable propagules in the risk assessment area?	Yes	The female tree can produces thousands of seeds (https://plants.ces.ncsu.edu/plants/phellodendron-amurense/).	High
30	6.03	Is the taxon likely to hybridise with native species under natural conditions?	No	no evidence	Low
31	6.04	Is the taxon likely to be hermaphroditic or to exhibit asexual reproduction?	No	no evidence	Low
32	6.05	Is the taxon dependent on the presence of another species (or specific habitat features) to complete its life cycle?	Yes	Arbuscular mycorrhizal fungi were isolated from rhizospheric soil samples collected from Phellodendron amurense (doi: 10.1007/s13213-012-0435-6).	High
33	6.06	Is the taxon likely to produce a large number of propagules?	Yes	The female tree can produces thousands of seeds (https://plants.ces.ncsu.edu/plants/phellodendron-amurense/).	Very high
34	6.07	How many time units (days, months, years) does the taxon require to reach the age at first reproduction?	5	Amur Cork Tree reaches reproductive maturity at about 3 to 5 years of age (https://www.invasive.org/alien/fact/pham1.htm).	High
7. Dispersal mechanisms					
35	7.01	How many potential vectors or pathways could the taxon use to disperse within the risk assessment area (with suitable habitats nearby)?	>1	The trees produce abundant seed which is dispersed by birds and probably by water via streams and other drainages (https://www.invasive.org/alien/fact/pham1.htm). P. amurense is also dispersed by humans.	High
36	7.02	Will any of these vectors or pathways bring the taxon in close proximity to one or more protected areas?	Yes	spreading by birds and probably by water via streams and other drainages (https://www.invasive.org/alien/fact/pham1.htm).	High
37	7.03	Does the taxon have a specialised means of attachment such that it enhances the likelihood of dispersal?	No	no evidence	Low
38	7.04	Is natural dispersal of the taxon likely to occur as seeds or spores in the risk assessment area?	Yes	The tree can produces thousands of seeds which are dispersed by birds and probably by water via streams and other drainages (https://www.invasive.org/alien/fact/pham1.htm).	High
39	7.05	Is natural dispersal of the taxon likely to occur as fragments or seedlings in the risk assessment area?	Yes	by seedlings, with human intervention	Medium
40	7.06	Are any life stages of the taxon likely to migrate into the risk assessment area for reproduction?	Yes	The seeds can be dispersed by birds and probably by water via streams and other drainages (https://www.invasive.org/alien/fact/pham1.htm).	High
41	7.07	Are propagules of the taxon likely to be dispersed in the risk assessment area by other species?	Yes	The seeds can be dispersed by birds, squirrels and other mammals (Gilman and Watson 1994).	High
42	7.08	Is dispersal of the taxon along any of the vectors or pathways mentioned in the previous seven Questions (35–41: i.e. either unintentional or intentional) likely to be	Yes	dispersal by birds, squirrels and other mammals	High
43	7.09	Is dispersal of the taxon density dependent?	Yes	more trees have more seeds	Low
8. Tolerance attributes					
44	8.01	Is the taxon able to withstand being in water for extended periods at some stage of its life cycle?	No	The seed may remain viable in the soil for a number of years (https://www.invasive.org/alien/fact/pham1.htm).	Low
45	8.02	Is the taxon tolerant of a wide range of soil or air quality conditions?	Yes	Its suitability for soil is extremely strong (doi: 10.3389/fevo.2023.1186627).	High

46	8.03	Can the taxon be controlled or eradicated in the wild with chemical, biological or other agents and means?	Yes	https://www.invasive.org/alien/fact/pham1.htm	High
47	8.04	Is the taxon likely to benefit from environmental or human disturbance?	Yes	The seeds can be dispersed by water via streams and other drainages (https://www.invasive.org/alien/fact/pham1.htm).	Medium
48	8.05	Is the taxon able to tolerate soil acidity, salinity or other parameter levels that are higher or lower than those found in its usual	Yes	It can tolerate periods of dry soils and a wide range of soil pH from 5.0 to 8.2 (https://www.invasive.org/alien/fact/pham1.htm).	High
49	8.06	Are there effective natural enemies of the taxon present in the risk assessment area?	No	professional judgement	Low
**C. Climate change**					
9. Climate change					
50	9.01	Under the predicted future climatic conditions, are the risks of entry into the risk assessment area posed by the taxon likely to increase, decrease or not change?	Increase	professional judgement	Low
51	9.02	Under the predicted future climatic conditions, are the risks of establishment within the risk assessment area posed by the taxon likely to increase, decrease or not	Increase	professional judgement	Low
52	9.03	Under the predicted future climatic conditions, are the risks of dispersal within the risk assessment area posed by the taxon likely to increase, decrease or not change?	Increase	professional judgement	Low
53	9.04	Under the predicted future climatic conditions, what is the likely magnitude of future potential impacts by the taxon on biodiversity or ecosystem integrity and	Higher	professional judgement	Low
54	9.05	Under the predicted future climatic conditions, what is the likely magnitude of future potential impacts by the taxon on ecosystem structure or function?	Higher	professional judgement	Low
55	9.06	Under the predicted future climatic conditions, what is the likely magnitude of future potential impacts by the taxon on ecosystem services and related socio-	Higher	professional judgement	Low

Statistics	
**Scores**	
**BRA**	**46.0**
**BRA Outcome**	**High**
**BRA+CCA**	**58.0**
**BRA+CCA Outcome**	**High**
**Score partition**	
**A. Biogeography/Historical**	**21.0**
1. Domestication/Cultivation	*2.0*
2. Climate, distribution and introduction risk	*1.0*
3. Invasive elsewhere	*18.0*
**B. Biology/Ecology**	**25.0**
4. Undesirable (or persistence) traits	*8.0*
5. Resource exploitation	*7.0*
6. Reproduction	*-2.0*
7. Dispersal mechanisms	*7.0*
8. Tolerance attributes	*5.0*
**C. Climate change**	**12.0**
9. Climate change	*12.0*
**Answered Questions**	
Total	***55***
**A. Biogeography/Historical**	**13**
1. Domestication/Cultivation	*3*
2. Climate, distribution and introduction risk	*5*
3. Invasive elsewhere	*5*
**B. Biology/Ecology**	**36**
4. Undesirable (or persistence) traits	*12*
5. Resource exploitation	*2*
6. Reproduction	*7*
7. Dispersal mechanisms	*9*
8. Tolerance attributes	*6*
**C. Climate change**	**6**
9. Climate change	*6*
**Sectors affected**	
**Commercial**	**20**
**Environmental**	**17**
**Species or population nuisance traits**	**25**

Thresholds	
**BRA**	**41**
**BRA+CCA**	**41**
**Confidence**	
**BRA+CCA**	**0.47**
**BRA**	**0.50**
**CCA**	**0.25**

Date and Time
**27/06/2024 10:47:00**

TPS-ISK v2

Taxon and Assessor details	
Category	Angiosperms
Taxon name	*Populus simonii*
Common name	Simon Poplar
Assessor	Mihaela Britvec
**Risk screening context**	
Reason and socio-economic benefits	
Risk assessment area	Continental Europe
Taxonomy	
Native range	
Introduced range	
URL	

			Response	Justification (references or other information)	Confidence
**A. Biogeography/Historical**					
1. Domestication/Cultivation					
1	1.01	Has the taxon been the subject of domestication?	Yes	Populus simonii is a species of poplar native to northeast China and to Mongolia, and commonly planted as a street tree in cool temperate areas of Europe (https://en.wikipedia.org/wiki/Populus_simonii). Into France was	High
2	1.02	Is the taxon harvested in the wild and likely to be sold or used in its live form?	Yes	Populus simonii is cultivated in horticulture (Bailey 1947).	High
3	1.03	Does the taxon have invasive races, varieties, sub-taxa or congeners?	Yes	For example: Populus alba and P. nigra (https://www.cabidigitallibrary.org/action/doSearch?AllField=populus&ConceptID=5000609.	Very high
2. Climate, distribution and introduction risk					
4	2.01	How similar are the climatic conditions between the risk assessment area and the taxon's native range?	Medium	Populus simonii grows in cool temperate areas such as those found in Continental Europe.	Low
5	2.02	What is the quality of the climate matching data?	Low	professional judgement	Low
6	2.03	Is the taxon known to be present outside of cultivation in the risk assessment area?	Yes	https://www.nobanis.org/species-info/?taxaId=3357	Medium
7	2.04	How many potential vectors could the taxon use to enter the risk assessment area?	>1	dispersion by wind and by humans	High
8	2.05	Is the taxon currently found in close proximity to, and likely to enter, the risk assessment area in the near future (e.g. unintentional or intentional introductions)?	Not applicable	For example, Populus simonii was established in Sweden (https://www.nobanis.org/species-info/?taxaId=3357).	Very high
3. Invasive elsewhere					
9	3.01	Has the taxon become naturalised outside its native range?	Yes	The species was established in Sweden (https://www.nobanis.org/species-info/?taxaId=3357).	Very high
10	3.02	In the taxon's introduced range, are there any known adverse impacts to wild or commercial species?	No	no evidence	Low
11	3.03	In the taxon's introduced range, are there any known adverse impacts to agriculture or forestry?	No	no evidence	Low
12	3.04	In the taxon's introduced range, are there any known adverse impacts to ecosystem	No	no evidence	Low
13	3.05	In the taxon's introduced range, are there any known adverse socio-economic impacts?	No	no evidence	Low
**B. Biology/Ecology**					
4. Undesirable (or persistence) traits					
14	4.01	Is the taxon likely to be poisonous or pose other risks to human health?	No	Populus simonii is suitable for new planting from an allergological perspective (Bergmann et al. 2012).	High
15	4.02	Is the taxon likely to suppress the growth of one or more native species?	Yes	It is very rapid grower (Bailey 1947).	Medium
16	4.03	Are there any threatened or protected native species that the taxon would parasitise in the risk assessment area?	No	no evidence	Low
17	4.04	Is the taxon adaptable in terms of climatic and other environmental conditions, thus enhancing its potential persistence if it has invaded or is likely to invade the risk	Yes	professional judgement	Low
18	4.05	Is the taxon likely to disrupt terrestrial ecosystem function if it has invaded or is likely to invade the risk assessment area?	Yes	professional judgement	Low
19	4.06	Is the taxon likely to exert adverse impacts on ecosystem services in the risk	Yes	professional judgement	Low
20	4.07	Is the taxon likely to host or function as a vector for recognised pests and pathogens that are present in the risk assessment area?	Yes	https://bladmineerders.nl/host-plants/plantae/spermatopsida/angiosperma/eudicots/superrosids/rosids/fabids/malpighiales/salicaceae/populus/populus-simonii/	Very high
21	4.08	Is the taxon likely to host or function as a vector for recognised pests and pathogens that are absent in the risk assessment area?	Yes	professional judgement	Low
22	4.09	Is the taxon likely to be released from cultivation?	Yes	Populus simoniii is very rapid grower (Bailey 1947).	High
23	4.10	Is the taxon versatile in habitat use?	Yes	https://www.trees-sa.co.za/tree/populus-simonii/	Low
24	4.11	Is it likely that the taxon's mode of existence or behaviours will reduce habitat quality for native species?	No	usually not toxic to livestock (https://www.vdberk.com/trees/populus-simonii/)	Low

25	4.12	Is the taxon likely to maintain a viable population even when present in low densities (or persist in adverse conditions by way of a dormant form)?	Yes	professional judgement	Low
5. Resource exploitation					
26	5.01	Is the taxon likely to put threatened or protected native species under competitive pressure in the risk assessment area?	Yes	professional judgement	Low
27	5.02	Is the taxon likely to sequester resources to the detriment of native species in the risk assessment area?	Yes	professional judgement	Low
6. Reproduction					
28	6.01	Is the taxon likely to exhibit changes in reproductive strategy in response to environmental conditions?	No	no evidence	Low
29	6.02	Is the taxon likely to produce viable propagules in the risk assessment area?	Yes	Its fruit is capsule, containing numerous seeds surrounded by tufts of long, soft, white hairs aiding wind dispersal.	High
30	6.03	Is the taxon likely to hybridise with native species under natural conditions?	Yes	It is known that genus Populus contains hybrids.	Low
31	6.04	Is the taxon likely to be hermaphroditic or to exhibit asexual reproduction?	Yes	Trees in the genus Populus can generally reproduce vegetatively by natural and artificial means.	Low
32	6.05	Is the taxon dependent on the presence of another species (or specific habitat features) to complete its life cycle?	No	Poplar roots can develop mycorrhizal associations with ectomycorrhizal and arbuscular mycorrhizal fungi, and with non-mycorrhizal fungal endophytes (https://doi.org/10.1007/s11056-021-09879-6).	Medium
33	6.06	Is the taxon likely to produce a large number of propagules?	Yes	Populus species set lots of wind dispersed seeds. A mature poplar can produce up to 50 million seeds (Fransson 2016).	Very high
34	6.07	How many time units (days, months, years) does the taxon require to reach the age at first reproduction?	7	The first flowering occurs at the age of about five to ten years.	High
7. Dispersal mechanisms					
35	7.01	How many potential vectors or pathways could the taxon use to disperse within the risk assessment area (with suitable habitats nearby)?	>1	dispersion by wind and by humans	High
36	7.02	Will any of these vectors or pathways bring the taxon in close proximity to one or more protected areas?	Yes	Poplar seeds can travel with the wind for over 10 km (Fransson 2016).	High
37	7.03	Does the taxon have a specialised means of attachment such that it enhances the likelihood of dispersal?	No	no evidence	Medium
38	7.04	Is natural dispersal of the taxon likely to occur as seeds or spores in the risk	Yes	Poplar seeds can travel with the wind (Fransson 2016).	Very high
39	7.05	Is natural dispersal of the taxon likely to occur as fragments or seedlings in the risk assessment area?	Yes	Poplar trees are planted as cuttings (https://www.ndsu.edu/pubweb/chiwonlee/plsc368/student/papers01/khovet/index.htm).	High
40	7.06	Are any life stages of the taxon likely to migrate into the risk assessment area for reproduction?	Yes	Poplar seeds can travel with the wind for over 10 km (Fransson 2016).	Very high
41	7.07	Are propagules of the taxon likely to be dispersed in the risk assessment area by other species?	No	no evidence	Low
42	7.08	Is dispersal of the taxon along any of the vectors or pathways mentioned in the previous seven Questions (35–41: i.e. either unintentional or intentional) likely to be	Yes	dispersion by wind	High
43	7.09	Is dispersal of the taxon density dependent?	Yes	more mature trees have more seeds	Low
8. Tolerance attributes					
44	8.01	Is the taxon able to withstand being in water for extended periods at some stage of its life cycle?	Yes	professional judgement	Low
45	8.02	Is the taxon tolerant of a wide range of soil or air quality conditions?	Yes	For example, this species can tolerate heavy clay soil (https://www.trees-sa.co.za/tree/populus-simonii/).	Low
46	8.03	Can the taxon be controlled or eradicated in the wild with chemical, biological or other agents and means?	Yes	https://www.poplarandwillow.org.nz/documents/killing-old-poplar-trees-using-chemicals.pdf	High
47	8.04	Is the taxon likely to benefit from environmental or human disturbance?	Yes	Poplar trees are fire-resistant (https://northstartreeservice.com/make-your-landscape-fire-	Medium
48	8.05	Is the taxon able to tolerate soil acidity, salinity or other parameter levels that are higher or lower than those found in its usual	Yes	https://envirocrops.com/resource/poplar-best-practice-guidelines	Low
49	8.06	Are there effective natural enemies of the taxon present in the risk assessment area?	No	no evidence	Low
**C. Climate change**					
9. Climate change					
50	9.01	Under the predicted future climatic conditions, are the risks of entry into the risk assessment area posed by the taxon likely to increase, decrease or not change?	No change	professional judgement	Low
51	9.02	Under the predicted future climatic conditions, are the risks of establishment within the risk assessment area posed by the taxon likely to increase, decrease or not	No change	professional judgement	Low

52	9.03	Under the predicted future climatic conditions, are the risks of dispersal within the risk assessment area posed by the taxon likely to increase, decrease or not change?	No change	professional judgement	Low
53	9.04	Under the predicted future climatic conditions, what is the likely magnitude of future potential impacts by the taxon on biodiversity or ecosystem integrity and	No change	professional judgement	Low
54	9.05	Under the predicted future climatic conditions, what is the likely magnitude of future potential impacts by the taxon on ecosystem structure or function?	No change	professional judgement	Low
55	9.06	Under the predicted future climatic conditions, what is the likely magnitude of future potential impacts by the taxon on ecosystem services and related socio-	No change	professional judgement	Low

**Statistics**	
**Scores**	
**BRA**	**40.0**
**BRA Outcome**	**Medium**
**BRA+CCA**	**40.0**
**BRA+CCA Outcome**	**Medium**
**Score partition**	
**A. Biogeography/Historical**	**8.0**
1. Domestication/Cultivation	*4.0*
2. Climate, distribution and introduction risk	*2.0*
3. Invasive elsewhere	*2.0*
**B. Biology/Ecology**	**32.0**
4. Undesirable (or persistence) traits	*9.0*
5. Resource exploitation	*7.0*
6. Reproduction	*4.0*
7. Dispersal mechanisms	*5.0*
8. Tolerance attributes	*7.0*
**C. Climate change**	**0.0**
9. Climate change	*0.0*
**Answered Questions**	
Total	***55***
**A. Biogeography/Historical**	**13**
1. Domestication/Cultivation	*3*
2. Climate, distribution and introduction risk	*5*
3. Invasive elsewhere	*5*
**B. Biology/Ecology**	**36**
4. Undesirable (or persistence) traits	*12*
5. Resource exploitation	*2*
6. Reproduction	*7*
7. Dispersal mechanisms	*9*
8. Tolerance attributes	*6*
**C. Climate change**	**6**
9. Climate change	*6*
**Sectors affected**	
**Commercial**	**7**
**Environmental**	**8**
**Species or population nuisance traits**	**30**

**Thresholds**	
**BRA**	**41**
**BRA+CCA**	**41**
**Confidence**	
**BRA+CCA**	**0.48**
**BRA**	**0.51**
**CCA**	**0.25**

**Date and Time**
**28/06/2024 11:21:29**

TPS-ISK v2

Taxon and Assessor details	
Category	Angiosperms
Taxon name	*Prunus serrulata*
Common name	Japanese Cherry
Assessor	Ivana Vitasović Kosić
**Risk screening context**	
Reason and socio-economic benefits	
Risk assessment area	Continental Europe
Taxonomy	
Native range	
Introduced range	
URL	

			Response	Justification (references or other information)	Confidence
**A. Biogeography/Historical**					
1. Domestication/Cultivation					
1	1.01	Has the taxon been the subject of domestication?	Yes	- Cerasus serrulata is cultivated a very long time for its flowers - Yi XG, Yu XQ, Chen J, Zhang M, Liu SW, Zhu H, Li M, Duan YF, Chen L, Wu L, Zhu S, Sun ZS, Liu XH, Wang XR (2020) The genome of Chinese flowering cherry (Cerasus serrulata) provides new insights into Cerasus species. Hortic Res 7:165. https://doi.org/10.1038/s41438-020-00382-1 but still - Prunus serrulata is species with deficient data for analysis (screening) – no other available data: Bayón, Á., & Vilà, M. (2019). Horizon scanning to identify invasion risk of ornamental plants marketed in Spain. NeoBiota, 52, 47-86. Bayón Medrano, Á., Godoy, Ó., & Vilà, M. (2022). Invasion risks and social interest of non-native woody plants in urban parks of mainland Spain. Anales del Jardin Botanico de Madrid, 79 (1), e121. This iconic flowering tree is a member of the Rosaceae or rose family. This tree is native to East and South China, Japan, and Korea. Cherry blossoms are the national flower of Japan and symbolize life, good health, and happiness. The genus name, Prunus, is derived from the Latin word meaning plum or cherry tree. Its potential as an invasive species is low, as it is susceptible to many common garden	Very high
2	1.02	Is the taxon harvested in the wild and likely to be sold or used in its live form?	Yes	yes, it is horticultural economic plant. The damage and loss of wild Cerasus plant genetic resources are very serious (Chen et al. 2020). Therefore, it is important to protect and collect these resources. Chen T, Hu GP, Yang Y, Chen Q, Zhang J, Wang L, Tang HR, Wang XR (2020) Survey, collection and conservation of wild Cerasus Mill. germplasm resources in China. J Plant Genet Resour	Very high
3	1.03	Does the taxon have invasive races, varieties, sub-taxa or congeners?	No	Its potential as an invasive species is low, as it is susceptible to many common garden diseases and pests such as fire blight, powdery mildew and Japanese beetles. https://www.gardenia.net/genus/prunus-serrulata-japanese-flowering-cherry In 2 paper below said: Prunus serrulata is species with deficient data for analysis (screening) – no other available data: Bayón, Á., & Vilà, M. (2019). Horizon scanning to identify invasion risk of ornamental plants marketed in Spain. NeoBiota, 52, 47-86. Bayón Medrano, Á., Godoy, Ó., & Vilà, M. (2022). Invasion risks and social interest of non-native woody plants in urban parks of mainland Spain. Anales del Jardin Botanico de	High
2. Climate, distribution and introduction risk					
4	2.01	How similar are the climatic conditions between the risk assessment area and the taxon's native range?	Medium	The climate of Japan is similar to the climate of the east coast of the United States. Both regions have a humid subtropical climate, characterized by hot, humid summers and mild winters. This type of climate is influenced by warm ocean currents and the prevailing	High
5	2.02	What is the quality of the climate matching data?	High	https://www.worlddata.info/country-comparison.php?country1=HRV&country2=JPN	High
6	2.03	Is the taxon known to be present outside of cultivation in the risk assessment area?	Yes	P.serrulata is already present in almoust all countries of EU www.gbif.org/species/3022609 but it is not invasive.	Very high
7	2.04	How many potential vectors could the taxon use to enter the risk assessment area?	One	Świerczyński, S., & Stachowiak, A. (2007). Propagation efficiency of four cultivars of Prunus serrulata (Lindl.) on two rootstocks. Nauka Przyroda Technologie, 1(3), 45. There are very few other ornamental trees that bloom more beautiful than cultivars of Prunus serrulata. Their blossom is abundant and the flowers are especially varied. Ornamental cherry trees are becoming more and more popular and their offer is expanding. Most of them are propagated by grafting on P. avium rootstock. The aim of these studies was an evaluation of propagation of four grafted cultivars of P. serrulata on two rootstocks in nursery production.	Very high
8	2.05	Is the taxon currently found in close proximity to, and likely to enter, the risk assessment area in the near future (e.g. unintentional or intentional introductions)?	Not applicable	already present www.gbif.org/species/3022609	Very high
3. Invasive elsewhere					
9	3.01	Has the taxon become naturalised outside its native range?	No	Most of them are propagated by grafting on P. avium rootstock. Świerczyński, S., & Stachowiak, A. (2007). Propagation efficiency of four cultivars of Prunus serrulata (Lindl.) on two rootstocks. Nauka Przyroda Technologie, 1(3), 45.	High
10	3.02	In the taxon's introduced range, are there any known adverse impacts to wild or commercial species?	No	it is not known, i didn't found in literature.	Medium

11	3.03	In the taxon's introduced range, are there any known adverse impacts to agriculture or forestry?	No	While pets may be at risk, research indicates that Japanese Flowering Cherry trees pose no significant threat to humans. The concerns often stem from the tree's leaves, stems, and seeds, which contain compounds toxic to animals. However, these same components have not been shown to have harmful effects on people., cherry blossom leaves and fruits contain poisonous cyanide, especially the unripe berries. https://www.gardenia.net/genus/prunus-serrulata-japanese-	Medium
12	3.04	In the taxon's introduced range, are there any known adverse impacts to ecosystem	No	no available literature data.	Medium
13	3.05	In the taxon's introduced range, are there any known adverse socio-economic impacts?	No	my opinion is no, no data.	Medium
**B. Biology/Ecology**					
4. Undesirable (or persistence) traits					
14	4.01	Is the taxon likely to be poisonous or pose other risks to human health?	Yes	https://www.gardenia.net/genus/prunus-serrulata-japanese-flowering-cherry leaves, seeds and stam toxic to cats, dogs, horses, but for humans no. Even the opposite: the anti-inflammatory effect of cherry blossom extract (Prunus yedoensis) used in soothing skincare product, Y Q Zhang 1, L Guan, Z Y Zhong, M Chang, D K Zhang, H Li, W Lai DOI: 10.1111/ics.12149 Cherry blossom extract shows good anti-inflammatory effect in vitro and in vivo and represents a promising functional ingredient in soothing skincare product by reducing skin inflammation.	Low
15	4.02	Is the taxon likely to suppress the growth of one or more native species?	No	my opinion	Medium
16	4.03	Are there any threatened or protected native species that the taxon would parasitise in the risk assessment area?	No	Its potential as an invasive species is low, as it is susceptible to many common garden diseases and pests such as fire blight, powdery mildew and Japanese beetles.	High
17	4.04	Is the taxon adaptable in terms of climatic and other environmental conditions, thus enhancing its potential persistence if it has invaded or is likely to invade the risk	No	it is spreading moustly by grafting, so vegetatively no.	High
18	4.05	Is the taxon likely to disrupt terrestrial ecosystem function if it has invaded or is likely to invade the risk assessment area?	No	there is no possibility of establishing a self-sustaining population, beside In the future, climate change may lead to a slight increase of 2.31% in the area of suitable habitat for Cerasus serrulata, while the optimal habitat will decrease from 20.81 to 14.55%. Therefore, conservation measures should be taken to protect these precious resources. from: Zhang, L., Huang, S., Yuan, Y. et al. Geographical distribution and predict potential distribution of Cerasus serrulata. Environ Sci Pollut Res 30, 43369–43376 (2023). https://doi.org/10.1007/s11356-023-25282-4	High
19	4.06	Is the taxon likely to exert adverse impacts on ecosystem services in the risk	No	there are no such indications.	Medium
20	4.07	Is the taxon likely to host or function as a vector for recognised pests and pathogens that are present in the risk assessment area?	Yes	EFSA Panel on Plant Health (PLH), Jeger, M., Bragard, C., Caffier, D., Dehnen-Schmutz, K., Gilioli, G., ... & Candresse, T. (2017). Pest categorisation of Little cherry pathogen (non-EU isolates). EFSA Journal, 15(7), e04926. Little cherry virus 1(LChV) are transmitted by vegetative multiplication of infected hosts and, for LChV2, by mealybug vectors. LChV are reported from a range of countries, both outside and within the EU. Non-EU isolates are not known to occur in the EU and therefore do not meet one of the criteria for being a Union regulated non-quarantine pest. The host ranges of LChV are restricted to Prunus species, in particular cultivated and ornamental cherries. Little cherry pathogen (non-European isolates) on Prunus serrulata Lindl.	Very high
21	4.08	Is the taxon likely to host or function as a vector for recognised pests and pathogens that are absent in the risk assessment area?	Yes	it is susceptible to many common garden diseases and pests such as fire blight, powdery mildew and Japanese beetles....	Medium
22	4.09	Is the taxon likely to be released from cultivation?	Not applicable	generative reproduction is not common in this species, only it reproduces by grafts.	Medium
23	4.10	Is the taxon versatile in habitat use?	No	Zhang, L., Huang, S., Yuan, Y. et al. Geographical distribution and predict potential distribution of Cerasus serrulata. Environ Sci Pollut Res 30, 43369–43376 (2023). https://doi.org/10.1007/s11356-023-25282-4 he MaxEnt model was used to simulate the current and future distribution, and the ecological distribution and richness of C. serrulata were analyzed. It grows mainly in the low and middle elevation areas of 10 to 1200 m. Based on this model, the precipitation of the warmest quarter, the precipitation of the driest month, and the mean temperature of the coldest were the most significant bioclimatic variables affecting the distribution of C. serrulata. In the future, climate change may lead to a slight increase of 2.31% in the area of suitable habitat for Cerasus serrulata, while the optimal habitat will decrease from 20.81 to 14.55%. Therefore, conservation measures should be taken to protect these precious resources. However, due to the unreasonable use of wild resources and the influence of environmental changes, the population of C. serrulata has declined and is rarely distributed in the western alpine areas	High
24	4.11	Is it likely that the taxon's mode of existence or behaviours will reduce habitat quality for native species?	Yes	https://www.gardenia.net/genus/prunus-serrulata-japanese-flowering-cherry leaves, stema dn seeds are toxic to cats, dogs and hoses.	Medium
25	4.12	Is the taxon likely to maintain a viable population even when present in low densities (or persist in adverse conditions by way of a dormant form)?	No	no data about.	Low

5. Resource exploitation					
26	5.01	Is the taxon likely to put threatened or protected native species under competitive pressure in the risk assessment area?	No	my opinion.	Medium
27	5.02	Is the taxon likely to sequester resources to the detriment of native species in the risk assessment area?	No	no, i believe no, it is cutivated species, it reproduces only with human assistence by grafts, no vegetatively, no generatively.	Medium
6. Reproduction					
28	6.01	Is the taxon likely to exhibit changes in reproductive strategy in response to environmental conditions?	No	only in urban environment , in nature this taxa is in risk. Ninić-Todorović, J., Ognjanov, V., Čukanović, J., Kurjakov, A., Mladenović, E., Ljubojević, M., & Dulić, J. (2012). ORNAMENTAL PRUNUS TAXONS (PRUNUS SERRULATA LINDL. AND PRUNUS FRUTICOSA PALL.'GLOBOSA') ON NOVI SAD GREEN AREAS. ECOLOGY OF URBAN AREAS 2012, 386. Both taxa showed high scores of vitality and decorativeness, indicating high level of adaptiveness to changing conditions of urban environment.	Medium
29	6.02	Is the taxon likely to produce viable propagules in the risk assessment area?	No	I'm not sure, no data. In 2 paper below said: Prunus serrulata is species with deficient data for analysis (screening) – no other available data: Bayón, Á., & Vilà, M. (2019). Horizon scanning to identify invasion risk of ornamental plants marketed in Spain. NeoBiota, 52, 47-86. Bayón Medrano, Á., Godoy, Ó., & Vilà, M. (2022). Invasion risks and social interest of non-native woody plants in urban parks of mainland Spain. Anales del Jardin Botanico de Madrid, 79 (1), e121.	Low
30	6.03	Is the taxon likely to hybridise with native species under natural conditions?	No	Even reproduction without human assistence is hardly possible.	Medium
31	6.04	Is the taxon likely to be hermaphroditic or to exhibit asexual reproduction?	No	I found data for similar species P.padus: Prunus padus is a plant which has both hermaphroditic flowers and is pollinated by insects. Hermaphroditic flower plants are known to have mixed mating and if ... Ahn, J. Y., Lee, J. W., Lim, H. I., & Hong, K. N. (2020). Genetic diversity and structure of Prunus padus populations in South Korea based on AFLP markers. Forest Science and Technology, 16(4), 171-179. Uusitalo, M. (2004). European bird cherry (Prunus padus L.)-a biodiverse wild plant for	Medium
32	6.05	Is the taxon dependent on the presence of another species (or specific habitat features) to complete its life cycle?	No	it is not known.	Low
33	6.06	Is the taxon likely to produce a large number of propagules?	No	In some cultivars, the pistil changes like a leaf and loses its fertility, and for example, 'Fugenzo' and 'Ichiyo', can only be propagated by artificial methods such as grafting and cutting. Toshio Katsuki. (2015) Sakura p.107 Iwanami Shoten. ISBN 978-4004315346 Unfortunately, for all their display, the flowers do not give rise to fertile seeds as Kwanzan trees are sterile. Propagation of this cultivar of Japanese cherry tree is by cloning only. The flowers are either fragrant or non-fragrant, depending on the individual tree. Occasionally Kwanzans may produce a small black cherry, which is technically a drupe.	High
34	6.07	How many time units (days, months, years) does the taxon require to reach the age at first reproduction?	>10	This tree is usually short-lived and average life span is 15 to 20 years. There are many cultivars available with bloom colors ranging from white, pale pink.	High
7. Dispersal mechanisms					
35	7.01	How many potential vectors or pathways could the taxon use to disperse within the risk assessment area (with suitable habitats nearby)?	None	it is very hardly reproducing without human assistence. P serrulata don't produce fruit. From: Russell, P. (1928). Japanese flowering cherries (No. 31). US Department of Agriculture.	Medium
36	7.02	Will any of these vectors or pathways bring the taxon in close proximity to one or more protected areas?	No	my opinion.	Medium
37	7.03	Does the taxon have a specialised means of attachment such that it enhances the likelihood of dispersal?	No	no,the fruit is like all other Punus - drupe (koštunica). P serrulata don't produce fruit. From: Russell, P. (1928). Japanese flowering cherries (No. 31). US Department of Agriculture.	High
38	7.04	Is natural dispersal of the taxon likely to occur as seeds or spores in the risk assessment area?	No	only with human assistence, it is cultivated, and often sterile. P serrulata don't produce fruit. From: Russell, P. (1928). Japanese flowering cherries (No. 31). US Department of Agriculture.	High
39	7.05	Is natural dispersal of the taxon likely to occur as fragments or seedlings in the risk assessment area?	No	no, it is cultivated, sterile. P serrulata don't produce fruit. From: Russell, P. (1928). Japanese flowering cherries (No. 31). US Department of Agriculture.	High
40	7.06	Are any life stages of the taxon likely to migrate into the risk assessment area for reproduction?	No	it is cultivated and sterile. P serrulata don't produce fruit. From: Russell, P. (1928). Japanese flowering cherries (No. 31). US Department of Agriculture.	High
41	7.07	Are propagules of the taxon likely to be dispersed in the risk assessment area by other species?	No	no, it is cv and sterile. P serrulata don't produce fruit. From: Russell, P. (1928). Japanese flowering cherries (No. 31). US Department of Agriculture.	High
42	7.08	Is dispersal of the taxon along any of the vectors or pathways mentioned in the previous seven Questions (35–41: i.e. either unintentional or intentional) likely to be	No	no! P serrulata don't produce fruit. From: Russell, P. (1928). Japanese flowering cherries (No. 31). US Department of Agriculture.	High
43	7.09	Is dispersal of the taxon density dependent?	No	i believe no, my opinion,	Medium
8. Tolerance attributes					
44	8.01	Is the taxon able to withstand being in water for extended periods at some stage of its life cycle?	No	i believe no.	Low

45	8.02	Is the taxon tolerant of a wide range of soil or air quality conditions?	Yes	- It also has good effects in preventing soil erosion, regulating the microclimate of the ecosystem and improving the air condition, etc. (Dai et al. 2017). - strong adaptability and stress resistance. Distribution expands in - Lower altitude areas. Dai L, Lai A, Chao F et al (2017) A study on seed germination of Cerasus serrulata. South China Forest Sci 45(01):1–4+13. https://doi.org/10.16259/j.cnki.36-1342/s.2017.01.001 Zhang, L., Huang, S., Yuan, Y. et al. Geographical distribution and predict potential distribution of Cerasus serrulata. Environ Sci Pollut Res 30, 43369–43376 (2023). https://doi.org/10.1007/s11356-023-	High
46	8.03	Can the taxon be controlled or eradicated in the wild with chemical, biological or other agents and means?	Yes	However, due to the unreasonable use of wild resources and the influence of environmental changes, the population of C. serrulata has declined and is rarely distributed in the western alpine areas (Li et al. 2014; Qiu et al. 2021, from: Zhang, L., Huang, S., Yuan, Y. et al. Geographical distribution and predict potential distribution of Cerasus serrulata. Environ Sci Pollut Res 30, 43369–43376 (2023). https://doi.org/10.1007/s11356-023-25282-4). The damage and loss of wild Cerasus plant genetic resources are very serious (Chen et al. 2020). Therefore, it is important to protect and collect these resources. A combination of in situ conservation and ex situ conservation is recommended for this resource, establishing a long-term protection mechanism and increasing the investigation, development, and utilization of wild C. serrulata plant resources. In addition, in the breeding and cultivation of cherry garden varieties, the application of biotechnology and genome technology can cultivate excellent varieties more efficiently. from: Chen T, Hu GP, Yang Y, Chen Q, Zhang J, Wang L, Tang HR, Wang XR (2020) Survey, collection and conservation of wild Cerasus Mill. germplasm resources in China. J Plant Genet Resour 21:532–541. Its potential as an invasive species is low, as it is susceptible to many common	High
47	8.04	Is the taxon likely to benefit from environmental or human disturbance?	No	my opinion	Medium
48	8.05	Is the taxon able to tolerate soil acidity, salinity or other parameter levels that are higher or lower than those found in its usual environment?	No	P serrulata is mesophylic species, it can not survive without water, Balsamo, R. A., Bauer, A. M., Davis, S. D., & Rice, B. M. (2003). Leaf biomechanics, morphology, and anatomy of the deciduous mesophyte Prunus serrulata (Rosaceae) and the evergreen sclerophyllous shrub Heteromeles arbutifolia (Rosaceae). American Journal of Botany, 90(1), 72-77.	Medium
49	8.06	Are there effective natural enemies of the taxon present in the risk assessment area?	Yes	P serrulata is very sensitive to pests. Babayan, A. H. (2022). Pest species composition of fragrant trees in the parks and gardens of Yerevan city, Armenia.	High
**C. Climate change**					
9. Climate change					
50	9.01	Under the predicted future climatic conditions, are the risks of entry into the risk assessment area posed by the taxon likely to increase, decrease or not change?	Decrease	Zhang, L., Huang, S., Yuan, Y. et al. Geographical distribution and predict potential distribution of Cerasus serrulata. Environ Sci Pollut Res 30, 43369–43376 (2023). https://doi.org/10.1007/s11356-023-25282-4 It grows mainly in the low and middle elevation areas of 10 to 1200 m. Based on this model, the precipitation of the warmest quarter, the precipitation of the driest month, and the mean temperature of the coldest were the most significant bioclimatic variables affecting the distribution of C. serrulata. In the future, climate change may lead to a slight increase of 2.31% in the area of suitable habitat for Cerasus serrulata, while the optimal habitat will decrease from 20.81 to 14.55%. Therefore, conservation measures should be	High
51	9.02	Under the predicted future climatic conditions, are the risks of establishment within the risk assessment area posed by the taxon likely to increase, decrease or not change?	No change	Zhang, L., Huang, S., Yuan, Y. et al. Geographical distribution and predict potential distribution of Cerasus serrulata. Environ Sci Pollut Res 30, 43369–43376 (2023). https://doi.org/10.1007/s11356-023-25282-4 Ninić-Todorović, J., Ognjanov, V., Čukanović, J., Kurjakov, A., Mladenović, E., Ljubojević, M., & Dulić, J. (2012). ORNAMENTAL PRUNUS TAXONS (PRUNUS SERRULATA LINDL. AND PRUNUS FRUTICOSA PALL.'GLOBOSA') ON NOVI SAD GREEN AREAS. ECOLOGY OF URBAN AREAS 2012, 386. Phenological occurrences and resistance to pests and diseases were observed with purpose of economical production and application of planting material in horticulture and	High
52	9.03	Under the predicted future climatic conditions, are the risks of dispersal within the risk assessment area posed by the taxon likely to increase, decrease or not change?	Decrease	Zhang, L., Huang, S., Yuan, Y. et al. Geographical distribution and predict potential distribution of Cerasus serrulata. Environ Sci Pollut Res 30, 43369–43376 (2023). https://doi.org/10.1007/s11356-023-25282-4	High
53	9.04	Under the predicted future climatic conditions, what is the likely magnitude of future potential impacts by the taxon on biodiversity or ecosystem integrity and status?	No change	it is cultivated, no harmful species. Zhang, L., Huang, S., Yuan, Y. et al. Geographical distribution and predict potential distribution of Cerasus serrulata. Environ Sci Pollut Res 30, 43369–43376 (2023). https://doi.org/10.1007/s11356-023-25282-4 In 2 paper below said: Prunus serrulata is species with deficient data for analysis (screening) – no other available data: Bayón, Á., & Vilà, M. (2019). Horizon scanning to identify invasion risk of ornamental plants marketed in Spain. NeoBiota, 52, 47-86. Bayón Medrano, Á., Godoy, Ó., & Vilà, M. (2022). Invasion risks and social interest of non-native woody plants in urban parks of mainland Spain. Anales del Jardin Botanico de Madrid, 79 (1),	High

54	9.05	Under the predicted future climatic conditions, what is the likely magnitude of future potential impacts by the taxon on ecosystem structure or function?	No change	Ninić-Todorović, J., Ognjanov, V., Čukanović, J., Kurjakov, A., Mladenović, E., Ljubojević, M., & Dulić, J. (2012). ORNAMENTAL PRUNUS TAXONS (PRUNUS SERRULATA LINDL. AND PRUNUS FRUTICOSA PALL.'GLOBOSA') ON NOVI SAD GREEN AREAS. ECOLOGY OF URBAN AREAS 2012, 386. Zhang, L., Huang, S., Yuan, Y. et al. Geographical distribution and predict potential distribution of Cerasus serrulata. Environ Sci Pollut Res 30, 43369–43376 (2023). https://doi.org/10.1007/s11356-023-	High
55	9.06	Under the predicted future climatic conditions, what is the likely magnitude of future potential impacts by the taxon on ecosystem services and related socio-economic factors?	No change	from : Zhang, L., Huang, S., Yuan, Y. et al. Geographical distribution and predict potential distribution of Cerasus serrulata. Environ Sci Pollut Res 30, 43369–43376 (2023). https://doi.org/10.1007/s11356-023-25282-4 In the future, climate change may lead to a slight increase of 2.31% in the area of suitable habitat for Cerasus serrulata, while the optimal habitat will decrease from 20.81 to 14.55%. Therefore, conservation measures should be taken to protect these precious resources. However, due to the unreasonable use of wild resources and the influence of environmental changes, the population of C. serrulata has declined and is rarely distributed in the western alpine areas	High

Statistics	
**Scores**	
**BRA**	**-6.0**
**BRA Outcome**	**Low**
**BRA+CCA**	**-10.0**
**BRA+CCA Outcome**	**Low**
**Score partition**	
**A. Biogeography/Historical**	**3.0**
1. Domestication/Cultivation	*2.0*
2. Climate, distribution and introduction risk	*1.0*
3. Invasive elsewhere	*0.0*
**B. Biology/Ecology**	**-9.0**
4. Undesirable (or persistence) traits	*3.0*
5. Resource exploitation	*0.0*
6. Reproduction	*-2.0*
7. Dispersal mechanisms	*-6.0*
8. Tolerance attributes	*-4.0*
**C. Climate change**	**-4.0**
9. Climate change	*-4.0*
**Answered Questions**	
Total	***55***
**A. Biogeography/Historical**	**13**
1. Domestication/Cultivation	*3*
2. Climate, distribution and introduction risk	*5*
3. Invasive elsewhere	*5*
**B. Biology/Ecology**	**36**
4. Undesirable (or persistence) traits	*12*
5. Resource exploitation	*2*
6. Reproduction	*7*
7. Dispersal mechanisms	*9*
8. Tolerance attributes	*6*
**C. Climate change**	**6**
9. Climate change	*6*
**Sectors affected**	
**Commercial**	**3**
**Environmental**	**1**
**Species or population nuisance traits**	**-14**

Thresholds	
**BRA**	**41**
**BRA+CCA**	**41**
**Confidence**	
**BRA+CCA**	**0.65**
**BRA**	**0.63**
**CCA**	**0.75**

Date and Time
**06/08/2024 15:50:46**

TPS-ISK v2

**Taxon and Assessor details**	
Category	Angiosperms
Taxon name	*Pterocarya fraxinifolia*
Common name	Caucasian Wingnut
Assessor	Božena Mitić
**Risk screening context**	
Reason and socio-economic benefits	ornamental tree, firewood
Risk assessment area	Continental Europe
Taxonomy	fam. Juglandaceae; Basionym: Juglans fraxinifolia Poir.; Syn. Pterocarya pterocarpa
Native range	Caucasus, northwestern Iran, Turkey
Introduced range	north America, Europe, NZ
URL	

			Response	Justification (references or other information)	Confidence
**A. Biogeography/Historical**					
1. Domestication/Cultivation					
1	1.01	Has the taxon been the subject of domestication?	Yes	Idžojtić M. (2013): DENDROLOGIJA – cvijet, češer, plod, sjeme. Denona d.d. (In Croatian).; Idžojtić et al. (2010): Šumarski list 1–2, CXXXIV, 5-18.; Pterocarya fraxinifolia (Poir.) Spach in GBIF Secretariat (2023). GBIF Backbone Taxonomy. Checklist dataset https://doi.org/10.15468/39omei accessed via GBIF.org on 2024-08-01.	Very high
2	1.02	Is the taxon harvested in the wild and likely to be sold or used in its live form?	Yes	Yilmaz (2014): Journal of Science and Technology - C – Life Sciences and Biotechnology 3 (2): 39-44.	Very high
3	1.03	Does the taxon have invasive races, varieties, sub-taxa or congeners?	Yes	https://edis.ifas.ufl.edu/publication/ST533	Very high
2. Climate, distribution and introduction risk					
4	2.01	How similar are the climatic conditions between the risk assessment area and the taxon's native range?	Medium	Peel et al. 2007	Medium
5	2.02	What is the quality of the climate matching data?	Medium	Peel et al. 2007	Medium
6	2.03	Is the taxon known to be present outside of cultivation in the risk assessment area?	Yes	https://alienplantsbelgium.myspecies.info/content/pterocarya-fraxinifolia	Very high
7	2.04	How many potential vectors could the taxon use to enter the risk assessment area?	>1	eg. https://alienplantsbelgium.myspecies.info/content/pterocarya-fraxinifolia;	Very high
8	2.05	Is the taxon currently found in close proximity to, and likely to enter, the risk assessment area in the near future (e.g. unintentional or intentional introductions)?	Not applicable	https://alienplantsbelgium.myspecies.info/content/pterocarya-fraxinifolia	Very high
3. Invasive elsewhere					
9	3.01	Has the taxon become naturalised outside its native range?	Yes	https://alienplantsbelgium.myspecies.info/content/pterocarya-fraxinifolia; Pterocarya fraxinifolia (Poir.) Spach in GBIF Secretariat (2023). GBIF Backbone Taxonomy. Checklist dataset https://doi.org/10.15468/39omei accessed via GBIF.org on 2024-08-01.	Very high
10	3.02	In the taxon's introduced range, are there any known adverse impacts to wild or commercial species?	No	Professional judgement based on https://alienplantsbelgium.myspecies.info/content/pterocarya-fraxinifolia.	Medium
11	3.03	In the taxon's introduced range, are there any known adverse impacts to agriculture or forestry?	No	Professional judgement based on https://alienplantsbelgium.myspecies.info/content/pterocarya-fraxinifolia.	Medium
12	3.04	In the taxon's introduced range, are there any known adverse impacts to ecosystem services?	No	Professional judgement based on https://alienplantsbelgium.myspecies.info/content/pterocarya-fraxinifolia.	Medium
13	3.05	In the taxon's introduced range, are there any known adverse socio-economic impacts?	No	Professional judgement based on https://alienplantsbelgium.myspecies.info/content/pterocarya-fraxinifolia.	Medium
**B. Biology/Ecology**					
4. Undesirable (or persistence) traits					
14	4.01	Is the taxon likely to be poisonous or pose other risks to human health?	No	Sadighara et al. (2009): Interdisc Toxicol. 2009; Vol. 2(4): 254–256. doi: 10.2478/v10102-009-0023-1	High
15	4.02	Is the taxon likely to suppress the growth of one or more native species?	No	Professional judgement based on https://alienplantsbelgium.myspecies.info/content/pterocarya-fraxinifolia.	Medium
16	4.03	Are there any threatened or protected native species that the taxon would parasitise in the risk assessment area?	No	No exact references found (professional judgement)	Medium
17	4.04	Is the taxon adaptable in terms of climatic and other environmental conditions, thus enhancing its potential persistence if it has invaded or is likely to invade the risk	Yes	https://alienplantsbelgium.myspecies.info/content/pterocarya-fraxinifolia	Medium
18	4.05	Is the taxon likely to disrupt terrestrial ecosystem function if it has invaded or is likely to invade the risk assessment area?	No	https://alienplantsbelgium.myspecies.info/content/pterocarya-fraxinifolia	Medium
19	4.06	Is the taxon likely to exert adverse impacts on ecosystem services in the risk	Yes	https://alienplantsbelgium.myspecies.info/content/pterocarya-fraxinifolia	Medium
20	4.07	Is the taxon likely to host or function as a vector for recognised pests and pathogens that are present in the risk assessment area?	Yes	Voglmayr, H., Jaklitsch, W.M., Mohammadi, H. et al. The genus Juglanconis (Diaporthales) on Pterocarya. Mycol Progress 18, 425–437 (2019). https://doi.org/10.1007/s11557-018-01464-0	Very high
21	4.08	Is the taxon likely to host or function as a vector for recognised pests and pathogens that are absent in the risk assessment area?	Yes	Voglmayr, H., Jaklitsch, W.M., Mohammadi, H. et al. The genus Juglanconis (Diaporthales) on Pterocarya. Mycol Progress 18, 425–437 (2019). https://doi.org/10.1007/s11557-018-01464-0	Very high

22	4.09	Is the taxon likely to be released from cultivation?	Yes	https://alienplantsbelgium.myspecies.info/content/pterocarya-fraxinifolia	Very high
23	4.10	Is the taxon versatile in habitat use?	No	https://alienplantsbelgium.myspecies.info/content/pterocarya-fraxinifolia; https://en.wikipedia.org/wiki/Pterocarya_fraxinifolia	High
24	4.11	Is it likely that the taxon's mode of existence or behaviours will reduce habitat quality for native species?	No	https://alienplantsbelgium.myspecies.info/content/pterocarya-fraxinifolia	Medium
25	4.12	Is the taxon likely to maintain a viable population even when present in low densities (or persist in adverse conditions by way of a dormant form)?	Yes	https://alienplantsbelgium.myspecies.info/content/pterocarya-fraxinifolia;	High
5. Resource exploitation					
26	5.01	Is the taxon likely to put threatened or protected native species under competitive pressure in the risk assessment area?	Yes	Professional judgement based on https://alienplantsbelgium.myspecies.info/content/pterocarya-fraxinifolia (riparian habitats).	Medium
27	5.02	Is the taxon likely to sequester resources to the detriment of native species in the risk assessment area?	Yes	Professional judgement based on https://alienplantsbelgium.myspecies.info/content/pterocarya-fraxinifolia (riparian habitats).	Medium
6. Reproduction					
28	6.01	Is the taxon likely to exhibit changes in reproductive strategy in response to environmental conditions?	Yes	Professional judgement based on https://alienplantsbelgium.myspecies.info/content/pterocarya-fraxinifolia (riparian habitats).	Medium
29	6.02	Is the taxon likely to produce viable propagules in the risk assessment area?	Yes	https://alienplantsbelgium.myspecies.info/content/pterocarya-fraxinifolia	High
30	6.03	Is the taxon likely to hybridise with native species under natural conditions?	Yes	There is not much data, but e.g. https://alienplantsbelgium.myspecies.info/search/site/pterocarya	Medium
31	6.04	Is the taxon likely to be hermaphroditic or to exhibit asexual reproduction?	Yes	eg. https://alienplantsbelgium.myspecies.info/content/pterocarya-fraxinifolia	Very high
32	6.05	Is the taxon dependent on the presence of another species (or specific habitat features) to complete its life cycle?	No	Idžojtić M. (2013): DENDROLOGIJA – cvijet, češer, plod, sjeme. Denona d.d. (In Croatian).; Yilmaz (2014): Journal of Science and Technology - C – Life Sciences and Biotechnology 3 (2): 39-44.	Very high
33	6.06	Is the taxon likely to produce a large number of propagules?	Yes	Idžojtić M. (2013): DENDROLOGIJA – cvijet, češer, plod, sjeme. Denona d.d. (In Croatian).; https://en.wikipedia.org/wiki/Pterocarya_fraxinifolia; https://www.treesandshrubsonline.org/articles/pterocarya/	Very high
34	6.07	How many time units (days, months, years) does the taxon require to reach the age at first reproduction?	>10	Idžojtić M. (2013): DENDROLOGIJA – cvijet, češer, plod, sjeme. Denona d.d. (In Croatian).	Very high
7. Dispersal mechanisms					
35	7.01	How many potential vectors or pathways could the taxon use to disperse within the risk assessment area (with suitable habitats nearby)?	>1	Idžojtić M. (2013): DENDROLOGIJA – cvijet, češer, plod, sjeme. Denona d.d. (In Croatian).; https://en.wikipedia.org/wiki/Pterocarya_fraxinifolia; https://www.treesandshrubsonline.org/articles/pterocarya/; https://alienplantsbelgium.myspecies.info/content/pterocarya-fraxinifolia	Very high
36	7.02	Will any of these vectors or pathways bring the taxon in close proximity to one or more protected areas?	Yes	https://alienplantsbelgium.myspecies.info/content/pterocarya-fraxinifolia (riparian habitats)	Very high
37	7.03	Does the taxon have a specialised means of attachment such that it enhances the likelihood of dispersal?	Yes	Idžojtić M. (2013): DENDROLOGIJA – cvijet, češer, plod, sjeme. Denona d.d. (In Croatian).; https://en.wikipedia.org/wiki/Pterocarya_fraxinifolia; https://www.treesandshrubsonline.org/articles/pterocarya/;	Very high
38	7.04	Is natural dispersal of the taxon likely to occur as seeds or spores in the risk	Yes	https://alienplantsbelgium.myspecies.info/content/pterocarya-fraxinifolia	Very high
39	7.05	Is natural dispersal of the taxon likely to occur as fragments or seedlings in the risk assessment area?	Yes	https://alienplantsbelgium.myspecies.info/content/pterocarya-fraxinifolia	Very high
40	7.06	Are any life stages of the taxon likely to migrate into the risk assessment area for reproduction?	Yes	https://alienplantsbelgium.myspecies.info/content/pterocarya-fraxinifolia	High
41	7.07	Are propagules of the taxon likely to be dispersed in the risk assessment area by other species?	No	https://alienplantsbelgium.myspecies.info/content/pterocarya-fraxinifolia	High
42	7.08	Is dispersal of the taxon along any of the vectors or pathways mentioned in the previous seven Questions (35–41: i.e. either unintentional or intentional) likely to be	No	No exact references found (professional judgement)	Medium
43	7.09	Is dispersal of the taxon density dependent?	Yes	https://alienplantsbelgium.myspecies.info/content/pterocarya-fraxinifolia;	High
8. Tolerance attributes					
44	8.01	Is the taxon able to withstand being in water for extended periods at some stage of its life cycle?	Yes	https://alienplantsbelgium.myspecies.info/content/pterocarya-fraxinifolia; Yilmaz (2014): Journal of Science and Technology - C – Life Sciences and Biotechnology 3 (2): 39-44. etc.	High
45	8.02	Is the taxon tolerant of a wide range of soil or air quality conditions?	No	Professional judgement based on https://alienplantsbelgium.myspecies.info/content/pterocarya-fraxinifolia and Yilmaz (2014): Journal of Science and Technology - C – Life Sciences and Biotechnology 3 (2): 39-44.	Medium
46	8.03	Can the taxon be controlled or eradicated in the wild with chemical, biological or other agents and means?	Yes	Voglmayr, H., Jaklitsch, W.M., Mohammadi, H. et al. The genus Juglanconis (Diaporthales) on Pterocarya. Mycol Progress 18, 425–437 (2019). https://doi.org/10.1007/s11557-018-01464-0 (pathogen)	Very high

47	8.04	Is the taxon likely to benefit from environmental or human disturbance?	No	Song, YG., Walas, Ł., Pietras, M. et al. Past, present and future suitable areas for the relict tree Pterocarya fraxinifolia (Juglandaceae): Integrating fossil records, niche modeling, and phylogeography for conservation. Eur J Forest Res 140, 1323–1339 (2021). https://doi.org/10.1007/s10342-021-01397-6 (because it is dependent on riparian habitats)	Medium
48	8.05	Is the taxon able to tolerate soil acidity, salinity or other parameter levels that are higher or lower than those found in its usual environment?	No	Song, YG., Walas, Ł., Pietras, M. et al. Past, present and future suitable areas for the relict tree Pterocarya fraxinifolia (Juglandaceae): Integrating fossil records, niche modeling, and phylogeography for conservation. Eur J Forest Res 140, 1323–1339 (2021). https://doi.org/10.1007/s10342-021-01397-6	Medium
49	8.06	Are there effective natural enemies of the taxon present in the risk assessment area?	Yes	Voglmayr, H., Jaklitsch, W.M., Mohammadi, H. et al. The genus Juglanconis (Diaporthales) on Pterocarya. Mycol Progress 18, 425–437 (2019). https://doi.org/10.1007/s11557-018-01464-0	High
**C. Climate change**					
9. Climate change					
50	9.01	Under the predicted future climatic conditions, are the risks of entry into the risk assessment area posed by the taxon likely to increase, decrease or not change?	Increase	Professional judgement based on Song, YG., Walas, Ł., Pietras, M. et al. Past, present and future suitable areas for the relict tree Pterocarya fraxinifolia (Juglandaceae): Integrating fossil records, niche modeling, and phylogeography for conservation. Eur J Forest Res 140, 1323–1339 (2021). https://doi.org/10.1007/s10342-021-01397-6 and https://alienplantsbelgium.myspecies.info/content/pterocarya-	High
51	9.02	Under the predicted future climatic conditions, are the risks of establishment within the risk assessment area posed by the taxon likely to increase, decrease or not change?	Increase	Professional judgement based on Song, YG., Walas, Ł., Pietras, M. et al. Past, present and future suitable areas for the relict tree Pterocarya fraxinifolia (Juglandaceae): Integrating fossil records, niche modeling, and phylogeography for conservation. Eur J Forest Res 140, 1323–1339 (2021). https://doi.org/10.1007/s10342-021-01397-6 and https://alienplantsbelgium.myspecies.info/content/pterocarya-	Medium
52	9.03	Under the predicted future climatic conditions, are the risks of dispersal within the risk assessment area posed by the taxon likely to increase, decrease or not change?	Increase	Professional judgement based on Song, YG., Walas, Ł., Pietras, M. et al. Past, present and future suitable areas for the relict tree Pterocarya fraxinifolia (Juglandaceae): Integrating fossil records, niche modeling, and phylogeography for conservation. Eur J Forest Res 140, 1323–1339 (2021). https://doi.org/10.1007/s10342-021-01397-6 and https://alienplantsbelgium.myspecies.info/content/pterocarya-	High
53	9.04	Under the predicted future climatic conditions, what is the likely magnitude of future potential impacts by the taxon on biodiversity or ecosystem integrity and status?	No change	Professional judgement based on Song, YG., Walas, Ł., Pietras, M. et al. Past, present and future suitable areas for the relict tree Pterocarya fraxinifolia (Juglandaceae): Integrating fossil records, niche modeling, and phylogeography for conservation. Eur J Forest Res 140, 1323–1339 (2021). https://doi.org/10.1007/s10342-021-01397-6 and https://alienplantsbelgium.myspecies.info/content/pterocarya-	Medium
54	9.05	Under the predicted future climatic conditions, what is the likely magnitude of future potential impacts by the taxon on ecosystem structure or function?	No change	Professional judgement based on Song, YG., Walas, Ł., Pietras, M. et al. Past, present and future suitable areas for the relict tree Pterocarya fraxinifolia (Juglandaceae): Integrating fossil records, niche modeling, and phylogeography for conservation. Eur J Forest Res 140, 1323–1339 (2021). https://doi.org/10.1007/s10342-021-01397-6 and https://alienplantsbelgium.myspecies.info/content/pterocarya-	Medium
55	9.06	Under the predicted future climatic conditions, what is the likely magnitude of future potential impacts by the taxon on ecosystem services and related socio-economic factors?	No change	Professional judgement based on Song, YG., Walas, Ł., Pietras, M. et al. Past, present and future suitable areas for the relict tree Pterocarya fraxinifolia (Juglandaceae): Integrating fossil records, niche modeling, and phylogeography for conservation. Eur J Forest Res 140, 1323–1339 (2021). https://doi.org/10.1007/s10342-021-01397-6 and https://alienplantsbelgium.myspecies.info/content/pterocarya-	Medium

**Statistics**	
**Scores**	
**BRA**	**27.5**
**BRA Outcome**	**Medium**
**BRA+CCA**	**33.5**
**BRA+CCA Outcome**	**Medium**
**Score partition**	
**A. Biogeography/Historical**	**7.5**
1. Domestication/Cultivation	*4.0*
2. Climate, distribution and introduction risk	*2.0*
3. Invasive elsewhere	*1.5*
**B. Biology/Ecology**	**20.0**
4. Undesirable (or persistence) traits	*6.0*
5. Resource exploitation	*7.0*
6. Reproduction	*5.0*
7. Dispersal mechanisms	*6.0*
8. Tolerance attributes	*-4.0*
**C. Climate change**	**6.0**
9. Climate change	*6.0*
**Answered Questions**	
Total	***55***
**A. Biogeography/Historical**	**13**
1. Domestication/Cultivation	*3*
2. Climate, distribution and introduction risk	*5*
3. Invasive elsewhere	*5*
**B. Biology/Ecology**	**36**

4. Undesirable (or persistence) traits	*12*
5. Resource exploitation	*2*
6. Reproduction	*7*
7. Dispersal mechanisms	*9*
8. Tolerance attributes	*6*
**C. Climate change**	**6**
9. Climate change	*6*
**Sectors affected**	
**Commercial**	**6**
**Environmental**	**7**
**Species or population nuisance traits**	**25**

**Thresholds**	
**BRA**	**41**
**BRA+CCA**	**41**
**Confidence**	
**BRA+CCA**	**0.73**
**BRA**	**0.75**
**CCA**	**0.58**

**Date and Time**	
	**14/08/2024 11:22:23**

TPS-ISK v2

Taxon and Assessor details	
Category	Angiosperms
Taxon name	*Rhus thyphina*
Common name	Staghorn Sumac
Assessor	Sara Essert
**Risk screening context**	
Reason and socio-economic benefits	
Risk assessment area	Continental Europe
Taxonomy	
Native range	
Introduced range	
URL	

			Response	Justification (references or other information)	Confidence
**A. Biogeography/Historical**					
1. Domestication/Cultivation					
1	1.01	Has the taxon been the subject of domestication?	Yes	"R. typhina has been cultivated in Europe for centuries as an ornamental...", "Fruit is produced on plants 3 to 4 years old..."- https://www.cabidigitallibrary.org/doi/10.1079/cabicompendium.47400#toDistributionMaps;	Very high
2	1.02	Is the taxon harvested in the wild and likely to be sold or used in its live form?	Yes	Uses List: Horticulture, oil production, source of medicine/pharmaceutical - https://www.cabidigitallibrary.org/doi/full/10.1079/cabicompendium.47400 For the area of Europe, I only found information that species is planted and used for decorative purposes.	High
3	1.03	Does the taxon have invasive races, varieties, sub-taxa or congeners?	No	I did not find such data in the GBFI database https://www.gbif.org/species/3190534 or anywhere else.	High
2. Climate, distribution and introduction risk					
4	2.01	How similar are the climatic conditions between the risk assessment area and the taxon's native range?	High	When comparing the climate where the species has original populations (https://en.wikipedia.org/wiki/Rhus_typhina) with the climate of the researched area (Peel et al (2007) Updated world map of the K¨oppen-Geiger climate classification, Hydrol. Earth Syst. Sci., 11, 1633–1644) the coincidence is confirmed. In the research area (continental Europe) there are large areas that have the same climate as the area where the species grows natively (Canada and some parts of USA).	Very high
5	2.02	What is the quality of the climate matching data?	High	A scientific paper (Peel et al (2007) Updated world map of the K¨oppen-Geiger climate classification, Hydrol. Earth Syst. Sci., 11, 1633–1644) was used in the assessment, so I estimate that it is of high quality.	High
6	2.03	Is the taxon known to be present outside of cultivation in the risk assessment area?	Yes	According to GBFI database the species is recorded as introduced and with evidence of impact for Switzerland, Czechia, Serbia and Liechtenstein - https://www.gbif.org/species/3190538 It is listed as invasivealien species in Luxembourgh - https://neobiota.lu/rhus-typhina/ and in Belgium -	Very high
7	2.04	How many potential vectors could the taxon use to enter the risk assessment area?	>1	"Natural dispersal of R. typhina is usually aided by birds or small animals such as ring-necked pheasants, rabbits, and quail, which may eat its seeds in winter. "The main dispersal manner, especially long distance, is the deliberate introduction as an ornamental or when used for soil protection and regeneration. " - https://www.cabidigitallibrary.org/doi/10.1079/cabicompendium.47400#sec-14, "Plant may be easily propagated via root fragments in soil movement." - https://ias.biodiversity.be/species/show/113	Very high
8	2.05	Is the taxon currently found in close proximity to, and likely to enter, the risk assessment area in the near future (e.g. unintentional or intentional introductions)?	Not applicable	The species is already present and naturalised in RA.	Very high
3. Invasive elsewhere					
9	3.01	Has the taxon become naturalised outside its native range?	Yes	It is listed as naturalised and invasive species in Belgium - https://ias.biodiversity.be/species/show/113, Switzerland - chrome-extension://efaidnbmnnnibpcajpcglclefindmkaj/https://www.infoflora.ch/en/assets/content/documents/neophyten/uw-2220-e-	Very high
10	3.02	In the taxon's introduced range, are there any known adverse impacts to wild or commercial species?	Yes	- "The plant has the potential to inhibit vegetation succession and tree regeneration. " - data for Luxenbourgh https://neobiota.lu/rhus-typhina/ - "With the ability to produce a dense monoculture, R. typhina can eliminate or reduce the abundance of many other species that cannot persist in the shade it creates." & "Impact outcomes: Damaged ecosystem services, Ecosystem change/ habitat alteration, Modification of hydrology, Modification of successional patterns, Monoculture formation, Negatively impacts agriculture, Negatively impacts human health, Reduced native biodiversity, Soil accretion, Threat to/ loss of native species, Damages animal/plant products" - https://www.cabidigitallibrary.org/doi/10.1079/cabicompendium.47400#toDistributionMaps, - "Rhus typhina represent invasive species that pose a low risk to autochthonous biodiversity...." - data for Serbia, chrome-extension://efaidnbmnnnibpcajpcglclefindmkaj/https://botanicaserbica.bio.bg.ac.rs/arhiva/pdf/2014_38_1_605_full.pdf - However, I have not found any scientific articles in which it is decidedly stated in which areas of RA and which native species' growth is	High

11	3.03	In the taxon's introduced range, are there any known adverse impacts to agriculture or forestry?	No	I haven't found such data.	Medium
12	3.04	In the taxon's introduced range, are there any known adverse impacts to ecosystem	No	I haven't found such data.	Medium
13	3.05	In the taxon's introduced range, are there any known adverse socio-economic impacts?	No	I haven't found such data.	Medium
**B. Biology/Ecology**					
4. Undesirable (or persistence) traits					
14	4.01	Is the taxon likely to be poisonous or pose other risks to human health?	Yes	"Contact with sap causes dermatitis in humans." - https://ias.biodiversity.be/species/show/113 " "...staghorn sumac has a milky sap that can produce dermatitis and its pollen causes severe allergies" - Cocirela & Oancea (2023) RHUS TYPHINA – FROM ENVIRONMENTAL THREAT TO INDUSTRY DEVELOPMENT OPPORTUNITY ,Current Trends in Natural Sciences, Vol. 12, Issue 23, pp. 305-320	Very high
15	4.02	Is the taxon likely to suppress the growth of one or more native species?	Yes	Given that the species is blacklisted in Switzerland and is on the list of invasive species in Belgium, the Czech Republic, Serbia... I think there is a possibility that it will suppress some native species. - "Impact on species, Competition: high" - for Belgium, https://ias.biodiversity.be/species/show/113, - "With the ability to produce a dense monoculture, R. typhina can eliminate or reduce the abundance of many other species that cannot persist in the shade it creates. " - https://www.cabidigitallibrary.org/doi/full/10.1079/cabicompendium.47400#sec-17	High
16	4.03	Are there any threatened or protected native species that the taxon would parasitise in the risk assessment area?	No	The species is not a parasite.	Very high
17	4.04	Is the taxon adaptable in terms of climatic and other environmental conditions, thus enhancing its potential persistence if it has invaded or is likely to invade the risk	Yes	The species is widely distributed outside its original range and is successfully cultivated in various climates, so I would say that it is adaptable.	Medium
18	4.05	Is the taxon likely to disrupt terrestrial ecosystem function if it has invaded or is likely to invade the risk assessment area?	Yes	I would say there is a possibility, but I don't think it's a big one.	Low
19	4.06	Is the taxon likely to exert adverse impacts on ecosystem services in the risk assessment area?	No	I found no evidence of that and according to the current distribution and behavior of the species in RA, I estimate that the species should not affect ecosystem services.	Medium
20	4.07	Is the taxon likely to host or function as a vector for recognised pests and pathogens that are present in the risk assessment area?	Yes	"The fungus, Botryosphaeria ribis may cause damage to R. typhina (Juhasova et al., 2005)." "Lygocoris communis have been found on R. typhina (Boivin and Stewart, 1982)." - https://www.cabidigitallibrary.org/doi/full/10.1079/cabicompendium.47400	High
21	4.08	Is the taxon likely to host or function as a vector for recognised pests and pathogens that are absent in the risk assessment area?	Yes	I believe there is a possibility, but I have not found information about any specialized pests that live exclusively in North America and feed on Rhus typhina.	Low
22	4.09	Is the taxon likely to be released from cultivation?	Not applicable	Taxon is already naturalised in some parts of RA (Switzerland...).	Very high
23	4.10	Is the taxon versatile in habitat use?	Yes	I would call this a wide range of habitats: "This pioneer plant occurs mainly in ruderal habitats and waster areas, but also along forest edges, in clearings, and shrublands. It thrives on relatively dry and poor soils, in well-lit conditions. It is resistant to abiotic stresses, incl. pollution." -	High
24	4.11	Is it likely that the taxon's mode of existence or behaviours will reduce habitat quality for native species?	No	"Rhus has no toxic effects reported." - https://www.gardenersworld.com/plants/rhus-typhina/	Very high
25	4.12	Is the taxon likely to maintain a viable population even when present in low densities (or persist in adverse conditions by way of a dormant form)?	Yes	In one locality in Serbia, populations with one and 25 individuals, which were naturalized, were recorded - Glišić et al (2005) GIS analysis of spatial distribution of invasive tree species in the protected natural area of Mt. Avala (Serbia), Botanica Serbica, 38 (1): (2014) 131-138	Medium
5. Resource exploitation					
26	5.01	Is the taxon likely to put threatened or protected native species under competitive pressure in the risk assessment area?	Yes	I have not found data that it endangers protected species, but since the species can do monocultures and is in competition with other plants in the habitat, it is not excluded that endangered species are under pressure.	Medium
27	5.02	Is the taxon likely to sequester resources to the detriment of native species in the risk assessment area?	Yes	The taxon will primarily be in competition for light in the habitat. "They strongly reduce light intensity and outcompete ground-layer perennial species. " - https://ias.biodiversity.be/species/show/113	High
6. Reproduction					
28	6.01	Is the taxon likely to exhibit changes in reproductive strategy in response to environmental conditions?	Yes	"The presence of fire also encourages increased germination..." - https://www.cabidigitallibrary.org/doi/full/10.1079/cabicompendium.47400#sec-14 I found that information only in this article and I found no confirmation on that in other literature sources, so I put Medium confidence level for this answer.	Medium
29	6.02	Is the taxon likely to produce viable propagules in the risk assessment area?	Yes	The taxon is naturalized and is listed on few alien invasive speicies in continetntal Europe (Switzerland, Luxembourg, Belgium...) - chrome-extension://efaidnbmnnnibpcajpcglclefindmkaj/https://www.infoflora.ch/en/assets/content/documents/neophyten/uw-2220-e-iga.pdf, https://ias.biodiversity.be/species/show/113,	Very high
30	6.03	Is the taxon likely to hybridise with native species under natural conditions?	No	I did not find any data with evidence of hibridisation with native species.	Medium

31	6.04	Is the taxon likely to be hermaphroditic or to exhibit asexual reproduction?	Yes	"Clonal spreading rate is rather high...", "Plant may be easily propagated via root fragments in soil movement...." - https://ias.biodiversity.be/species/show/113, "R. typhina can reproduce sexually by producing seeds or asexually by rhizomatous growth. ..."- https://www.cabidigitallibrary.org/doi/full/10.1079/cabicompendium.47400#sec-14	Very high
32	6.05	Is the taxon dependent on the presence of another species (or specific habitat features) to complete its life cycle?	No	I found no evidence about depending on any other species.	High
33	6.06	Is the taxon likely to produce a large number of propagules?	Yes	"Each cluster of drupes may contain 100 to 700 seeds. There are about 60,000 seeds per pound..." - https://www.cabidigitallibrary.org/doi/full/10.1079/cabicompendium.47400#sec-14 I am not sure if this number is high, but for a tree it is a quite large number of seeds.	Low
34	6.07	How many time units (days, months, years) does the taxon require to reach the age at first reproduction?	4	"Fruit is produced on plants 3 to 4 years old..." - https://www.cabidigitallibrary.org/doi/full/10.1079/cabicompendium.47400#sec-14	Very high
7. Dispersal mechanisms					
35	7.01	How many potential vectors or pathways could the taxon use to disperse within the risk assessment area (with suitable habitats nearby)?	>1	1) deliberate introduction as an ornamental or when used for soil protection and regeneration - ("R. typhina has been cultivated in Europe for centuries as an ornamental, prized for its vivid autumn foliage and distinctive fruit." - https://www.cabidigitallibrary.org/doi/full/10.1079/cabicompendium.47400#sec-14), 2) via animals ("At a local level in its native range, the seeds of R. typhina may disperse via birds .", "Natural dispersal of R. typhina is usually aided by birds or small animals such as ring-necked pheasants, rabbits, and quail, which may eat its seeds in winter. Passage through the digestive system of such organisms can encourage the germination of R. typhina seeds.") 3) unintentional transfer of the species when transferring the soil in which the species grew ("Plant may be easily propagated via root fragments in soil movement."-	Very high
36	7.02	Will any of these vectors or pathways bring the taxon in close proximity to one or more protected areas?	Yes	Given that the species is already naturalized in some countries, there is certainly a chance that it will come to protected areas. It has already happened in Serbia - Glišić et al (2005) GIS analysis of spatial distribution of invasive tree species in the protected natural area of Mt. Avala (Serbia), Botanica Serbica, 38 (1):	Very high
37	7.03	Does the taxon have a specialised means of attachment such that it enhances the likelihood of dispersal?	No	The plant has no hooks.	Very high
38	7.04	Is natural dispersal of the taxon likely to occur as seeds or spores in the risk assessment area?	No	It seems that in Europe the species is mostly distributed vegetatively: -"The main reproduction manner of R. typhina is vegetative rather than sexual, for its seeds have a low germination rate with a water-impermeable endocarp (Norton, 1985; Baskin et al., 2000)."- https://www.cabidigitallibrary.org/doi/full/10.1079/cabicompendium.47400#sec-14. -"Clonal spreading rate is rather high but reproduction by seeds is rarely reported from the field in Belgium (seeds exhibit dormancy, probably as a result of hard, impermeable seedcoats)." - https://ias.biodiversity.be/species/show/113 - However, there is data that the species also spreads via to birds and other animals ("Natural dispersal of R. typhina is usually aided by birds or small animals such as ring-necked pheasants, rabbits, and quail, which may eat its seeds in winter. " - https://www.cabidigitallibrary.org/doi/full/10.1079/cabicompendium.47400#sec-14), but I have not found papers for RA that prove	Medium
39	7.05	Is natural dispersal of the taxon likely to occur as fragments or seedlings in the risk assessment area?	Yes	"Plant may be easily propagated via root fragments in soil movement." - https://ias.biodiversity.be/species/show/113	Very high
40	7.06	Are any life stages of the taxon likely to migrate into the risk assessment area for reproduction?	Yes	" Natural dispersal of R. typhina is usually aided by birds or small animals such as ring-necked pheasants, rabbits, and quail, which may eat its seeds in winter. " - https://www.cabidigitallibrary.org/doi/full/10.1079/cabicompendium.47400#sec-14	Medium
41	7.07	Are propagules of the taxon likely to be dispersed in the risk assessment area by other species?	Yes	"Natural dispersal of R. typhina is usually aided by birds or small animals such as ring-necked pheasants, rabbits, and quail, which may eat its seeds in winter." - https://www.cabidigitallibrary.org/doi/full/10.1079/cabicompendium.47400#sec-14	Medium
42	7.08	Is dispersal of the taxon along any of the vectors or pathways mentioned in the previous seven Questions (35–41: i.e. either unintentional or intentional) likely to be	Yes	The species is still often used in horticulture, which is certainly a fast way of spreading.	Very high
43	7.09	Is dispersal of the taxon density dependent?	Yes	I wouldn't say that population density is crucial, but surely a larger number of seeds/trees increases the possibility of the species spreading.	Low
8. Tolerance attributes					
44	8.01	Is the taxon able to withstand being in water for extended periods at some stage of its life cycle?	No	I found no evidence that the species can be for a long time in water in any phase of life.	Medium

45	8.02	Is the taxon tolerant of a wide range of soil or air quality conditions?	Yes	"It also thrives in polluted city air, and grows in such inhospitable sites as cracks in the pavement. " - https://www.cabidigitallibrary.org/doi/full/10.1079/cabicompendium.47400#sec-14, "It thrives on relatively dry and poor soils, in well-lit conditions. It is resistant to abiotic stresses, incl. pollution." - https://ias.biodiversity.be/species/show/113	Very high
46	8.03	Can the taxon be controlled or eradicated in the wild with chemical, biological or other agents and means?	Yes	"Mechanical Control Small populations can be kept under control by conducting prescribed burns every 3 to 4 years. Spring burns are conducted to stimulate herbaceous vegetation. Double-cutting at ground level (once in July and once in August) is another control strategy. Cutting may need to be repeated for several consecutive years to effectively control dense populations. Sumac will re-sprout after each cutting, but dense vegetation may prevent sumac from receiving enough sunlight, causing the leaves to turn yellow and eventually die. Chemical Control During July and August a 20% concentration of glyphosate can be applied to freshly cut stumps. Oil-based triclopyr can be applied as directed on the label to the entire circumference of each stem of the clone; no cutting is carried out. Foliar application of water-based triclopyr as directed on the label or 1-2% solution of glyphosate can be applied in areas with little or no native vegetation. Another control measure is 41% amiton at 0.3 ml/m2 during July and August (Wu et al., 2007)." - https://www.cabidigitallibrary.org/doi/full/10.1079/cabicompendiu	Very high
47	8.04	Is the taxon likely to benefit from environmental or human disturbance?	Yes	"The presence of fire also encourages increased germination..." - https://www.cabidigitallibrary.org/doi/full/10.1079/cabicompendium.47400#sec-14 I found that information only in this article and I found no confirmation on that in other literature sources. In addition, the species most often occurs in ruderal and degraded places, where the climazonal vegetation has been destroyed, because there are no strong competitors and there is a sufficient amount of necessary light for survival. - "Thus, observing the highest abundance of R. typhina on Mt. Avala in an abandoned quarry and its surroundings, near the ascending part of the ring road, was to be expected. A large number of R. typhina trees were present in this location, along with a large number of young sprouts. Furthermore, the staghorn sumac was observed in an abandoned quarry near the onumental complex at the top of Mt. Avala, also in the vicinity of the ring road. It is present at several other locations near the roads, as well as in the stand of planted conifers on the southern slopes of Mt. Avala, where it grows together with the black locust. It was not observed in natural forest communities." - Glišić et al (2005) GIS analysis of spatial distribution of invasive tree species in the protected natural area	High
48	8.05	Is the taxon able to tolerate soil acidity, salinity or other parameter levels that are higher or lower than those found in its usual environment?	No	"Soil Tolerances Soil texture > light Soil texture > medium Soil reaction > acid Soil reaction > neutral Soil drainage > free Special soil tolerances > shallow Special soil tolerances > infertile" - https://www.cabidigitallibrary.org/doi/full/10.1079/cabicompendium.47400#sec-25	High
49	8.06	Are there effective natural enemies of the taxon present in the risk assessment area?	Yes	"Lygocoris communis have been found on R. typhina (Boivin and Stewart, 1982). The fungus, Botryosphaeria ribis may cause damage to R. typhina (Juhasova et al., 2005)." - https://www.cabidigitallibrary.org/doi/full/10.1079/cabicompendium.47400#sec-26 The species is attacked by some pests, but I found no research if those pests are effective natural enemies that could maintain the population in such a way as to prevent excessive expansion.	Medium
**C. Climate change**					
9. Climate change					
50	9.01	Under the predicted future climatic conditions, are the risks of entry into the risk assessment area posed by the taxon likely to increase, decrease or not change?	Not applicable	The species ia already in the RA.	Very high
51	9.02	Under the predicted future climatic conditions, are the risks of establishment within the risk assessment area posed by the taxon likely to increase, decrease or not	No change	Climate change could have a positive effect on Rhus in the way that climazonal communities start to collapse and are taken over by various neophytes and pioneers like this species. However, I haven't found any research data about that, so this is just my	Low
52	9.03	Under the predicted future climatic conditions, are the risks of dispersal within the risk assessment area posed by the taxon likely to increase, decrease or not change?	Increase	Climate change could have a positive effect on Rhus in the way that climazonal communities start to collapse and are taken over by various neophytes and pioneers, like this species. However, I haven't found any research data about that, so this is just my	Low
53	9.04	Under the predicted future climatic conditions, what is the likely magnitude of future potential impacts by the taxon on biodiversity or ecosystem integrity and status?	Higher	Given that climate change will weaken the current plant communities, Rhus will have more opportunities to occupy space, and since it often grows in monocultures, it may impact the reduction of biological diversity. The level of confidence is Low, as I haven't found research on this topic.	Low
54	9.05	Under the predicted future climatic conditions, what is the likely magnitude of future potential impacts by the taxon on ecosystem structure or function?	No change	I don't think the species will disrupt ecosystems so much that it will greatly affect ecosystem function.	Low
55	9.06	Under the predicted future climatic conditions, what is the likely magnitude of future potential impacts by the taxon on ecosystem services and related socio-	No change	I don't think the species will disrupt the ecosystems so much that it will affect socio-economic factors (a lot).	Low

Statistics	
**Scores**	
**BRA**	**33.0**
**BRA Outcome**	**Medium**
**BRA+CCA**	**37.0**
**BRA+CCA Outcome**	**Medium**
**Score partition**	
**A. Biogeography/Historical**	**10.0**
1. Domestication/Cultivation	*2.0*
2. Climate, distribution and introduction risk	*2.0*
3. Invasive elsewhere	*6.0*
**B. Biology/Ecology**	**23.0**
4. Undesirable (or persistence) traits	*8.0*
5. Resource exploitation	*7.0*
6. Reproduction	*3.0*
7. Dispersal mechanisms	*5.0*
8. Tolerance attributes	*0.0*
**C. Climate change**	**4.0**
9. Climate change	*4.0*
**Answered Questions**	
Total	***55***
**A. Biogeography/Historical**	**13**
1. Domestication/Cultivation	*3*
2. Climate, distribution and introduction risk	*5*
3. Invasive elsewhere	*5*
**B. Biology/Ecology**	**36**
4. Undesirable (or persistence) traits	*12*
5. Resource exploitation	*2*
6. Reproduction	*7*
7. Dispersal mechanisms	*9*
8. Tolerance attributes	*6*
**C. Climate change**	**6**
9. Climate change	*6*
**Sectors affected**	
**Commercial**	**11**
**Environmental**	**9**
**Species or population nuisance traits**	**22**

Thresholds	
**BRA**	**41**
**BRA+CCA**	**41**
**Confidence**	
**BRA+CCA**	**0.70**
**BRA**	**0.74**
**CCA**	**0.38**

Date and Time
**09/08/2024 15:37:02**

TPS-ISK v2

**Taxon and Assessor details**	
Category	Angiosperms
Taxon name	*Robinia pseudoacacia*
Common name	Black Locust (False acacia)
Assessor	Sara Essert
**Risk screening context**	
Reason and socio-economic benefits	
Risk assessment area	Continental Europe
Taxonomy	
Native range	
Introduced range	
URL	

			**Response**	**Justification (references or other information)**	**Confidence**
**A. Biogeography/Historical**					
1. Domestication/Cultivation					
1	1.01	Has the taxon been the subject of domestication?	Yes	"Black locust was the first North American tree species to be introduced into Europe in 1601" (Muller 2004; Hemery and Simblet 2014) - Valeriu-Norocel Nicolescu et al (2020) Ecology, growth and management of black locust (Robinia pseudoacacia L.), a non-native species integrated into European forests. J. For. Res. 31(4):1081–1101, Muller S (2004) Robinia pseudoacacia L. Black locust. In: Muller S (eds) Invasive plants in France. Muséum national d'Histoire naturelle, Patrimoins aturels, Paris, vol 62, pp 92–93 (in French); Hemery G, Simblet S (2014) The New Sylva. A discourse of forest and orchard trees for the twenty-first century.	Very high
2	1.02	Is the taxon harvested in the wild and likely to be sold or used in its live form?	Yes	"This wide-spreading planting is because black locust is an important multipurpose species, producing wood, fodder, and a source of honey as well as bio-oil and biomass."- Valeriu-Norocel Nicolescu et al (2020) Ecology, growth and management of black locust (Robinia pseudoacacia L.), a non-native species integrated into European forests. J. For. Res. (2020) 31(4):1081–1101	Very high
3	1.03	Does the taxon have invasive races, varieties, sub-taxa or congeners?	No	I did not find articles about invasive varieties or taxa from the Robinia genus. But the species R. hispida and R. viscosa live in USA mostly on anthropogenic (man-made or disturbed habitats), meadows and fields (https://gobotany.nativeplanttrust.org/species/robinia/hispida/) and are naturalised in many USA states, so I would say they have	Medium
2. Climate, distribution and introduction risk					
4	2.01	How similar are the climatic conditions between the risk assessment area and the taxon's native range?	High	When comparing the climate where the species has original populations (https://hr.wikipedia.org/wiki/Datoteka:Robinia_pseudoacacia_distribution_map.png) with the climate of the researched area (Peel et al (2007) Updated world map of the K¨oppen-Geiger climate classification, Hydrol. Earth Syst. Sci., 11, 1633–1644) the coincidence is confirmed, which is surely one of the reasons for the naturalization of the species in many countries in Europe	Very high
5	2.02	What is the quality of the climate matching data?	High	A scientific paper (Peel et al (2007) Updated world map of the K¨oppen-Geiger climate classification, Hydrol. Earth Syst. Sci., 11, 1633–1644) was used in the assessment, so I estimate that it is of high quality	High
6	2.03	Is the taxon known to be present outside of cultivation in the risk assessment area?	Yes	"Currently black locust occurs in forty-two European countries and is naturalised in thirty-two (Pyšek et al. 2009)." - Valeriu-Norocel Nicolescu et al (2020) Ecology, growth and management of black locust (Robinia pseudoacacia L.), a non-native species integrated into European forests. J. For. Res. (2020) 31(4):1081–1101, Pyšek P, Lambdon PW, Arianoutsou M, Kühn I, Pino J, Winter M (2009) Alien vascular plants of Europe. In: Nentwig W (ed) Handbook of Alien Species in Europe, DAISIE. Invading nature: Springer series in invasion ecology, vol 3. Springer, Dordrecht, pp	Very high
7	2.04	How many potential vectors could the taxon use to enter the risk assessment area?	>1	The reasons for planting the trees are: 1) "Initially, black locust was used as a park and garden tree and the first important forest use, in the nineteenth century, was to stabilize mobile sand dunes (Hungary, Romania, Austria) and to create shelterbelts (Austria) 2) "In Europe, black locust is cultivated on a large scale for timber production." 3) nitrogen-fixing species for waste land and surface-mine reclamation, 4) for erosion prevention and control 5) for carbon sequestration 6) as windbreaks and shelterbelts, as an ornamental tree in parks, gardens, alleys, and as a street tree as it is tolerant of air pollution and salinity and thrives in the urban environment - Valeriu-Norocel Nicolescu et al (2020) Ecology, growth and management of black locust (Robinia pseudoacacia L.), a non-native species integrated into European forests. J. For. Res. 31(4):1081–1101 The plant can also "escapes" from parks	Very high
8	2.05	Is the taxon currently found in close proximity to, and likely to enter, the risk assessment area in the near future (e.g. unintentional or intentional introductions)?	Not applicable	"The species has become naturalized (i.e., forming freeliving, self-sustaining populations in the wild unsupported by and independent of humans; Pyšek et al. 2009) in all sub-Mediterranean and temperate regions..." - Valeriu-Norocel Nicolescu et al (2020) Ecology, growth and management of black locust (Robinia pseudoacacia L.), a non-native species integrated into European forests. J. For. Res. 31(4):1081–1101	Very high
3. Invasive elsewhere					

9	3.01	Has the taxon become naturalised outside its native range?	Yes	"Currently black locust occurs in forty-two European countries and is naturalised in thirty-two (Pyšek et al. 2009)." - Valeriu-Norocel Nicolescu et al (2020) Ecology, growth and management of black locust (Robinia pseudoacacia L.), a non-native species integrated into European forests. J. For. Res. 31(4):1081–1101 , Pyšek P, Lambdon PW, Arianoutsou M, Kühn I, Pino J, Winter M (2009) Alien vascular plants of Europe. In: Nentwig W (ed) Handbook of Alien Species in Europe, DAISIE. Invading nature: Springer series in invasion ecology, vol 3. Springer, Dordrecht, pp 43–61	Very high
10	3.02	In the taxon's introduced range, are there any known adverse impacts to wild or commercial species?	Yes	"As a N-fixing species, black locust creates irreversible changes in physico-chemical and biological soil properties. It influences the species composition of ground flora under its canopy by favouring ruderal and species-poor neutrophilous weed associations, eliminating oligotrophic and acidophilous ones typical of forests, thereby reducing plant diversity (Kleinbauer et al. 2010; Benesperi et al. 2012; Cseresnyés and Csontos 2012; Dimitrova 2012; Vuković et al. 2013). In invaded stands, as soil pH is significantly lower and soil NO3 − significantly higher, soil microcroarthropode communities suffer significant loss of abundance and richness while nematode taxon richness is significantly lower (Lazzaro et al. 2018). Black locust also produces different allelopathic compounds (e.g., robinetin, myricetin and quercetin) which may affect the ecological functioning of natural areas by inhibiting the growth of various weeds and crop species (Nasir et al. 2005; Boer 2013). However, as the allelopathic effects of black locust have not yet been tested under natural conditions, it seems that vegetation change towards ruderal and nitrophilous species is caused by the change in the availability of soil nutrients rather than by allelopathy (Vítková and Kolbek 2010). Once introduced in an area, black locusts expands readily into open sites where they form dense colonies and their shade reduces competition from other sun-loving species hindering their natural regeneration and thus a local, small-scale reduction of biodiversity can occur (Kutnar and Kobler 2013)." - Valeriu-Norocel Nicolescu et al (2020) Ecology, growth and management of black locust (Robinia pseudoacacia L.), a	Very high
11	3.03	In the taxon's introduced range, are there any known adverse impacts to agriculture or forestry?	No	Although the "growth of species often causes a decrease in the number of species in the habitat (Benesperi et al. 2012)", I would not say that it brings great economic damage in agriculture or forestry. "Under comparable environmental conditions (slope, elevation, canopy cover), the replacement of native forests by pure black-locust stands results in both plant richness loss and shift in species composition. Moreover, these impacts on species heterogeneity and species composition indicate that plant communities of native forests are more diverse with respect to black-locust stands, suggesting that in Northern Apennines black-locust spread also causes a homogenization of the plant forest biota. These results are in accordance with several previous studies demonstrating that biotic homogenization, with consequent biodiversity loss, is among the most detrimental effects of biological invasions (e.g., Sax et al. 2002; Davis 2003). - Benesperi et al. (2012) Forest plant diversity is threatened by Robinia pseudoacacia (black-locust) Invasion Biodivers Conserv	High
12	3.04	In the taxon's introduced range, are there any known adverse impacts to ecosystem services?	Yes	"In the study area black-locust is rapidly spreading over nutrient poor chestnut and oak forests" - Benesperi et al. (2012) This could mean that the chestut, which could be used as a food, is replaced with Robinia and that is why I answered "Yes".	Low
13	3.05	In the taxon's introduced range, are there any known adverse socio-economic impacts?	No	I did not come to such knowledge by searching the literature.	Medium
**B. Biology/Ecology**					
4. Undesirable (or persistence) traits					
14	4.01	Is the taxon likely to be poisonous or pose other risks to human health?	Yes	"Black locust trees contain the toxalbumins (toxic protein molecules produced by certain plants) robin and phasin in their leaves, bark, and seeds. These toxic compounds inhibit protein synthesis and can cause cell death ... Based on the reports of ingestions by humans in the literature, the most common symptoms after ingestion include nausea, weakness, flushing, lethargy, and vomiting. Rare reports of more severe symptoms like liver injury and seizures have been reported. There have been suggestions that symptoms appear to be related to the amount of material ingested and the extent of chewing—higher amounts and more chewing leads to potentially more serious symptoms. ... there have also been several reports of skin punctures and injury from the thorns located on the bark and branches. Most of these exposures have resulted in local symptoms such as pain, swelling, and rash. However, there have been a few reports of more severe outcomes following punctures from black locust thorns including neurologic (brain related) symptoms and local tissue death (necrosis)." -https://www.poison.org/articles/are-black-locust-trees-toxic-221 Although severe toxicity after exploratory exposures to black locust seeds and bark is uncommon, the handful of cases reported indicate that all black locust exposures	Very high

15	4.02	Is the taxon likely to suppress the growth of one or more native species?	Yes	"Black locust also produces different allelopathic compounds (e.g., robinetin, myricetin and quercetin) which may affect the ecological functioning of natural areas by inhibiting the growth of various weeds and crop species (Nasir et al. 2005; Boer 2013). However, as the allelopathic effects of black locust have not yet been tested under natural conditions, it seems that vegetation change towards ruderal and nitrophilous species is caused by the change in the availability of soil nutrients rather than by allelopathy (Vítková and Kolbek 2010). Once introduced in an area, black locusts expands readily into open sites where they form dense colonies and their shade reduces competition from other sun-loving species hindering their natural regeneration and thus a local, small-scale reduction of biodiversity can occur (Kutnar and Kobler 2013)." - Valeriu-Norocel Nicolescu et al (2020) Ecology, growth and management of black locust (Robinia pseudoacacia L.), a non-native species integrated into European forests. J. For. Res. 31(4):1081–1101; Boer E (2013) Risk assessment Robinia pseudoacacia L. Naturalis Biodiversity Center, Leiden, p 18; Nasir H, Iqbal Z, Hiradate S, Fujii Y (2005) Allelopathic potential of Robinia pseudo-acacia L. J Chem Ecol 9:2179–2192; Vítková M, Müllerová J, Sádlo J, Pyšek P (2017) Black locust (Robinia pseudoacacia) beloved and despised: a story of an invasive tree in Central Europe. For Ecol Manag 384:287–302 „Plant communities of native forests are more diverse with respect to pure black-locust stands, suggesting that black-locust invasion also causes a homogenization of the plant forest biota." - Benesperi et al. (2012) Forest plant diversity is	Very high
16	4.03	Are there any threatened or protected native species that the taxon would parasitise in the risk assessment area?	No	The species is not a parasite.	Very high
17	4.04	Is the taxon adaptable in terms of climatic and other environmental conditions, thus enhancing its potential persistence if it has invaded or is likely to invade the risk assessment area?	Yes	"As its potential to adapt to predicted climate changes, particularly drought, seems to be high, the importance of black locust, a species both "beloved and despised" (Vítková et al. 2017) in Europe, is expected to increase in the future." "In its native range, black locust grows in a humid climate (annual total precipitation 1020–1830 mm), with 150–210 frost-free days (Huntley 1990). In Europe, black locust behaves like a thermophilous species, i.e., it grows well where mean annual temperatures are over 8 °C (Roșu 1996; Rédei et al. 2017) and is very drought tolerant (Claessens et al. 2006; Grünewald et al. 2009; Mantovani et al. 2014; CRPF Nord Pas-de-Calais Picardie 2015). In this respect, black locust can adapt to prolonged drought by reducing water loss through both reduced transpiration and leaf size (Mantovani et al. 2014), as well as due to an extended rootsystem which access water in deeper soil layers (Grünewald et al. 2009). Consequently, the species can grow in regions with 500–550 mm annual precipitation (Roșu 1996; Mantovani et al. 2014), with reports of it tolerating less than 400 mm year−1 (Engel et al. 2014), high summer air temperatures of 30–35 °C, and relative air humidity in July of 20–50%, as found in Hungary (Rédei et al. 2002)." - Valeriu-Norocel Nicolescu et al (2020) Ecology, growth and management of black locust (Robinia	Very high
18	4.05	Is the taxon likely to disrupt terrestrial ecosystem function if it has invaded or is likely to invade the risk assessment area?	Yes	"Our study confirmed that the replacement of native forests by pure black-locust stands causes both plant richness loss and shifts in species composition. In black-locust stands plant communities are dominated by nitrophilous species and lack many of the oligothrophic and acidophilus species typical of native forests. Plant communities of native forests are more diverse with respect to pure black-locust stands, suggesting that black-locust invasion also causes a homogenization of the plant forest biota." - Benesperi et al. (2012) Forest plant diversity is threatened by Robinia pseudoacacia (black-locust) InvasionBiodivers Conserv	Very high
19	4.06	Is the taxon likely to exert adverse impacts on ecosystem services in the risk assessment area?	Yes	"In the study area black-locust is rapidly spreading over nutrient poor chestnut and oak forests" - Benesperi et al. (2012) Forest plant diversity is threatened by Robinia pseudoacacia (black-locust) InvasionBiodivers Conserv 21:3555–3568 (What is the difference between this question and the question number 12?)	Medium
20	4.07	Is the taxon likely to host or function as a vector for recognised pests and pathogens that are present in the risk assessment area?	Yes	"In Europe, black locust appears to have high pest and disease resistance; however, in recent decades, several insects, fungi and semi-parasites have been reported to affect mature trees, seeds and seedlings. For instance, the leaves have been affected by black locust leaf miner (Phyllonorycter robiniae Clemens), locust digitate leaf miner (Parectopa robiniella Clemens), black locust gall midge (Obolodiplosis robiniae Hald.), gall midge (Platygaster robiniae n.sp.), soft scale (Parthenolecanium corni Bouché), and black bean aphid (Aphis fabae Scopoli)..." - Valeriu-Norocel Nicolescu et al (2020) Ecology, growth and management of black locust (Robinia pseudoacacia L.), a non-native species integrated into European forests. J. For. Res. 31(4):1081–1101	Very high

21	4.08	Is the taxon likely to host or function as a vector for recognised pests and pathogens that are absent in the risk assessment area?	Yes	Although the pathogen is not yet present in the researched area, it is absolutely possible that it will appear and attack the species in the future "Fortunately, the most important pest in native range, the locust borer Megacyllene robiniae (Forst.), is not yet present in Europe. Tunnels produced by its larvae in the heartwood weaken tree limbs, making them susceptible to breakage by wind and also serve as primary infection sites for windborne spores of the fungus Phellinus rimosus (Berk.) Pilát which causes damaging heart rot disease. This combined attack makes growing black locust for timber production impractical where this pest is present (Huntley 1990)." - Valeriu-Norocel Nicolescu et al (2020) Ecology, growth and management of black locust (Robinia pseudoacacia L.), a non-native species integrated	Very high
22	4.09	Is the taxon likely to be released from cultivation?	Not applicable	The taxon is already realeased from captivity in RA. "Currently black locust occurs in forty-two European countries and is naturalised in thirty-two (Pyšek et al. 2009)." - Valeriu-Norocel Nicolescu et al (2020) Ecology, growth and management of black locust (Robinia pseudoacacia L.), a non-native species integrated into European forests. J. For. Res. 31(4):1081–1101	Very high
23	4.10	Is the taxon versatile in habitat use?	Yes	"In the United States, black locust is found naturally over a wide range of soils but grows best on moist, rich, loamy soils or those of limestone origin (Huntley 1990) ... In Europe, the species has been introduced over a wide range of soils and tolerates diverse pH from extremely acid (3.2) to alkaline (8.8) (Vítková et al. 2015) ... In Europe, black locust has been used across a wide range of altitudes, from sea level to 1640 m a.s.l. in the Southern Alps of Italy (Sitzia et al. 2016), but grows best in plains and hilly areas at altitudes below 600–700 m a.s.l. (Vuković et al. 2013; Šnjegota et al. 2017)." - Valeriu-Norocel Nicolescu et al (2020) Ecology, growth and management of black locust (Robinia pseudoacacia L.), a non-native species integrated into European	Very high
24	4.11	Is it likely that the taxon's mode of existence or behaviours will reduce habitat quality for native species?	Yes	https://www.poison.org/articles/are-black-locust-trees-toxic-221- "The earliest reports of possible poisoning associated with the black locust involved horses, which either became severely ill or died from swallowing parts of the tree." https://www.sciencedirect.com/topics/pharmacology-toxicology-and-pharmaceutical-science/robinia-pseudoacacia - "The black locust tree has been described as toxic to horses. The toxic principle is classified as a lectin, which is present in seeds, sap, roots, wood, leaves, and bark of the plant. Lectin has been used in cytologic research because of its ability to stimulate glycoprotein biosynthesis and cell proliferation in lymphocytes of various animal species. Horses may become intoxicated by ingesting the bark. Only small amounts of lectin are reported to precipitate	Very high
25	4.12	Is the taxon likely to maintain a viable population even when present in low densities (or persist in adverse conditions by way of a dormant form)?	Yes	The species grows without problems in larger or smaller populations. Proof of this is individual cultivation in horticulture, planting next to agricultural plots, etc. In addition, it can survive for over 10 years in the form of viable seeds. "They may persist in a dormant state in the soil for a long time, with a viability longer than 10 years (Haralamb 1967; Boer 2013) or, in some circumstances, up to 40 years (Toole and Brown 1946, cited by Cseresnyés and Csontos 2012), accumulating in a permanent seed bank (Stone 2009; Filipas 2013)." - Valeriu-Norocel Nicolescu et al (2020) Ecology, growth and management of black locust (Robinia pseudoacacia L.), a non-native species integrated into European	Very high
5. Resource exploitation					
26	5.01	Is the taxon likely to put threatened or protected native species under competitive pressure in the risk assessment area?	Yes	There is a possibility that the species exerts pressure on endangered species as well, although I have not found a paper in which particularly endangered species are mentioned.	Medium
27	5.02	Is the taxon likely to sequester resources to the detriment of native species in the risk assessment area?	Yes	This pioneer species will use/need a lot of light, so over time the heliophytic species in open habitats will be replaced by those that tolerate shade (which will be under growing trees of Robinia). I haven't found data that Robinia exploits significantly other resources to the detriment of native species "Once introduced in an area, black locusts expands readily into open sites where they form dense colonies and their shade reduces competition from other sun-loving species hindering their natural regeneration and thus a local, small-scale reduction of biodiversity can occur (Kutnar and Kobler 2013). - Valeriu-Norocel Nicolescu et al (2020) Ecology, growth and management of black locust (Robinia pseudoacacia L.), a non-native species integrated into European forests. J. For. Res. 31(4):1081–1101 I did not find or calculate	Medium
6. Reproduction					

28	6.01	Is the taxon likely to exhibit changes in reproductive strategy in response to environmental conditions?	Yes	Human influence on the habitat through mechanization favors the seed and vegetative propagation of the species. "However, natural regeneration from seed can be facilitated by seed wounding with heavy machinery (Tănăsescu 1961) or by natural thermal shock. The latter occurs particularly on sand dunes where temperatures of the surface layer reach 60–65 °C in summer (Spîrchez et al. 1962). ...natural regeneration is primarily by root suckers. These are produced abundantly even by trees over 70 years old (Vítková et al. 2017) from superficial roots, particularly following mechanical damage to the root system (Boer 2013; Vítková et al. 2016) or soil disturbance (Savill 2013)." - Valeriu-Norocel Nicolescu et al (2020) Ecology, growth and management of black locust (Robinia pseudoacacia L.), a non-native species integrated into European forests. J. For. Res. 31(4):1081–1101	Very high
29	6.02	Is the taxon likely to produce viable propagules in the risk assessment area?	Yes	"Black locust produces abundant seed crops with 120–150 kg of seeds ha−1 in an average seed year, beginning from around 6 years of age (Rédei et al. 2001; Rédei 2013a, b, c, d). Good seed crops occur every 1–2 years in both Europe (Haralamb 1967; Rédei et al. 2012; Boer 2013; Los et al. 2014; Meyer-Münzer et al. 2015) and the USA (Huntley 1990)." - Valeriu-Norocel Nicolescu et al (2020) Ecology, growth and management of black locust (Robinia pseudoacacia L.), a non-native species integrated into European forests. J. For. Res. 31(4):1081–1101	Very high
30	6.03	Is the taxon likely to hybridise with native species under natural conditions?	No	I did not find such information in the literature.	High
31	6.04	Is the taxon likely to be hermaphroditic or to exhibit asexual reproduction?	Yes	This species, like most other plants, has the possibility of vegetative reproduction. "Both in native range (McAlister 1971; Huntley 1990) and in Europe (Stoyanov 1926; Negulescu and Săvulescu 1957; Haralamb 1967; CRPF 2007; Branquart et al. 2007; Rédei et al. 2008; Vuković et al. 2013) as well as in Asia (Luna 1996), natural regeneration is primarily by root suckers. ... Black locust also regenerates on a much smaller scale by stool shoots arising from dormant buds on the stump crown (the callus zone), or on the lower portion of the trunk; sprouting is intensive and persistent (Brus 2011; Boer 2013)." - Valeriu-Norocel Nicolescu et al (2020) Ecology, growth and management of black locust (Robinia pseudoacacia L.), a non-native species integrated into European forests. J. For. Res. 31(4):1081–1101	Very high
32	6.05	Is the taxon dependent on the presence of another species (or specific habitat features) to complete its life cycle?	No	The species is well researched and there is no evidence any other taxon is necessary for to complete the life cycle.	High
33	6.06	Is the taxon likely to produce a large number of propagules?	Yes	I am not sure what exactly means "large number of propagules". I think 120-159 kg/ha is a lot, but I am not sure. The seed are light, so I would presume that it could mean that there are a lot of them, especiially for the tree. The literature says: "Black locust produces abundant seed crops with 120–150 kg of seeds ha− 1 in an average seed year, beginning from around 6 years of age (Rédei et al. 2001; Rédei 2013a, b, c, d). Good seed crops occur every 1–2 years in both Europe (Haralamb 1967; Rédei et al. 2012; Boer 2013; Los et al. 2014; Meyer-Münzer et al. 2015) and the USA (Huntley 1990). The seeds are light (20–23 g for 1000 seeds) and long-lived, mostly gravity- and wind-dispersed in the vicinity of the parent tree." - Valeriu-Norocel Nicolescu et al (2020) Ecology, growth and management of black locust (Robinia pseudoacacia L.), a non-native species integrated into European	Low
34	6.07	How many time units (days, months, years) does the taxon require to reach the age at first reproduction?	6	"Black locust produces abundant seed crops with 120–150 kg of seeds ha-1 in an average seed year, beginning from around 6 years of age (Rédei et al. 2001; Rédei 2013a, b, c, d)." - Valeriu-Norocel Nicolescu et al (2020) Ecology, growth and management of black locust (Robinia pseudoacacia L.), a non-native species integrated into European forests. J. For. Res.	Very high
7. Dispersal mechanisms					
35	7.01	How many potential vectors or pathways could the taxon use to disperse within the risk assessment area (with suitable habitats nearby)?	>1	1) Intentional introduction by man - Robinia is still planted because of its many positive qualities (honey-bearing, quality wood, stabilization of bare soils...) 2) Seed dispersal by wind – seeds can be unintentionally spread from cultivated or naturalized stands 3) By moving the soil in which there are parts of the roots damaged by mechanization, from which a new individual can grow	Very high
36	7.02	Will any of these vectors or pathways bring the taxon in close proximity to one or more protected areas?	Yes	The species is already so widley present that it is found in numerous protected areas throughout Europe (Natura 2000, national parks...)	Very high
37	7.03	Does the taxon have a specialised means of attachment such that it enhances the likelihood of dispersal?	No	The taxon has no morphological possibility to actively attach itself/seed to hard supstrat.	Very high
38	7.04	Is natural dispersal of the taxon likely to occur as seeds or spores in the risk assessment area?	Yes	Seed dispersal by wind – seeds can be unintentionally spread from cultivated or naturalized stands . "Long-distance spread of seeds by natural means is not very common (Boer 2013; Vítková et al. 2017); however, they have been found up to 67 m away from the parent tree (Morimoto et al. 2010, cited by van Loo et al. 2017)." - Valeriu-Norocel Nicolescu et al (2020) Ecology, growth and management of black locust (Robinia pseudoacacia L.), a non-native species integrated into European forests. J. For. Res.	Very high

39	7.05	Is natural dispersal of the taxon likely to occur as fragments or seedlings in the risk assessment area?	Yes	If the soil in which the R. pseudoacacia grew is transported, it is possible for Robinia trees to be transported to the new area. "...natural regeneration is primarily by root suckers. These are produced abundantly, particularly following mechanical damage to the root system (Boer 2013; Vítková et al. 2016) or soil disturbance (Savill 2013)." - Valeriu-Norocel Nicolescu et al (2020) Ecology, growth and management of black locust (Robinia pseudoacacia L.), a non-native species integrated into European forests. J. For. Res. 31(4):1081–1101	High
40	7.06	Are any life stages of the taxon likely to migrate into the risk assessment area for reproduction?	Yes	Question similar to the Q 38. "Black locust produces abundant seed crops with 120–150 kg of seeds ha− 1 in an average seed year, beginning from around 6 years of age (Rédei et al. 2001; Rédei 2013a, b, c, d). Good seed crops occur every 1–2 years in both Europe (Haralamb 1967; Rédei et al. 2012; Boer 2013; Los et al. 2014; Meyer-Münzer et al. 2015) and the USA (Huntley 1990). The seeds are light (20–23 g for 1000 seeds) and long-lived, mostly gravity- and wind-dispersed in the vicinity of the parent tree. Long-distance spread of seeds by natural means is not very common (Boer 2013; Vítková et al. 2017); however, they have been found up to 67 m away from the parent tree (Morimoto et al. 2010, cited by van Loo et al. 2017)." - Valeriu-Norocel Nicolescu et al (2020) Ecology, growth and management of black locust (Robinia pseudoacacia L.), a non-native species integrated	Very high
41	7.07	Are propagules of the taxon likely to be dispersed in the risk assessment area by other species?	No	I did not find that kind of data in literature.	High
42	7.08	Is dispersal of the taxon along any of the vectors or pathways mentioned in the previous seven Questions (35–41: i.e. either unintentional or intentional) likely to be	Yes	The species is deliberately planted in some areas of the researched area and this certainly greatly accelerates its spread.	Very high
43	7.09	Is dispersal of the taxon density dependent?	Yes	I wouldn't say that population density is crucial, but surely a larger number of seeds increases the possibility of the species	Medium
8. Tolerance attributes					
44	8.01	Is the taxon able to withstand being in water for extended periods at some stage of its life cycle?	No	It is not an aquatic species and I found no evidence that seed prefers or survives for long time in the water.	High
45	8.02	Is the taxon tolerant of a wide range of soil or air quality conditions?	No	Although the species can survive in areas with polluted air - "Our results indicate that the investigated species R. pseudoacacia and M. album can grow in metal polluted areas and that the contamination alters its physiological parameters." the species appears to be sensitive to pollution - "In our study, M. album and R. pseudoacacia proved to be sensitive species with the air pollution tolerance index lower than 11 and can be recommended as bioindicators." - Nadgórska-Socha et al (2016) Robinia pseudoacacia and Melandrium album in trace elements biomonitoring and air pollution tolerance index study. Int. J.	Medium
46	8.03	Can the taxon be controlled or eradicated in the wild with chemical, biological or other agents and means?	Yes	The taxon can theoretically be controlled or eradicated, but in the real life it is very expensive, so prevention and growth stimulation or preservation of already existing climazonal vegetation are the best solutions to suppress the spread of the species. "The combination of mechanical and chemical control involves (1) cutting or girdling the trees; (2) applying herbicide on the stump or the girdled area of the trunk (Vítková 2011; Vítková et al. 2016; https ://invas oras.pt/en/galle ry/ robin ia-pseud oacac ia-en). The chemical substances are as above (most cited: glyphosate) (Sabo 2000; https ://invas oras. pt/en/galle ry/robin ia-pseud oacac ia-en). Biological control makes use of domestic goats and cattle which defoliate black locust; in the native range after 4 years of browsing, all trees had been killed (Stone 2009). As complete eradication of black locust is not possible since such treatment is expensive because of continuous stump sprouting, one way to fulfill the objective of replacing the species is prolonging the rotation age until the trees become senescent and are replaced naturally by native species (Motta et al. 2009). Prevention measures, like avoidance of disturbances (e.g., soil disruption) that favor black locust colonization/dispersal by root suckers, are also possible (Boer 2013)." - Valeriu-Norocel Nicolescu et al (2020) Ecology, growth and management of black locust (Robinia pseudoacacia L.), a non-native species integrated	Very high
47	8.04	Is the taxon likely to benefit from environmental or human disturbance?	Yes	Natural (storm, strong wind, fire...) or human impacted degradation of forest areas opens the forest floor to sunlight, so it is more likely that these areas will be occupied by Robinia, if there are mature trees nearby. „…natural regeneration is primarily by root suckers. These are produced abundantly even by trees over 70 years old (Vítková et al. 2017) from superficial roots, particularly following mechanical damage to the root system (Boer 2013; Vítková et al. 2016) or soil disturbance (Savill 2013). … "However, natural regeneration from seed can be facilitated by seed wounding with heavy machinery (Tănăsescu 1961)." - Valeriu-Norocel Nicolescu et al (2020) Ecology, growth and management of black locust (Robinia pseudoacacia L.), a	Very high

48	8.05	Is the taxon able to tolerate soil acidity, salinity or other parameter levels that are higher or lower than those found in its usual environment?	Yes	In Europe, the plant seems to tolerate slightly more acidic soil, and is very drought tolerant. pH USA – https://www.srs.fs.usda.gov/pubs/misc/ag_654/volume_2/robinia/pseudoacacia.htm - "Soil pH from 4.6 to 8.2 and the amount of mineral nutrients present showed no relationship to growth."; pH Europe - "In Europe, the species has been introduced over a wide range of soils and tolerates diverse pH from extremely acid (3.2) to alkaline (8.8) (Vítková et al. 2015)." - Valeriu-Norocel Nicolescu et al (2020) Ecology, growth and management of black locust (Robinia pseudoacacia L.), a non-native species integrated into European forests. J. For. Res. 31(4):1081–1101 Climate – „In its native range, black locust grows in a humid climate (annual total precipitation 1020–1830 mm), with 150–210 frost-free days (Huntley 1990). In Europe, black locust behaves like a thermophilous species, i.e., it grows well where mean annual temperatures are over 8 °C (Roșu 1996; Rédei et al. 2017) and is very drought tolerant (Claessens et al. 2006; Grünewald et al. 2009; Mantovani et al. 2014; CRPF Nord Pas-de-Calais Picardie 2015)." - Valeriu-Norocel Nicolescu et al (2020) Ecology, growth and management of black locust (Robinia pseudoacacia L.), a non-native species integrated into European forests. J. For. Res.	High
49	8.06	Are there effective natural enemies of the taxon present in the risk assessment area?	No	There are some pests and diseases, but none of them is/could be used as effective controling factor. "In Europe, black locust appears to have high pest and disease resistance; however, in recent decades, several insects, fungi and semi-parasites have been reported to affect mature trees, seeds and seedlings." - Valeriu-Norocel Nicolescu et al (2020) Ecology, growth and management of black locust (Robinia pseudoacacia L.), a non-native species integrated into European forests. J. For. Res.	High
**C. Climate change**					
9. Climate change					
50	9.01	Under the predicted future climatic conditions, are the risks of entry into the risk assessment area posed by the taxon likely to increase, decrease or not change?	Not applicable	The species is already present in RA.	Very high
51	9.02	Under the predicted future climatic conditions, are the risks of establishment within the risk assessment area posed by the taxon likely to increase, decrease or not change?	Increase	"As its potential to adapt to predicted climate changes, particularly drought, seems to be high, the importance of black locust, a species both "beloved and despised" (Vítková et al. 2017) in Europe, is expected to increase in the future. However, not only will its importance increase under expected climate conditions, but also its invasiveness potential." - Valeriu-Norocel Nicolescu et al (2020) Ecology, growth and management of black locust (Robinia pseudoacacia L.), a non-native species integrated into European forests. J. For. Res. 31(4):1081–1101. "We showed that the majority of the range changes expected in 2070 will occur as early as 2050. In comparison to previous studies, we demonstrated a greater eastward shift of potential niches of this species and a greater decline of potential niches in Southern Europe. Consequently, future climatic conditions will likely favor the occurrence of R. pseudoacacia in Central and Northeastern Europe where this species is still absent or relatively rare."- Puchałka R. et al (2020) Black locust (Robinia pseudoacacia L.) range contraction and expansion in Europe under changing	Very high
52	9.03	Under the predicted future climatic conditions, are the risks of dispersal within the risk assessment area posed by the taxon likely to increase, decrease or not change?	Increase	"As its potential to adapt to predicted climate changes, particularly drought, seems to be high, the importance of black locust, a species both "beloved and despised" (Vítková et al. 2017) in Europe, is expected to increase in the future. However, not only will its importance increase under expected climate conditions, but also its invasiveness potential." - Valeriu-Norocel Nicolescu et al (2020) Ecology, growth and management of black locust (Robinia pseudoacacia L.), a non-native species integrated into European forests. J. For. Res. 31(4):1081–1101. "We showed that the majority of the range changes expected in 2070 will occur as early as 2050. In comparison to previous studies, we demonstrated a greater eastward shift of potential niches of this species and a greater decline of potential niches in Southern Europe. Consequently, future climatic conditions will likely favor the occurrence of R. pseudoacacia in Central and Northeastern Europe where this species is still absent or relatively rare."- Puchałka R. et al (2020) Black locust (Robinia pseudoacacia L.) range contraction and expansion in Europe under changing	Very high

53	9.04	Under the predicted future climatic conditions, what is the likely magnitude of future potential impacts by the taxon on biodiversity or ecosystem integrity and status?	Higher	"As its potential to adapt to predicted climate changes, particularly drought, seems to be high, the importance of black locust, a species both "beloved and despised" (Vítková et al. 2017) in Europe, is expected to increase in the future. However, not only will its importance increase under expected climate conditions, but also its invasiveness potential." - Valeriu-Norocel Nicolescu et al (2020) Ecology, growth and management of black locust (Robinia pseudoacacia L.), a non-native species integrated into European forests. J. For. Res. 31(4):1081–1101. "We showed that the majority of the range changes expected in 2070 will occur as early as 2050. In comparison to previous studies, we demonstrated a greater eastward shift of potential niches of this species and a greater decline of potential niches in Southern Europe. Consequently, future climatic conditions will likely favor the occurrence of R. pseudoacacia in Central and Northeastern Europe where this species is still absent or relatively rare."- Puchałka R. et al (2020) Black locust (Robinia pseudoacacia L.) range contraction and expansion in Europe under changing	Very high
54	9.05	Under the predicted future climatic conditions, what is the likely magnitude of future potential impacts by the taxon on ecosystem structure or function?	Higher	The range of the species will probably increase and thus the impact on the ecosystem od RA. "As its potential to adapt to predicted climate changes, particularly drought, seems to be high, the importance of black locust, a species both "beloved and despised" (Vítková et al. 2017) in Europe, is expected to increase in the future. However, not only will its importance increase under expected climate conditions, but also its invasiveness potential." - Valeriu-Norocel Nicolescu et al (2020) Ecology, growth and management of black locust (Robinia pseudoacacia L.), a non-native species integrated into European forests. J. For. Res.	High
55	9.06	Under the predicted future climatic conditions, what is the likely magnitude of future potential impacts by the taxon on ecosystem services and related socio-	No change	Although the range of Robinia will probaby expand, judging by the species' impact so far, I would say the expansion should not affect socio-economic factors.	Medium

Statistics	
**Scores**	
**BRA**	**42.0**
**BRA Outcome**	**High**
**BRA+CCA**	**50.0**
**BRA+CCA Outcome**	**High**
**Score partition**	
**A. Biogeography/Historical**	**14.0**
1. Domestication/Cultivation	*2.0*
2. Climate, distribution and introduction risk	*2.0*
3. Invasive elsewhere	*10.0*
**B. Biology/Ecology**	**28.0**
4. Undesirable (or persistence) traits	*10.0*
5. Resource exploitation	*7.0*
6. Reproduction	*3.0*
7. Dispersal mechanisms	*5.0*
8. Tolerance attributes	*3.0*
**C. Climate change**	**8.0**
9. Climate change	*8.0*
**Answered Questions**	
Total	***55***
**A. Biogeography/Historical**	**13**
1. Domestication/Cultivation	*3*
2. Climate, distribution and introduction risk	*5*
3. Invasive elsewhere	*5*
**B. Biology/Ecology**	**36**
4. Undesirable (or persistence) traits	*12*
5. Resource exploitation	*2*
6. Reproduction	*7*
7. Dispersal mechanisms	*9*
8. Tolerance attributes	*6*
**C. Climate change**	**6**
9. Climate change	*6*
**Sectors affected**	
**Commercial**	**11**
**Environmental**	**17**
**Species or population nuisance traits**	**27**

Thresholds	
**BRA**	**41**
**BRA+CCA**	**41**
**Confidence**	
**BRA+CCA**	**0.85**
**BRA**	**0.85**
**CCA**	**0.88**

Date and Time
**09/08/2024 15:32:19**

TPS-ISK v2

**Taxon and Assessor details**	
Category	Angiosperms
Taxon name	*Styphnolobium japonicum*
Common name	Chinese Scholar Tree (Pagoda Tree)
Assessor	Dario Hruševar
**Risk screening context**	
Reason and socio-economic benefits	ornamental tree; ethnobotanical uses
Risk assessment area	Continental Europe
Taxonomy	fam. Fabaceae; Basionym: Sophora japonica L.
Native range	Asia (mainly temperat, Chinae)
Introduced range	all other continents
URL	

			Response	Justification (references or other information)	Confidence
**A. Biogeography/Historical**					
1. Domestication/Cultivation					
1	1.01	Has the taxon been the subject of domestication?	Yes	Styphnolobium japonicum (L.) Schott in GBIF Secretariat (2023). GBIF Backbone Taxonomy. Checklist dataset https://doi.org/10.15468/39omei accessed via GBIF.org on 2024-07-28.; https://www.sciencedirect.com/topics/pharmacology-toxicology-and-pharmaceutical-science/styphnolobium-japonicum	Very high
2	1.02	Is the taxon harvested in the wild and likely to be sold or used in its live form?	Yes	https://www.sciencedirect.com/topics/pharmacology-toxicology-and-pharmaceutical-science/styphnolobium-japonicum	High
3	1.03	Does the taxon have invasive races, varieties, sub-taxa or congeners?	Yes	https://bugwoodcloud.org/CDN/sceppc/publications/Styphnolobium_japonicum2012NCREPMT-1.pdf	Very high
2. Climate, distribution and introduction risk					
4	2.01	How similar are the climatic conditions between the risk assessment area and the taxon's native range?	Medium	Peel et al. 2007	Medium
5	2.02	What is the quality of the climate matching data?	Medium	Peel et al. 2007	Medium
6	2.03	Is the taxon known to be present outside of cultivation in the risk assessment area?	Yes	casual: Musarella, C.M.; Laface, V.L.A.; Angiolini, C.; Bacchetta, G.; Bajona, E.; Banfi, E.; Barone, G.; Biscotti, N.; Bonsanto, D.; Calvia, G.; et al. New Alien Plant Taxa for Italy and Europe: An Update. Plants 2024, 13, 620.	High
7	2.04	How many potential vectors could the taxon use to enter the risk assessment area?	>1	https://en.wikipedia.org/wiki/Styphnolobium_japonicum	High
8	2.05	Is the taxon currently found in close proximity to, and likely to enter, the risk assessment area in the near future (e.g. unintentional or intentional introductions)?	Not applicable	CASUAL: Styphnolobium japonicum (L.) Schott in GBIF Secretariat (2023). GBIF Backbone Taxonomy. Checklist dataset https://doi.org/10.15468/39omei accessed via GBIF.org on 2024-07-28.; Musarella, C.M.; Laface, V.L.A.; Angiolini, C.; Bacchetta, G.; Bajona, E.; Banfi, E.; Barone, G.; Biscotti, N.; Bonsanto, D.; Calvia, G.; et al. New Alien Plant Taxa for Italy and Europe: An Update. Plants 2024, 13, 620.	Very high
3. Invasive elsewhere					
9	3.01	Has the taxon become naturalised outside its native range?	No	CASUAL (Styphnolobium japonicum (L.) Schott in GBIF Secretariat (2023). GBIF Backbone Taxonomy. Checklist dataset https://doi.org/10.15468/39omei accessed via GBIF.org on 2024-07-28.; Musarella, C.M.; Laface, V.L.A.; Angiolini, C.; Bacchetta, G.; Bajona, E.; Banfi, E.; Barone, G.; Biscotti, N.; Bonsanto, D.; Calvia, G.; et al. New Alien Plant Taxa for Italy and Europe: An Update. Plants 2024, 13, 620.	High
10	3.02	In the taxon's introduced range, are there any known adverse impacts to wild or commercial species?	No	No data found	Medium
11	3.03	In the taxon's introduced range, are there any known adverse impacts to agriculture or forestry?	No	No data found	Medium
12	3.04	In the taxon's introduced range, are there any known adverse impacts to ecosystem	No	No data found (professional judgement)	Medium
13	3.05	In the taxon's introduced range, are there any known adverse socio-economic impacts?	No	No data found (professional judgement)	Medium
**B. Biology/Ecology**					
4. Undesirable (or persistence) traits					
14	4.01	Is the taxon likely to be poisonous or pose other risks to human health?	No	No data found (professional judgement)	High
15	4.02	Is the taxon likely to suppress the growth of one or more native species?	No	No data found (professional judgement)	Medium
16	4.03	Are there any threatened or protected native species that the taxon would parasitise in the risk assessment area?	No	No data found (professional judgement)	Medium
17	4.04	Is the taxon adaptable in terms of climatic and other environmental conditions, thus enhancing its potential persistence if it has invaded or is likely to invade the risk	Yes	https://bugwoodcloud.org/CDN/sceppc/publications/Styphnolobium_japonicum2012NCREPMT-1.pdf	Very high
18	4.05	Is the taxon likely to disrupt terrestrial ecosystem function if it has invaded or is likely to invade the risk assessment area?	No	Professional judgement based on eg. Musarella, C.M.; Laface, V.L.A.; Angiolini, C.; Bacchetta, G.; Bajona, E.; Banfi, E.; Barone, G.; Biscotti, N.; Bonsanto, D.; Calvia, G.; et al. New Alien Plant Taxa for Italy and Europe: An Update. Plants 2024, 13, 620. https://doi.org/10.3390/plants13050620	Medium
19	4.06	Is the taxon likely to exert adverse impacts on ecosystem services in the risk	No	No data found (professional judgement)	Medium

20	4.07	Is the taxon likely to host or function as a vector for recognised pests and pathogens that are present in the risk assessment area?	No	No data found (professional judgement)	Medium
21	4.08	Is the taxon likely to host or function as a vector for recognised pests and pathogens that are absent in the risk assessment area?	No	No data found (professional judgement)	Medium
22	4.09	Is the taxon likely to be released from cultivation?	Yes	Musarella, C.M.; Laface, V.L.A.; Angiolini, C.; Bacchetta, G.; Bajona, E.; Banfi, E.; Barone, G.; Biscotti, N.; Bonsanto, D.; Calvia, G.; et al. New Alien Plant Taxa for Italy and Europe: An Update. Plants 2024, 13, 620.	Medium
23	4.10	Is the taxon versatile in habitat use?	Yes	https://www.missouribotanicalgarden.org/PlantFinder/PlantFinderDetails.aspx?taxonid=254548	High
24	4.11	Is it likely that the taxon's mode of existence or behaviours will reduce habitat quality for native species?	No	No data found (professional judgement)	Low
25	4.12	Is the taxon likely to maintain a viable population even when present in low densities (or persist in adverse conditions by way of a dormant form)?	No	No data found (professional judgement)	Low
5. Resource exploitation					
26	5.01	Is the taxon likely to put threatened or protected native species under competitive pressure in the risk assessment area?	No	No data found (professional judgement)	Low
27	5.02	Is the taxon likely to sequester resources to the detriment of native species in the risk assessment area?	No	No data found (professional judgement)	Low
6. Reproduction					
28	6.01	Is the taxon likely to exhibit changes in reproductive strategy in response to environmental conditions?	No	No data found (professional judgement)	Medium
29	6.02	Is the taxon likely to produce viable propagules in the risk assessment area?	Yes	eg. Musarella, C.M.; Laface, V.L.A.; Angiolini, C.; Bacchetta, G.; Bajona, E.; Banfi, E.; Barone, G.; Biscotti, N.; Bonsanto, D.; Calvia, G.; et al. New Alien Plant Taxa for Italy and Europe: An Update. Plants 2024, 13, 620.	High
30	6.03	Is the taxon likely to hybridise with native species under natural conditions?	No	No data found	Low
31	6.04	Is the taxon likely to be hermaphroditic or to exhibit asexual reproduction?	Yes	https://en.wikipedia.org/wiki/Styphnolobium_japonicum; https://bugwoodcloud.org/CDN/sceppc/publications/Styphnolobium_japonicum2012NCREPMT-1.pdf	Very high
32	6.05	Is the taxon dependent on the presence of another species (or specific habitat features) to complete its life cycle?	No	No data found (professional judgement)	Medium
33	6.06	Is the taxon likely to produce a large number of propagules?	Yes	https://bugwoodcloud.org/CDN/sceppc/publications/Styphnolobium_japonicum2012NCREPMT-1.pdf; https://www.missouribotanicalgarden.org/PlantFinder/PlantFinderDetails.aspx?taxonid=254548	High
34	6.07	How many time units (days, months, years) does the taxon require to reach the age at first reproduction?	>10	https://www.vdberk.com/trees/styphnolobium-japonicum/;	Very high
7. Dispersal mechanisms					
35	7.01	How many potential vectors or pathways could the taxon use to disperse within the risk assessment area (with suitable habitats nearby)?	>1	eg. https://bugwoodcloud.org/CDN/sceppc/publications/Styphnolobium_japonicum2012NCREPMT-1.pdf; https://www.vdberk.com/trees/styphnolobium-japonicum/; Musarella, C.M.; Laface, V.L.A.; Angiolini, C.; Bacchetta, G.; Bajona, E.; Banfi, E.; Barone, G.; Biscotti, N.; Bonsanto, D.; Calvia, G.; et al. New Alien Plant Taxa for Italy and Europe: An Update. Plants 2024, 13, 620.	Very high
36	7.02	Will any of these vectors or pathways bring the taxon in close proximity to one or more protected areas?	Yes	eg. https://bugwoodcloud.org/CDN/sceppc/publications/Styphnolobium_japonicum2012NCREPMT-1.pdf; https://www.vdberk.com/trees/styphnolobium-japonicum/; Musarella, C.M.; Laface, V.L.A.; Angiolini, C.; Bacchetta, G.; Bajona, E.; Banfi, E.; Barone, G.; Biscotti, N.; Bonsanto, D.; Calvia, G.; et al. New Alien Plant Taxa for Italy and Europe: An Update. Plants 2024, 13, 620.	Very high
37	7.03	Does the taxon have a specialised means of attachment such that it enhances the likelihood of dispersal?	No	eg. https://www.missouribotanicalgarden.org/PlantFinder/PlantFinderDetails.aspx?taxonid=254548	Very high
38	7.04	Is natural dispersal of the taxon likely to occur as seeds or spores in the risk assessment area?	Yes	Musarella, C.M.; Laface, V.L.A.; Angiolini, C.; Bacchetta, G.; Bajona, E.; Banfi, E.; Barone, G.; Biscotti, N.; Bonsanto, D.; Calvia, G.; et al. New Alien Plant Taxa for Italy and Europe: An Update. Plants 2024, 13, 620.	Very high
39	7.05	Is natural dispersal of the taxon likely to occur as fragments or seedlings in the risk assessment area?	Yes	Musarella, C.M.; Laface, V.L.A.; Angiolini, C.; Bacchetta, G.; Bajona, E.; Banfi, E.; Barone, G.; Biscotti, N.; Bonsanto, D.; Calvia, G.; et al. New Alien Plant Taxa for Italy and Europe: An Update. Plants 2024, 13, 620.	Very high
40	7.06	Are any life stages of the taxon likely to migrate into the risk assessment area for reproduction?	Yes	Musarella, C.M.; Laface, V.L.A.; Angiolini, C.; Bacchetta, G.; Bajona, E.; Banfi, E.; Barone, G.; Biscotti, N.; Bonsanto, D.; Calvia, G.; et al. New Alien Plant Taxa for Italy and Europe: An Update. Plants 2024, 13, 620.	Very high
41	7.07	Are propagules of the taxon likely to be dispersed in the risk assessment area by other species?	No	No data found (professional judgement)	Medium

42	7.08	Is dispersal of the taxon along any of the vectors or pathways mentioned in the previous seven Questions (35–41: i.e. either unintentional or intentional) likely to be	No	No data found (professional judgement)	Medium
43	7.09	Is dispersal of the taxon density dependent?	No	No data found (professional judgement)	Medium
8. Tolerance attributes					
44	8.01	Is the taxon able to withstand being in water for extended periods at some stage of its life cycle?	No	No data found (professional judgement)	Medium
45	8.02	Is the taxon tolerant of a wide range of soil or air quality conditions?	Yes	https://bugwoodcloud.org/CDN/sceppc/publications/Styphnolobium_japonicum2012NCREPMT-1.pdf;	Very high
46	8.03	Can the taxon be controlled or eradicated in the wild with chemical, biological or other agents and means?	Yes	https://bugwoodcloud.org/CDN/sceppc/publications/Styphnolobium_japonicum2012NCREPMT-1.pdf	Very high
47	8.04	Is the taxon likely to benefit from environmental or human disturbance?	No	No data found (professional judgement)	Low
48	8.05	Is the taxon able to tolerate soil acidity, salinity or other parameter levels that are higher or lower than those found in its usual	Yes	https://bugwoodcloud.org/CDN/sceppc/publications/Styphnolobium_japonicum2012NCREPMT-1.pdf	Very high
49	8.06	Are there effective natural enemies of the taxon present in the risk assessment area?	No	No data found (professional judgement)	Low
**C. Climate change**					
9. Climate change					
50	9.01	Under the predicted future climatic conditions, are the risks of entry into the risk assessment area posed by the taxon likely to increase, decrease or not change?	Decrease	No data found (professional judgement)	Medium
51	9.02	Under the predicted future climatic conditions, are the risks of establishment within the risk assessment area posed by the taxon likely to increase, decrease or not	No change	No data found (professional judgement)	Medium
52	9.03	Under the predicted future climatic conditions, are the risks of dispersal within the risk assessment area posed by the taxon likely to increase, decrease or not change?	No change	No data found (professional judgement)	Low
53	9.04	Under the predicted future climatic conditions, what is the likely magnitude of future potential impacts by the taxon on biodiversity or ecosystem integrity and	No change	No data found (professional judgement)	Medium
54	9.05	Under the predicted future climatic conditions, what is the likely magnitude of future potential impacts by the taxon on ecosystem structure or function?	No change	No data found (professional judgement)	Medium
55	9.06	Under the predicted future climatic conditions, what is the likely magnitude of future potential impacts by the taxon on ecosystem services and related socio-	No change	No data found (professional judgement)	Medium

**Statistics**	
**Scores**	
**BRA**	**15.0**
**BRA Outcome**	**Medium**
**BRA+CCA**	**13.0**
**BRA+CCA Outcome**	**Medium**
**Score partition**	
**A. Biogeography/Historical**	**6.0**
1. Domestication/Cultivation	*4.0*
2. Climate, distribution and introduction risk	*2.0*
3. Invasive elsewhere	*0.0*
**B. Biology/Ecology**	**9.0**
4. Undesirable (or persistence) traits	*3.0*
5. Resource exploitation	*0.0*
6. Reproduction	*2.0*
7. Dispersal mechanisms	*3.0*
8. Tolerance attributes	*1.0*
**C. Climate change**	**-2.0**
9. Climate change	*-2.0*
**Answered Questions**	
Total	***55***
**A. Biogeography/Historical**	**13**
1. Domestication/Cultivation	*3*
2. Climate, distribution and introduction risk	*5*
3. Invasive elsewhere	*5*
**B. Biology/Ecology**	**36**
4. Undesirable (or persistence) traits	*12*
5. Resource exploitation	*2*
6. Reproduction	*7*
7. Dispersal mechanisms	*9*
8. Tolerance attributes	*6*
**C. Climate change**	**6**
9. Climate change	*6*
**Sectors affected**	
**Commercial**	**6**
**Environmental**	**1**
**Species or population nuisance traits**	**10**

Thresholds	
BRA	41
BRA+CCA	41
**Confidence**	
BRA+CCA	0.64
BRA	0.66
CCA	0.46

Date and Time
12/08/2024 17:27:00

TPS-ISK v2

Taxon and Assessor details	
Category	Gymnosperms
Taxon name	*Cedrus libani*
Common name	Cedar of Lebanon
Assessor	Ivana Vitasović Kosić
**Risk screening context**	
Reason and socio-economic benefits	
Risk assessment area	Continental Europe
Taxonomy	
Native range	
Introduced range	
URL	

			Response	Justification (references or other information)	Confidence
**A. Biogeography/Historical**					
1. Domestication/Cultivation					
1	1.01	Has the taxon been the subject of domestication?	Yes	species of tree in the genus Cedrus, a part of the pine family, native to the mountains of the Eastern Mediterranean basin. It is a large evergreen conifer that has great religious and historical significance in the cultures of the Middle East, and is referenced many times in the literature of ancient civilisations. It is the national emblem of Lebanon and is widely used as an ornamental tree in parks and gardens. https://en.wikipedia.org/wiki/Cedrus_libani#cite_note-18 the Epic of Gilgamesh, one of the earliest great works of literature, the Sumerian hero Gilgamesh and his friend Enkidu travel to the legendary Cedar Forest to kill its guardian and cut down its trees. While early versions of the story place the forest in Iran, later Babylonian accounts of the story place the Cedar Forest in the Lebanon. from: Sherratt, Susan; Bennet, John (2017). Archaeology and Homeric epic. Oxford: Oxbow Books. p. 127. ISBN 9781785702969. OCLC 959610992 already 5000 years! from: Boydak, M. (2003). Regeneration of Lebanon cedar (Cedrus libani A. Rich.) on karstic lands in Turkey. Forest ecology and management, 178(3), 231-243. Özcan, A. U., & Çiçek, K. (2023). How long do we think humans have been planting forests? A case study with Cedrus libani A. Rich. New Forests, 54(1), 49-65. said: Secondly, the species had to be kept away from competition. This would require silviculture and protection activities. However, what is most surprising is that if all this is true, then the species must [illegible]	Very high
2	1.02	Is the taxon harvested in the wild and likely to be sold or used in its live form?	Yes	yes, it is ornamental tree, solding out https://www.woodlandtrust.org.uk/trees-woods-and-wildlife/british-trees/a-z-of-british-trees/cedar/ Cedar wood is prized for its fine grain, attractive yellow color, and fragrance. It is exceptionally durable and immune to insect ravages. Wood from C. libani has a density of 560 kg/m3; it is used for furniture, construction, and handicrafts. In Turkey, shelterwood cutting and clearcutting techniques are used to harvest timber and promote uniform forest regeneration. Cedar resin (cedria) and cedar essential oil (cedrum) are prized extracts from the timber and cones of the cedar tree.[CABI 2013, p. 117][Coxe 1808, p. CED]	High
3	1.03	Does the taxon have invasive races, varieties, sub-taxa or congeners?	No	Boydak, M. (2003). Regeneration of Lebanon cedar (Cedrus libani A. Rich.) on karstic lands in Turkey. Forest ecology and management, 178(3), 231-243.	High
2. Climate, distribution and introduction risk					
4	2.01	How similar are the climatic conditions between the risk assessment area and the taxon's native range?	High	Messinger, J., Güney, A., Zimmermann, R. et al. Cedrus libani: A promising tree species for Central European forestry facing climate change?. Eur J Forest Res 134, 1005–1017 (2015). https://doi.org/10.1007/s10342-015-0905-z Messinger et al already did successfully experiment in Beyrouth (Gemany)	High
5	2.02	What is the quality of the climate matching data?	High	Messinger, J., Güney, A., Zimmermann, R. et al. Cedrus libani: A promising tree species for Central European forestry facing climate change?. Eur J Forest Res 134, 1005–1017 (2015). https://doi.org/10.1007/s10342-015-0905-z	High
6	2.03	Is the taxon known to be present outside of cultivation in the risk assessment area?	Yes	in Spain, Germany, Serbia, Italia https://www.gbif.org/species/5284682	Very high
7	2.04	How many potential vectors could the taxon use to enter the risk assessment area?	One	it is ornamental plant, and planted on purpose. Boydak, M. (2003). Regeneration of Lebanon cedar (Cedrus libani A. Rich.) on karstic lands in Turkey. Forest ecology and management, 178(3), 231-243. Messinger, J., Güney, A., Zimmermann, R. et al. Cedrus libani: A promising tree species for Central European forestry facing climate change?. Eur J Forest Res 134, 1005–1017 (2015). https://doi.org/10.1007/s10342-015-0905-z Višnjić, Ć., Čilaš, M., & Ivojević, S. (2022). Ecological and silvicultural characteristics and the possibility of introducing Lebanese cedar (Cedrus libani A. Rich.) in BiH facing climate change.Našpe šume, 53-67.	High
8	2.05	Is the taxon currently found in close proximity to, and likely to enter, the risk assessment area in the near future (e.g. unintentional or intentional introductions)?	Not applicable	Messinger, J., Güney, A., Zimmermann, R. et al. Cedrus libani: A promising tree species for Central European forestry facing climate change?. Eur J Forest Res 134, 1005–1017 (2015). https://doi.org/10.1007/s10342-015-0905-z	Very high
3. Invasive elsewhere					

9	3.01	Has the taxon become naturalised outside its native range?	No	seeds have good germination, sporadic natural regeneration from seeds has been observed next to mature trees in Germany recently from 2012 (Messinger et al , 2015) Messinger, J., Güney, A., Zimmermann, R. et al. Cedrus libani: A promising tree species for Central European forestry facing climate change?. Eur J Forest Res 134, 1005–1017 (2015). https://doi.org/10.1007/s10342-015-	Very high
10	3.02	In the taxon's introduced range, are there any known adverse impacts to wild or commercial species?	No	my opinion	Medium
11	3.03	In the taxon's introduced range, are there any known adverse impacts to agriculture or forestry?	No	completely opposite: Xiao, S., Li, S., Wang, X., Chen, L., & Su, T. (2022). Cedrus distribution change: past, present, and future. Ecological Indicators, 142, 109159. said: In general, this study identifies the key climate factors restricting the natural distribution of Cedrus. It shows that the distribution of Cedrus would be reduced in response to global climate change in the future, which indicates an urgent need for the protection and management of Cedrus populations.	Very high
12	3.04	In the taxon's introduced range, are there any known adverse impacts to ecosystem services?	No	no , this taxa is useful against air pollution: Onder, S., & Dursun, S. (2006). Air borne heavy metal pollution of Cedrus libani (A. Rich.) in the city centre of Konya (Turkey). Atmospheric Environment, 40(6), 1122-1133. The contents of heavy metal accumulated by air pollution in the cedar tree needles from the green area of Konya city centre were measured by chemical analyses. The needle samples were collected from two types of trees (about 10–15 and 20–25-years-old trees) from eight different pollution regions for two different sampling periods. Results of the present study showed that accumulations of heavy metals in the old trees were generally higher than those of young	High
13	3.05	In the taxon's introduced range, are there any known adverse socio-economic impacts?	No	no sucjh data	High
**B. Biology/Ecology**					
4. Undesirable (or persistence) traits					
14	4.01	Is the taxon likely to be poisonous or pose other risks to human health?	No	no such data, in my opinion, the species is not poisonous, it does not pose a danger to human health, especially if we consider that it has existed for 5000 years	High
15	4.02	Is the taxon likely to suppress the growth of one or more native species?	No	it is harmful species Two of these cedars – the Atlas and the Lebanon – run the risk of extinction in the wild. Centuries of over-harvesting, over-grazing of regenerating saplings and wild fires have taken their toll on stocks in their native mountainous terrain. Of the vast cedar forests that once clothed most of Lebanon's highlands, only roughly 17 km² of untouched climax stands remain, in scattered groves. https://www.forestryjournal.co.uk/features/forestry-journal-	High
16	4.03	Are there any threatened or protected native species that the taxon would parasitise in the risk assessment area?	No	Xiao, S., Li, S., Wang, X., Chen, L., & Su, T. (2022). Cedrus distribution change: past, present, and future. Ecological Indicators, 142, 109159. López-Tirado, J., Vessella, F., Stephan, J., Ayan, S., Schirone, B., & Hidalgo, P. J. (2021). Effect of climate change on potential distribution of Cedrus libani A. Rich in the twenty-first century: an Ecological Niche Modeling assessment. New Forests, 52, 363-376.	High
17	4.04	Is the taxon adaptable in terms of climatic and other environmental conditions, thus enhancing its potential persistence if it has invaded or is likely to invade the risk assessment area?	No	Migration might be necessary for C. libani to cope with projected change. A similar trendhas been detected in Cedrus atlantica populations in Algeria where its southern distribution limit shows a high level of tree mortality during recent decades (Slimani et al. 2014).Factors that are correlated with altitude seem to be the most significant (Körner 2007). This is consistent with other tree species studied in the Mediterranean basin (López-Tirado et al.2018). Habitat suitability area results wider in the forecasted scenarios than in the presentperiod. These results can be useful to manage C. libani natural and non-natural stands.Afforestation programs by ENM models could be carried out to deal with the expected climate change together with other disciplines such as remote sensing (Camarretta et al. 2020) from: López-Tirado, J., Vessella, F., Stephan, J., Ayan, S., Schirone, B., & Hidalgo, P. J. (2021). Effect of climate change on potential distribution of Cedrus libani A. Rich in the twenty-first century: an Ecological Niche Modeling assessment. New Forests, 52, 363-376. Highlights Winter precipitation and temperature are critical for Cedrus Climate change can significantly affect the distribution of Cedrus Distribution of Cedrus would be reduced in response to global climate change in the future. from: Xiao, S., Li, S., Wang, X., Chen, L., & Su, T. (2022). Cedrus distribution change: past, present, and future. Ecological Indicators, 142, 109159 Status of C. libani: Two of these cedars – the Atlas and the Lebanon – run the risk of extinction in the wild. Centuries of over-harvesting, over-grazing of regenerating saplings and wild fires have taken their toll on stocks in their native mountainous terrain. Of the vast cedar forests that once clothed most of Lebanon's highlands, only roughly 17 km² of untouched climax stands remain, in scattered [illegible]	Very high
18	4.05	Is the taxon likely to disrupt terrestrial ecosystem function if it has invaded or is likely to invade the risk assessment area?	No	literature said no.	High

19	4.06	Is the taxon likely to exert adverse impacts on ecosystem services in the risk assessment area?	No	completely opposite, it is welcome for planting it in Central Europeaun forests, Messinger, J., Güney, A., Zimmermann, R. et al. Cedrus libani: A promising tree species for Central European forestry facing climate change?. Eur J Forest Res 134, 1005–1017 (2015). https://doi.org/10.1007/s10342-015-0905-z	High
20	4.07	Is the taxon likely to host or function as a vector for recognised pests and pathogens that are present in the risk assessment area?	No	Messinger, J., Güney, A., Zimmermann, R. et al. Cedrus libani: A promising tree species for Central European forestry facing climate change?. Eur J Forest Res 134, 1005–1017 (2015). https://doi.org/10.1007/s10342-015-0905-z	High
21	4.08	Is the taxon likely to host or function as a vector for recognised pests and pathogens that are absent in the risk assessment area?	No	Messinger, J., Güney, A., Zimmermann, R. et al. Cedrus libani: A promising tree species for Central European forestry facing climate change?. Eur J Forest Res 134, 1005–1017 (2015). https://doi.org/10.1007/s10342-015-0905-z	Medium
22	4.09	Is the taxon likely to be released from cultivation?	No	Thus, its promising potential for establishing stable and productive forest stands in Central Europe under a changing climate should be confirmed in further studies. Messinger, J., Güney, A., Zimmermann, R. et al. Cedrus libani: A promising tree species for Central European forestry facing climate change?. Eur J Forest Res 134, 1005–1017 (2015). https://doi.org/10.1007/s10342-015-0905-z Višnjić, Ć., Čilaš, M., & Ivojević, S. (2022). Ecological and silvicultural characteristics and the possibility of introducing Lebanese cedar (Cedrus libani A. Rich.) in BiH facing climate	Medium
23	4.10	Is the taxon versatile in habitat use?	Yes	It is a light-demanding, calciphile coniferous species that is most vigorous when planted in montane regions.	Medium
24	4.11	Is it likely that the taxon's mode of existence or behaviours will reduce habitat quality for native species?	Yes	Onder, S., & Dursun, S. (2006). Air borne heavy metal pollution of Cedrus libani (A. Rich.) in the city centre of Konya (Turkey). Atmospheric Environment, 40(6), 1122-1133. C. libani is an air purifier and can be dangerous to some organisms if consumed.	Medium
25	4.12	Is the taxon likely to maintain a viable population even when present in low densities (or persist in adverse conditions by way of a dormant form)?	No	I'm not sure, did'n find references.	Medium
5. Resource exploitation					
26	5.01	Is the taxon likely to put threatened or protected native species under competitive pressure in the risk assessment area?	No	NO, this species does not grow fast. The species run the risk of extinction in the wild. https://www.forestryjournal.co.uk/features/forestry-journal-features/24231782.cedrus-libani-future-hold-lebanons-cedar-	Medium
27	5.02	Is the taxon likely to sequester resources to the detriment of native species in the risk assessment area?	No	my opinion.	Medium
6. Reproduction					
28	6.01	Is the taxon likely to exhibit changes in reproductive strategy in response to environmental conditions?	Yes	Messinger, J., Güney, A., Zimmermann, R. et al. Cedrus libani: A promising tree species for Central European forestry facing climate change?. Eur J Forest Res 134, 1005–1017 (2015). https://doi.org/10.1007/s10342-015-0905-z Considering climate change, the discussion intensifies whether and to what extent exotic tree species should be taken into account for forest cultivation, especially when indigenous species are no longer able to fulfill essential forest functions. In this study, for the first time growth potential of Cedrus libani was evaluated under climatic conditions in Central Europe (Bayreuth, Germany). The sampled trees exhibited extraordinary growth with tree ring widths averaging 4.9 mm year−1 during the past 23 years. A continuously available soil water supply enhanced radial stem growth. Thus, growth declined during the dry year of 2003, but recovered to average values the following year. Our results confirm that C. libani is a light-demanding species which is sensitive to competition and which shows a typical age trend. In a second study, we compared cambial growth in Bayreuth with a natural stand in Elmali (Turkey) in 2009. Cambial growing season in Bayreuth was 45 days longer, and radial growth rates in Bayreuth were four times higher than in Elmali. Interestingly, C. libani maintained a slow but continuous radial growth at Elmali even during the dry summer period, confirming its exceptional drought tolerance. Our results indicate a high adaption of C. libani to current and future climate conditions in Central Europe. It tolerates extreme cold in winter and prolonged droughts during [illegible]	Medium
29	6.02	Is the taxon likely to produce viable propagules in the risk assessment area?	Yes	seeds have good germination, sporadic natural regeneration from seed was observed next to mature trees in Germany as recently as 2012 (Messinger et al, 2015) Messinger, J., Güney, A., Zimmermann, R. et al. Cedrus libani: A promising tree species for Central European forestry facing climate change?. Eur J Forest Res 134, 1005–1017 (2015). https://doi.org/10.1007/s10342-015-	Medium
30	6.03	Is the taxon likely to hybridise with native species under natural conditions?	No	it is not known til now.	Medium
31	6.04	Is the taxon likely to be hermaphroditic or to exhibit asexual reproduction?	No	it is not known till now.	Medium
32	6.05	Is the taxon dependent on the presence of another species (or specific habitat features) to complete its life cycle?	No	i didn't find reference about.	Medium

33	6.06	Is the taxon likely to produce a large number of propagules?	Yes	in the first years of cultivation up to 10,000 plants per ha, a controlled fire is carried out, after that it can be up to 50,000 plants/ha (Boydak, 1996) Boydak, M. (1996). Ecology and silviculture of cedar of lebanon (Cedrus libani A. Rich.) and conservation of its natural forests. https://agris.fao.org/search/en/providers/122621/records/647396b0ce9437aa7600461f	High
34	6.07	How many time units (days, months, years) does the taxon require to reach the age at first reproduction?	>10	They start bearing fruit after 25 years (Messinger et al, 2015), Messinger, J., Güney, A., Zimmermann, R. et al. Cedrus libani: A promising tree species for Central European forestry facing climate change?. Eur J Forest Res 134, 1005–1017 (2015). https://doi.org/10.1007/s10342-015-0905-z It is rejuvenated by shelterwood silvicultural system with a production period of 120 to 140 years. from: Višnjić, Ć., Čilaš, M., & Ivojević, S. (2022). Ecological and silvicultural characteristics and the possibility of introducing Lebanese cedar (Cedrus libani A. Rich.) in BiH facing climate change. Naše šume, 66-67, pp. 53-67.	Very high
7. Dispersal mechanisms					
35	7.01	How many potential vectors or pathways could the taxon use to disperse within the risk assessment area (with suitable habitats nearby)?	One	seeds have good germination, sporadic natural regeneration from seeds was observed next to adult trees in Germany recently - 2012 (Messinger et al, 2015)	High
36	7.02	Will any of these vectors or pathways bring the taxon in close proximity to one or more protected areas?	No	Status of C. libani: Two of these cedars – the Atlas and the Lebanon – run the risk of extinction in the wild. Centuries of over-harvesting, over-grazing of regenerating saplings and wild fires have taken their toll on stocks in their native mountainous terrain. Of the vast cedar forests that once clothed most of Lebanon's highlands, only roughly 17 km² of untouched climax stands remain, in scattered groves. https://www.forestryjournal.co.uk/features/forestry-journal-	High
37	7.03	Does the taxon have a specialised means of attachment such that it enhances the likelihood of dispersal?	No	i din't find such info in the literature.	Medium
38	7.04	Is natural dispersal of the taxon likely to occur as seeds or spores in the risk assessment area?	No	- Further research is needed to determine whether it can be transmitted by anemochore and zoochore vectors and what conditions are required for successful germination (Messinger et al, 2015) - -as C. libani has large seeds, it is not expected to spread long distances by wind.	Medium
39	7.05	Is natural dispersal of the taxon likely to occur as fragments or seedlings in the risk assessment area?	No	no, it is possible naturally only by seeds.	High
40	7.06	Are any life stages of the taxon likely to migrate into the risk assessment area for reproduction?	No	no, i didn't find such information.	Medium
41	7.07	Are propagules of the taxon likely to be dispersed in the risk assessment area by other species?	No	it is not known.	Low
42	7.08	Is dispersal of the taxon along any of the vectors or pathways mentioned in the previous seven Questions (35–41: i.e. either unintentional or intentional) likely to be	No	the species has slow growth, and it takes more than 25 years to reach sexual maturity, by some authors even 40 years. (Messinger et al, 2015).	High
43	7.09	Is dispersal of the taxon density dependent?	No	i really dont know	Low
8. Tolerance attributes					
44	8.01	Is the taxon able to withstand being in water for extended periods at some stage of its life cycle?	No	https://www.waldwissen.net/en/forestry/silviculture/plant-cultivation/the-lebanon-cedar-in-climate-change-experiences-from-turkey	Low
45	8.02	Is the taxon tolerant of a wide range of soil or air quality conditions?	Yes	Onder, S., & Dursun, S. (2006). Air borne heavy metal pollution of Cedrus libani (A. Rich.) in the city centre of Konya (Turkey). Atmospheric Environment, 40(6), 1122-1133. The contents of heavy metal accumulated by air pollution in the cedar tree needles from the green area of Konya city centre were measured by chemical analyses. The needle samples were collected from two types of trees (about 10–15 and 20–25-years-old trees) from eight different pollution regions for two different sampling periods. Results of the present study showed that accumulations of heavy metals in the old trees were generally higher than those of young trees. Similarly, heavy metal contents of needles collected in spring 2004 were higher than those of needles collected in autumn 2003. Accumulation of heavy metals via sulphur dioxide pollution and particle matter originated from usage of low quality fossil fuels, which might affect the living organisms in the city centre. On the other hand, Pb levels in the samples from Karatay Industry Park (3.53 ppm in 2004) showed that people are health living around the industry, and heavy traffic area is under risk. According to the other sampling areas, the chromium levels were also very high around the Chrome–Magnesite Factory Garden (87.15 ppm in 2004); it could be a toxic risk for people working around the factory. The heavy metal levels obtained from the	Medium
46	8.03	Can the taxon be controlled or eradicated in the wild with chemical, biological or other agents and means?	Not applicable	i dont have data about	Medium

47	8.04	Is the taxon likely to benefit from environmental or human disturbance?	Yes	yes, from the forest fires: in the first years of cultivation up to 10,000 plants per ha, a controlled fire is carried out, after that it can be up to 50,000 plants/ha (Boydak, 1996) -) Boydak, M. (1996). Ecology and silviculture of cedar of lebanon (Cedrus libani A. Rich.) and conservation of its natural forests.https://agris.fao.org/search/en/providers/122621/records/647396b0ce9437aa7600461f	Medium
48	8.05	Is the taxon able to tolerate soil acidity, salinity or other parameter levels that are higher or lower than those found in its usual	No	It is a light-demanding, calciphile coniferous species that is most vigorous when planted in montane regions.	Low
49	8.06	Are there effective natural enemies of the taxon present in the risk assessment area?	Yes	Major C. libani pests: 1. Lymantria dispar 2. Cephalcia tannourinensis 3. Dichelia cedricola 4. Thaumetopoea libanotica 3. M2. The Cedar Web-spinning Sawfly - This major pest of C. libani, causes needle browning, defoliation and tree decline. Heavily infested forests turn brown, looking as if burnt. In Lebanon the rate of attack came to 70-80%. Such heavy infestations may cause the death of cedars. chrome-extension://efaidnbmnnnibpcajpcglclefindmkaj/https://danyelobeid.wordpress.com/wp-content/uploads/2016/06/common-pest-and-diseases-on-cedrus-libani-forest.pdf Pests insects of cedars: Cedrus atlantica Manetti, C. libani A. Richard and C. brevifolia Henry in the Mediterranean area. In Entomological Research in Mediterranean Forest Ecosystems, INRA Editions, Science Update Series, INRA, Paris: 89-104 January 2005 Mohamed MounaMohamed Mouna https://www.researchgate.net/publication/263806872_Pests_insects_of_cedars_Cedrus_atlantica_Manetti_C_libani_A_Richard_and_C_brevifolia_Henry_in_the_Mediterranean_area_In_Entomological	Medium
**C. Climate change**					
9. Climate change					
50	9.01	Under the predicted future climatic conditions, are the risks of entry into the risk assessment area posed by the taxon likely to increase, decrease or not change?	Decrease	Distribution of Cedrus would be reduced in response to global climate change in the future. from: Xiao, S., Li, S., Wang, X., Chen, L., & Su, T. (2022). Cedrus distribution change: past, present, and future. Ecological Indicators, 142, 109159. Status of C. libani: Two of these cedars – the Atlas and the Lebanon – run the risk of extinction in the wild. Centuries of over-harvesting, over-grazing of regenerating saplings and wild fires have taken their toll on stocks in their native mountainous terrain. Of the vast cedar forests that once clothed most of Lebanon's highlands, only roughly 17 km² of untouched climax stands remain, in scattered groves. https://www.forestryjournal.co.uk/features/forestry-journal-features/24231782.cedrus-libani-future-hold-lebanons-	High
51	9.02	Under the predicted future climatic conditions, are the risks of establishment within the risk assessment area posed by the taxon likely to increase, decrease or not change?	Decrease	Xiao, S., Li, S., Wang, X., Chen, L., & Su, T. (2022). Cedrus distribution change: past, present, and future. Ecological Indicators, 142, 109159. In general, this study identifies the key climate factors restricting the natural distribution of Cedrus. It shows that the distribution of Cedrus would be reduced in response to global climate change in the future, which indicates an urgent need for the protection and management of Cedrus	Medium
52	9.03	Under the predicted future climatic conditions, are the risks of dispersal within the risk assessment area posed by the taxon likely to increase, decrease or not change?	Decrease	Xiao, S., Li, S., Wang, X., Chen, L., & Su, T. (2022). Cedrus distribution change: past, present, and future. Ecological Indicators, 142, 109159. In general, this study identifies the key climate factors restricting the natural distribution of Cedrus. It shows that the distribution of Cedrus would be reduced in response to global climate change in the future, which indicates an urgent need for the protection and management of Cedrus populations. Highlights Winter precipitation and temperature are critical for Cedrus Climate change can significantly affect the distribution of Cedrus Distribution of Cedrus would be reduced in response to global climate change in the future. 5. Conclusions In this study we used the MaxEnt model, in combination with climate data and the current distribution data of Cedrus to simulate the past, present and future distribution of potentially suitable habitats for Cedrus in the Mediterranean region and western Himalaya. Our results indicate that winter precipitation is the key factor constraining the spatial extent of the Cedrus distribution, while winter temperature is also a relatively important factor. In the Mediterranean region, summer precipitation has an important bearing on the distribution of Cedrus. Cedrus was more widely distributed during the LGM than the present. The distribution of Cedrus remained relatively stable from the MH to the present. In the future, a low representative concentration pathway will be beneficial to the survival of Cedrus. In the western Himalaya, Cedrus was most widely distributed in the MH, while the spatial extent of the future distribution will be significantly reduced in [illegible]	High
53	9.04	Under the predicted future climatic conditions, what is the likely magnitude of future potential impacts by the taxon on biodiversity or ecosystem integrity and	Not applicable	In the future, we suggest priority be given to the protection of Cedrus in the Mediterranean regionXiao, S., Li, S., Wang, X., Chen, L., & Su, T. (2022). Cedrus distribution change: past, present, and future. Ecological Indicators, 142, 109159.	High

54	9.05	Under the predicted future climatic conditions, what is the likely magnitude of future potential impacts by the taxon on ecosystem structure or function?	No change	Xiao, S., Li, S., Wang, X., Chen, L., & Su, T. (2022). Cedrus distribution change: past, present, and future. Ecological Indicators, 142, 109159. In general, this study identifies the key climate factors restricting the natural distribution of Cedrus. It shows that the distribution of Cedrus would be reduced in response to global climate change in the future, which indicates an urgent need for the protection and management of Cedrus populations. Highlights Winter precipitation and temperature are critical for Cedrus Climate change can significantly affect the distribution of Cedrus Distribution of Cedrus would be reduced in response to global climate change in the future. 5. Conclusions	High
55	9.06	Under the predicted future climatic conditions, what is the likely magnitude of future potential impacts by the taxon on ecosystem services and related socio-economic factors?	No change	Xiao, S., Li, S., Wang, X., Chen, L., & Su, T. (2022). Cedrus distribution change: past, present, and future. Ecological Indicators, 142, 109159. In general, this study identifies the key climate factors restricting the natural distribution of Cedrus. It shows that the distribution of Cedrus would be reduced in response to global climate change in the future, which indicates an urgent need for the protection and management of Cedrus populations. Highlights Winter precipitation and temperature are critical for Cedrus Climate change can significantly affect the distribution of Cedrus Distribution of Cedrus would be reduced in response to global climate change in the future. 5. Conclusions	High

Statistics	
**Scores**	
**BRA**	**1.0**
**BRA Outcome**	**Medium**
**BRA+CCA**	**-5.0**
**BRA+CCA Outcome**	**Low**
**Score partition**	
**A. Biogeography/Historical**	**3.0**
1. Domestication/Cultivation	*2.0*
2. Climate, distribution and introduction risk	*1.0*
3. Invasive elsewhere	*0.0*
**B. Biology/Ecology**	**-2.0**
4. Undesirable (or persistence) traits	*1.0*
5. Resource exploitation	*0.0*
6. Reproduction	*1.0*
7. Dispersal mechanisms	*-5.0*
8. Tolerance attributes	*1.0*
**C. Climate change**	**-6.0**
9. Climate change	*-6.0*
**Answered Questions**	
Total	***55***
**A. Biogeography/Historical**	**13**
1. Domestication/Cultivation	*3*
2. Climate, distribution and introduction risk	*5*
3. Invasive elsewhere	*5*
**B. Biology/Ecology**	**36**
4. Undesirable (or persistence) traits	*12*
5. Resource exploitation	*2*
6. Reproduction	*7*
7. Dispersal mechanisms	*9*
8. Tolerance attributes	*6*
**C. Climate change**	**6**
9. Climate change	*6*
**Sectors affected**	
**Commercial**	**4**
**Environmental**	**1**
**Species or population nuisance traits**	**-9**

Thresholds	
**BRA**	**41**
**BRA+CCA**	**41**
**Confidence**	
**BRA+CCA**	**0.65**
**BRA**	**0.64**
**CCA**	**0.71**

Date and Time
**01/08/2024 16:09:47**

TPS-ISK v2

Taxon and Assessor details	
Category	Gymnosperms
Taxon name	*Chamaecyparis lawsoniana*
Common name	Lawson Cypress (Port Orford Cedar)
Assessor	Božena Mitić
**Risk screening context**	
Reason and socio-economic benefits	ornamental plant
Risk assessment area	Continental Europe
Taxonomy	fam. Cupressaceae
Native range	North America (Oregon and northwestern California)
Introduced range	across North America and all other continents
URL	

			Response	Justification (references or other information)	Confidence
**A. Biogeography/Historical**					
1. Domestication/Cultivation					
1	1.01	Has the taxon been the subject of domestication?	Yes	Chamaecyparis lawsoniana (A.Murray bis) Parl. in GBIF Secretariat (2023). GBIF Backbone Taxonomy. Checklist dataset https://doi.org/10.15468/39omei accessed via GBIF.org on 2024-08-01.; https://en.wikipedia.org/wiki/Chamaecyparis_lawsoniana; Idžojtić et al. (2010): Šumarski list 1–2, CXXXIV, 5-18.	Very high
2	1.02	Is the taxon harvested in the wild and likely to be sold or used in its live form?	Yes	eg. https://club.global.flowers/en/chamaecyparis/description; https://www.vdberk.com/trees/chamaecyparis-lawsoniana/	Very high
3	1.03	Does the taxon have invasive races, varieties, sub-taxa or congeners?	Yes	https://www.invasive.org/browse/subinfo.cfm?sub=3210	High
2. Climate, distribution and introduction risk					
4	2.01	How similar are the climatic conditions between the risk assessment area and the taxon's native range?	Medium	Peel et al. 2007	High
5	2.02	What is the quality of the climate matching data?	Medium	Peel et al. 2007	Medium
6	2.03	Is the taxon known to be present outside of cultivation in the risk assessment area?	Yes	Chamaecyparis lawsoniana (A.Murray bis) Parl. in GBIF Secretariat (2023). GBIF Backbone Taxonomy. Checklist dataset https://doi.org/10.15468/39omei accessed via GBIF.org on 2024-08-01.; https://alienplantsbelgium.myspecies.info/content/chamaecyparis-	Very high
7	2.04	How many potential vectors could the taxon use to enter the risk assessment area?	>1	Chamaecyparis lawsoniana (A.Murray bis) Parl. in GBIF Secretariat (2023). GBIF Backbone Taxonomy. Checklist dataset https://doi.org/10.15468/39omei accessed via GBIF.org on 2024-08-01.; Idžojtić M. (2013): DENDROLOGIJA – cvijet, češer, plod, sjeme. Denona d.d. (In Croatian).	Very high
8	2.05	Is the taxon currently found in close proximity to, and likely to enter, the risk assessment area in the near future (e.g. unintentional or intentional introductions)?	Yes	Chamaecyparis lawsoniana (A.Murray bis) Parl. in GBIF Secretariat (2023). GBIF Backbone Taxonomy. Checklist dataset https://doi.org/10.15468/39omei accessed via GBIF.org on 2024-08-01.	High
3. Invasive elsewhere					
9	3.01	Has the taxon become naturalised outside its native range?	No	CASUAL (Chamaecyparis lawsoniana (A.Murray bis) Parl. in GBIF Secretariat (2023). GBIF Backbone Taxonomy. Checklist dataset https://doi.org/10.15468/39omei accessed via GBIF.org on 2024-08-01.)	High
10	3.02	In the taxon's introduced range, are there any known adverse impacts to wild or commercial species?	No	No exact references found (professional judgement)	Medium
11	3.03	In the taxon's introduced range, are there any known adverse impacts to agriculture or forestry?	No	No exact references found (professional judgement)	Medium
12	3.04	In the taxon's introduced range, are there any known adverse impacts to ecosystem	No	No exact references found (professional judgement)	High
13	3.05	In the taxon's introduced range, are there any known adverse socio-economic impacts?	No	No exact references found (professional judgement)	Medium
**B. Biology/Ecology**					
4. Undesirable (or persistence) traits					
14	4.01	Is the taxon likely to be poisonous or pose other risks to human health?	Yes	Allergenic pollen - eg. Židovec et al. (2023): JCEA 24(1): 245-259. However, also non-toxic for humans and animals, see eg. https://greg.app/lawson-cypress-toxic-to-dogs/	High
15	4.02	Is the taxon likely to suppress the growth of one or more native species?	No	No exact references found (professional judgement)	Medium
16	4.03	Are there any threatened or protected native species that the taxon would parasitise in the risk assessment area?	No	No exact references found (professional judgement)	Medium
17	4.04	Is the taxon adaptable in terms of climatic and other environmental conditions, thus enhancing its potential persistence if it has invaded or is likely to invade the risk	Yes	https://en.wikipedia.org/wiki/Chamaecyparis_lawsoniana; https://alienplantsbelgium.myspecies.info/content/chamaecyparis-lawsoniana	High
18	4.05	Is the taxon likely to disrupt terrestrial ecosystem function if it has invaded or is likely to invade the risk assessment area?	No	https://alienplantsbelgium.myspecies.info/content/chamaecyparis-lawsoniana	Medium
19	4.06	Is the taxon likely to exert adverse impacts on ecosystem services in the risk	No	No exact references found (professional judgement)	Medium
20	4.07	Is the taxon likely to host or function as a vector for recognised pests and pathogens that are present in the risk assessment area?	Yes	invasive pathogen Phytophthora lateralis: Robin et al. (2011): Forest Pathology 41(5): 417-424. https://doi.org/10.1111/j.1439-0329.2010.00688.x; Green et al. (2013): Forest Pathology 42(1): 19-28. https://doi.org/10.1111/j.1439-0329.2012.00788.x	Very high

21	4.08	Is the taxon likely to host or function as a vector for recognised pests and pathogens that are absent in the risk assessment area?	No	No exact references found (professional judgement)	Medium
22	4.09	Is the taxon likely to be released from cultivation?	Yes	https://alienplantsbelgium.myspecies.info/content/chamaecyparis-lawsoniana;	High
23	4.10	Is the taxon versatile in habitat use?	Yes	https://en.wikipedia.org/wiki/Chamaecyparis_lawsoniana; https://alienplantsbelgium.myspecies.info/content/chamaecyparis-lawsoniana	High
24	4.11	Is it likely that the taxon's mode of existence or behaviours will reduce habitat quality for native species?	No	No exact references found (professional judgement)	High
25	4.12	Is the taxon likely to maintain a viable population even when present in low densities (or persist in adverse conditions by way of a dormant form)?	Yes	Professional judgement based on https://alienplantsbelgium.myspecies.info/content/chamaecyparis-lawsoniana	Medium
5. Resource exploitation					
26	5.01	Is the taxon likely to put threatened or protected native species under competitive pressure in the risk assessment area?	Yes	No exact references found (professional judgement)	Medium
27	5.02	Is the taxon likely to sequester resources to the detriment of native species in the risk assessment area?	No	No exact references found (professional judgement)	Medium
6. Reproduction					
28	6.01	Is the taxon likely to exhibit changes in reproductive strategy in response to environmental conditions?	No	No exact references found (professional judgement)	Medium
29	6.02	Is the taxon likely to produce viable propagules in the risk assessment area?	Yes	https://alienplantsbelgium.myspecies.info/content/chamaecyparis-lawsoniana	High
30	6.03	Is the taxon likely to hybridise with native species under natural conditions?	No	No exact references found (professional judgement)	Medium
31	6.04	Is the taxon likely to be hermaphroditic or to exhibit asexual reproduction?	No	Idžojtić M. (2013): DENDROLOGIJA – cvijet, češer, plod, sjeme. Denona d.d. (In Croatian).	Very high
32	6.05	Is the taxon dependent on the presence of another species (or specific habitat features) to complete its life cycle?	No	Idžojtić M. (2013): DENDROLOGIJA – cvijet, češer, plod, sjeme. Denona d.d. (In Croatian).	Very high
33	6.06	Is the taxon likely to produce a large number of propagules?	Yes	Idžojtić M. (2013): DENDROLOGIJA – cvijet, češer, plod, sjeme. Denona d.d. (In Croatian).	Very high
34	6.07	How many time units (days, months, years) does the taxon require to reach the age at first reproduction?	>10	Idžojtić M. (2013): DENDROLOGIJA – cvijet, češer, plod, sjeme. Denona d.d. (In Croatian).	Very high
7. Dispersal mechanisms					
35	7.01	How many potential vectors or pathways could the taxon use to disperse within the risk assessment area (with suitable habitats nearby)?	>1	Idžojtić M. (2013): DENDROLOGIJA – cvijet, češer, plod, sjeme. Denona d.d. (In Croatian).; https://alienplantsbelgium.myspecies.info/content/chamaecyparis-lawsoniana	Very high
36	7.02	Will any of these vectors or pathways bring the taxon in close proximity to one or more protected areas?	Yes	Idžojtić M. (2013): DENDROLOGIJA – cvijet, češer, plod, sjeme. Denona d.d. (In Croatian).; Idžojtić et al. (2010): Šumarski list 1–2, CXXXIV, 5-18.	Very high
37	7.03	Does the taxon have a specialised means of attachment such that it enhances the likelihood of dispersal?	Yes	Idžojtić M. (2013): DENDROLOGIJA – cvijet, češer, plod, sjeme. Denona d.d. (In Croatian).	Very high
38	7.04	Is natural dispersal of the taxon likely to occur as seeds or spores in the risk	Yes	Idžojtić M. (2013): DENDROLOGIJA – cvijet, češer, plod, sjeme. Denona d.d. (In Croatian).	Very high
39	7.05	Is natural dispersal of the taxon likely to occur as fragments or seedlings in the risk assessment area?	Yes	Idžojtić M. (2013): DENDROLOGIJA – cvijet, češer, plod, sjeme. Denona d.d. (In Croatian).	Medium
40	7.06	Are any life stages of the taxon likely to migrate into the risk assessment area for reproduction?	Yes	Idžojtić M. (2013): DENDROLOGIJA – cvijet, češer, plod, sjeme. Denona d.d. (In Croatian).; Idžojtić et al. (2010): Šumarski list 1–2, CXXXIV, 5-18.	Medium
41	7.07	Are propagules of the taxon likely to be dispersed in the risk assessment area by other species?	No	No exact references found (professional judgement)	Low
42	7.08	Is dispersal of the taxon along any of the vectors or pathways mentioned in the previous seven Questions (35–41: i.e. either unintentional or intentional) likely to be	No	No exact references found (professional judgement)	Medium
43	7.09	Is dispersal of the taxon density dependent?	No	No exact references found (professional judgement)	Low
8. Tolerance attributes					
44	8.01	Is the taxon able to withstand being in water for extended periods at some stage of its life cycle?	Yes	https://en.wikipedia.org/wiki/Chamaecyparis_lawsoniana	Low
45	8.02	Is the taxon tolerant of a wide range of soil or air quality conditions?	Yes	https://en.wikipedia.org/wiki/Chamaecyparis_lawsoniana; https://threatenedconifers.rbge.org.uk/conifers/chamecyparis-lawsoniana	Medium
46	8.03	Can the taxon be controlled or eradicated in the wild with chemical, biological or other agents and means?	Yes	C. pisifera: https://edis.ifas.ufl.edu/publication/ST158	High
47	8.04	Is the taxon likely to benefit from environmental or human disturbance?	No	No exact references found (professional judgement)	Medium
48	8.05	Is the taxon able to tolerate soil acidity, salinity or other parameter levels that are higher or lower than those found in its usual	Yes	https://en.wikipedia.org/wiki/Chamaecyparis_lawsoniana	High

49	8.06	Are there effective natural enemies of the taxon present in the risk assessment area?	Yes	Pathogen Phytophthora lateralis. However, this pathogen is also invasive: Robin et al. (2011): Forest Pathology 41(5): 417-424. https://doi.org/10.1111/j.1439-0329.2010.00688.x Green et al. (2013): Forest Pathology 42(1): 19-28. https://doi.org/10.1111/j.1439-0329.2012.00788.x	Very high
**C. Climate change**					
9. Climate change					
50	9.01	Under the predicted future climatic conditions, are the risks of entry into the risk assessment area posed by the taxon likely to increase, decrease or not change?	Increase	Professional judgement	Medium
51	9.02	Under the predicted future climatic conditions, are the risks of establishment within the risk assessment area posed by the taxon likely to increase, decrease or not	Increase	Professional judgement	Low
52	9.03	Under the predicted future climatic conditions, are the risks of dispersal within the risk assessment area posed by the taxon likely to increase, decrease or not change?	Increase	Professional judgement	High
53	9.04	Under the predicted future climatic conditions, what is the likely magnitude of future potential impacts by the taxon on biodiversity or ecosystem integrity and	No change	Professional judgement	Low
54	9.05	Under the predicted future climatic conditions, what is the likely magnitude of future potential impacts by the taxon on ecosystem structure or function?	No change	Professional judgement	Low
55	9.06	Under the predicted future climatic conditions, what is the likely magnitude of future potential impacts by the taxon on ecosystem services and related socio-	No change	Professional judgement	Low

**Statistics**	
**Scores**	
**BRA**	**23.0**
**BRA Outcome**	**Medium**
**BRA+CCA**	**29.0**
**BRA+CCA Outcome**	**Medium**
**Score partition**	
**A. Biogeography/Historical**	**6.0**
1. Domestication/Cultivation	*4.0*
2. Climate, distribution and introduction risk	*4.0*
3. Invasive elsewhere	*-2.0*
**B. Biology/Ecology**	**17.0**
4. Undesirable (or persistence) traits	*6.0*
5. Resource exploitation	*5.0*
6. Reproduction	*0.0*
7. Dispersal mechanisms	*5.0*
8. Tolerance attributes	*1.0*
**C. Climate change**	**6.0**
9. Climate change	*6.0*
**Answered Questions**	
Total	***55***
**A. Biogeography/Historical**	**13**
1. Domestication/Cultivation	*3*
2. Climate, distribution and introduction risk	*5*
3. Invasive elsewhere	*5*
**B. Biology/Ecology**	**36**
4. Undesirable (or persistence) traits	*12*
5. Resource exploitation	*2*
6. Reproduction	*7*
7. Dispersal mechanisms	*9*
8. Tolerance attributes	*6*
**C. Climate change**	**6**
9. Climate change	*6*
**Sectors affected**	
**Commercial**	**8**
**Environmental**	**6**
**Species or population nuisance traits**	**21**

**Thresholds**	
**BRA**	**41**
**BRA+CCA**	**41**
**Confidence**	
**BRA+CCA**	**0.66**
**BRA**	**0.69**
**CCA**	**0.38**

**Date and Time**
**13/08/2024 15:19:56**

TPS-ISK v2

Taxon and Assessor details	
Category	Gymnosperms
Taxon name	*Ginkgo biloba*
Common name	Ginkgo
Assessor	Seokmin Kim
**Risk screening context**	
Reason and socio-economic benefits	Often planted in urban streets and cultivated for consumption
Risk assessment area	Continental Europe
Taxonomy	Family: Ginkgoaceae, Genus: Ginkgo
Native range	South east and South central China
Introduced range	Norh America, Europe (Continental + Britain), East Asia (Japan / Korea), Australia, New Zealand,
URL	https://edis.ifas.ufl.edu/publication/ST273

			Response	Justification (references or other information)	Confidence
**A. Biogeography/Historical**					
1. Domestication/Cultivation					
1	1.01	Has the taxon been the subject of domestication?	Yes	1. Ginkgo has been cultivated in China for several thousand years 2. Survival until present attributed to cultivation. However, unknown if plant has been subjected to intentional human selection. Answered YES with LOW confidence. https://www.ncbi.nlm.nih.gov/pmc/articles/PMC10410194/ 1: https://www.researchgate.net/profile/Peter-Del-Tredici/publication/284761587_The_evolution_ecology_and_cultivation_of_Ginkgo_biloba/links/597a42230f7e9b0469b22d9d/The-evolution-ecology-and-cultivation-of-Ginkgo-biloba.pdf 2.	Low
2	1.02	Is the taxon harvested in the wild and likely to be sold or used in its live form?	Yes	1. 2. Seeds sold online in Europe. 1: https://www.seeds-gallery.eu/en/home/maidenhair-tree-seeds-ginkgo-biloba.html 2: https://www.growmart.eu/Plant-Seeds-Ginkgo 1: https://www.seeds-gallery.eu/en/home/maidenhair-tree-seeds-ginkgo-biloba.html 2: https://www.growmart.eu/Plant-Seeds-	Very high
3	1.03	Does the taxon have invasive races, varieties, sub-taxa or congeners?	No	1. Plant is the only species in the genus. 2. Only surviving member of the order. 1: https://sites.redlands.edu/trees/species-accounts/ginkgo/ 2: https://www.gbif.org/species/113553317 1: https://sites.redlands.edu/trees/species-accounts/ginkgo/ 2: https://www.gbif.org/species/113553317	Very high
2. Climate, distribution and introduction risk					
4	2.01	How similar are the climatic conditions between the risk assessment area and the taxon's native range?	High	1. Plant native to south-central and southeast China, 2. where climate is subtropical and tropical. 3. Parts in Europe that have this climate are along the Meditteranean. 4. However, plants are found in other parts of Europe as well. 1. https://plants.ces.ncsu.edu/plants/ginkgo-biloba/#:~:text=It%20is%20native%20to%20south,and%20good%20drainage%20to%20thrive. 2. https://www.metoffice.gov.uk/weather/travel/holiday-weather/asia/china/china-weather 3. https://www.britannica.com/place/Europe/Climate 4.	Medium
5	2.02	What is the quality of the climate matching data?	Medium	Did not conduct a full climate matching process as it is difficult to pinpoint where the native range overlays with gbif observations.	Medium
6	2.03	Is the taxon known to be present outside of cultivation in the risk assessment area?	Yes	1. 2. Ginkgo trees are planted commonly on streets, therefore outside of confined areas. 1. https://www.uib.no/en/universitygardens/146973/ginkgo-biloba 2. https://www.vdberk.com/trees/ginkgo-biloba/	Very high
7	2.04	How many potential vectors could the taxon use to enter the risk assessment area?	One	1. 2. Tree predominantly thought to have been introduced through intentional human mediated means. Biological dispersal is possible (2), but not reported to have caused introductions. 1. https://pubs.cif-ifc.org/doi/pdf/10.5558/tfc68612-5 2. https://besjournals.onlinelibrary.wiley.com/doi/pdf/10.1111/1365-2745.13856	Medium
8	2.05	Is the taxon currently found in close proximity to, and likely to enter, the risk assessment area in the near future (e.g. unintentional or intentional introductions)?	Not applicable	1. 2. 3. Plant is already present in Europe. 1. https://www.uib.no/en/universitygardens/146973/ginkgo-biloba 2. https://www.vdberk.com/trees/ginkgo-biloba/ 3. https://www.gbif.org/species/2687885	Very high
3. Invasive elsewhere					
9	3.01	Has the taxon become naturalised outside its native range?	Yes	1. Noted to have never become naturalized outside of China 2. Noted to be able to produce and grow from seeds in Europe. However, unclear if this occurs outside of human intervention. 3. Noted to be rarely naturalized in Europe as it is typically cultivated. Suggests that naturalization CAN occur. 1. https://www.vmnh.net/article/it-s-time-for-another-beninnature-update/11-18-2020 2. https://www.uib.no/en/universitygardens/146973/ginkgo-biloba 3. https://blog.irontreeservice.com/tree-profiles-ginkgo-part-1-	Low
10	3.02	In the taxon's introduced range, are there any known adverse impacts to wild or commercial species?	No	No reports of adverse effects of the plant on wild or commercial species found. Given lack of evidence and reporting, and the well-studied nature of this plant, answered NO with MODERATE confidence.	Medium
11	3.03	In the taxon's introduced range, are there any known adverse impacts to agriculture or forestry?	No	No reports of adverse effects of the plant on agriculture or forestry found. Given lack of evidence and reporting, answered NO with MODERATE confidence.	Medium
12	3.04	In the taxon's introduced range, are there any known adverse impacts to ecosystem services?	No	No reports of adverse effects of the plant on ecosystem services found. Given lack of evidence and reporting, answered NO with MODERATE confidence.	Medium

13	3.05	In the taxon's introduced range, are there any known adverse socio-economic impacts?	Yes	1. Fruit noted to be unwanted due to foul smells. 2. Control measures documented to prevent fruiting of plants. Answered Yes with MODERATE confidence since I could not find instances of the plant causing adverse effects in Europe. Not LOW confidence as foul smell is likely to cause issues anywhere. 1. https://www.uib.no/en/universitygardens/146973/ginkgo-biloba 2. https://ddot.dc.gov/release/ddot-begin-spraying-ginkgo-trees-overnight-0a	Medium
**B. Biology/Ecology**					
4. Undesirable (or persistence) traits					
14	4.01	Is the taxon likely to be poisonous or pose other risks to human health?	Yes	1. Plant commonly consumed. Known to cause some harm if seeds are consumed by causing seizures. 2. Leaf may contain carcinogenic extracts. However, these will likely not pose direct harm to people in its wild state. Answered YES with LOW confidence. 1. https://www.mayoclinic.org/drugs-supplements-ginkgo/art-20362032#:~:text=Allergic%20skin%20reactions-,Don't%20eat%20raw%20or%20roasted%20ginkgo%20seeds%2C%20which%20can,prone%20to%20seizures%2C%20avoid%20ginkgo. 2. https://www.ncbi.nlm.nih.gov/pmc/articles/PMC6373469/	Low
15	4.02	Is the taxon likely to suppress the growth of one or more native species?	Yes	1. 2. Plant contains allelopathic compounds. However, no report found of plant suppressing growth of native species in the wild in Europe. 1. https://www.sciencedirect.com/science/article/pii/S017616171300	Low
16	4.03	Are there any threatened or protected native species that the taxon would parasitise in the risk assessment area?	Yes	No evidence plant functions as a parasite	High
17	4.04	Is the taxon adaptable in terms of climatic and other environmental conditions, thus enhancing its potential persistence if it has invaded or is likely to invade the risk	Yes	1. Plant grows well in USDA plant hardiness zones 4 to 9. 2. Found throughout Europe 1. https://www.masterclass.com/articles/ginkgo-tree-care-tips= 2. https://www.gbif.org/species/2687885	High
18	4.05	Is the taxon likely to disrupt terrestrial ecosystem function if it has invaded or is likely to invade the risk assessment area?	No	No reports of plant disrupting terrestrial ecosystem function. 1. Noted as not being invasive. Given lack of evidence, answered NO with MODERATE confidence 1. https://greg.app/ginkgo-biloba-weed/	Medium
19	4.06	Is the taxon likely to exert adverse impacts on ecosystem services in the risk	No	No reports of plant exerting adverse impacts on ecosystem services in the risk assessment area	Medium
20	4.07	Is the taxon likely to host or function as a vector for recognised pests and pathogens that are present in the risk assessment area?	No	1. 2. Ginkgo trees are generally resistent to pest or insects. No reports of ginkgos significant carrying pathogens or pests 1. https://sites.redlands.edu/trees/species-accounts/ginkgo/ginkgoinsects/ 2. https://edis.ifas.ufl.edu/publication/ST273	Medium
21	4.08	Is the taxon likely to host or function as a vector for recognised pests and pathogens that are absent in the risk assessment area?	No	1. 2. Ginkgo trees are generally resistent to pest or insects. No reports of ginkgos significant carrying pathogens or pests 1. https://sites.redlands.edu/trees/species-accounts/ginkgo/ginkgoinsects/ 2. https://edis.ifas.ufl.edu/publication/ST273	Medium
22	4.09	Is the taxon likely to be released from cultivation?	Yes	1. 2. Ginkgo grows outside, but grows slowly. 1. https://edis.ifas.ufl.edu/publication/ST273 2. https://besjournals.onlinelibrary.wiley.com/doi/pdf/10.1111/1365-2745.13856	Low
23	4.10	Is the taxon versatile in habitat use?	Yes	1. Noted to tolerate most soil types. 2. Noted to be able to tolerate high levels of pollution, making it able to inhabit urban areas. 1. https://edis.ifas.ufl.edu/publication/ST273 2. https://besjournals.onlinelibrary.wiley.com/doi/pdf/10.1111/1365-2745.13856	Medium
24	4.11	Is it likely that the taxon's mode of existence or behaviours will reduce habitat quality for native species?	No	1. Noted to have some palatability to browsing animals. 2. Fruits and nuts eaten by a variety of animals. 1. https://plants.usda.gov/home/plantProfile?symbol=GIBI2 2. https://tylerarboretum.org/wild-fruits-and-nuts/	Medium
25	4.12	Is the taxon likely to maintain a viable population even when present in low densities (or persist in adverse conditions by way of a dormant form)?	No	No reports of established populations of the taxon persisting at low densities. However, it is important to note that these plants are long-living. Therefore, populations could persist for long periods of time.	Medium
5. Resource exploitation					
26	5.01	Is the taxon likely to put threatened or protected native species under competitive pressure in the risk assessment area?	No	No evidence that plant is likely to threaten or protected native species.	High
27	5.02	Is the taxon likely to sequester resources to the detriment of native species in the risk assessment area?	No	No evidence plant is likely to sequester large amount of resources. 1. However, is noted as a shade plant, and could limit light access in high densities. RIP value not calculated. 1. https://www.thespruce.com/ginkgo-biloba-trees-fall-foliage-champ-4119900	Medium
6. Reproduction					
28	6.01	Is the taxon likely to exhibit changes in reproductive strategy in response to environmental conditions?	No	No evidence plant exhibits changes in reproductive strategy.	Medium
29	6.02	Is the taxon likely to produce viable propagules in the risk assessment area?	Yes	1. Noted to produces seeds that are able to germinate in Europe. 2. Seeds able to be harvested and grown in general. 1. https://www.uib.no/en/universitygardens/146973/ginkgo-biloba 2. https://espacepourlavie.ca/en/growing-ginkgo-seed	High
30	6.03	Is the taxon likely to hybridise with native species under natural conditions?	No	As the only species in the order, unlikely to be able to hybridize with any native species	Medium

31	6.04	Is the taxon likely to be hermaphroditic or to exhibit asexual reproduction?	No	1. 2. Plant noted to be dioecious 1. https://besjournals.onlinelibrary.wiley.com/doi/pdf/10.1111/1365-2745.13856 2. https://www.missouribotanicalgarden.org/PlantFinder/PlantFinderDetails.aspx?kempercode=z990	Medium
32	6.05	Is the taxon dependent on the presence of another species (or specific habitat features) to complete its life cycle?	No	No evidence plant has specialized pollinators or relies on symbiosis	High
33	6.06	Is the taxon likely to produce a large number of propagules?	No	1. Plant is a gymnosperm. Therefore each "fruit" is a seed with multiple layers. Difficult to find information on how many fruits a mature plant can produce, but from available images, it is unlikely it produces prolific amount of seeds/fruits. 1. https://engagement.virginia.edu/learn/2020/12/04/from-green-to-gold-the-fall-transformation-of-ginkgoes	Medium
34	6.07	How many time units (days, months, years) does the taxon require to reach the age at first reproduction?	>10	1. 2. Noted to take as long as 20 years or more for the plant to fruit. 1. https://edis.ifas.ufl.edu/publication/ST273 2. https://engagement.virginia.edu/learn/2020/12/04/from-green-to-gold-the-fall-transformation-of-ginkgoes	Medium
7. Dispersal mechanisms					
35	7.01	How many potential vectors or pathways could the taxon use to disperse within the risk assessment area (with suitable habitats nearby)?	One	1. 2. Tree predominantly thought to have been introduced through intentional human mediated means. Biological dispersal is possible (2), but not reported to have caused introductions. 3. No instance found of plant being introduced to an area unintentionally. 1. https://pubs.cif-ifc.org/doi/pdf/10.5558/tfc68612-5 2. https://besjournals.onlinelibrary.wiley.com/doi/pdf/10.1111/1365-2745.13856 3. https://www.cabidigitallibrary.org/doi/10.1079/cabicompendium.2	Medium
36	7.02	Will any of these vectors or pathways bring the taxon in close proximity to one or more protected areas?	No	No evidence that plant is grown near protected areas, as it is primarily grown in urban areas.	Medium
37	7.03	Does the taxon have a specialised means of attachment such that it enhances the likelihood of dispersal?	No	Plant does not possess specialised means of attachment	High
38	7.04	Is natural dispersal of the taxon likely to occur as seeds or spores in the risk assessment area?	Yes	1. 2. Plant propagates through seed 1. https://besjournals.onlinelibrary.wiley.com/doi/pdf/10.1111/1365-2745.13856 2. https://www.researchgate.net/publication/284761587_The_evolution_ecology_and_cultivation_of_Ginkgo_biloba	High
39	7.05	Is natural dispersal of the taxon likely to occur as fragments or seedlings in the risk assessment area?	No	1. While plant is able to be propagate through seedlings, those have only been reported as artificial. 2. Naturally, plant propagates through seed. 1. https://www.thespruce.com/ginkgo-biloba-trees-fall-foliage-champ-4119900#toc-propagating-a-gingko-biloba-tree 2. https://www.researchgate.net/publication/284761587_The_evoluti	Medium
40	7.06	Are any life stages of the taxon likely to migrate into the risk assessment area for reproduction?	Yes	1. In Virginia, seedlings have been found growing far from parent trees. However, not evidence found in Europe. Possible, but as no records found in Europe, answered YES with LOW confidence 2. Squirrels identified as main dispersers in California. 1. https://engagement.virginia.edu/learn/2020/12/04/from-green-to-gold-the-fall-transformation-of-ginkgoes 2. https://ucmp.berkeley.edu/seedplants/ginkgoales/ginkgolh.html	Low
41	7.07	Are propagules of the taxon likely to be dispersed in the risk assessment area by other species?	Yes	1. In Virginia, seedlings have been found growing far from parent trees. However, not evidence found in Europe. Possible, but as no records found in Europe, answered YES with LOW confidence 2. Squirrels identified as main dispersers in California. 1. https://engagement.virginia.edu/learn/2020/12/04/from-green-to-gold-the-fall-transformation-of-ginkgoes 2. https://ucmp.berkeley.edu/seedplants/ginkgoales/ginkgolh.html	Low
42	7.08	Is dispersal of the taxon along any of the vectors or pathways mentioned in the previous seven Questions (35–41: i.e. either unintentional or intentional) likely to be	Yes	Intentional dispersal means may be rapid	Medium
43	7.09	Is dispersal of the taxon density dependent?	Yes	As plant is dioceious, male and female plants need to be in relatively close proximity for successful reproduction. 1. Plant pollinates primarily through wind. As no documented instance of taxon being density dependent is found, answered YES with LOW confidence 1. https://naturewalk.yale.edu/trees/ginkgoaceae/ginkgo-	Low
8. Tolerance attributes					
44	8.01	Is the taxon able to withstand being in water for extended periods at some stage of its life cycle?	No	No evidnece found regarding seed being submerged in water and surviving.	Low
45	8.02	Is the taxon tolerant of a wide range of soil or air quality conditions?	Yes	1. Noted to tolerate most soil types. 2. Noted to be able to tolerate high levels of pollution, making it able to inhabit urban	High
46	8.03	Can the taxon be controlled or eradicated in the wild with chemical, biological or other agents and means?	Yes	1. Herbicides noted to be able to harm plant 2. Herbicide used in Washington DC to prevent fruiting. 1. https://tfsweb.tamu.edu/uploadedFiles/TFS_Main/Urban_and_Community_Forestry/About_Urban_and_Community_Forestry/Urban_Forest_Information_Sheets/Tree%20Health%20-%20Abiotic%20Disorders_Herbicide%20Damage.pdf 2. https://ddot.dc.gov/release/ddot-begin-spraying-ginkgo-trees-overnight-0a	High

47	8.04	Is the taxon likely to benefit from environmental or human disturbance?	No	Plant not reported to benefit from disturbance. However 1. plant is known to be able to resist extreme disturbance events such as fires and even nuclear explosions. As plant is not shown to perform better after disturbance, answered NO with MODERATE confidence 1. https://besjournals.onlinelibrary.wiley.com/doi/10.1111/1365-	Medium
48	8.05	Is the taxon able to tolerate soil acidity, salinity or other parameter levels that are higher or lower than those found in its usual environment?	Yes	1. Noted to tolerate most soil types. 2. Noted to be able to tolerate high levels of pollution, making it able to inhabit urban areas. 1. https://edis.ifas.ufl.edu/publication/ST273 2. https://besjournals.onlinelibrary.wiley.com/doi/pdf/10.1111/1365-2745.13856	Medium
49	8.06	Are there effective natural enemies of the taxon present in the risk assessment area?	No	No effective natural enemies reported.	Low
**C. Climate change**					
9. Climate change					
50	9.01	Under the predicted future climatic conditions, are the risks of entry into the risk assessment area posed by the taxon likely to increase, decrease or not change?	No change	Entry is primarily intentional, and 1. plant is already present throughout continental Europe. 1. https://www.gbif.org/species/2687885	Medium
51	9.02	Under the predicted future climatic conditions, are the risks of establishment within the risk assessment area posed by the taxon likely to increase, decrease or not	No change	Entry is primarily intentional, and 1. plant is already present throughout continental Europe. 1. https://www.gbif.org/species/2687885	Medium
52	9.03	Under the predicted future climatic conditions, are the risks of dispersal within the risk assessment area posed by the taxon likely to increase, decrease or not change?	No change	Dispersal is primarily intentional, and 1. plant is already present throughout continental Europe. 1. https://www.gbif.org/species/2687885	Medium
53	9.04	Under the predicted future climatic conditions, what is the likely magnitude of future potential impacts by the taxon on biodiversity or ecosystem integrity and	No change	Impacts of plant appear low under any climate conditions.	Medium
54	9.05	Under the predicted future climatic conditions, what is the likely magnitude of future potential impacts by the taxon on ecosystem structure or function?	No change	Impacts of plant appear low under any climate conditions.	Medium
55	9.06	Under the predicted future climatic conditions, what is the likely magnitude of future potential impacts by the taxon on ecosystem services and related socio-	No change	Impacts of plant appear low under any climate conditions.	Medium

**Statistics**	
**Scores**	
**BRA**	**18.0**
**BRA Outcome**	**Medium**
**BRA+CCA**	**18.0**
**BRA+CCA Outcome**	**Medium**
**Score partition**	
**A. Biogeography/Historical**	**9.0**
1. Domestication/Cultivation	*2.0*
2. Climate, distribution and introduction risk	*1.0*
3. Invasive elsewhere	*6.0*
**B. Biology/Ecology**	**9.0**
4. Undesirable (or persistence) traits	*6.0*
5. Resource exploitation	*0.0*
6. Reproduction	*-1.0*
7. Dispersal mechanisms	*3.0*
8. Tolerance attributes	*1.0*
**C. Climate change**	**0.0**
9. Climate change	*0.0*
**Answered Questions**	
Total	***55***
**A. Biogeography/Historical**	**13**
1. Domestication/Cultivation	*3*
2. Climate, distribution and introduction risk	*5*
3. Invasive elsewhere	*5*
**B. Biology/Ecology**	**36**
4. Undesirable (or persistence) traits	*12*
5. Resource exploitation	*2*
6. Reproduction	*7*
7. Dispersal mechanisms	*9*
8. Tolerance attributes	*6*
**C. Climate change**	**6**
9. Climate change	*6*
**Sectors affected**	
**Commercial**	**9**
**Environmental**	**0**
**Species or population nuisance traits**	**11**

**Thresholds**	
**BRA**	**41**
**BRA+CCA**	**41**
**Confidence**	
**BRA+CCA**	**0.53**
**BRA**	**0.54**
**CCA**	**0.50**

Date and Time
15/08/2024 10:21:54

TPS-ISK v2

**Taxon and Assessor details**	
Category	Gymnosperms
Taxon name	*Picea pungens*
Common name	Blue Spruce
Assessor	Ivica Ljubičić
**Risk screening context**	
Reason and socio-economic benefits	
Risk assessment area	Continental Europe
Taxonomy	
Native range	
Introduced range	
URL	

			**Response**	**Justification (references or other information)**	**Confidence**
**A. Biogeography/Historical**					
1. Domestication/Cultivation					
1	1.01	Has the taxon been the subject of domestication?	Yes	Picea pungens is cultivated for the blue-green color of the needles, which differs from the native population. / Manual of Woody Landscape Plants: Their Identification, Ornamental Characteristics, Culture, Propogation and Uses Paperback (1990).	High
2	1.02	Is the taxon harvested in the wild and likely to be sold or used in its live form?	Yes	Picea pungens is often used as an ornamental plant in gardens, parks and urban landscapes. Its distinctive blue or silver-blue needle color makes it a popular choice for adding color and texture to garden designs. / Manual of Woody Landscape Plants: Their Identification, Ornamental Characteristics, Culture, Propogation and Uses Paperback (1990).	High
3	1.03	Does the taxon have invasive races, varieties, sub-taxa or congeners?	No	Flora of North America / National Invasive Species Information Center (NISIC)	High
2. Climate, distribution and introduction risk					
4	2.01	How similar are the climatic conditions between the risk assessment area and the taxon's native range?	Medium	https://doi.org/10.1038/ngeo1595	Medium
5	2.02	What is the quality of the climate matching data?	High	https://worldclim.org/data/worldclim21.html#google_vignette	High
6	2.03	Is the taxon known to be present outside of cultivation in the risk assessment area?	Yes	https://www.gbif.org/species/5284871	High
7	2.04	How many potential vectors could the taxon use to enter the risk assessment area?	>1	Manual of Woody Landscape Their Identification, Ornamental Characteristics, Culture, Propogation and Uses 6th edition by Michael A. Dirr. Stipes Pub Llc,2009	High
8	2.05	Is the taxon currently found in close proximity to, and likely to enter, the risk assessment area in the near future (e.g. unintentional or intentional introductions)?	Yes	https://www.gbif.org/species/5284871	Medium
3. Invasive elsewhere					
9	3.01	Has the taxon become naturalised outside its native range?	Yes	It is grown in Central Europe as an ornamental species and for wood pulp. https://www.gbif.org/species/5284871	Very high
10	3.02	In the taxon's introduced range, are there any known adverse impacts to wild or commercial species?	No	Picea pungens can grow and spread rapidly, potentially displacing native tree species. It can compete with commercial tree species for water, nutrients and sunlight.	Low
11	3.03	In the taxon's introduced range, are there any known adverse impacts to agriculture or forestry?	No	Professional judgement	Low
12	3.04	In the taxon's introduced range, are there any known adverse impacts to ecosystem	No	Professional judgement	Low
13	3.05	In the taxon's introduced range, are there any known adverse socio-economic impacts?	No	Professional judgement	Low
**B. Biology/Ecology**					
4. Undesirable (or persistence) traits					
14	4.01	Is the taxon likely to be poisonous or pose other risks to human health?	No	Professional judgement	Low
15	4.02	Is the taxon likely to suppress the growth of one or more native species?	Yes	Picea pungens can compete with native plant species for resources such as light, water and nutrients, which can lead to a decrease in biodiversity and changes in ecosystem structure.	Low
16	4.03	Are there any threatened or protected native species that the taxon would parasitise in the risk assessment area?	No	Picea pungens is not known as a parasitic plant species. In its natural habitat does not usually parasitize other plants; instead, it grows as an independent tree or together with other species in forest stands. ISBN: 9780801892431	High
17	4.04	Is the taxon adaptable in terms of climatic and other environmental conditions, thus enhancing its potential persistence if it has invaded or is likely to invade the risk assessment area?	Yes	When introduced to other parts of the world as an ornamental, Picea pungens can show adaptability to a variety of climates, including temperate or mild climates. However, its adaptability may vary depending on local conditions, such as soil, sunlight and water availability.	Medium
18	4.05	Is the taxon likely to disrupt terrestrial ecosystem function if it has invaded or is likely to invade the risk assessment area?	No	Picea pungens does not usually pose a significant risk to terrestrial ecosystems because it is an endemic species that has evolved in the specific conditions of the mountainous regions of the western United States. The invasiveness of Picea pungens has not been recorded outside of its natural environment, so the actual potential to disrupt terrestrial ecosystems is very small.	Low
19	4.06	Is the taxon likely to exert adverse impacts on ecosystem services in the risk assessment area?	No	Like other plants, there is the potential for Picea pungens to cause changes in the ecosystems to which it has been introduced. These impacts may include competition with native plants for resources, changes in wildlife habitats, and changes in ecosystem structure and function.	Low

20	4.07	Is the taxon likely to host or function as a vector for recognised pests and pathogens that are present in the risk assessment area?	No	It is not known to be particularly susceptible or resistant to certain pests or pathogens compared to other conifer species. With proper care and management, pests and pathogens are usually not a significant problem for this plant species.	Medium
21	4.08	Is the taxon likely to host or function as a vector for recognised pests and pathogens that are absent in the risk assessment area?	No	Professional judgement	Low
22	4.09	Is the taxon likely to be released from cultivation?	Yes	https://www.gbif.org/species/5284871	High
23	4.10	Is the taxon versatile in habitat use?	Yes	Within the species Picea pungens there are different cultivars that may have different levels of adaptability to specific habitats or conditions. For example, there are several cultivars that have been bred for horticultural purposes and can adapt to different climates and soil types. It is known for its resistance to droughts and extreme temperatures.	Medium
24	4.11	Is it likely that the taxon's mode of existence or behaviours will reduce habitat quality for native species?	No	It is not known to be toxic to animals. Its needles can be sharp and hard, which can cause irritation in the mouth and digestive tract of animals if consumed in large quantities. The resin it produces can be irritating to the skin of animals if it comes into	Medium
25	4.12	Is the taxon likely to maintain a viable population even when present in low densities (or persist in adverse conditions by way of a dormant form)?	Yes	It prefers dense forests or groups of trees where it has greater protection from the wind and competition from other plant species. However, it can also grow in conditions of low density, but this can affect its form and development, so it can develop wider crowns and less concentrated branches. Variation in environmental conditions, understorey species number, abundance and composition among natural and managed Picea abies forest stands. Forest Ecology and Management 177 (2003):	Medium
5. Resource exploitation					
26	5.01	Is the taxon likely to put threatened or protected native species under competitive pressure in the risk assessment area?	No	Professional judgement	Low
27	5.02	Is the taxon likely to sequester resources to the detriment of native species in the risk assessment area?	No	Professional judgement	Low
6. Reproduction					
28	6.01	Is the taxon likely to exhibit changes in reproductive strategy in response to environmental conditions?	Not applicable	no reference	Low
29	6.02	Is the taxon likely to produce viable propagules in the risk assessment area?	Yes	has propagules	High
30	6.03	Is the taxon likely to hybridise with native species under natural conditions?	Yes	Some species of the genus Picea can hybridize. https://doi.org/10.1139/b78-303	Low
31	6.04	Is the taxon likely to be hermaphroditic or to exhibit asexual reproduction?	No	Picea pungens is not hermaphroditic, but a dioecious plant. It can be propagated asexually by cuttings or seedlings.	Medium
32	6.05	Is the taxon dependent on the presence of another species (or specific habitat features) to complete its life cycle?	No	Picea pungens is pollinated by the wind, not by insects or other pollinators.	Medium
33	6.06	Is the taxon likely to produce a large number of propagules?	Yes	has propagules	Medium
34	6.07	How many time units (days, months, years) does the taxon require to reach the age at first reproduction?	8	DOI: 10.3389/fevo.2022.869130	High
7. Dispersal mechanisms					
35	7.01	How many potential vectors or pathways could the taxon use to disperse within the risk assessment area (with suitable habitats nearby)?	>1	It spreads naturally through the dispersal of seeds contained in cones that can be carried by wind from the mother plant. It can also be transferred to new locations by humans as an ornamental plant.	Low
36	7.02	Will any of these vectors or pathways bring the taxon in close proximity to one or more protected areas?	Yes	professional judgement	Low
37	7.03	Does the taxon have a specialised means of attachment such that it enhances the likelihood of dispersal?	No	professional judgement	Low
38	7.04	Is natural dispersal of the taxon likely to occur as seeds or spores in the risk	Yes	anemophilic plant	Medium
39	7.05	Is natural dispersal of the taxon likely to occur as fragments or seedlings in the risk assessment area?	Yes	by human action in the production of seedlings	Medium
40	7.06	Are any life stages of the taxon likely to migrate into the risk assessment area for reproduction?	Yes	Koski, V., T. Skrøppa, L. Paule, H. Wolf and J. Turok. 1997. Technical guidelines for genetic conservation of Norway spruce (Picea abies (L.) Karst). International Plant Genetic Resources	Medium
41	7.07	Are propagules of the taxon likely to be dispersed in the risk assessment area by other species?	No	has not active dispersal mechanisms	Medium
42	7.08	Is dispersal of the taxon along any of the vectors or pathways mentioned in the previous seven Questions (35–41: i.e. either unintentional or intentional) likely to be	Yes	professional judgement	Low
43	7.09	Is dispersal of the taxon density dependent?	Yes	professional judgement	Low
8. Tolerance attributes					
44	8.01	Is the taxon able to withstand being in water for extended periods at some stage of its life cycle?	No	Picea pungens is not a species that is naturally adapted to a long stay in water, it prefers well-drained soil. If it is exposed to a prolonged stay in water, it is likely to suffer from a lack of oxygen and may experience root rot or other adverse effects.	High

45	8.02	Is the taxon tolerant of a wide range of soil or air quality conditions?	Yes	It is quite tolerant of different soil types and can grow in a wide range of soil qualities, including different textures, pH values and humidity. However, it prefers well-drained soils with a slightly acidic to neutral pH value. Also, it reacts well to soils that are rich in humus. In terms of air quality, it can tolerate different conditions, including those with slightly higher concentrations of	Low
46	8.03	Can the taxon be controlled or eradicated in the wild with chemical, biological or other agents and means?	Yes	Control or eradication of Picea pungens can be attempted using a variety of methods, including chemical, biological, or other methods, but the success of these methods may vary depending on specific conditions and resources. Herbicides can be used to control the growth of Picea pungens. Manual removal or grubbing of Picea pungens can be an effective method of population control. This method can be practical for smaller areas or individual	Low
47	8.04	Is the taxon likely to benefit from environmental or human disturbance?	Not applicable	no reference	Low
48	8.05	Is the taxon able to tolerate soil acidity, salinity or other parameter levels that are higher or lower than those found in its usual environment?	Yes	Picea pungens is known to be quite tolerant of different soil types and can grow in a wide range of soil qualities, including different textures, pH values and moisture. However, it prefers well-drained soils with a slightly acidic to neutral pH value.	Low
49	8.06	Are there effective natural enemies of the taxon present in the risk assessment area?	Yes	A variety of insect species can attack Picea pungens, including Ips spp., Rhyacionia spp., Choristoneura spp., and various species of spruce moths. These insects can cause damage to needles, branches or bark, which can weaken the plant. Various fungal diseases, such as powdery mildew, rust, rust, and others, can attack Picea pungens and cause damage or decay of the plant. Some animals, such as deer, rabbits or roe deer, can feed on	Medium
**C. Climate change**					
9. Climate change					
50	9.01	Under the predicted future climatic conditions, are the risks of entry into the risk assessment area posed by the taxon likely to increase, decrease or not change?	Decrease	Picea pungens is more resistant to cold and drought than most other types of spruce. Since climate change is usually associated with higher temperatures and changes in precipitation, there is no risk that areas previously unsuitable for Picea pungens could change and become more favorable for its growth and spread.	Low
51	9.02	Under the predicted future climatic conditions, are the risks of establishment within the risk assessment area posed by the taxon likely to increase, decrease or not change?	Decrease	Picea pungens is more resistant to cold and drought than most other types of spruce. Since climate change is usually associated with higher temperatures and changes in precipitation, there is no risk that areas previously unsuitable for Picea pungens could change and become more favorable for its growth and spread.	Low
52	9.03	Under the predicted future climatic conditions, are the risks of dispersal within the risk assessment area posed by the taxon likely to increase, decrease or not change?	Decrease	Picea pungens is more resistant to cold and drought than most other types of spruce. Since climate change is usually associated with higher temperatures and changes in precipitation, there is no risk that areas previously unsuitable for Picea pungens could change and become more favorable for its growth and spread.	Low
53	9.04	Under the predicted future climatic conditions, what is the likely magnitude of future potential impacts by the taxon on biodiversity or ecosystem integrity and	No change	no reference	Low
54	9.05	Under the predicted future climatic conditions, what is the likely magnitude of future potential impacts by the taxon on ecosystem structure or function?	No change	no reference	Low
55	9.06	Under the predicted future climatic conditions, what is the likely magnitude of future potential impacts by the taxon on ecosystem services and related socio-	No change	no reference	Low

**Statistics**	
**Scores**	
**BRA**	**19.0**
**BRA Outcome**	**Medium**
**BRA+CCA**	**13.0**
**BRA+CCA Outcome**	**Medium**
**Score partition**	
**A. Biogeography/Historical**	**7.0**
1. Domestication/Cultivation	*2.0*
2. Climate, distribution and introduction risk	*4.0*
3. Invasive elsewhere	*1.0*
**B. Biology/Ecology**	**12.0**
4. Undesirable (or persistence) traits	*5.0*
5. Resource exploitation	*0.0*
6. Reproduction	*2.0*
7. Dispersal mechanisms	*5.0*
8. Tolerance attributes	*0.0*
**C. Climate change**	**-6.0**
9. Climate change	*-6.0*
**Answered Questions**	
Total	***55***
**A. Biogeography/Historical**	**13**
1. Domestication/Cultivation	*3*
2. Climate, distribution and introduction risk	*5*
3. Invasive elsewhere	*5*
**B. Biology/Ecology**	**36**
4. Undesirable (or persistence) traits	*12*
5. Resource exploitation	*2*
6. Reproduction	*7*

7. Dispersal mechanisms	*9*
8. Tolerance attributes	*6*
**C. Climate change**	**6**
9. Climate change	*6*
**Sectors affected**	
**Commercial**	**9**
**Environmental**	**1**
**Species or population nuisance traits**	**10**

**Thresholds**	
**BRA**	**41**
**BRA+CCA**	**41**
**Confidence**	
**BRA+CCA**	**0.43**
**BRA**	**0.45**
**CCA**	**0.25**

**Date and Time**
**26/06/2024 12:31:47**

TPS-ISK v2

**Taxon and Assessor details**	
Category	Gymnosperms
Taxon name	*Pinus strobus*
Common name	Eastern white pine
Assessor	Ivana
**Risk screening context**	
Reason and socio-economic benefits	
Risk assessment area	Continental Europe
Taxonomy	
Native range	
Introduced range	
URL	

			Response	Justification (references or other information)	Confidence
**A. Biogeography/Historical**					
1. Domestication/Cultivation					
1	1.01	Has the taxon been the subject of domestication?	Yes	Yes was farmed last 100 years in RA area. https://www.afrjournal.org/index.php/afr/article/view/150/196 http://www.editurasilvica.ro/afr/51/1/radu_s.pdf Seed production Eastern White Pine normally begins seed production at 5 to 10 years of age (Fowells, 1965; Sargent, 1965), although little pollen is produced during the early years of flowering (Wendel and Smith, 1990). The interval between heavy seed crops is usually 3 to 10 years (Krugman and Jenkinson, 1974; Wendel and Smith, 1990), becoming less frequent as trees become over mature (Horton and Bedell, 1960). A study in Germany recorded seed production as high as 73 kg/ha in a 90-year-old stand (Messer, 1956), while in Maine, a stand considered to be intermediate in density with a basal area of 28 m2 /ha produced over 4.4 million seeds per hectare in a "bumper" year (Graber, 1970). This	Medium
2	1.02	Is the taxon harvested in the wild and likely to be sold or used in its live form?	Yes	Eastern White Pine is used for construction, millwork, trim, and pulpwood. Younger trees and plantations have replaced the once seemingly inexhaustible lumber supply of virgin forests. Constituents: Vit C (needles), many different acids in needles, essential oils (including terpenes, monoterpenes, sesqueterpenes), resin, starch (and more....) Herbal Actions: Expectorant, circulatory stimulant, mild diuretic, pectoral, immune stimulant, grief support Medicinal Uses: Specific for respiratory and bronchial complaints. A tea of the needles, or a decoction of the needles with thin twigs included (the strongest preparation), is helpful to promote expectoration and removal and thinning of mucous from the lungs. Suitable for both wet and dry coughs but when there's dryness I like to combine it with moistening demulcent herbs (see cough recipe below for a balanced formula). Use for coughs, colds, bronchitis, laryngitis, croup. A pea-sized piece of the pitch can also be chewed to promote expectoration. https://www.milkandhoneyherbs.com/blog/2018/1/19/white-pine-medicine	Very high
3	1.03	Does the taxon have invasive races, varieties, sub-taxa or congeners?	Yes	Yes Pinus halepensis - ref: Lavi, A., Perevolotsky, A., Kigel, J., & Noy-Meir, I. (2005). Invasion of Pinus halepensis from plantations into adjacent natural habitats. Applied Vegetation Science, 8(1), 85-92. https://www.researchgate.net/publication/227666227_Invasion_of	Very high
2. Climate, distribution and introduction risk					
4	2.01	How similar are the climatic conditions between the risk assessment area and the taxon's native range?	High	Mandak, B., Hadincová, V., Mahelka, V., & Wildova, R. (2013). European invasion of North American Pinus strobus at large and fine scales: High genetic diversity and fine-scale genetic clustering over time in the adventive range. PLoS One, 8(7), e68514. Radu S (2008). Pinus strobus: past and future in Europe. A page of silvicultural history and international scientific cooperation. Annals of Forest Research 51:133-140. https://www.afrjournal.org/ index.php/afr/article/viewFile/150/196	Very high
5	2.02	What is the quality of the climate matching data?	High	https://www.davey.com/media/t20nwe5f/davey_climate_change_northeast_projections.pdf	Very high
6	2.03	Is the taxon known to be present outside of cultivation in the risk assessment area?	Yes	Mandak, B., Hadincová, V., Mahelka, V., & Wildova, R. (2013). European invasion of North American Pinus strobus at large and fine scales: High genetic diversity and fine-scale genetic clustering over time in the adventive range. PLoS One, 8(7), e68514.https://www.euforgen.org/species/pinus-strobus/ https://www.monumentaltrees.com/en/europe-pinusstrobus/	Very high
7	2.04	How many potential vectors could the taxon use to enter the risk assessment area?	>1	Pinus strobus is an outcrossing, wind-pollinated species whose winged seeds are dispersed by wind. In the adventive area, individual trees can be fertile at the age of twenty years, with the substantial production of seeds at the age of 50 years and older (Mandak et al. 2013).so yes, unintentional and intentional	Very high

8	2.05	Is the taxon currently found in close proximity to, and likely to enter, the risk assessment area in the near future (e.g. unintentional or intentional introductions)?	Yes	Mandak, B., Hadincová, V., Mahelka, V., & Wildova, R. (2013). European invasion of North American Pinus strobus at large and fine scales: High genetic diversity and fine-scale genetic clustering over time in the adventive range. PLoS One, 8(7), e68514. Radu S (2008). Pinus strobus: past and future in Europe. A page of silvicultural history and international scientific cooperation. Annals of Forest Research 51:133-140. https://www.afrjournal.org/ index.php/afr/article/viewFile/150/196 Status in Europe:	Very high
3. Invasive elsewhere					
9	3.01	Has the taxon become naturalised outside its native range?	Yes	Stephan, B. R. (2004). Studies of genetic variation with five-needle pines in Germany. In Proceedings of the IUFRO Five-needle Pines WP Conference. Breeding and Genetic Resources of Five-needle Pines: Growth, adaptability, and Pest Resistance. Edited by RA Sniezko, S. Samman, SE Schlarbaum, and HB Kriebel. USDA Forest Service, Medford, USA (pp. 98-102). Mandak, B., Hadincová, V., Mahelka, V., & Wildova, R. (2013). European invasion of North American Pinus strobus at large and fine scales: High genetic diversity and fine-scale genetic clustering over time in the adventive range. PLoS One, 8(7), e68514. Radu S (2008). Pinus strobus: past and future in Europe. A page of silvicultural history and international scientific cooperation. Annals of Forest Research 51:133-140. https://www.afrjournal.org/	High
10	3.02	In the taxon's introduced range, are there any known adverse impacts to wild or commercial species?	Yes	Wohlgemuth, T., Gossner, M. M., Campagnaro, T., Marchante, H., van Loo, M., Vacchiano, G., ... & Silva, J. S. (2022). Impact of non-native tree species in Europe on soil properties and biodiversity: a review. NeoBiota, 78, 45-69. Radu S (2008). Pinus strobus: past and future in Europe. A page of silvicultural history and international scientific cooperation. Annals of Forest Research 51:133-140. https://www.afrjournal.org/	High
11	3.03	In the taxon's introduced range, are there any known adverse impacts to agriculture or forestry?	Yes	Lavi, A., Perevolotsky, A., Kigel, J., & Noy-Meir, I. (2005). Invasion of Pinus halepensis from plantations into adjacent natural habitats. Applied Vegetation Science, 8(1), 85-92.	Very high
12	3.04	In the taxon's introduced range, are there any known adverse impacts to ecosystem	Yes	it's neophyte species	Medium
13	3.05	In the taxon's introduced range, are there any known adverse socio-economic impacts?	No	In my opinion, the adverse can be fires. Fernandes, P. M., Vega, J. A., Jimenez, E., & Rigolot, E. (2008). Fire resistance of European pines. Forest Ecology and Management, 256(3), 246-255. http://publicationslist.org/data/pfern/ref-25/Fernandes%20et%20al.%20FEM%202008.pdf	Medium
**B. Biology/Ecology**					
4. Undesirable (or persistence) traits					
14	4.01	Is the taxon likely to be poisonous or pose other risks to human health?	No	It's medicinal plant del Castillo, R. F., & Acosta, S. (2002). Ethnobotanical notes on Pinus strobus var. chiapensis. Anales del Instituto de Biología. Serie Botánica, 73(2), 319-327. Matsuura, S. (1957). The Structure of Cryptostrobin and Strobopinin, the Flavanones from the Heartwood of Pinus strobus. Pharmaceutical bulletin, 5(3), 195-198. etc.	Very high
15	4.02	Is the taxon likely to suppress the growth of one or more native species?	Yes	Due to a combination of effective seed spread, high seedling recruitment, fast growth and enormous litter production (Hadincová et al. 2007; Kubartová 2007), P. strobus inhibits the growth of understorey vegetation and regeneration of native trees, especially Pinus sylvestris L. According to Carrillo-Gavilan and Vila (2010), P. strobus is the only invasive conifer in Europe. The role of mutualistic symbioses, including EcM, in the P. strobus, invasions remain unknown. Kohout, P., Sýkorová, Z., Bahram, M. et al. Ericaceous dwarf shrubs affect ectomycorrhizal fungal community of the invasive Pinus strobus and native Pinus sylvestris in a pot experiment. Mycorrhiza 21, 403–412 (2011). https://doi.org/10.1007/s00572-010-0350-2 Hadincová V, Köhnleinová I, Marešová J (2007) Invasive behaviour of white pine (Pinus strobus L.) in sandstone areas in the Czech Republic. In: Härtel H, Cílek V, Herben T, Jackson A, Williams R (eds) Sandstones Landscapes. Academia, Prague, pp 219–224 Kubartová A (2007) Decomposition of needle litter in Pinus sylvestris and Pinus strobus forests in the Bohemian Switzerland National Park (Czech Republic). In: Härtel H, Cílek V, Herben T,	Very high
16	4.03	Are there any threatened or protected native species that the taxon would parasitise in the risk assessment area?	Yes	yes, Pinus sylvestris, but there is a plenty of species more.... Kohout, P., Sýkorová, Z., Bahram, M. et al. Ericaceous dwarf shrubs affect ectomycorrhizal fungal community of the invasive Pinus strobus and native Pinus sylvestris in a pot experiment. Mycorrhiza 21, 403–412 (2011). https://doi.org/10.1007/s00572-	Very high
17	4.04	Is the taxon adaptable in terms of climatic and other environmental conditions, thus enhancing its potential persistence if it has invaded or is likely to invade the risk	Yes	Hadincová V, Köhnleinová I, Marešová J (2007) Invasive behaviour of white pine (Pinus strobus L.) in sandstone areas in the Czech Republic. In: Härtel H, Cílek V, Herben T, Jackson A, Williams R (eds) Sandstones Landscapes. Academia, Prague, pp	Very high
18	4.05	Is the taxon likely to disrupt terrestrial ecosystem function if it has invaded or is likely to invade the risk assessment area?	Yes	Hadincová V, Köhnleinová I, Marešová J (2007) Invasive behaviour of white pine (Pinus strobus L.) in sandstone areas in the Czech Republic. In: Härtel H, Cílek V, Herben T, Jackson A, Williams R (eds) Sandstones Landscapes. Academia, Prague, pp	Very high
19	4.06	Is the taxon likely to exert adverse impacts on ecosystem services in the risk assessment area?	Yes	Hadincová V, Köhnleinová I, Marešová J (2007) Invasive behaviour of white pine (Pinus strobus L.) in sandstone areas in the Czech Republic. In: Härtel H, Cílek V, Herben T, Jackson A, Williams R (eds) Sandstones Landscapes. Academia, Prague, pp	High

20	4.07	Is the taxon likely to host or function as a vector for recognised pests and pathogens that are present in the risk assessment area?	No	Kohout, P., Sýkorová, Z., Bahram, M. et al. Ericaceous dwarf shrubs affect ectomycorrhizal fungal community of the invasive Pinus strobus and native Pinus sylvestris in a pot experiment. Mycorrhiza 21, 403–412 (2011). https://doi.org/10.1007/s00572-	Medium
21	4.08	Is the taxon likely to host or function as a vector for recognised pests and pathogens that are absent in the risk assessment area?	Yes	Bormann, F. H. (1966). The structure, function, and ecological significance of root grafts in Pinus strobus L. Ecological Monographs, 36(1), 2-26. https://www.jstor.org/stable/1948486 However, emerging health issues of eastern white pine have increased dramatically in recent decades, raising serious concerns over the health and future of this species in N America (Costanza et al. 2018). Costanza, K. K., Whitney, T. D., McIntire, C. D., Livingston, W. H., & Gandhi, K. J. (2018). A synthesis of emerging health issues of eastern white pine (Pinus strobus) in eastern North America. Forest ecology and management, 423, 3-17. Allelopathy Pinus died Chick, T. A., & Kielbaso, J. J. (1998). Allelopathy as an inhibition factor in ornamental tree growth: implications from the literature. Journal of Arboriculture, 24, 274-279. file:///C:/Users/korisnik/Downloads/p0274-0279.pdf in US and Canada ... Prasad, A., Leites, L. Ecological analysis of intraspecific variability of eastern white pine (Pinus strobus) under climate change by combining provenance and demographic data. Landsc Ecol 37, 109–128 (2022). https://doi.org/10.1007/s10980-	Very high
22	4.09	Is the taxon likely to be released from cultivation?	Yes	https://www.missouribotanicalgarden.org/PlantFinder/PlantFinderDetails.aspx?taxonid=285004This tree grows at a fast rate, with height increases of more than 24" per year.	Very high
23	4.10	Is the taxon versatile in habitat use?	No	Eastern white pine (Pinus strobus) can be a majestic plant in the landscape. It prefers a soil pH that falls in the 4.5 to 6.0 range. When used in the landscape, white pine are frequently planted as a windbreak tree amidst a sea of turfgrass where the soil has been limed repeatedly to maintain a soil pH of around 6.5. White pines planted in high-pH soils can experience nutrient imbalances and may "fail to thrive" in these hostile sites. When counseling homeowners and landscape managers, we often encourage them to avoid limiting the turf areas adjacent to the white pine plantings to preserve the white pine's health. https://extension.psu.edu/avoid-epic-landscape-fails-know-the-preferred-ph-ranges-of-ornamental-plants Boucher, J. F., Bernier, P. Y., & Munson, A. D. (2001). Radiation and soil temperature interactions on the growth and physiology of eastern white pine (Pinus strobus L.) seedlings. Plant and soil, 236, 165-174.	High
24	4.11	Is it likely that the taxon's mode of existence or behaviours will reduce habitat quality for native species?	Yes	only in soil ph range, it will acidify the habitat	Low
25	4.12	Is the taxon likely to maintain a viable population even when present in low densities (or persist in adverse conditions by way of a dormant form)?	Yes	In the adventive area, individual trees can be fertile at the age of twenty years, with the substantial production of seeds at the age of 50 years and older (Mandak et al. 2013).Mandak, B., Hadincová, V., Mahelka, V., & Wildova, R. (2013). European invasion of North American Pinus strobus at large and fine scales: High genetic diversity and fine-scale genetic clustering over time in the adventive range. PLoS One, 8(7), e68514.	Very high
5. Resource exploitation					
26	5.01	Is the taxon likely to put threatened or protected native species under competitive pressure in the risk assessment area?	Yes	Kubartová A (2007) Decomposition of needle litter in Pinus sylvestris and Pinus strobus forests in the Bohemian Switzerland National Park (Czech Republic). In: Härtel H, Cílek V, Herben T, Jackson A, Williams R (eds) Sandstones landscapes. Academia,	Medium
27	5.02	Is the taxon likely to sequester resources to the detriment of native species in the risk assessment area?	No	my opinion	Medium
6. Reproduction					
28	6.01	Is the taxon likely to exhibit changes in reproductive strategy in response to environmental conditions?	No	my opinion	Medium
29	6.02	Is the taxon likely to produce viable propagules in the risk assessment area?	Yes	Mandak, B., Hadincová, V., Mahelka, V., & Wildova, R. (2013). European invasion of North American Pinus strobus at large and fine scales: High genetic diversity and fine-scale genetic clustering over time in the adventive range. PLoS One, 8(7),	High

30	6.03	Is the taxon likely to hybridise with native species under natural conditions?	Yes	Crosses Other members of subgenus Strobus do not occur within the natural range of Eastern White Pine, and introgressive hybridisation does not occur. Most artificial crosses among North American members of subsection Strobi have been successful, the exception being those involving Pinus lambertiana Dougl. (Critchfield, 1986; Critchfield and Kinloch, 1986). Successful crosses involving Eastern White Pine are summarised in Table 3.1. Only two of these hybrids have been widely field tested, including that with P. monticola Dougl. and its reciprocal, and with P. wallichiana A.B. Jackson (formerly P. griffithii McClell.) and its reciprocal (Kriebel, 1983). No successful hybrid crosses have been reported with species in other sections of Pinus (Critchfield, 1975). Table 3.1 Summary of successful crosses with P. strobus Species Origin References P. monticola Dougl. ex D.Don. western US and Canada Wright, 1959, 1970; Kriebel, 1972b P. wallichiana A.B. Jackson (syn P. griffithi McClell., P. excelsa Wallich ex D.Don), hybrid = P. ꝑschwerinii Fitschen Himalayas Wright, 1959, 1970; Kriebel, 1972b; Garrett, 1979; Zsuffa, 1979b; Blada, 1992 P. ayacahuite Ehrenb. Mexico, Guatemala Johnson and Heimburger, 1946; Wright, 1959; Garrett, 1979 P. parviflora Sieb. and Zucc. hybrid = P. ꝑhunnewelli A.G. Johnson Japan Johnson, 1952; Wright, 1959 P. peuce Griseb. S.E. Europe Fowler and Heimburger, 1958; Radu, 1976; Santamour and Zinkel, 1978 P. flexilis James (only one parent successful, may in fact be P.	High
31	6.04	Is the taxon likely to be hermaphroditic or to exhibit asexual reproduction?	No	E. Vegetative reproduction in nature Eastern White Pine does not regenerate vegetatively under natural conditions (Wendel and Smith, 1990). Eastern White Pine is monoecious. Production of female strobili occurs as early as 4 years (Buckingham, 1963) while pollen production may not start for 10 to 20 years (Wright, 1970). https://www.oecd-ilibrary.org/docserver/9789264095403-2-en.pdf?expires=1685382471&id=id&accname=guest&checksum=1	Very high
32	6.05	Is the taxon dependent on the presence of another species (or specific habitat features) to complete its life cycle?	No	https://www.srs.fs.usda.gov/pubs/misc/ag_654/volume_1/pinus/strobus.htm	Very high
33	6.06	Is the taxon likely to produce a large number of propagules?	No	In a comprehensive German study of white pine seed production, it was found that a 90-year-old stand produced about 73 kg of seeds per hectare (65 lb/acre); a comparable 60-year-old stand produced only one-fifth as much. In these stands, dominant trees produced twice as many cones as codominant trees (71). In Maine, intermediate density stands 27.6 m²/ha (120 ft²/acre) produced 4,430,000 viable seeds per hectare (1,793,220/acre) in a good seed year (29). In high density stands 42.9 m²/ha (187 ft²/acre), seedfall was 36 percent less and in low density stands 18.4 m²/ha (80 ft²/acre), seedfall was 30 percent less than in the intermediate density stands. There are 58,400 seeds per kilogram (26,500/1b) with a range from 38,600 to 116,800/kg (17,500 to 53,000/1b) (39). In a study of 250 different parents from all parts of the white pine range, the number of good seeds per cone ranged from 0 to 73. The lowest sets were found in stands at the extremes of the range. https://www.srs.fs.usda.gov/pubs/misc/ag_654/volume_1/pinus/st	High
34	6.07	How many time units (days, months, years) does the taxon require to reach the age at first reproduction?	10	Trees may start to bear female flowers when 5 to 10 years old (71). In the Philadelphia area, quantity production of female flowers does not begin until the trees are about 6 m (20 ft) tall. At that size, 200 to 300 flowers may be produced in 1 year; the number is only a little greater on larger or older trees. Few or no male flowers appear during the early flowering years. Femaleness persists even on older trees 30 to 61 cm (12 to 24 in) in diameter, although trees of this size do produce small to moderate amounts of pollen (71). https://www.srs.fs.usda.gov/pubs/misc/ag_654/volume_1/pinus/strobus.htm Wilson, Robert W., and William F. McQuilkin. 1965. In Silvics of forest trees of the United States. p. 329-337. H. A. Fowells, comp. U.S. Department of Agriculture, Agriculture	Medium
7. Dispersal mechanisms					
35	7.01	How many potential vectors or pathways could the taxon use to disperse within the risk assessment area (with suitable habitats nearby)?	One	sexual (unintentional and intentional) White pine is monoecious. The male strobili are oval, 8 to 10 mm (0.3 to 0.4 in) long and occur mostly on the basal part of new shoots and mostly on older lateral branches in the lower crown. At the time of pollen shed, they are light brown to brown. Female flowers are found most often in the upper crown, primarily at the apical end of the main branches in the position of subterminal or lateral buds (39). At the time of pollination, they are green, and 5 to 38 mm (0.2 to 1.5 in) long. In the northeastern United States, flowering occurs between May and June. The male flowers develop from one to several weeks before the female flowers. https://www.srs.fs.usda.gov/pubs/misc/ag_654/volume_1/pinus/st	High
36	7.02	Will any of these vectors or pathways bring the taxon in close proximity to one or more protected areas?	Yes	It's already noted in many countries and Croatia too, the present od P. strobus in the protected areas	High

37	7.03	Does the taxon have a specialised means of attachment such that it enhances the likelihood of dispersal?	Yes	Good seed years are thought to occur every 3 to 5 years, a few seeds being produced in most intervening years. However, at the Massabesic Experimental Forest in southwestern Maine, and at other New England locations, there was virtually no seed produced for 7 years and no good seed crop for 10 years. The major cause of these failures probably is the white pine cone beetle (Conophthorus coniperda). Cones are green when immature and turn yellow-green to light brown when ripe. Cones that float in linseed oil are considered ripe. Cones should be collected from trees having superior growth and form. Widely spaced dominant trees with full crowns produce the most seeds per cone (39). Krugman, Stanley L., and James L. Jenkinson. 1974. Pinus L. Pine, In Seeds of woody plants in the United States. p. 598-638. C. S. Schopmeyer, tech. coord. U.S. Department of Agriculture	High
38	7.04	Is natural dispersal of the taxon likely to occur as seeds or spores in the risk assessment area?	Yes	Most of the seeds are dispersed within the month following cone maturity. The seeds travel at least 60 m (200 ft) within a white pine stand and more than 210 m (700 ft) in the open (71). Wilson, Robert W., and William F. McQuilkin. 1965. In Silvics of forest trees of the United States. p. 329-337. H. A. Fowells, comp. U.S. Department of Agriculture, Agriculture Handbook 271. Washington, DC. Gray squirrels were found responsible for much of the white pine reproduction under mature red oak stands in southern New Hampshire; they bury and recover the seeds (3). Alexander, Lee. 1980. The influence of gray squirrels and red oaks in establishing eastern white pine. Thesis (M.S.), University of	Very high
39	7.05	Is natural dispersal of the taxon likely to occur as fragments or seedlings in the risk assessment area?	Yes	Scions from the crown of mature trees can be grafted on young stock (31). Side grafts of scions on 3- or 4-year-old white pine stocks seem to be a more reliable method of vegetative propagation than rooted cuttings (71). Wilson, Robert W., and William F. McQuilkin. 1965. In Silvics of forest trees of the United States. p. 329-337. H. A. Fowells, comp. U.S. Department of Agriculture, Agriculture Handbook 271. Washington, DC. Buds from main terminal or lateral terminal positions should be used in grafting if early erect growth is desired (1). Ahlgren, C. E. 1967. A relationship between scion, bud origin and growth of white pine grafts. Minnesota Forestry Notes 180. University of Minnesota, St.	High
40	7.06	Are any life stages of the taxon likely to migrate into the risk assessment area for reproduction?	Yes	Gray squirrels were found responsible for much of the white pine reproduction under mature red oak stands in southern New Hampshire; they bury and recover the seeds (3). Alexander, Lee. 1980. The influence of gray squirrels and red oaks in establishing eastern white pine. Thesis (M.S.), University of New Hampshire,	High
41	7.07	Are propagules of the taxon likely to be dispersed in the risk assessment area by other species?	No	my opinion	Medium
42	7.08	Is dispersal of the taxon along any of the vectors or pathways mentioned in the previous seven Questions (35–41: i.e. either unintentional or intentional) likely to be	No	https://www.srs.fs.usda.gov/pubs/misc/ag_654/volume_1/pinus/strobus.htm	Medium
43	7.09	Is dispersal of the taxon density dependent?	Yes	In a comprehensive German study of white pine seed production, it was found that a 90-year-old stand produced about 73 kg of seeds per hectare (65 lb/acre); a comparable 60-year-old stand produced only one-fifth as much. In these stands, dominant trees produced twice as many cones as codominant trees (71). Wilson, Robert W., and William F. McQuilkin. 1965. In Silvics of forest trees of the United States. p. 329-337. H. A. Fowells, comp. U.S. Department of Agriculture, Agriculture Handbook 271. Washington, DC. In Maine, intermediate density stands 27.6 m²/ha (120 ft²/acre) produced 4,430,000 viable seeds per hectare (1,793,220/acre) in a good seed year (29). In high density stands 42.9 m²/ha (187 ft²/acre), seedfall was 36 percent less and in low density stands 18.4 m²/ha (80 ft²/acre), seedfall was 30 percent less than in the intermediate density stands. Graber, R. E. 1970. Natural seed fall in white pine stands of varying density. USDA Forest Service, Research Note NE-119. Northeastern Forest	High
8. Tolerance attributes					
44	8.01	Is the taxon able to withstand being in water for extended periods at some stage of its life cycle?	Yes	The survival of white pine 2-2 stock was increased on shallow old pasture soils in eastern Ontario when wedge-shaped pieces of peat saturated with water were placed at the bottom of the planting holes to provide water and prevent desiccation during drought periods (61). Stroempl, G. 1976. Peat wedges aid seedling establishment on shallow soils. Forestry Chronicle 52:47-51. Two-O stock stored in a refrigerator can be planted until mid-June without significant reduction in survival rate. However, seedlings planted in July and August will not be hardened off by the first fall frost (56). Rexrode, K. R., and K. L. Carvell. 1981. The effects of late planting on survival, height growth, and vigor of eastern white pine. Tree Planters' Notes 32: 30-32.	High

45	8.02	Is the taxon tolerant of a wide range of soil or air quality conditions?	No	Reaction to Competition- White pine is intermediate in shade tolerance, and vegetative competition is a major problem (60). Stiell, W. M. 1985. Silviculture of eastern white pine. Proceedings of the Entomological Society of Ontario 116 (Supplement): 95-107. Although it will tolerate up to 80 percent shade, tree growth increases as shade is reduced (6). Balmer, W. E., and H. L. Williston. 1983. Managing eastern white pine in the Southeast. USDA Forest Service, Forestry Report R8-FR 1. Southern Region. 11 p. It can achieve maximum height growth in as little as 45 percent full sunlight (60). In competition with light-foliaged species such as the birches and pitch pine, white pine usually gains dominance in the stand. It can grow successfully in competition with black walnut (15). Camp, Richard F. 1986. Walnuts and white pine can be grown together successfully. Tree Planters'Notes 37:29-31. Against the stronger competition of species such as the aspens, oaks, and maples, however, white pine usually fails to gain a place in the upper canopy and eventually dies (71). Wilson, Robert W., and William F. McQuilkin. 1965. In Silvics of forest trees of the United States. p. 329-337. H. A. Fowells, comp. U.S. Department of Agriculture, Agriculture Handbook 271. Washington, DC. Pure stands of white pine seldom stagnate because of inherent variations in vigor. This characteristic is more pronounced on better sites and in natural stands than in plantations (6). Balmer, W. E., and H. L. Williston	Medium
46	8.03	Can the taxon be controlled or eradicated in the wild with chemical, biological or other agents and means?	Yes	White pine has been regenerated successfully by a wide variety of methods including clearcutting, seed tree, shelterwood, and group selection (44). Little, Silas, Donald E. Beck, and Lino Della-Bianca. 1973. Eastern white pine. In Silvicultural systems for the major forest types of the United States. p. 73-75. U.S. Department of Agriculture, Agriculture Handbook 445. Washington, DC. If there is abundant advanced reproduction, overstory removal is all that is necessary. Clearcutting during or just after heavy seed crops often results in well stocked stands on light soils. Clearcutting in small patches or stands with seed dispersed from adjacent stands is also possible. Because of competition from other vegetation and poor seed crops, mechanical site preparation and planting may be necessary sometimes in conjunction with clearcutting	Medium
47	8.04	Is the taxon likely to benefit from environmental or human disturbance?	Yes	deforestation, agricultural drainage intensive farming The majority of P. strobus stands were deforested by the late 19th century, and therefore, not many old-growth stands are left in its native area. However, because it is among the most rapidly growing northern conifers, it has been used extensively for reforestation projects. Wendel GW, Smith HC (1990) Pinus strobus L. Eastern white pine. In: Burns RM, Honkala BH, editors. Silvics of North America. Vol. 1. Conifers. U.S. Department of Agriculture, Forest Service.	Very high
48	8.05	Is the taxon able to tolerate soil acidity, salinity or other parameter levels that are higher or lower than those found in its usual	Yes	tolerate acidity and acidifies the soil	Medium

49	8.06	Are there effective natural enemies of the taxon present in the risk assessment area?	Yes	There is a lot of insects, but i'm not zoologist so im not sure, these are present in Europe.... Damaging Agents- There are a total of 277 insects and 110 disease organisms known to attack white pine. Only 16 insects and 7 diseases cause sufficient injury or mortality to be of concern. The three most important are white pine weevil (Pissodes strobi), white pine blister rust (Cronartium ribicola), and Armillaria mellea (63). Syme, P. D. 1985. Eastern white pine in Ontario: Its entomological, pathological, physiological and other problems. Proceedings of the Entomological Society of Ontario 116 (Supplement): 21-31. The white pine weevil kills the terminal shoot, which may include the last 2 or 3 years of growth. The tree is seldom killed unless it is very small; lateral branches from the highest live whorl turn upward to produce new terminal shoots. Bole crook and loss of stem length result from this injury (71).Wilson, Robert W., and William F. McQuilkin. 1965. In Silvics of forest trees of the United States. p. 329-337. H. A. Fowells, comp. U.S. Department of Agriculture, Agriculture Handbook 271. Washington, DC. There is evidence that white pine provenances differ in resistance to weevils but even the lowest levels of injury are unacceptable (25).Garrett, P. W. 1973. Geographic variation in resistance to white pine weevil (Pissodes strobi) by eastern white pine (Pinus strobus). The Canadian Entomologist 105:347-350. Among other insect enemies are white pine aphid (Cinara strobi), which causes damage to twigs and branches of large trees and sometimes kills small trees; white pine sawfly (Neodiprion pinetum), which feeds on old and new foliage; Zimmerman pine moth (Dioryctria zimmermani); the Allegheny mound ant (Formica exsectoides), which injects formic acid into the tree tissue; pales weevil (Hylobius pales), which feeds on bark of young twigs and seedlings; pine root collar weevil (H. radicis); European pine shoot moth (Rhyacionia buoliana), which feeds on buds and twigs causing crooked trunks and branches; eastern pine shoot borer (Eucosma gloriola), which attacks terminal needle sheaths, often causing bushiness after repeated attacks; introduced pine sawfly (Diprion similis), which feeds on foliage and may defoliate an entire tree in one season; and white pine cone borer (Eucosma tocullionana), which feeds on white pine cones and is a potentially serious pest (5). Baker, Whiteford L. 1972. Eastern forest insects. U.S. Department of Agriculture, Miscellaneous Publication 1175. Washington, DC. 642 p White pine blister rust (Cronartium ribicola) is highly virulent throughout the range of white pine. Trees are susceptible from the seedling stage through maturity. Blister rust can cause high losses both in regeneration and in immature timber stands (71). Red ring rot caused by Phellinus pini is the most important heart rot of white pine. The fungus enters through wounds, dead limbs, or tips killed by weevils.	Medium
**C. Climate change**					
9. Climate change					
50	9.01	Under the predicted future climatic conditions, are the risks of entry into the risk assessment area posed by the taxon likely to increase, decrease or not change?	Increase	this is my best quess	Medium
51	9.02	Under the predicted future climatic conditions, are the risks of establishment within the risk assessment area posed by the taxon likely to increase, decrease or not	Increase	my opinion	Medium
52	9.03	Under the predicted future climatic conditions, are the risks of dispersal within the risk assessment area posed by the taxon likely to increase, decrease or not change?	No change	its very resisent ro drought Chhin, S., Zalesny, R.S., Parker, W.C. et al. Dendroclimatic analysis of white pine (Pinus strobus L.) using long-term provenance test sites across eastern North America. For. Ecosyst. 5, 18 (2018). https://doi.org/10.1186/s40663-018-0136-0 https://vtechworks.lib.vt.edu/bitstream/handle/10919/86504/Fahey_et_al_2013.pdf?sequence=2&isAllowed=y Vose JM, Swank WT (1994) Effects of long-term drought on the hydrology and growth of a white pine plantation in the southern Appalachians. Forest	High
53	9.04	Under the predicted future climatic conditions, what is the likely magnitude of future potential impacts by the taxon on biodiversity or ecosystem integrity and	Lower	Radu S (2008). Pinus strobus: past and future in Europe. A page of silvicultural history and international scientific cooperation. Annals of Forest Research 51:133-140. https://www.afrjournal.org/ index.php/afr/article/viewFile/150/196	Medium
54	9.05	Under the predicted future climatic conditions, what is the likely magnitude of future potential impacts by the taxon on ecosystem structure or function?	Higher	best guess	Medium
55	9.06	Under the predicted future climatic conditions, what is the likely magnitude of future potential impacts by the taxon on ecosystem services and related socio-	No change	best guess	Medium

Statistics	
**Scores**	
**BRA**	**45.0**
**BRA Outcome**	**High**
**BRA+CCA**	**49.0**

**BRA+CCA Outcome**	**High**
**Score partition**	
**A. Biogeography/Historical**	**22.0**
1. Domestication/Cultivation	*4.0*
2. Climate, distribution and introduction risk	*4.0*
3. Invasive elsewhere	*14.0*
**B. Biology/Ecology**	**23.0**
4. Undesirable (or persistence) traits	*9.0*
5. Resource exploitation	*5.0*
6. Reproduction	*1.0*
7. Dispersal mechanisms	*5.0*
8. Tolerance attributes	*3.0*
**C. Climate change**	**4.0**
9. Climate change	*4.0*
**Answered Questions**	
Total	***55***
**A. Biogeography/Historical**	**13**
1. Domestication/Cultivation	*3*
2. Climate, distribution and introduction risk	*5*
3. Invasive elsewhere	*5*
**B. Biology/Ecology**	**36**
4. Undesirable (or persistence) traits	*12*
5. Resource exploitation	*2*
6. Reproduction	*7*
7. Dispersal mechanisms	*9*
8. Tolerance attributes	*6*
**C. Climate change**	**6**
9. Climate change	*6*
**Sectors affected**	
**Commercial**	**15**
**Environmental**	**13**
**Species or population nuisance traits**	**26**

**Thresholds**	
**BRA**	**41**
**BRA+CCA**	**41**
**Confidence**	
**BRA+CCA**	**0.75**
**BRA**	**0.77**
**CCA**	**0.54**

**Date and Time**
**29/05/2023 20:47:13**

TPS-ISK v2

**Taxon and Assessor details**	
Category	Gymnosperms
Taxon name	*Pinus wallichiana*
Common name	Bhutan Pine
Assessor	Mihaela Britvec
**Risk screening context**	
Reason and socio-economic benefits	
Risk assessment area	Continental Europe
Taxonomy	
Native range	
Introduced range	
URL	

			**Response**	**Justification (references or other information)**	**Confidence**
**A. Biogeography/Historical**					
1. Domestication/Cultivation					
1	1.01	Has the taxon been the subject of domestication?	Yes	The tree became available through the European nursery trade in 1836, nine years after the Danish botanist Nathaniel Wallich first introduced seeds to England (https://www.ebben.nl/en/treeebb/piwallic-pinus-	High
2	1.02	Is the taxon harvested in the wild and likely to be sold or used in its live form?	Yes	Pinus wallichiana is a popular tree for planting in parks and large gardens, grown for its attractive foliage and large, decorative cones (https://en.wikipedia.org/wiki/Pinus_wallichiana).	High
3	1.03	Does the taxon have invasive races, varieties, sub-taxa or congeners?	No	For example, Pinus strobus is an invasive plant (https://www.nobanis.org/species-info/?taxaId=2197).	High
2. Climate, distribution and introduction risk					
4	2.01	How similar are the climatic conditions between the risk assessment area and the taxon's native range?	Low	professional judgement	Medium
5	2.02	What is the quality of the climate matching data?	Low	professional judgement	Low
6	2.03	Is the taxon known to be present outside of cultivation in the risk assessment area?	No	no evidence	Low
7	2.04	How many potential vectors could the taxon use to enter the risk assessment area?	>1	dispersion by wind and by humans	High
8	2.05	Is the taxon currently found in close proximity to, and likely to enter, the risk assessment area in the near future (e.g. unintentional or intentional introductions)?	Not applicable	https://www.nobanis.org/species-info/?taxaId=2197	High
3. Invasive elsewhere					
9	3.01	Has the taxon become naturalised outside its native range?	Yes	https://www.nobanis.org/species-info/?taxaId=2197	Medium
10	3.02	In the taxon's introduced range, are there any known adverse impacts to wild or commercial species?	No	no evidence	Low
11	3.03	In the taxon's introduced range, are there any known adverse impacts to agriculture or forestry?	No	no evidence	Low
12	3.04	In the taxon's introduced range, are there any known adverse impacts to ecosystem	No	no evidence	Low
13	3.05	In the taxon's introduced range, are there any known adverse socio-economic impacts?	No	no evidence	Low
**B. Biology/Ecology**					
4. Undesirable (or persistence) traits					
14	4.01	Is the taxon likely to be poisonous or pose other risks to human health?	No	In general, allergy to pine trees is generally held to be rare and clinically insignificant.	High
15	4.02	Is the taxon likely to suppress the growth of one or more native species?	Yes	Pinus wallichiana reducing the amount of plants that can grow under the trees (https://practicalplants.org/wiki/pinus_wallichiana/#Propagation).	Low
16	4.03	Are there any threatened or protected native species that the taxon would parasitise in the risk assessment area?	No	no evidence	Low
17	4.04	Is the taxon adaptable in terms of climatic and other environmental conditions, thus enhancing its potential persistence if it has invaded or is likely to invade the risk	Yes	professional judgement	Low
18	4.05	Is the taxon likely to disrupt terrestrial ecosystem function if it has invaded or is likely to invade the risk assessment area?	Yes	no reference	Low
19	4.06	Is the taxon likely to exert adverse impacts on ecosystem services in the risk	Yes	no reference	Low
20	4.07	Is the taxon likely to host or function as a vector for recognised pests and pathogens that are present in the risk assessment area?	Yes	May be susceptible to adelgids, conifer aphid, sawfly larvae, and pine shoot moth. May be susceptible to honey fungus and needle cast diseases (https://www.rhs.org.uk/plants/13150/pinus-wallichiana/details).	Low
21	4.08	Is the taxon likely to host or function as a vector for recognised pests and pathogens that are absent in the risk assessment area?	Yes	Pinus wallichiana is the primary host for Himalayan dwarf mistletoe (https://en.wikipedia.org/wiki/Pinus_wallichiana).	Low
22	4.09	Is the taxon likely to be released from cultivation?	Yes	It is a very fast growing tree (https://practicalplants.org/wiki/pinus_wallichiana/#Propagation).	Medium
23	4.10	Is the taxon versatile in habitat use?	Yes	https://www.ebben.nl/en/treeebb/piwallic-pinus-wallichiana/pdf/	Low

24	4.11	Is it likely that the taxon's mode of existence or behaviours will reduce habitat quality for native species?	No	no evidence	Low
25	4.12	Is the taxon likely to maintain a viable population even when present in low densities (or persist in adverse conditions by way of a dormant form)?	No	no reference	Low
5. Resource exploitation					
26	5.01	Is the taxon likely to put threatened or protected native species under competitive pressure in the risk assessment area?	No	no evidence	Low
27	5.02	Is the taxon likely to sequester resources to the detriment of native species in the risk assessment area?	No	no evidence	Low
6. Reproduction					
28	6.01	Is the taxon likely to exhibit changes in reproductive strategy in response to environmental conditions?	No	no evidence	Low
29	6.02	Is the taxon likely to produce viable propagules in the risk assessment area?	Yes	Vidaković and Franjić 2004	High
30	6.03	Is the taxon likely to hybridise with native species under natural conditions?	Yes	This species hybridize freely with other members of this genus (Vidaković and Franjić 2004, https://practicalplants.org/wiki/pinus_wallichiana/#Propagation).	High
31	6.04	Is the taxon likely to be hermaphroditic or to exhibit asexual reproduction?	No	no evidence	Low
32	6.05	Is the taxon dependent on the presence of another species (or specific habitat features) to complete its life cycle?	Yes	Suillus sibiricus, an edible fungus has been noted to be associated in large numbers with Pinus wallichiana plantations in north western Himalayan region indicating that it formes mycorrhiza with this tree (Sagar and Lakhanpal 2005).	Medium
33	6.06	Is the taxon likely to produce a large number of propagules?	No	professional judgement	Low
34	6.07	How many time units (days, months, years) does the taxon require to reach the age at first reproduction?	>10	min. 10 to 12 years	High
7. Dispersal mechanisms					
35	7.01	How many potential vectors or pathways could the taxon use to disperse within the risk assessment area (with suitable habitats nearby)?	>1	dispersion by humans (by cultivation), birds and small mammals (forage plant)	High
36	7.02	Will any of these vectors or pathways bring the taxon in close proximity to one or more protected areas?	Yes	humans and animals	High
37	7.03	Does the taxon have a specialised means of attachment such that it enhances the likelihood of dispersal?	No	no evidence	Low
38	7.04	Is natural dispersal of the taxon likely to occur as seeds or spores in the risk	Yes	Cones can be dispersed by wind and water.	High
39	7.05	Is natural dispersal of the taxon likely to occur as fragments or seedlings in the risk assessment area?	No	no evidence	Low
40	7.06	Are any life stages of the taxon likely to migrate into the risk assessment area for reproduction?	Yes	Cones can be dispersed by wind and water.	Medium
41	7.07	Are propagules of the taxon likely to be dispersed in the risk assessment area by other species?	Yes	Pinus wallichiana is forage plant for birds and small mammals.	Medium
42	7.08	Is dispersal of the taxon along any of the vectors or pathways mentioned in the previous seven Questions (35–41: i.e. either unintentional or intentional) likely to be	Yes	by animals	Low
43	7.09	Is dispersal of the taxon density dependent?	Yes	more trees have more seeds	Medium
8. Tolerance attributes					
44	8.01	Is the taxon able to withstand being in water for extended periods at some stage of its life cycle?	No	no evidence	Low
45	8.02	Is the taxon tolerant of a wide range of soil or air quality conditions?	No	https://www.ebben.nl/en/treeebb/piwallic-pinus-wallichiana/pdf/	Low
46	8.03	Can the taxon be controlled or eradicated in the wild with chemical, biological or other agents and means?	Yes	Tree management: https://apps.worldagroforestry.org/treedb/AFTPDFS/Pinus_wallichiana.PDF	Low
47	8.04	Is the taxon likely to benefit from environmental or human disturbance?	No	no evidence	Low
48	8.05	Is the taxon able to tolerate soil acidity, salinity or other parameter levels that are higher or lower than those found in its usual	No	no reference	Low
49	8.06	Are there effective natural enemies of the taxon present in the risk assessment area?	No	no evidence	Low
**C. Climate change**					
9. Climate change					
50	9.01	Under the predicted future climatic conditions, are the risks of entry into the risk assessment area posed by the taxon likely to increase, decrease or not change?	Decrease	professional judgement	Low

51	9.02	Under the predicted future climatic conditions, are the risks of establishment within the risk assessment area posed by the taxon likely to increase, decrease or not	Decrease	professional judgement	Low
52	9.03	Under the predicted future climatic conditions, are the risks of dispersal within the risk assessment area posed by the taxon likely to increase, decrease or not change?	Decrease	professional judgement	Low
53	9.04	Under the predicted future climatic conditions, what is the likely magnitude of future potential impacts by the taxon on biodiversity or ecosystem integrity and	Lower	professional judgement	Low
54	9.05	Under the predicted future climatic conditions, what is the likely magnitude of future potential impacts by the taxon on ecosystem structure or function?	Lower	professional judgement	Low
55	9.06	Under the predicted future climatic conditions, what is the likely magnitude of future potential impacts by the taxon on ecosystem services and related socio-	Lower	professional judgement	Low

Statistics	
**Scores**	
**BRA**	**13.0**
**BRA Outcome**	**Medium**
**BRA+CCA**	**1.0**
**BRA+CCA Outcome**	**Medium**
**Score partition**	
**A. Biogeography/Historical**	**5.0**
1. Domestication/Cultivation	*2.0*
2. Climate, distribution and introduction risk	*1.0*
3. Invasive elsewhere	*2.0*
**B. Biology/Ecology**	**8.0**
4. Undesirable (or persistence) traits	*8.0*
5. Resource exploitation	*0.0*
6. Reproduction	*-1.0*
7. Dispersal mechanisms	*5.0*
8. Tolerance attributes	*-4.0*
**C. Climate change**	**-12.0**
9. Climate change	*-12.0*
**Answered Questions**	
Total	***55***
**A. Biogeography/Historical**	**13**
1. Domestication/Cultivation	*3*
2. Climate, distribution and introduction risk	*5*
3. Invasive elsewhere	*5*
**B. Biology/Ecology**	**36**
4. Undesirable (or persistence) traits	*12*
5. Resource exploitation	*2*
6. Reproduction	*7*
7. Dispersal mechanisms	*9*
8. Tolerance attributes	*6*
**C. Climate change**	**6**
9. Climate change	*6*
**Sectors affected**	
**Commercial**	**4**
**Environmental**	**-1**
**Species or population nuisance traits**	**2**

Thresholds	
**BRA**	**41**
**BRA+CCA**	**41**
**Confidence**	
**BRA+CCA**	**0.39**
**BRA**	**0.41**
**CCA**	**0.25**

Date and Time
**27/06/2024 13:00:27**

TPS-ISK v2

**Taxon and Assessor details**	
Category	Gymnosperms
Taxon name	*Pseudotsuga menziesii*
Common name	Douglas Fir
Assessor	Mihaela Britvec
**Risk screening context**	
Reason and socio-economic benefits	
Risk assessment area	Continental Europe
Taxonomy	
Native range	
Introduced range	
URL	

			Response	Justification (references or other information)	Confidence
**A. Biogeography/Historical**					
1. Domestication/Cultivation					
1	1.01	Has the taxon been the subject of domestication?	Yes	Pseudotsuga menziesii was first introduced to Europe from North America more than 150 years ago, was then planted on a large scale and is now the economically most important non-native tree species in European forests (Schmid et al. 2014).	Very high
2	1.02	Is the taxon harvested in the wild and likely to be sold or used in its live form?	Yes	Pseudotsuga menziensii is a major species for afforestation in Western Europe after WWII. The species is generally regenerated by planting, using seedlings of European provenance derived from seed orchards or certified seed stands (Nicolescu et al 2023). The species has ornamental value in large parks and gardens. It has been commonly used as a Christmas tree since the 1920s, and the trees are typically grown on plantations.	Very high
3	1.03	Does the taxon have invasive races, varieties, sub-taxa or congeners?	Yes	There are two varieties: P. menziesii var. menziesii and P. menziesii var. glauca (Eckhart et al. 2019,	Medium
2. Climate, distribution and introduction risk					
4	2.01	How similar are the climatic conditions between the risk assessment area and the taxon's native range?	High	The natural distribution range of Douglas Fir extends along the entire Pacific coast of North America, from British Columbia to Mexico. It thrives in a wide range of climatic conditions (Levanič and Štraus 2022). Such climatic conditions can also be found in Continental Europe.	Very high
5	2.02	What is the quality of the climate matching data?	High	professional judgement	High
6	2.03	Is the taxon known to be present outside of cultivation in the risk assessment area?	Yes	Pseudotsuga menziesii has been planted extensively for forestry in many European countries (Broncano et al. 2005).	Very high
7	2.04	How many potential vectors could the taxon use to enter the risk assessment area?	>1	Douglas Fir seeds are an important food source for birds and small mammals such as white-footed deer mice, creeping voles, chipmunks, and shrews (Hermann ans Lavender 1990). Seeds are dispersed by wind, water, or humans.	Very high
8	2.05	Is the taxon currently found in close proximity to, and likely to enter, the risk assessment area in the near future (e.g. unintentional or intentional introductions)?	Not applicable	Pseudotsuga menziensii currently grows in Western Europe on over 0.83 million ha (Nicolescu et al 2023).	Very high
3. Invasive elsewhere					
9	3.01	Has the taxon become naturalised outside its native range?	Yes	Douglas Fir currently grows in 35 European countries and is one of the most widespread nonnative tree species across the continent (Nicolescu et al. 2023).	Very high
10	3.02	In the taxon's introduced range, are there any known adverse impacts to wild or commercial species?	Yes	Fungal diversity is reduced by Douglas Fir. Special microclimatic conditions in the crown of Douglas Fir can lead to reduced arthropod densities during winter with possible negative consequences for birds. Douglas Fir can negatively impact single groups of organisms or species and is now regenerating itself naturally in Europe. Although Douglas Fir has not been the subject of large-scale outbreaks of pests in Europe so far, the further introduction of exotic organisms associated with Douglas Fir in its native range could be more problematic than the introduction of Douglas Fir itself (Schmid et al. 2014).	Low
11	3.03	In the taxon's introduced range, are there any known adverse impacts to agriculture or forestry?	Yes	From an ecological point of view, cultivation of Douglas Fir in Europe is likely to have significant impacts on forest ecosystems, particularly in case of stands of pure Douglas fir and with high density of this species over large areas. Douglas Fir plantations are particularly deleterious from a nature conservation point of view when they replace species-rich and threatened ecosystems such as forest glades and heathlands (Schmid et al. 2014).	Medium
12	3.04	In the taxon's introduced range, are there any known adverse impacts to ecosystem	Yes	Fungal diversity is reduced by Douglas Fir (Schmid at al. 2014).	Medium
13	3.05	In the taxon's introduced range, are there any known adverse socio-economic impacts?	Yes	Douglas Fir can threaten landscape character and recreation value.	Low
**B. Biology/Ecology**					
4. Undesirable (or persistence) traits					
14	4.01	Is the taxon likely to be poisonous or pose other risks to human health?	Yes	https://www.wood-database.com/wood-articles/wood-allergies-and-toxicity/	Low
15	4.02	Is the taxon likely to suppress the growth of one or more native species?	Yes	Douglas Fir regenerates naturally especially on poor sites (dry, acidic), where it is not outcompeted by native tree species, but the ecological impacts of Douglas Fir seem to be minor compared to other non-native trees (e.g., Robinia pseudoacacia). Nevertheless, Douglas-fir can cause changes in species composition (Eckhart et al. 2019).	Medium

16	4.03	Are there any threatened or protected native species that the taxon would parasitise in the risk assessment area?	No	Douglas Fir is not known as parasitic plant.	High
17	4.04	Is the taxon adaptable in terms of climatic and other environmental conditions, thus enhancing its potential persistence if it has invaded or is likely to invade the risk	Yes	https://doi.org/10.1007/s11676-023-01607-4	Very high
18	4.05	Is the taxon likely to disrupt terrestrial ecosystem function if it has invaded or is likely to invade the risk assessment area?	Yes	doi: 10.3389/ffgc.2022.844580	Low
19	4.06	Is the taxon likely to exert adverse impacts on ecosystem services in the risk	Yes	doi: 10.3389/ffgc.2022.844580	Low
20	4.07	Is the taxon likely to host or function as a vector for recognised pests and pathogens that are present in the risk assessment area?	Yes	doi: 10.1007/s10342-013-0745-7	Low
21	4.08	Is the taxon likely to host or function as a vector for recognised pests and pathogens that are absent in the risk assessment area?	Yes	Although Douglas Fir has not been the subject of large-scale outbreaks of pests in Europe so far, the further introduction of exotic organisms associated with Douglas Fir in its native range could be more problematic than the introduction of Douglas Fir itself (Schmid et al. 2014).	Medium
22	4.09	Is the taxon likely to be released from cultivation?	Yes	Douglas Fir has high growth rates as well as rapid wound closure, good wood properties, resistance against the fungal pathogen and a low number of pests and diseases are factors that have contributed to the large present-day distribution of Douglas Fir in European coutries (Schmid et al. 2014).	Medium
23	4.10	Is the taxon versatile in habitat use?	Yes	European experience indicates that Douglas Fir can grow on a range of soil types apart from heavy soils with pseudogley close to the surface, on dry or heavily waterlogged soils where rooting is restricted, leading to instability, and on calcareous soils (Nicolescu et al. 2023).	High
24	4.11	Is it likely that the taxon's mode of existence or behaviours will reduce habitat quality for native species?	No	no reference	Low
25	4.12	Is the taxon likely to maintain a viable population even when present in low densities (or persist in adverse conditions by way of a dormant form)?	Yes	The natural regeneration of Douglas fir in Europe is not a local and recent phenomenon: it has already been reported from Austria, Bulgaria, France, Germany, Switzerland, Italy and the Czech Republic since the 1950s. Recently, natural regeneration of Douglas Fir was reported in Mediterranean mountain forests in Spain. The ability to regenerate naturally is important because it provides the possibility for Douglas Fir to increase its local abundance and regional distribution in Europe (Schmid et al.	High
5. Resource exploitation					
26	5.01	Is the taxon likely to put threatened or protected native species under competitive pressure in the risk assessment area?	Yes	In particular, the impact of tree species on the light supply in the understory is important for the understory vegetation (Schmid et al. 2014).	Medium
27	5.02	Is the taxon likely to sequester resources to the detriment of native species in the risk assessment area?	Yes	Analogously to soil chemistry, the overall soil, light, water and probably temperature conditions in Douglas Fir stands are not much different from those of native tree species (Schmid et al.	Medium
6. Reproduction					
28	6.01	Is the taxon likely to exhibit changes in reproductive strategy in response to environmental conditions?	Yes	An important characteristic of Douglas Fir is a greater tolerance of summer drought than many other species (Nicolescu et al. 2023).	Medium
29	6.02	Is the taxon likely to produce viable propagules in the risk assessment area?	Yes	doi: 10.3389/ffgc.2022.844580	Very high
30	6.03	Is the taxon likely to hybridise with native species under natural conditions?	No	no reference	Low
31	6.04	Is the taxon likely to be hermaphroditic or to exhibit asexual reproduction?	No	no evidence	Low
32	6.05	Is the taxon dependent on the presence of another species (or specific habitat features) to complete its life cycle?	Yes	Douglas Fir is dependent on a mycorrhizal relationship for efficient uptake of mineral nutrients and water (Hermann and Lavender 1990).	Very high
33	6.06	Is the taxon likely to produce a large number of propagules?	Yes	Seed production is often higher in years following a dry summer (Van Vredenburch and La Bastide 1968).	Low
34	6.07	How many time units (days, months, years) does the taxon require to reach the age at first reproduction?	>10	Douglas Fir trees start to produce seed cones at 30 − 35 years, with the best production occurring from 50 − 60 years (Nicolescu et al. 2023).	High
7. Dispersal mechanisms					
35	7.01	How many potential vectors or pathways could the taxon use to disperse within the risk assessment area (with suitable habitats nearby)?	>1	For example, seeds are spread by wind, by animals, and by humans. Douglas Fir is also planted by humans.	Very high
36	7.02	Will any of these vectors or pathways bring the taxon in close proximity to one or more protected areas?	Yes	The seeds are dispersed by wind for distances of 25 − 30 m up to 50 to 100 m or even 100 − 150 m (Nicolescu et al. 2023).	High
37	7.03	Does the taxon have a specialised means of attachment such that it enhances the likelihood of dispersal?	No	no evidence	Medium
38	7.04	Is natural dispersal of the taxon likely to occur as seeds or spores in the risk	Yes	The seeds are dispersed by wind (Nicolescu et al. 2023).	Very high
39	7.05	Is natural dispersal of the taxon likely to occur as fragments or seedlings in the risk assessment area?	Yes	Rooting cuttings is also a very popular form of propagation used in commercial settings. Cuttings is a good form of propagation in that it takes less time for the tree to grow and you can also choose cuttings based on favorable characteristics. However, the success of the cuttings rooting is very slim and it can often be expensive because you have to buy rooting hormones (Lemmon	Medium

40	7.06	Are any life stages of the taxon likely to migrate into the risk assessment area for reproduction?	Yes	The seeds are dispersed by wind for distances of 25 − 30 m up to 50 to 100 m or even 100 − 150 m (Nicolescu et al. 2023).	Very high
41	7.07	Are propagules of the taxon likely to be dispersed in the risk assessment area by other species?	No	no evidence	Low
42	7.08	Is dispersal of the taxon along any of the vectors or pathways mentioned in the previous seven Questions (35–41: i.e. either unintentional or intentional) likely to be	Yes	dispersal by wind, planting by humans	Medium
43	7.09	Is dispersal of the taxon density dependent?	No	no reference	Low
8. Tolerance attributes					
44	8.01	Is the taxon able to withstand being in water for extended periods at some stage of its life cycle?	No	professional judgement	Medium
45	8.02	Is the taxon tolerant of a wide range of soil or air quality conditions?	Yes	European experience indicates that Douglas Fir can grow on a range of soil types apart from heavy soils with pseudogley close to the surface, on dry or heavily waterlogged soils where rooting is restricted, leading to instability, and on calcareous soils. It grows best on deep, well aerated, moderately acidic (pH 5−6), free draining soils of loamy-sand or sandy-loam texture which allow good root development (Nicolescu et al. 2023).	Very high
46	8.03	Can the taxon be controlled or eradicated in the wild with chemical, biological or other agents and means?	Yes	doi 10.1186/s40490-016-0076-9	High
47	8.04	Is the taxon likely to benefit from environmental or human disturbance?	Yes	For example, fire is an important disturbance affecting the dynamics of stands in its native range where moderate severe fires allow thicker bark Douglas Fir to survive and regenerate at the expense of thin bark species of lower fire resistance. Clear-cutting are also being carried out in different European	Medium
48	8.05	Is the taxon able to tolerate soil acidity, salinity or other parameter levels that are higher or lower than those found in its usual	No	When Psuedotsuga menziensii grown on shallow or poorly drained soils, plate-like root systems develop. Under such conditions, the species is prone to windthrow (Nicolescu et al.).	Low
49	8.06	Are there effective natural enemies of the taxon present in the risk assessment area?	No	doi.org/10.1007/s11676-023-01607-4	Medium
**C. Climate change**					
9. Climate change					
50	9.01	Under the predicted future climatic conditions, are the risks of entry into the risk assessment area posed by the taxon likely to increase, decrease or not change?	Increase	In recent decades, above-average summer precipitation has also had a positive influence on the radial growth of Douglas Fir. Douglas Fir is not very sensitive to extreme drought events (Levanič and Štraus 2022).	Medium
51	9.02	Under the predicted future climatic conditions, are the risks of establishment within the risk assessment area posed by the taxon likely to increase, decrease or not	Increase	professional judgement	Low
52	9.03	Under the predicted future climatic conditions, are the risks of dispersal within the risk assessment area posed by the taxon likely to increase, decrease or not change?	Increase	professional judgement	Low
53	9.04	Under the predicted future climatic conditions, what is the likely magnitude of future potential impacts by the taxon on biodiversity or ecosystem integrity and status?	Higher	Nevertheless, climate change as well as globalization may have implications for the management of Douglas Fir, for example if biotic pests from its native range are introduced and become adapted to Douglas Fir in Europe. Under anticipated climate change, a major problem will be to identify provenances best adapted to future climatic conditions. This could require the use of new seed sources and provenances from its natural range, adapted to drier conditions such as those from southern Oregon and northern California (Nicolescu et al. 2023).	Medium
54	9.05	Under the predicted future climatic conditions, what is the likely magnitude of future potential impacts by the taxon on ecosystem structure or function?	Higher	Under anticipated climate change, a major problem will be if biotic pests from its native range are introduced and become adapted to Douglas Fir in Europe (Nicolescu et al. 2023).	Medium
55	9.06	Under the predicted future climatic conditions, what is the likely magnitude of future potential impacts by the taxon on ecosystem services and related socio-	Higher	professional judgement	Low

**Statistics**	
**Scores**	
**BRA**	**46.0**
**BRA Outcome**	**High**
**BRA+CCA**	**58.0**
**BRA+CCA Outcome**	**High**
**Score partition**	
**A. Biogeography/Historical**	**24.0**
1. Domestication/Cultivation	*4.0*
2. Climate, distribution and introduction risk	*2.0*
3. Invasive elsewhere	*18.0*
**B. Biology/Ecology**	**22.0**
4. Undesirable (or persistence) traits	*10.0*
5. Resource exploitation	*7.0*
6. Reproduction	*-1.0*
7. Dispersal mechanisms	*4.0*
8. Tolerance attributes	*2.0*
**C. Climate change**	**12.0**

9. Climate change	*12.0*
**Answered Questions**	
Total	***55***
**A. Biogeography/Historical**	**13**
1. Domestication/Cultivation	*3*
2. Climate, distribution and introduction risk	*5*
3. Invasive elsewhere	*5*
**B. Biology/Ecology**	**36**
4. Undesirable (or persistence) traits	*12*
5. Resource exploitation	*2*
6. Reproduction	*7*
7. Dispersal mechanisms	*9*
8. Tolerance attributes	*6*
**C. Climate change**	**6**
9. Climate change	*6*
**Sectors affected**	
**Commercial**	**21**
**Environmental**	**16**
**Species or population nuisance traits**	**26**

**Thresholds**	
**BRA**	**41**
**BRA+CCA**	**41**
**Confidence**	
**BRA+CCA**	**0.59**
**BRA**	**0.61**
**CCA**	**0.38**

**Date and Time**	
**18/06/2024 09:52:49**	

TPS-ISK v2

Taxon and Assessor details	
Category	Gymnosperms
Taxon name	*Quercus rubra*
Common name	Northern Red Oak
Assessor	Mihaela Britvec
**Risk screening context**	
Reason and socio-economic benefits	
Risk assessment area	Continental Europe
Taxonomy	
Native range	
Introduced range	
URL	

			Response	Justification (references or other information)	Confidence
**A. Biogeography/Historical**					
1. Domestication/Cultivation					
1	1.01	Has the taxon been the subject of domestication?	Yes	Northern Red Oak (Quercus rubra) is a valuable broadleaved tree species originating from the eastern half of the United States and southeast and south-central Canada. The species was introduced in Europe at the end of the seventeenth century, the frst record comes from France in 1691. Iit was planted mainly as an ornamental tree because it is very attractive with its red leaves in the autumn (https://doi.org/10.1007/s11756-023-01502-7).	Very high
2	1.02	Is the taxon harvested in the wild and likely to be sold or used in its live form?	Yes	As Quercus rubra has a large crown and crimson-coloured leaves, almost entirely marcescent on young trees in the winter, with a high landscape and ornamental value, and good resistance to urban pollution, the species is used in parks, public gardens and is a common street tree (doi:10.1093/forestry/cpy032).	Very high
3	1.03	Does the taxon have invasive races, varieties, sub-taxa or congeners?	Yes	For example: Quercus cerris in Poland (https://www.nobanis.org/species-info/?taxaId=2186).	Very high
2. Climate, distribution and introduction risk					
4	2.01	How similar are the climatic conditions between the risk assessment area and the taxon's native range?	High	doi:10.1093/forestry/cpy032	High
5	2.02	What is the quality of the climate matching data?	High	doi:10.1093/forestry/cpy032	High
6	2.03	Is the taxon known to be present outside of cultivation in the risk assessment area?	Yes	Franjić and Škvorc (2010), https://www.gbif.org/species/2880539	High
7	2.04	How many potential vectors could the taxon use to enter the risk assessment area?	>1	Seeds of Northern Red Oak are primarily dispersed by birds and mammals (https://www.fs.usda.gov/database/feis/plants/tree/querub/all.html#DISTRIBUTION%20AND%20OCCURRENCE). The presence of this tree species is also the result of cultivation and its further spontaneous spread (doi:10.3390/f11050586).	Very high
8	2.05	Is the taxon currently found in close proximity to, and likely to enter, the risk assessment area in the near future (e.g. unintentional or intentional introductions)?	Not applicable	doi:10.1093/forestry/cpy032	Very high
3. Invasive elsewhere					
9	3.01	Has the taxon become naturalised outside its native range?	Yes	For example: https://hirc.botanic.hr/fcd/DetaljiFrame.aspx?IdVrste=8268&taxon=Quercus+rubra+L.	Very high
10	3.02	In the taxon's introduced range, are there any known adverse impacts to wild or commercial species?	Yes	Quercus rubra significantly reduces native species richness and abundance, both in old-growth and in secondary (post-agricultural) forests (doi: 10.5586/asbp.2013.035).	Very high
11	3.03	In the taxon's introduced range, are there any known adverse impacts to agriculture or forestry?	Yes	The natural restocking of all native woody species is strongly limited by Quercus rubra (doi: 10.5586/asbp.2013.035).	High
12	3.04	In the taxon's introduced range, are there any known adverse impacts to ecosystem services?	Yes	Studies show that Quercus rubra introduction in Querco roboris-Pinetum definitely decreases supporting ecosystem services (doi: 10.5586/asbp.2013.035).	High
13	3.05	In the taxon's introduced range, are there any known adverse socio-economic impacts?	Yes	Northern Red Oak significantly limits the environmental functions of the Querco roboris-Pinetum ecosystem and weakens its economic and social aspects (doi: 10.5586/asbp.2013.035).	High
**B. Biology/Ecology**					
4. Undesirable (or persistence) traits					
14	4.01	Is the taxon likely to be poisonous or pose other risks to human health?	Yes	Northern Red Oak pollen is an important cause of allergic respiratory (http://doi.org/10.1016/j.waojou.2020.100111).	High
15	4.02	Is the taxon likely to suppress the growth of one or more native species?	Yes	Not one resident vascular plant benefits from the introduction of Northern Red Oak and only a few are able to tolerate its co-occurrence (doi: 10.5586/asbp.2013.035).	High
16	4.03	Are there any threatened or protected native species that the taxon would parasitise in the risk assessment area?	No	Quercus rubra is not a parasitic plant.	High
17	4.04	Is the taxon adaptable in terms of climatic and other environmental conditions, thus enhancing its potential persistence if it has invaded or is likely to invade the risk	Yes	The adaptation potential of Northern Red Oak to predicted climate change, especially drought, seems to be higher than for European native oaks (doi:10.1093/forestry/cpy032).	Medium
18	4.05	Is the taxon likely to disrupt terrestrial ecosystem function if it has invaded or is likely to invade the risk assessment area?	Yes	doi: 10.5586/asbp.2013.035	High
19	4.06	Is the taxon likely to exert adverse impacts on ecosystem services in the risk	Yes	doi: 10.5586/asbp.2013.035	High

20	4.07	Is the taxon likely to host or function as a vector for recognised pests and pathogens that are present in the risk assessment area?	Yes	Many fungal pathogens and insect pests have been found in Northern Red Oak stands across Europe (doi:10.1093/forestry/cpy032).	Medium
21	4.08	Is the taxon likely to host or function as a vector for recognised pests and pathogens that are absent in the risk assessment area?	Yes	The species can be affected by pests and diseases (Gilman and Watson 1994).	Medium
22	4.09	Is the taxon likely to be released from cultivation?	Yes	Northern Red Oak is a fastgrowing species (doi:10.1093/forestry/cpy032).	High
23	4.10	Is the taxon versatile in habitat use?	Yes	Quercus rubra can grow on poor, sandy and acid soils, if they are not too dry, as well as on heavy-textured, clay-rich (loamy-clay and clayey) soils (doi:10.1093/forestry/cpy032).	Medium
24	4.11	Is it likely that the taxon's mode of existence or behaviours will reduce habitat quality for native species?	No	no evidence	Low
25	4.12	Is the taxon likely to maintain a viable population even when present in low densities (or persist in adverse conditions by way of a dormant form)?	Yes	The Northern Red Oak has also developed tolerance mechanisms for heat stress, particularly observed in deciduous forests in the Southeastern United States, where, during summer heat waves, temperatures can exceed 40 °C. The leaves of the Northern Red Oak have been observed to have an acclimation to Rubisco activase activity that is directly correlated to acclimations with repeated exposure to heat waves. Consistent photosynthetic activity in the Northern Red Oak has also been observed in the presence of high carbon dioxide levels that often occur as a result of elevated temperatures (doi:10.1007/s11258-014-0352-9).	Medium
5. Resource exploitation					
26	5.01	Is the taxon likely to put threatened or protected native species under competitive pressure in the risk assessment area?	Yes	https://doi.org/10.1007/s11756-023-01502-7	Medium
27	5.02	Is the taxon likely to sequester resources to the detriment of native species in the risk assessment area?	Yes	professional judgement	Low
6. Reproduction					
28	6.01	Is the taxon likely to exhibit changes in reproductive strategy in response to environmental conditions?	Yes	Seedlings and young trees sprout prolifically also when killed by fire (doi:10.1093/forestry/cpy032).	Low
29	6.02	Is the taxon likely to produce viable propagules in the risk assessment area?	Yes	doi:10.1093/forestry/cpy032	Very high
30	6.03	Is the taxon likely to hybridise with native species under natural conditions?	Yes	Northern Red Oak hybridizes readily with other species in the subgenus Erythrobalanus (https://www.srs.fs.usda.gov/pubs/misc/ag_654/volume_2/quercus/rubra.htm).	Medium
31	6.04	Is the taxon likely to be hermaphroditic or to exhibit asexual reproduction?	Yes	by root stem cuttings (Stanturf, John A., ed. 2010. Proceedings of the 14th biennial southern silvicultural research conference. Gen. Tech. Rep. SRS-121. Asheville, NC: U.S. Department of Agriculture, Forest Service, Southern Research Station. 614 p.).	Medium
32	6.05	Is the taxon dependent on the presence of another species (or specific habitat features) to complete its life cycle?	No	no evidence	Low
33	6.06	Is the taxon likely to produce a large number of propagules?	Yes	https://www.srs.fs.usda.gov/pubs/misc/ag_654/volume_2/quercus/rubra.htm	Medium
34	6.07	How many time units (days, months, years) does the taxon require to reach the age at first reproduction?	>10	about 20–30 years	Very high
7. Dispersal mechanisms					
35	7.01	How many potential vectors or pathways could the taxon use to disperse within the risk assessment area (with suitable habitats nearby)?	>1	Seeds of Northern Red Oak are primarily dispersed by birds and mammals. The presence of this tree species is also the result of cultivation and its further spontaneous spread by birds and mammals.	Very high
36	7.02	Will any of these vectors or pathways bring the taxon in close proximity to one or more protected areas?	Yes	birds and mammals (doi:10.1093/forestry/cpy032)	Very high
37	7.03	Does the taxon have a specialised means of attachment such that it enhances the likelihood of dispersal?	No	no evidence	Medium
38	7.04	Is natural dispersal of the taxon likely to occur as seeds or spores in the risk	Yes	doi:10.1093/forestry/cpy032	Very high
39	7.05	Is natural dispersal of the taxon likely to occur as fragments or seedlings in the risk assessment area?	Yes	doi:10.1093/forestry/cpy032	Low
40	7.06	Are any life stages of the taxon likely to migrate into the risk assessment area for reproduction?	Yes	For example: by water and animals (https://www.srs.fs.usda.gov/pubs/misc/ag_654/volume_2/quercus/rubra.htm)	Medium
41	7.07	Are propagules of the taxon likely to be dispersed in the risk assessment area by other species?	Yes	The acorns are dispersed by birds and mammals (doi:10.1093/forestry/cpy032).	Very high
42	7.08	Is dispersal of the taxon along any of the vectors or pathways mentioned in the previous seven Questions (35–41: i.e. either unintentional or intentional) likely to be	Yes	doi:10.1093/forestry/cpy032	Very high
43	7.09	Is dispersal of the taxon density dependent?	Yes	more trees have more acorns	Low
8. Tolerance attributes					
44	8.01	Is the taxon able to withstand being in water for extended periods at some stage of its life cycle?	Yes	professional judgement	Low

45	8.02	Is the taxon tolerant of a wide range of soil or air quality conditions?	Yes	Northern Red Oak has better resistance to soil drought than native sessile oak and pedunculate oak and therefore can be more vigorous and productive on less fertile sites	Medium
46	8.03	Can the taxon be controlled or eradicated in the wild with chemical, biological or other agents and means?	Yes	For examples: exposure to herbicides (Samtani et al. 2010)	Low
47	8.04	Is the taxon likely to benefit from environmental or human disturbance?	Yes	Young Northern Red Oaks commonly sprout vigorously from the stumps or root collar after aboveground portions of the plant are killed by fire (https://www.fs.usda.gov/database/feis/plants/tree/querub/all.html#FIRE%20EFFECTS).	Medium
48	8.05	Is the taxon able to tolerate soil acidity, salinity or other parameter levels that are higher or lower than those found in its usual	Yes	Its soil salt tolerance is good (Gilman and Watson 1994).	Low
49	8.06	Are there effective natural enemies of the taxon present in the risk assessment area?	No	Many fungal pathogens and insect pests have been found in Northern Red Oak stands across Europe, but so far none of them have been reported to cause serious damages to the species (doi:10.1093/forestry/cpy032).	Low
**C. Climate change**					
9. Climate change					
50	9.01	Under the predicted future climatic conditions, are the risks of entry into the risk assessment area posed by the taxon likely to increase, decrease or not change?	Increase	The adaptation potential of Northern Red Oak to predicted climate change, especially drought, seems to be higher than for European native oaks, the importance of the species is expected to increase in the future (doi:10.1093/forestry/cpy032).	High
51	9.02	Under the predicted future climatic conditions, are the risks of establishment within the risk assessment area posed by the taxon likely to increase, decrease or not	Increase	doi:10.1093/forestry/cpy032	High
52	9.03	Under the predicted future climatic conditions, are the risks of dispersal within the risk assessment area posed by the taxon likely to increase, decrease or not change?	Increase	doi:10.1093/forestry/cpy032	High
53	9.04	Under the predicted future climatic conditions, what is the likely magnitude of future potential impacts by the taxon on biodiversity or ecosystem integrity and	Higher	professional judgement	Medium
54	9.05	Under the predicted future climatic conditions, what is the likely magnitude of future potential impacts by the taxon on ecosystem structure or function?	Higher	professional judgement	Medium
55	9.06	Under the predicted future climatic conditions, what is the likely magnitude of future potential impacts by the taxon on ecosystem services and related socio-	Higher	professional judgement	Medium

**Statistics**	
**Scores**	
**BRA**	**60.0**
**BRA Outcome**	**High**
**BRA+CCA**	**72.0**
**BRA+CCA Outcome**	**High**
**Score partition**	
**A. Biogeography/Historical**	**24.0**
1. Domestication/Cultivation	*4.0*
2. Climate, distribution and introduction risk	*2.0*
3. Invasive elsewhere	*18.0*
**B. Biology/Ecology**	**36.0**
4. Undesirable (or persistence) traits	*10.0*
5. Resource exploitation	*7.0*
6. Reproduction	*5.0*
7. Dispersal mechanisms	*7.0*
8. Tolerance attributes	*7.0*
**C. Climate change**	**12.0**
9. Climate change	*12.0*
**Answered Questions**	
Total	***55***
**A. Biogeography/Historical**	**13**
1. Domestication/Cultivation	*3*
2. Climate, distribution and introduction risk	*5*
3. Invasive elsewhere	*5*
**B. Biology/Ecology**	**36**
4. Undesirable (or persistence) traits	*12*
5. Resource exploitation	*2*
6. Reproduction	*7*
7. Dispersal mechanisms	*9*
8. Tolerance attributes	*6*
**C. Climate change**	**6**
9. Climate change	*6*
**Sectors affected**	
**Commercial**	**21**
**Environmental**	**16**
**Species or population nuisance traits**	**40**

**Thresholds**	
**BRA**	**41**

BRA+CCA	41
**Confidence**	
BRA+CCA	0.65
BRA	0.65
CCA	0.63

Date and Time
04/07/2024 13:15:39
